# Behavioral regulation by perineuronal nets in the prefrontal cortex of the CNTNAP2 mouse model of autism spectrum disorder

**DOI:** 10.3389/fnbeh.2023.1114789

**Published:** 2023-03-14

**Authors:** Tanya Gandhi, Chin-Chi Liu, Tolulope T. Adeyelu, Cade R. Canepa, Charles C. Lee

**Affiliations:** ^1^Department of Comparative Biomedical Sciences, Louisiana State University School of Veterinary Medicine, Baton Rouge, LA, United States; ^2^Department of Veterinary Clinical Sciences, Louisiana State University School of Veterinary Medicine, Baton Rouge, LA, United States

**Keywords:** perineuronal nets (PNNs), repetitive behavior, CNTNAP2 associated neurodevelopmental disorders, autism model, neural mechanisms, neuroanatomical alterations, extracellular matrix, social behavior

## Abstract

Autism spectrum disorders (ASDs) arise from altered development of the central nervous system, and manifest behaviorally as social interaction deficits and restricted and repetitive behaviors. Alterations to parvalbumin (PV) expressing interneurons have been implicated in the neuropathological and behavioral deficits in autism. In addition, perineuronal nets (PNNs), specialized extracellular matrix structures that enwrap the PV-expressing neurons, also may be altered, which compromises neuronal function and susceptibility to oxidative stress. In particular, the prefrontal cortex (PFC), which regulates several core autistic traits, relies on the normal organization of PNNs and PV-expressing cells, as well as other neural circuit elements. Consequently, we investigated whether PNNs and PV-expressing cells were altered in the PFC of the CNTNAP2 knockout mouse model of ASD and whether these contributed to core autistic-like behaviors in this model system. We observed an overexpression of PNNs, PV-expressing cells, and PNNs enwrapping PV-expressing cells in adult CNTNAP2 mice. Transient digestion of PNNs from the prefrontal cortex (PFC) by injection of chondroitinase ABC in CNTNAP2 mutant mice rescued some of the social interaction deficits, but not the restricted and repetitive behaviors. These findings suggest that the neurobiological regulation of PNNs and PVs in the PFC contribute to social interaction behaviors in neurological disorders including autism.

## 1. Introduction

Autism spectrum disorder (ASD) as initially described by Kanner (1943) results from developmental and environmental alterations to normal brain structure. The prevalence of autistic cases has substantially increased through improved diagnostics and an expansion of inclusion criteria, worldwide around 1 in 154 individuals (Kassim and Mohamed, 2019; Lord et al., 2020; Zeidan et al., 2022) and in the United States 1 in 54 people (GuiFeng et al., 2018; Zablotsky et al., 2019; Maenner et al., 2020, 2021). The disorder affects core domains of social behavior and communication along with characteristic repetitive and restricted behaviors (Osterling and Dawson, 1994; Baranek, 1999; Lord et al., 2000; Wetherby et al., 2004). In addition, those with autism often exhibit a variety of co-morbid neurological disorders such as intellectual disability, epilepsy, mood, and anxiety disorders (Tuchman and Rapin, 2002; DiCicco-Bloom et al., 2006; Fombonne, 2006; Lecavalier, 2006; Richler et al., 2007; King et al., 2009). Pharmacological, psychological, and behavioral interventions for people with ASD target specific behavioral features and associated symptoms (Eissa et al., 2018; Chahin et al., 2020). However, more robust therapeutic interventions are needed that target the underlying mechanisms and address the core autistic behaviors (GuiFeng et al., 2018; Sheldrick and Carter, 2018).

Genetic and environmental factors contribute to the emergence of ASD (Muhle et al., 2004; Levitt and Campbell, 2009; Satterstrom et al., 2020). Among the environmental factors, prenatal and postnatal infections, nutritional deficiencies, and exposure to neurotoxic chemicals are implicated in the development of ASD (Grabrucker, 2013; Sealey et al., 2016; Karimi et al., 2017). Autistic outcomes are also strongly linked to genetic mutations (Ronemus et al., 2014), with a higher concordance rate in siblings and twins (Murphy et al., 2000; Geschwind, 2011; Stubbs et al., 2016; Eissa et al., 2018; Castelbaum et al., 2020). Several genes are involved in the etiological heterogeneity of autism. These genes affect neural development via altered structure, connectivity, excitatory/inhibitory balance and varied neurodevelopmental signaling pathways during early postnatal periods (Muhle et al., 2004; Levitt and Campbell, 2009; Satterstrom et al., 2020).

Among the genetic factors, common variations in the contactin-associated protein-like 2 (CNTNAP2) gene encode altered forms of the related protein (CASPR2), which is associated with ASD (Strauss et al., 2006; Alarcón et al., 2008; Arking et al., 2008; Bakkaloglu et al., 2008; Penagarikano et al., 2011). Mouse models with the CNTNAP2 mutation display several phenotypic characteristics of ASD-like deficits in social and communication behavior along with repetitive and hyperactive behaviors. In addition, the CNTNAP2 mutant mice manifest abnormal neuronal migration, decreased cortical inhibitory interneuron number, and aberrant cortical network activity (Penagarikano et al., 2011; Peñagarikano and Geschwind, 2012). Further, imaging studies in people with the CNTNAP2 mutation demonstrate alterations in brain structure and functional connectivity (Scott-Van Zeeland et al., 2010; Dennis et al., 2011). CNTNAP2 is enriched in several cortical circuits that are also crucial to language development and processing (Toma et al., 2013). Hence, CNTNAP2 is important for the development of brain regions involved in social and linguistic processing and its mutation results in aberrant neural development and processing, thus contributing to the autistic phenotype.

Alterations to normal physiology and behavior in CNTNAP2 mice emerges partially due to imbalanced cortical excitation and inhibition (E/I) (Gandhi and Lee, 2021). This E/I balance is regulated by excitatory glutamatergic neurons and inhibitory GABAergic interneurons, resulting in propagation and synchronization of complex neural activity underlying normal cognition and behavior (Canitano and Pallagrosi, 2017). Glutamatergic and GABAergic pathways are also crucial during neural development, mediating the migration and positioning of pyramidal cells and interneurons to facilitate cortical organization (Manent and Represa, 2007).

In this regard, an important sub-population of GABAergic cells in the neocortex is the group of fast-spiking interneurons that express the calcium binding protein, parvalbumin (PV), which is necessary for fast rhythmic neuronal synchrony (Sohal et al., 2009; Whittington et al., 2011). PV-positive neurons are sensitive to oxidative stress due to their fast-spiking properties that impose high metabolic demand leading to potential physiological impairments (Hu et al., 2010; Cabungcal et al., 2013b). PV-positive neurons mature in parallel with the assembly of specialized extracellular matrix structures around them, such as perineuronal nets (PNNs), as the critical period ends (Pizzorusso et al., 2002; Shen, 2018). PNNs provide neuroprotection against oxidative damage due to high metabolic activity of PV-positive interneurons and regulate PV cell synaptic and network stability (Morawski et al., 2004; Sugiyama et al., 2009; Kwok et al., 2011; Suttkus et al., 2012; Cabungcal et al., 2013a,b; Reichelt et al., 2019). Consequently, alterations to PNN structure and function negatively impacts inhibitory PV-positive neuronal activity, leading to E/I imbalance.

Therefore, in this study, we assessed whether alterations in PNNs and PV-positive cells in the prefrontal cortex (PFC) of CNTNAP2^–/–^ mice could underlie autistic-like behaviors in adult mice. We characterized the formation of PNNs around PV interneurons in young (PD30), adult (PD60), and aged (PD395-P425) CNTNAP2^–/–^ and wild-type (WT) mice. We then assessed the effects of enzymatic removal of PNNs in the PFC on behaviors associated with the core deficits in ASD. Overall, we find that altered PNN expression in the PFC likely contributes to alterations in social, but not restricted and repetitive, behaviors in the CNTNAP2^–/–^ mouse model of ASD, as detailed below.

## 2. Materials and methods

### 2.1. Animal care and housing

Wild-type (WT) C57BL/6J (strain: 000664) and CNTNAP2 mutant (strain: 017482) mouse breeder pairs were procured from the Jackson Laboratory (Bar Harbor, ME, USA). Offspring of the breeders were used in the study. All protocols were authorized by the Institutional Animal Care and Use Committee (IACUC) of the Louisiana State University (Baton Rouge, LA, USA). Mice were housed in a temperature and humidity-controlled room with a 12 h light/dark cycle with lights on at 7:00 am and food and water provided *ad libitum*.

### 2.2. Histology

To examine the expression of PNNs and PVs in the PFC, mice were first anesthetized via inhalation of isoflurane until cessation of reflex responses. Anesthetized mice were then transcardially perfused with 1X phosphate buffered saline (PBS) at a concentration of 10 mM (pH 7.4). Mice were then perfused with 4% paraformaldehyde solution (PFA) (diluted from 32% stock solution in 1× PBS) (#15714, Electron Microscopy Sciences, PA, USA). The whole brain samples were harvested and placed in 4% PFA solution overnight for post-fixation of the sample at 4°C. Samples were transferred the next day to a 4% PFA solution containing 30% sucrose for cryopreservation and then stored at 4°C. Brain sections were prepared coronally at 40 μm thickness by cryosectioning on a freezing microtome (cryostat). Sections were collected in 24 well plates containing 1X PBS (pH 7.4) and processed further for immunostaining.

Free-floating sections containing the prefrontal cortex (PFC) were stained for PNN and PV expression. Sections were first stained for PNNs by rinsing three times (5 min each) in 1X PBS (10 mM) solution, then incubating in Avidin D and biotin solutions (15 min each) to block all biotin and streptavidin binding sites (#SP-2002, Vector Laboratories, Burlingame, CA, USA). Next, sections were washed three times in 1X PBS before incubation with biotinylated WFA/WFL (Wisteria floribunda agglutinin/lectin) (#B-1355, Vector Laboratories, Burlingame, CA, USA) solution at 19.8 μg/ml (dilution 1:500) in 1X PBS (10 mM) overnight at room temperature. Finally, sections were washed three times in 1X PBS before incubation with the streptavidin conjugated with Alexa Fluor 568 (CF-29035, Biotium, CA, USA) at a dilution of 1:500 in 1X PBS solution for 1 hour at room temperature and protected from light.

For the immunostaining for PV-positive neurons, sections were rinsed in 1X PBS and blocked using normal goat serum (NGS) (#S-1000 Vector Laboratories, Burlingame, CA, USA) and 0.03% Triton X-100 for 1 h at room temperature. Following blocking, sections were incubated overnight at 4°C in primary antibody solution i.e., rabbit polyclonal anti-parvalbumin antibody (#ab11427, Abcam, Boston, MA, USA) at a dilution of 1:500 in blocking solution. Sections were washed two times before incubation with secondary goat anti-rabbit Alexa Fluor 488 pre-adsorbed antibody (#ab150081, Abcam, Boston, MA, USA) at a dilution 1:500 for 1 h at room temperature. Subsequently, sections were rinsed in 1×X PBS (three times for 5 min each). After rinsing, sections were mounted on gelatin slides and cover-slipped using Vectashield anti-fade mounting media (#H-1400, Vector Laboratories, Burlingame, CA, USA).

### 2.3. Imaging and quantification

Fluorescent images were obtained using a Nanozoomer whole slide scanner (Hamamatsu Photonics, Bridgewater, NJ, USA) with semi-automatic settings. Whole slide scan image data was viewed, analyzed, and quantitated using NDP2 software (Hamamatsu Photonics, Bridgewater, NJ, USA) and ImageJ (NIH, Bethesda, MD). PNNs and PV-positive cells were counted in a blinded and randomized manner to the observed image data. A standard atlas was used to identify the PFC regions of interest (ROIs) and borders based on cytoarchitecture and gross morphology (Paxinos and Franklin, 2019). Cell counting was performed manually by an investigator blinded to the identity of the specimens in a defined area over the ROIs PNNs and PV-positive cells were quantified by criteria of well-defined shape and developed form. PNNs, PV-positive neurons and PNNs co-localized with PV-positive neurons were counted separately in the determined area over the ROIs. An average of 3–4 ROIs were quantified per sample. Statistical analysis was conducted using GraphPad software (San Diego, CA, USA). Unpaired student’*s t*-tests were used to determine statistical difference in the stereological data with *p*-value < 0.05 to be considered statistically significant. The data is visually presented in the form of bar plots and/or scatter line plots with variability within the sample shown by standard error of mean (SEM).

### 2.4. Stereotactic surgery and enzymatic digestion of PNNs

Stereotactic surgery was conducted in adult mice by first anesthetizing with intraperitoneal injections of a ketamine (100 mg/kg body weight) and xylazine (20 mg/kg body weight) cocktail at 30 mg/kg concentration. Loss of consciousness of the animal was confirmed with no reciprocation on the toe pinch withdrawal reflex. Eyedrops were applied to protect and lubricate the eyes. Next, the head of the animal was adjusted and fixed in a mouse stereotactic instrument (Stoelting, Wood Dale, IL, USA). After shaving and cleaning the scalp with 70% alcohol and betadine, an incision was made across the midline of the scalp. A cotton tipped applicator soaked in 3% hydrogen peroxide solution was used to clean the skull surface to enhance visibility of the bregma. Stereotactic coordinates were marked on the cleaned skull surface in reference to bregma position. Craniotomy above the injection sites was performed using a dental micro-drill.

To digest PNN components, protease-free chondroitinase ABC (ChABC) (#C3667, MilliporeSigma, MA, USA) was stereotaxically injected in the prefrontal cortex (PFC). Penicillinase (#P0389, Sigma Aldrich, MA, USA) was used as a control for ChABC in the study. ChABC was diluted to a 0.05 U/μl concentration (Lensjø et al., 2017) and penicillinase was diluted to a concentration of 50 U/μl in filtered 1× PBS. The coordinates of the prefrontal cortical region were determined from a standard atlas (Paxinos and Franklin, 2019). Bilateral injections were performed in the PFC region targeting the following coordinates: anterior-posterior (AP) + 2.1 mm; mediolateral (ML) ± 0.4 mm and ventral (V) - 1.4 mm. A 5 μl Neuros syringe with a 33-gauge needle (Hamilton, Reno, NV, USA) was utilized to inject 1 μl of diluted ChABC or penicillinase solution into the injection site at a rate of 0.04 μl/min using a micro syringe pump (World Precision Instruments, Sarasota, FL, USA). The needle was slowly inserted to the injection site and remained in place at the injection site for a period of 3 min following the injection to allow for equilibration of the solution prior to removal.

After the injection, the surgical incised area was cleaned and sutured. Post-operative generic antibiotic cream was applied to prevent infections over the suture site. Next, buprenorphine was given intraperitoneally at a 0.1 mg/kg concentration to manage the pain following craniotomy and during the recovery period. The animals were monitored during the post-operative period of 7 days before the behavioral testing for any signs of distress and infection. Following the recovery period, behavioral testing was conducted as described below. Following the above-mentioned experiments, the animals were sacrificed with a time gap of 18–20 days after surgery involving chondroitinase and/or penicillinase injections to accommodate additional experiments described elsewhere (Gandhi, 2022).

### 2.5. Behavioral tests

Mice were subjected to behavioral testing relevant to core behavioral domains of ASD, including deficits in sociability and communication and repetitive behaviors (Gandhi, 2022). The behavioral testing was carried out before and after the stereotactic injection of ChABC to determine the outcome of temporary PNN digestion on behavioral alterations in these mice. We performed the tests 7 days after the ChABC injection; PNNs begin to reappear after this time period (Lensjø et al., 2017).

#### 2.5.1. Self-grooming

To assess repetitive self-grooming behavior, the subject mice were acclimated to the testing room 2 days prior to the test and 30 min before the start of the test. The subject mouse was habituated to the test apparatus, a standard empty mouse cage for 5 min. Self-grooming was recorded for the next 5 min using a camera (1080 HD) (Logitech, CA, USA) placed above a standard mouse cage containing the subject mouse. The test apparatus was cleaned with 70% ethanol to remove any residual odors before starting the next test. The video recording was analyzed manually in a randomized manner utilizing MATLAB software (MathWorks, USA). Stereotypic behaviors evaluated include repeated self-grooming and vertical rearing i.e., time spent by the mice climbing and/or jumping on the side walls of the test apparatus.

#### 2.5.2. Three-chamber social interaction and social novelty test

The three-chamber social interaction test assesses time spent by the subject mice with stranger mice (Moy et al., 2004, 2008; Kaidanovich-Beilin et al., 2011; Yang et al., 2011). Test mice were acclimated to the testing environment for two days before the test. On the day of the test, they were further acclimated to the surroundings for 30 min prior to testing. The stranger mice were familiarized to an upside-down holding cup in the side chambers of the 3-chamber apparatus for 15 min every day for 2 days prior to the test. This ensured that the stranger mice would become adapted to sitting quietly in the inverted holding cup during the testing duration. During habituation, the stranger mice were placed in the left and right sides of the chamber alternatively to avoid inclination toward any one side of the chamber apparatus.

The testing comprised of the following three phases: center chamber habituation (phase I), sociability test (phase II) and social novelty test (phase III). In phase I, subject mice explored the center chamber of the apparatus for 5 min with blocked entrance and visibility of the side chambers. During the sociability test (phase II), the subject mice explored all three chambers for 5 min, with one side of the chamber holding an inverted empty holding cup (represented by the letter “E” in the calculations below) and the other side of the chamber holding the gender matched stranger (C57BL/6J) mouse in the inverted holding cup. The positioning of the stranger mice during the sociability test was alternated between the left and right side of chamber to control for bias by the subject mice between the sides of the chamber.

After the sociability test, the subject mice were allowed to briefly return to the center chamber with sides of the chamber secured. The previously employed stranger mouse, referred to as “stranger 1 (S1)” in the sociability test remained in place during the brief interval gap. In the opposite side of the chamber that housed the S1 mouse, another stranger mouse, referred to as “stranger 2 (S2),” was placed in an inverted wire cup. For the social novelty test (phase III), the subject mice explored the 3-chamber apparatus for 5 min, containing S1 in one side and S2 in other side of the chamber. The amount of time the subject mice interacted with either S1 or S2 was analyzed using the ANY-maze behavioral analysis software (Wood Dale, IL, US). The percentage of time spent with the stranger 1 (S1) mice during sociability test and stranger 2 (S2) mice during social novelty test was calculated as [(S1/S1 + E) × 100] and [(S2/S1 + S2) × 100], respectively.

#### 2.5.3. Statistical methods

For Figures 2E–G and Supplementary Figures 11–15: Data analyses were performed using JMP Pro 16.2.0 (SAS Institute Inc., Cary, NC, USA). Cell densities were evaluated with two-way analysis of variance (ANOVA) with mouse type, treatment, and their interaction as the fixed effects. Logarithmic transformation was performed for data that did not meet the normality criteria. Assumptions of the model, normality of residuals and homoscedasticity, were accessed by examining standardized residual and quantile plots. The normality of the residuals was also confirmed via Shapiro-Wilk test. When a fixed interaction was detected, *post-hoc* Fisher LSD comparisons were performed. Significance was set at *p* < 0.05. Data were expressed as mean ± SEM and significance were presented as *, 0.05 > *p* > 0.01; **, 0.01 > *p* > 0.001; ***, 0.001 > *p* > 0.0001; ****, *p* < 0.0001. Details of the statistical analysis are included as supplementary data files. Statistical results for supplementary figures are also provided in the supplementary figure legends (Supplementary Figures 11–15).

For Figures 3–5 and Supplementary Figures 16–37: Data analyses were performed using JMP Pro 16.2.0 (SAS Institute Inc., Cary, NC, USA). Variable measured before and after the treatment were evaluated with a mixed analysis of variance (ANOVA) model with treatment (P or ChABC), time (before or after), and mouse type (C57 or CNTNAP) and all their interactions as the fixed effects and each mouse was entered as the random effect. Assumptions of the model, normality of residuals and homoscedasticity, were accessed by examining standardized residual and quantile plots. The normality of the residuals was also confirmed via Shapiro–Wilk test. When a fixed interaction was detected, *post-hoc* Fisher LSD comparisons were performed. Significance was set at *p* < 0.05. Data were expressed as mean ± SEM and significance were presented as *, 0.05 > *p* > 0.01; **, 0.01 > *p* > 0.001; ***, 0.001 > *p* > 0.0001; ****, *p* < 0.0001. Details of the statistical analysis are included as supplementary data files. Statistical results for supplementary figures are also provided in the supplementary figure legends (Supplementary Figure 16–37).

## 3. Results

### 3.1. Expression of PNNs and pV-positive interneurons in the prefrontal cortex (PFC) of CNTNAP2^–/–^ mice

**FIGURE 1:**
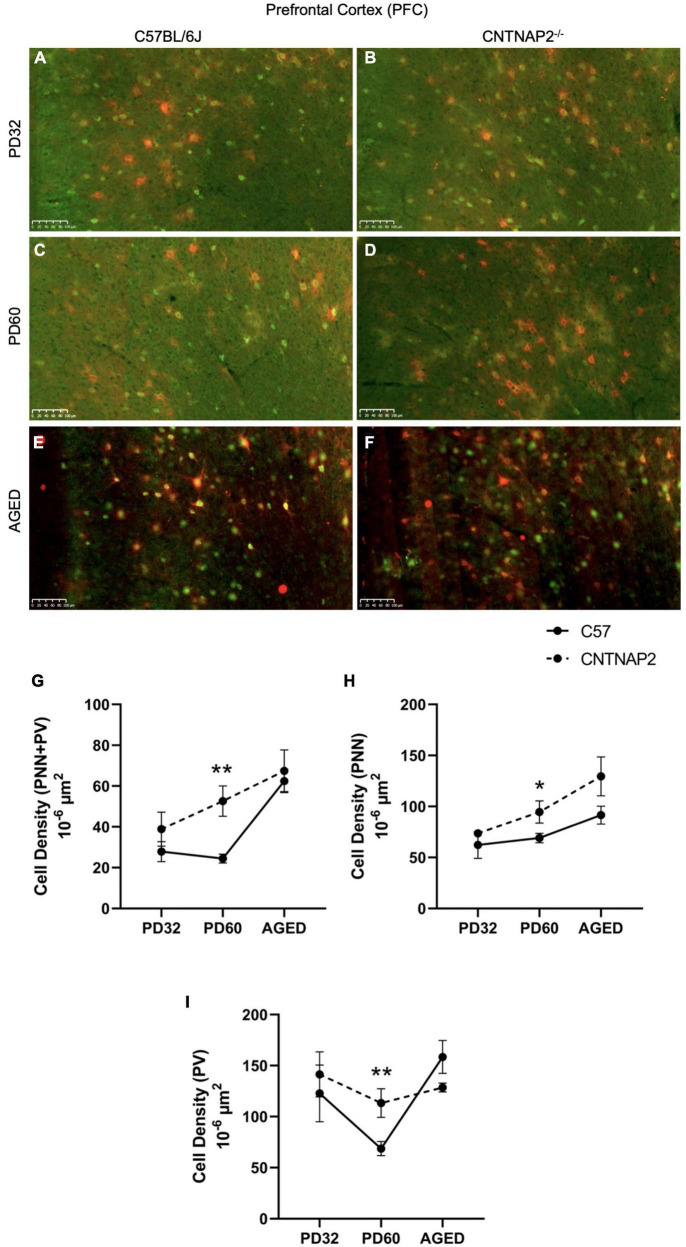
Expression of perineuronal nets (PNNs) and parvalbumin-positive interneurons (PVs) in the prefrontal cortex (PFC) of C57BL/6J and CNTNAP2^–/–^ mice. **(A–E)** Immunofluorescence images depicting co-localized PNNs and PV-positive cells in prefrontal cortex (PFC) of C57BL/6J (left images) and CNTNAP2^–/–^ (right images) mice at PD 32 **(A,B)**, PD 60 **(C,D)**, and PD 395-425 (aged) **(E,F)**. Scale bar 100 μm. **(G–I)** Quantitative estimation of PNNs co-localized with PV-positive neurons **(G)**, PNNs **(H)**, and PV-positive cells **(I)** in prefrontal cortex (PFC) of C57BL/6J (*n* = 3–7) and CNTNAP2^–/–^ (*n* = 3–6) mice at different postnatal ages. Significance levels (*, **) are as noted in the Methods.

Perineuronal nets, PVs, and their co-localization in the PFC region were analyzed in brain sections of control C57BL/6J (WT) and CNTNAP2 mutant mice at different postnatal ages, i.e., PD 32 (young), PD 60 (adult), and P395-P425 (aged) (Figure 1 and Supplementary Figures 1–8). The quantitative distribution of PNNs and PVs were determined from average counts in a defined PFC ROI to obtain cell densities (see section “Materials and methods”). At PD 32, no difference was observed in PNNs, PVs and their co-localization densities in PFC region between CNTNAP2^–/–^ and control groups (Figures 1A, B, G–I and Supplementary Figure 2). But at PD 60, CNTNAP2^–/–^ mice exhibited significant increases in PNNs, PVs, and co-localized cell densities in PFC in comparison with WT mice (Figures 1C, D G-I and Supplementary Figures 3, 5, 6). The CNTNAP2^–/–^ mice exhibited 37% increased densities of PNNs and 65% increased densities of PVs compared to C57 mice at PD 60 (Figures 1H, I). In aged CNTNAP2^–/–^ mice, there is a decline in PV-positive neuron number per unit area (∼19%) in PFC compared to C57 mice (Figures 1G–I and Supplementary Figures 7, 8). The observed differences were mostly independent of gender at PD 60, but in aged animals, increased numbers of PNNs and PNNs surrounding PVs were observed, particularly in aged female CNTNAP2^–/–^ mice compared to wild-type controls (Supplementary Figures 5–8).

### 3.2. Enzymatic degradation of PNNs in PFC via chondroitinase

**FIGURE 2:**
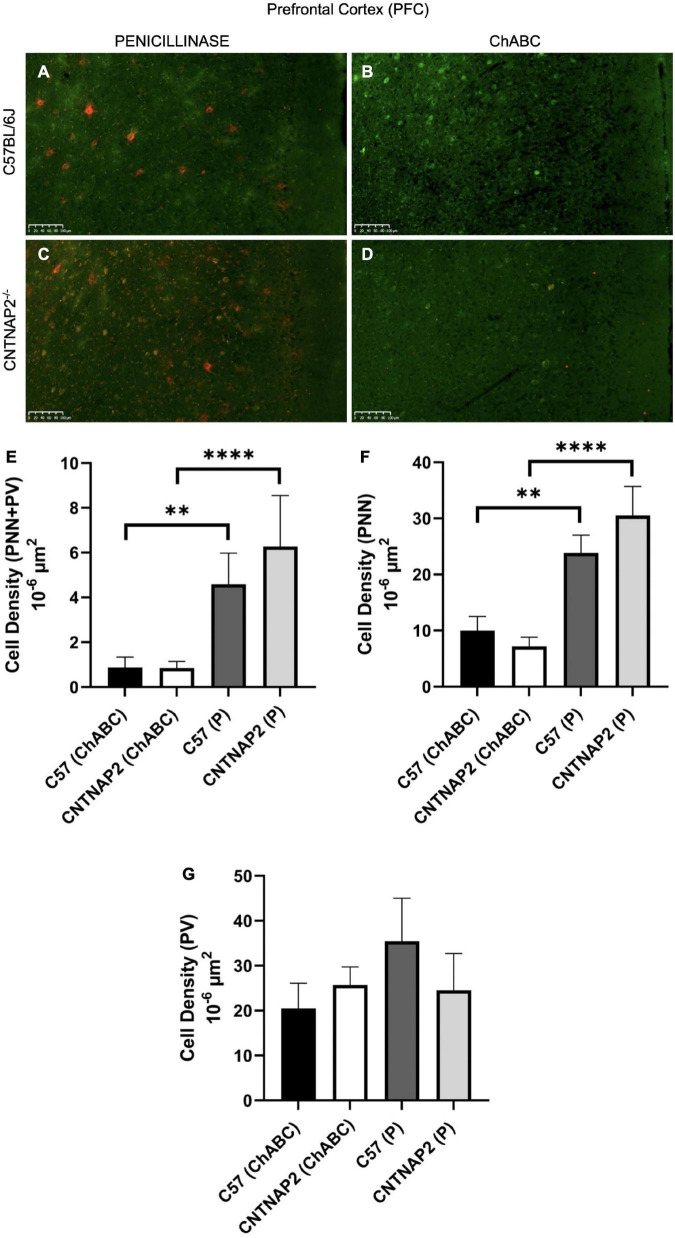
Validation of perineuronal net digestion by chondroitinase ABC (ChABC) injections in the prefrontal cortex (PFC) of adult mice (*PD 60*) after behavioral tests. **(A–D)** Immunofluorescence images depicting distribution of co-localized PNNs and PV-positive cells after penicillinase (left images) and ChABC (right images) injections in the PFC of adult C57BL/6J **(A,B)** and CNTNAP2^–/–^ **(C,D)** mice. Scale bar 100 μm. **(E–G)** Quantitative estimation of PNNs co-localized with PV-positive neurons **(E)**, PNNs **(F)**, and PV-positive cells **(G)** in prefrontal cortex (PFC) of adult C57BL/6J and CNTNAP2^–/–^ mice treated with chondroitinase (C57BL/6J, *n* = 11; CNTNAP2^–/–^, *n* = 13) and penicillinase (C57BL/6J, *n* = 10; CNTNAP2^–/–^, *n* = 10). Data expressed as mean ± SEM (*p* < 0.05). Two-way ANOVA and Fisher LSD *post-hoc* comparisons were performed with significant treatment effects (E: *F*_1,40_ = 30.89, *p* < 0.0001; F: *F*_1,40_ = 34.12, *p* < 0.0001; G: *F*_1,40_ = 0.63, p = 0.4308) on panels **(E,F)**. Significance levels (**, ****) are as noted in the Methods.

To understand the role of the increased expression of PNNs and their co-localization with PV-positive neurons underlying autistic-like phenotypes in the CNTNAP2^–/–^ mutant mice, we employed chondroitinase ABC injections to enzymatically digest PNNs in the prefrontal cortical region. We injected both mutant and WT adult (∼PD 60) mice with chondroitinase and penicillinase (serving as an inert control) to assess neuroanatomical and behavioral parameters in CNTNAP2 mutant mice with respect to control C57 mice. We employed bacterial derived penicillinase enzyme injections in the PFC region in a different group of animals to serve as a control for animals injected with ChABC. Pencillinase lacks enzymatic activity in the PFC but matches the biophysical properties of chondroitinase to serve as an inert protein for control injections (Massey et al., 2006; Lensjø et al., 2017) (Figure 2 and Supplementary Figures 9–12). Injections were performed in bilaterally matched PFC regions.

To validate the effects of these injections, we examined expression of PNNs and PVs following behavioral assessments, described below. As expected, we found that chondroitinase injections reduced the density of expression by 2- to 3-fold of well-formed PNNs in the PFC, in comparison with penicillinase controls (Figure 2). Both PNNs and PNNs surrounding PVs were substantially reduced by over 60%, but PV expression was largely unaffected (Figure 2). Two-way ANOVA and Fisher LSD *post hoc* comparisons were performed with significant treatment effects (Figure 2E: F_1,40_ = 30.89, *p* < 0.0001; Figure 2F:F_1,40_ = 34.12, *p* < 0.0001; Figure 2G: F_1,40_ = 0.63, p = 0.4308) on (E) and (F). The effects of chondroitinase injections were similar by gender (Supplementary Figures 11, 12). The change in the number of PNNs after digestion likely reflects a disruption of PNN extracellular matrix structures that influence PV-positive neuronal dynamics in the PFC region and may account for some of the observed behavioral alterations discussed in the following section. Overall, we found that chondroitinase injections effectively digested PNN structures in the PFC.

#### 3.2.1. Repetitive self-grooming

Behavioral tests applicable to ASD behavioral deficits and PFC-related behaviors were conducted in both the knockout and wild-type animals before and after injection of chondroitinase and penicillinase. Repetitive behaviors including motor stereotypies are often associated with the ASD behavioral repertoire (Goldman et al., 2009; Singer, 2009; Péter et al., 2017). Stereotypic behavior including repetitive grooming and rearing have been described in CNTNAP2^–/–^ mice at different postnatal ages (Penagarikano et al., 2011; Scott et al., 2019; Wang et al., 2020) and were assessed here.

**FIGURE 3:**
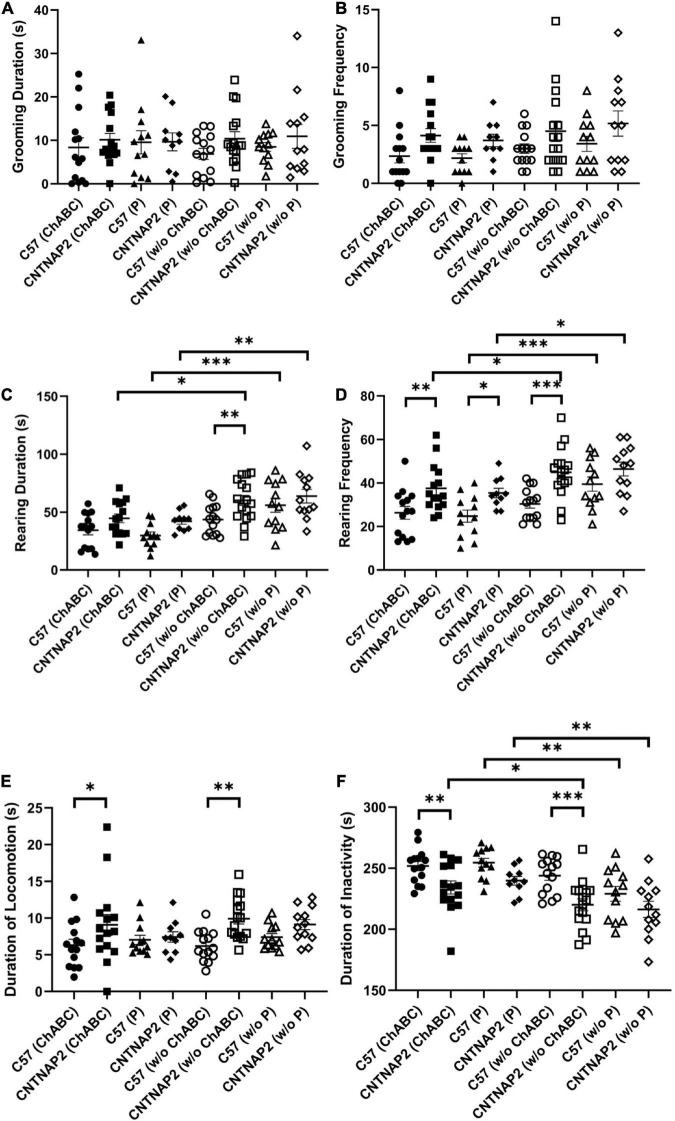
Analysis of repetitive grooming and rearing behaviors following ChABC and penicillinase injections. **(A–D)** Behavioral analyses of grooming duration and frequency **(A,B)**, rearing duration and frequency **(C,D)**, and duration of locomotion and inactivity **(E,F)** in adult C57BL/6J and CNTNAP2^–/–^ mice treated with chondroitinase (C57BL/6J, *n* = 14; CNTNAP2^–/–^, *n* = 15) and penicillinase (C57BL/6J, *n* = 12; CNTNAP2^–/–^, *n* = 10). Data expressed as mean ± SEM (*p* < 0.05) (*p* < 0.1). Mixed ANOVA and Fisher LSD *post-hoc* comparisons were performed with 3-way interactions (A: *F*_1,49.3_ = 0.02, *p* = 0.8868; B: *F*_1,48.9_ = 0.08, *p* = 0.7722; C: *F*_1,50.2_ = 0.67, *p* = 0.4171; D: *F*_1,50.3_ = 0.81, *p* = 0.3728, E: *F*_1,50.7_ = 0.02, *p* = 0.8750, F: *F*_1,50.2_ = 0.34, *p* = 0.5605). Significance levels (*, **, ****) are as noted in the Methods.

For repetitive grooming behaviors, we found that the CNTNAP2^–/–^ group exhibited a general trend toward increased grooming frequency and duration in comparison to matched wild-type groups, however these differences were variably significant across gender and injections (Figures 3A, B and Supplementary Figure 13). For instance, the ChABC and penicillinase injected female CNTNAP2^–/–^ mice exhibited increased duration and frequency of grooming than matched female C57 mice, but these were not significantly observed among male groups (Supplementary Figures 13B, D). Importantly, injection of ChABC did not significantly affect these repetitive grooming behaviors, suggesting that PNNs in the PFC do not contribute to these behaviors (Figures 3A, B and Supplementary Figures 13, 14). Mixed ANOVA and Fisher LSD *post hoc* comparisons were performed with 3-way interactions (Figure 3A: *F*_1,49.3_ = 0.02, *p* = 0.8868; Figure 3B: *F*_1,48.9_ = 0.08, *p* = 0.7722).

In terms of rearing behaviors, CNTNAP2^–/–^ mice exhibited significantly increased duration and frequency of vertical rears compared with matched wild-type controls (Figures 3C, D and Supplementary Figure 14). As with the grooming behaviors above, many of these differences were most significant among female mice compared with condition-matched males (Supplementary Figure 14). Injections of either ChABC or penicillinase resulted in reduced duration and frequency of vertical rears in both the WT and CNTNAP2^–/–^ mutant group compared to before injections (Figures 3C, D and Supplementary Figure 14). However, this overall reduction in rearing behaviors following injection of either ChABC or penicillinase is likely explained by the corresponding decrease in overall activity observed among injected groups after surgeries (Figure 3F and Supplementary Figure 15). Mixed ANOVA and Fisher LSD *post hoc* comparisons were performed with 3-way interactions (Figure 3C: *F*_1,50.2_ = 0.67, *p* = 0.4171; Figure 3D: *F*_1,50.3_ = 0.81, *p* = 0.3728, Figure 3E: *F*_1,50.7_ = 0.02, *p* = 0.8750, Figure 3F: *F*_1,50.2_ = 0.34, *p* = 0.5605). Overall, these data suggest that repetitive grooming and rearing behaviors are not altered by ChABC treatment in the PFC.

#### 3.2.2. Social interaction tests

As a measure of social interaction deficits in ASD, we performed a three-chamber social interaction test comprised of both sociability and social novelty phases in WT and mutant mice (Figures 4, 5 and Supplementary Figures 16–37). Prior to ChABC treatment, the CNTNAP2^–/–^ group displayed sociability deficits, as manifested by less time spent with the stranger mouse in comparison to WT mice, but oddly not observed in the penicillinase groups (Figures 4A, B, E, F, I, J). Similarly, the CNTNAP2^–/–^ group showed decreased time spent in the chamber housing the stranger mouse compared to the WT group before ChABC injection (Figures 4F–I), but no significant differences in entries to the stranger mouse chamber were observed (Supplementary Figure 18). Mixed ANOVA and Fisher LSD *post hoc* comparisons were performed with 3-way interactions (Figure 4I: *F*_1,50.2_ = 5.02, *p* = 0.0295; Figure 4J: *F*_1,50.4_ = 4.65, *p* = 0.0359).

**FIGURE 4:**
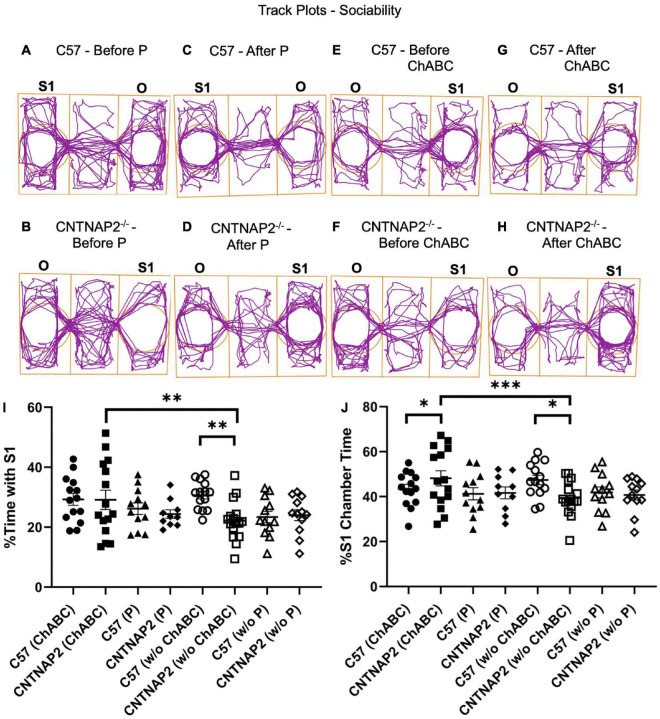
Assessment of sociability in the three-chamber sociability test. **(A–H)** Track plot examples display the position of the animal’s center point for the total test duration before penicillinase injection **(A,B)**, after penicillinase injection **(C,D)**, before chondroitinase ABC injection **(E,F)**, and after chondroitinase ABC injection **(G,H)** in adult C57BL/6J and CNTNAP2^–/–^ mice. **(I,J)** Quantification of three-chamber sociability performance according to percentage time spent with stranger mice **(I)** and percentage time spent in chamber housing stranger mice **(J)** in adult C57BL/6J and CNTNAP2^–/–^ mice. C57BL/6J mice treated with chondroitinase (*n* = 8 males; *n* = 6 females) and penicillinase (*n* = 6 males; *n* = 6 females). CNTNAP2^–/–^ mice treated with chondroitinase (*n* = 8 males; *n* = 7 females) and penicillinase (*n* = 4 males; *n* = 6 females). Data expressed as mean ± SEM. Mixed ANOVA and Fisher LSD *post-hoc* comparisons were performed with 3-way interactions (I: *F*_1,50.2_ = 5.02, *p* = 0.0295; J: *F*_1,50.4_ = 4.65, *p* = 0.0359). Significance levels (*, **, ****) are as noted in the Methods.

**FIGURE 5:**
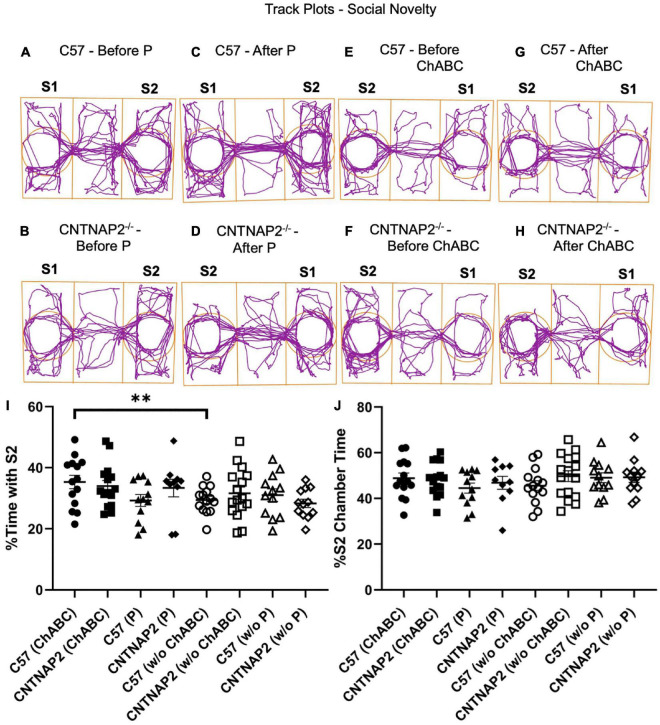
Assessment of social novelty behaviors in the three-chamber social interaction task. **(A–H)** Example track plots display the position of the animal’s center point for the total test duration before penicillinase injection **(A,B)**, after penicillinase injection **(C,D)**, before chondroitinase ABC injection **(E,F)**, and after chondroitinase ABC **(G,H)** injections in C57BL/6J and CNTNAP2^–/–^ mice. **(I,J)** Quantification of three-chamber social novelty according to percentage time spent with stranger mice performance **(I)** and percentage time spent in chamber housing stranger mice **(J)** for C57BL/6J and CNTNAP2^–/–^ mice. C57BL/6J mice treated with chondroitinase (*n* = 8 males; *n* = 6 females) and penicillinase (*n* = 6 males; *n* = 6 females). CNTNAP2^–/–^ mice treated with chondroitinase (*n* = 8 males; *n* = 7 females) and penicillinase (*n* = 4 males; *n* = 6 females). Data expressed as mean ± SEM. Mixed ANOVA and Fisher LSD *post-hoc* comparisons were performed with 3-way interactions (I: *F*_1,47.9_ = 5.07, *p* = 0.0289; J: *F*_1,48.7_ = 1.38, *p* = 0.2451). Significance level (**) is as noted in the Methods.

In terms of the empty holding cup chamber, CNTNAP2^–/–^ mice prior to ChABC injections spent more time in the empty holding cup chamber than wild-type controls and was mainly observed among male mice (Supplementary Figure 20). Similarly, in terms of the center chamber, the CNTNAP2^–/–^ male, but not female, mice spent more time in the center than wild-type controls (Supplementary Figure 22). Conversely, female, but not male, CNTNAP2^–/–^ mice exhibited increased locomotor activity compared to wild-type controls, as measured by total distance traveled and mean speed, indicating hyperactive behavior that is gender specific (Supplementary Figures 25, 26, 36, 37). Mixed ANOVA and Fisher LSD *post-hoc* comparisons were performed with 3-way interactions (e.g., Supplementary Figure 25A: *F*_1,47.6_ = 5.53, *p* = 0.0228; e.g., Supplementary Figure 25B: *F*_1,48.6_ = 5.44, *p* = 0.0239). Overall, the CNTNAP2 mutant group exhibited general sociability deficits as indicated by a preference for the empty holding cup and center chambers over the stranger chamber, particularly in male mice (Figure 4 and Supplementary Figures 17–26).

Interestingly, after ChABC treatment, the CNTNAP2^–/–^ group exhibited enhanced sociability behavior, as indicated by increased time spent in the stranger mouse chamber with respect to the C57 group (Figures 4F–J and Supplementary Figures 16, 17). By gender, mutant male mice, but not females, particularly showed pre-injection deficits and post-injection increases in percent time spent in the chamber housing the stranger mouse after ChABC treatment compared to the CNTNAP2^–/–^ group before ChABC treatment (Figure 4 and Supplementary Figures 16, 17). Mixed ANOVA and Fisher LSD *post hoc* comparisons were performed with 3-way interactions (Figure 4I: *F*_1,50.2_ = 5.02, *p* = 0.0295; Figure 4J: *F*_1,50.4_ = 4.65, *p* = 0.0359). The change in sociability after ChABC treatment in the mutant group is not related to increased locomotor activity displayed by the mutant group, since the penicillinase treated CNTNAP2^–/–^ group did not exhibit changes in sociability measures, even after showing greater distance traveled and mean speed than C57 penicillinase treated group (Supplementary Figures 16–21, 25-26). Overall, these results suggest that ChABC treatment ameliorates sociability deficits in the CNTNAP2 mutant group, particularly the male CNTNAP2^–/–^ group.

However, for the social novelty test, no differences between the genotypes were observed in either the time spent or entries in the chambers housing the familiar mouse (S1) and second stranger mouse (S2), although some behavioral differences were observed among some groups (Figure 5 and Supplementary Figures 27–5). For instance, the CNTNAP2^–/–^ group treated with penicillinase spent less time in the center chamber in contrast to WT group (Supplementary Figure 33), and entries in the center chamber and total entries in the familiar and stranger mouse chambers were different in the CNTNAP2^–/–^ group before and after ChABC application (Supplementary Figures 34, 35). As noted above, the CNTNAP2^–/–^ group traveled a greater distance and mean speed compared to WT group, confirming the hyperactivity induced locomotor behavior displayed by this mutant strain, particularly in the females (Supplementary Figures 37, 38). Mixed ANOVA and Fisher LSD *post hoc* comparisons were performed with 3-way interactions (Figure 5I: *F*_1,47.9_ = 5.07, *p* = 0.0289; Figure 5J: *F*_1,48.7_ = 1.38, *p* = 0.2451). Overall, though, no differences in social novelty behaviors were observed following ChABC injections.

## 4. Discussion

In this study, we found alterations in WFA-labeled PNNs, PVs, and PNNs enwrapping PV-positive cells in the PFC of CNTNAP2^–/–^ mice, particularly in adult animals. In line with prior findings in CNTNAP2 knockout mice at PD25 (Lauber et al., 2018a), we did not observe any significant changes in the numbers of PNNs, parvalubmin-positive neurons and double-labeled cells in the PFC region of the mutant mice in young animals at PD32. However, we observed significant increases in the densities of PNNs, PV-positive neurons and double-labeled cells in CNTNAP2 knockout mice in adult animals, at PD 60. Further, in aged female mutant mice, the PNNs and PNNs co-localized with PV neurons showed significantly increased densities compared to control mice, indicating alterations in PNNs and parvalubmin-positive neuron numbers and dynamics with age. These alterations suggest accelerated growth and maturation of PNN components and enwrapped PV-positive cell types that may be involved in disrupting E/I balance and contributing to the abnormal behavioral phenotype in CNTNAP2 mutant mice.

In addition, we found that transient removal of PNNs in adult animals affected social interactions, but not repetitive behaviors, in adult male CNTNAP2 mutant mice. Utilizing digestion of PNNs in the PFC may restore juvenile-like plastic states in adult mice to reveal improvements in social behavior of CNTNAP2 knockout mouse model of autism (Table 1). This behavioral change via PNN degradation might be due to changes in the activity of PNNs and PNN enwrapped parvalubmin-positive neurons resulting in restoration of E/I balance and regulation of PV-expressing cell networks in a region- and circuit-specific manner. For instance, optogenetically increasing parvalubmin-positive neuronal excitability in the mPFC rescues social deficits in CNTNAP2 mutant mice and is linked to increased inhibitory tone and decreased activity of cortico-striatal circuits (Turrigiano, 2011; Selimbeyoglu et al., 2017). This indicates that removal of PNNs in the PFC of the CNTNAP2 KO mice may similarly modulate inhibition in the brain to rescue social behavioral deficits in these mutant mice.

**TABLE 1:** Summary of behavioral tests and effects after chondroitinase treatment.

Behavior	C57BL/6J	CNTNAP2^–/–^
Repetitive self-grooming	-	−
Sociability	-	+
Social novelty	-	−

Gender specific variations were observed in histology and some behaviors, however, it was not robustly or generally skewed toward one gender. The variations observed in gender could be due to random sampling of male and female mice groups selected for the study. Based on previous clinical and preclinical studies in ASD, it is unclear whether the observed gender-specific differences result from general variation between genders or are particular to the autistic conditions (Hull et al., 2017, 2020; Wood-Downie et al., 2021). Previous studies present conflicting views on observed gender differences in autism. Some studies suggest that the diagnostic tools for evaluating the autistic condition do not consider aspects of male and female differences, accounting for underestimation of ASD prevalence in female population (Beggiato et al., 2017; Hull et al., 2017; Antezana et al., 2019). However, other studies suggest that female protective effects result in the prevalence of autistic conditions diagnosed in males (Sedgewick et al., 2019; Zhang et al., 2020).

Parvalbumin-positive cells regulate excitatory principal neuronal output, development of neural networks, information processing, and cognitive flexibility (Sohal et al., 2009; Whittington et al., 2011; Cho et al., 2015). GABAergic PV-expressing interneuron dysfunction is observed in several neurological disorders including autism, bipolar disorder, and schizophrenia (Bailey et al., 1998; Blatt et al., 2001; Fatemi et al., 2002; Blatt, 2005; Torrey et al., 2005; Pantazopoulos et al., 2007; Andreazza et al., 2008; Lewis et al., 2011; Uhlhaas and Singer, 2011; Kulak et al., 2013; Steullet et al., 2018). Post-mortem samples from ASD individuals reveal considerable decreases in parvalbumin mRNA levels in frontal and temporal cortices (Parikshak et al., 2016). Furthermore, PV-expressing interneuronal signaling and reduced numbers of PV neurons are observed in human ASD patients and ASD mouse models (Penagarikano et al., 2011; Wöhr et al., 2015; Filice et al., 2016; Lauber et al., 2016, 2018a; Hashemi et al., 2017).

Alterations to PV populations result in several neurophysiological effects. Absence of the parvalbumin calcium binding protein in PV-expressing interneurons enhances short-term facilitation that leads to a rise in PV neuronal frequency-contingent output and shaping GABA release at presynaptic terminals (Caillard et al., 2000; Collin et al., 2005; Müller et al., 2007; Schwaller, 2012). Conversely, increased expression of PV results in asynchronous inhibitory responses that affect the timing of neuronal firing, thereby leading to desynchronization of neural networks and disruption of cortical information processing (Manseau et al., 2010). This indicates that modulation of PV neuronal activity during the critical window of neurodevelopment might alleviate some core autistic symptoms (Lauber et al., 2018b).

The critical period window is linked with the maturation of PV-positive inhibitory cells that suppresses excitatory neuronal spontaneous activity after sensory stimulation (Fagiolini and Hensch, 2000; Fagiolini et al., 2004; Hensch, 2005; Toyoizumi et al., 2013). At the end of the critical period, PV-positive cell maturation coincides with formation of specialized extracellular matrix structures around them, such as PNNs (Hockfield et al., 1990; Pizzorusso et al., 2002; Balmer et al., 2009; Carulli et al., 2010). Interneuron maturation is promoted by the conducive extracellular environment provided by PNNs, which results in stabilization and maintenance of inhibitory synaptic and network activity, regulation of excitatory and inhibitory balance, remodeling of circuits, plasticity, and learning (Sugiyama et al., 2009; Kwok et al., 2011; Beurdeley et al., 2012; Cabungcal et al., 2013b; Donato et al., 2013; Liu et al., 2013; Morawski et al., 2015).

Perineuronal nets consists of chondroitin sulfate proteoglycans (CSPGs) such as aggrecan, neurocan, brevican, hyaluronan, tenascin, and link proteins (Carulli et al., 2010). Changes in CSPG-containing PNNs restrict plasticity, and the enzymatic digestion of chondroitin sulfate (CS) chains of PNNs by chondroitinase ABC results in reopening of critical timepoint and reactivating plasticity in the adult central nervous system (CNS). Removal of PNNs leads to regeneration of axons in the adult CNS which has important implications in neurodegenerative diseases, addiction and recovery from CNS related injury (Massey et al., 2006; Galtrey and Fawcett, 2007; Kwok et al., 2008; Alilain et al., 2011; Blanchard et al., 2012; Mercier et al., 2012; Xue et al., 2014; Yang et al., 2015; Kasinathan et al., 2016). CS modulates concentrations of intracellular Ca^2+^ by activating non-voltage-gated Ca^2+^ channels (Snow et al., 1994). Digestion of PNNs reduces excitability of inhibitory neurons and restores plasticity (Sugiyama et al., 2009; Balmer, 2016; Lensjø et al., 2017; Carulli and Verhaagen, 2021). Altered inhibitory activity, resulting in abnormal neural synchrony, particularly in the PFC and sensory cortical regions, is implicated in several neurological disorders including schizophrenia, autism, and bipolar disorder (Cho et al., 2006; Spencer et al., 2008; Barr et al., 2010; Uhlhaas and Singer, 2011; Gandal et al., 2012; Wen et al., 2018).

The PFC plays a critical role in executive function, learning, recognition memory, social and aggressive behavior. Postmortem studies in patients with schizophrenia report changes in parvalubmin-positive neuronal function and depletion of PNN structures particularly in the PFC region (Zhang and Reynolds, 2002; Do et al., 2009; Pantazopoulos et al., 2010, 2015; Lewis et al., 2011; Berretta, 2012; Mauney et al., 2013; Cabungcal et al., 2014; Enwright et al., 2016). In a mouse model of schizophrenia, drug induced alterations in dopaminergic signaling in the PFC increased network activity that was amplified by ChABC digestion of PNNs (Steullet et al., 2014). Degradation of PNNs by chondroitinase application decreased long-term potentiation (LTP) in hippocampal CA1 region, suggesting a crucial role for chondroitin sulfate (CS) in long-term plasticity (Bukalo et al., 2001). Further, CSPGs regulate synaptic plasticity underlying learning and memory (Galtrey and Fawcett, 2007; Nakamura et al., 2009; Hou et al., 2017). Hence, PNNs and PV-positive interneurons are involved in the onset and closure of the critical period and plasticity (Hensch, 2005; Lensjø et al., 2017; Reichelt et al., 2019; Carulli and Verhaagen, 2021).

Degrading PNNs in the PFC has several behavioral consequences. It impairs drug-related memories and is an impressive therapeutic target for drug associated addiction and relapse (Slaker et al., 2018). Additionally, PNN removal by ChABC in the mPFC reduces inhibitory current frequency onto pyramidal cells, leading to impairment of cocaine influenced memory in a conditioned place preference paradigm (Slaker et al., 2015). As PNNs have a crucial role in maturation of PV neurons, de-maturation of these neurons might be involved in the therapeutic mechanism of the antidepressant drug, fluoxetine. Chronic treatment with fluoxetine leads to reduction in expression of PV and PNN in the hippocampus (Ohira et al., 2013; Guirado et al., 2014).

While ChABC application is useful in understanding PNN function and their effect on physiological activity, it has more broad-ranging effects in the brain. Future studies targeting specific removal of PNN components are required to understand individual component effect on neural plasticity in distinct brain regions. For instance, Brevican, a critical component of PNNs and highly abundant CSPGs in the brain, regulates cellular and synaptic plasticity in PV-expressing neurons via modulating AMPA receptors and clustering of potassium channels (Favuzzi et al., 2017). Interestingly, genetic depletion of PNN components cartilage-link-1 protein and Tenascin-R, improves object recognition in perirhinal cortex and reversal learning and working memory models (Morellini et al., 2010; Romberg et al., 2013). Understanding the underlying mechanisms of PNN development and modulation in normal and diseased brain states may help reveal novel therapeutic targets for relevant brain disorders.

Further refining the role of PNNs in regulating neuronal E/I balance, cortical and physiological function is necessary for ultimately understanding and treating neurodevelopmental disorders such as ASD. Our findings demonstrate altered expression of PNNs and GABAergic PV-expressing interneurons in the PFC region at various developmental timepoints in a mouse model of autism and that these mediate aspects of social behaviors in the mutant mice. These results support prior findings that implicate PNNs in the PFC as an important neurobiological substrate for some ASD associated behaviors.

## Data availability statement

The original contributions presented in this study are included in the article/Supplementary material, further inquiries can be directed to the corresponding author.

## Ethics statement

The animal study was reviewed and approved by Institutional Animal Care and Use Committee of the Louisiana State University.

## Author contributions

TG and CCL conceived, designed the study, and wrote the manuscript. TG and TA conducted the experiments. All authors analyzed the data.
