# Supplementary material for: Behavioral regulation by perineuronal nets in the prefrontal cortex of the CNTNAP2 mouse model of autism spectrum disorder
Source: Front Behav Neurosci. 2023 Mar 14;17:1114789. doi: 10.3389/fnbeh.2023.1114789 (PMC10043266; doi:10.3389/fnbeh.2023.1114789)
Supplement: Supplementary file 1 [file Data_Sheet_1.pdf]

A

B

C

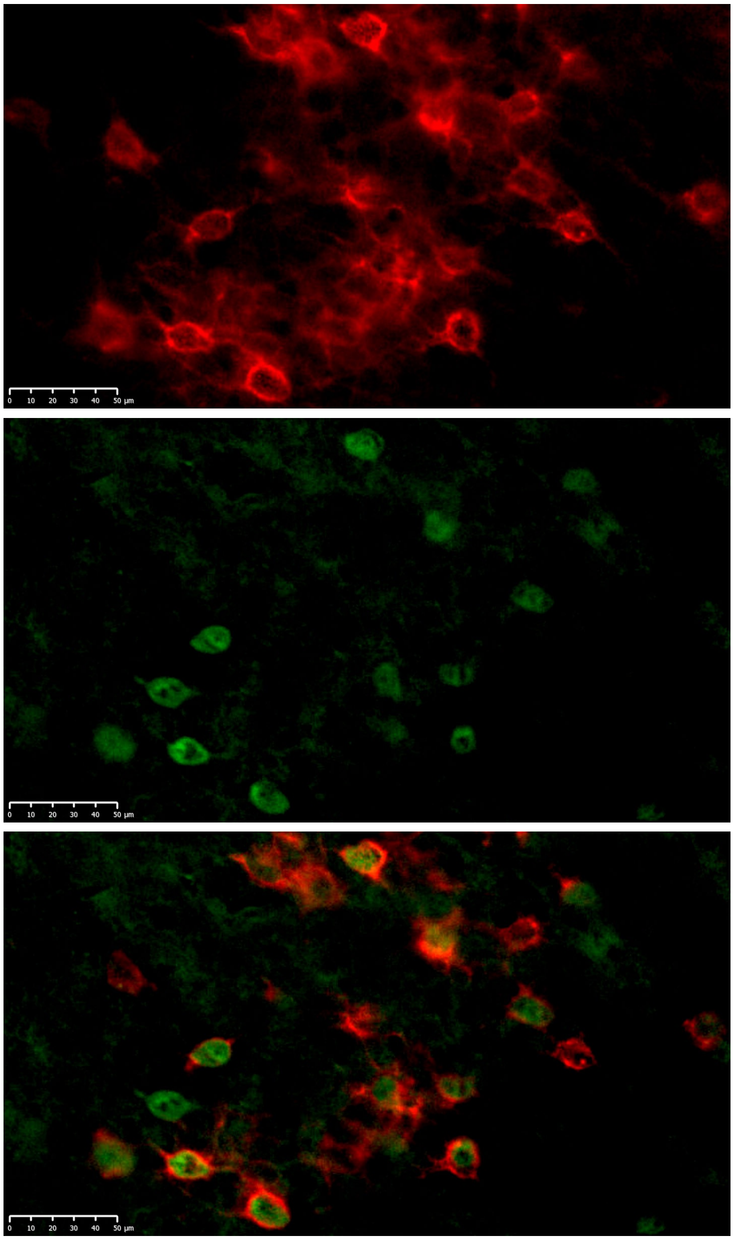

Figure 1. Images showing (A) WFA-labeled PNNs (red), (B) PV-positive neurons (green) and (C) co-localized PNNs and PV+ cells. Scale bar 50 μm.

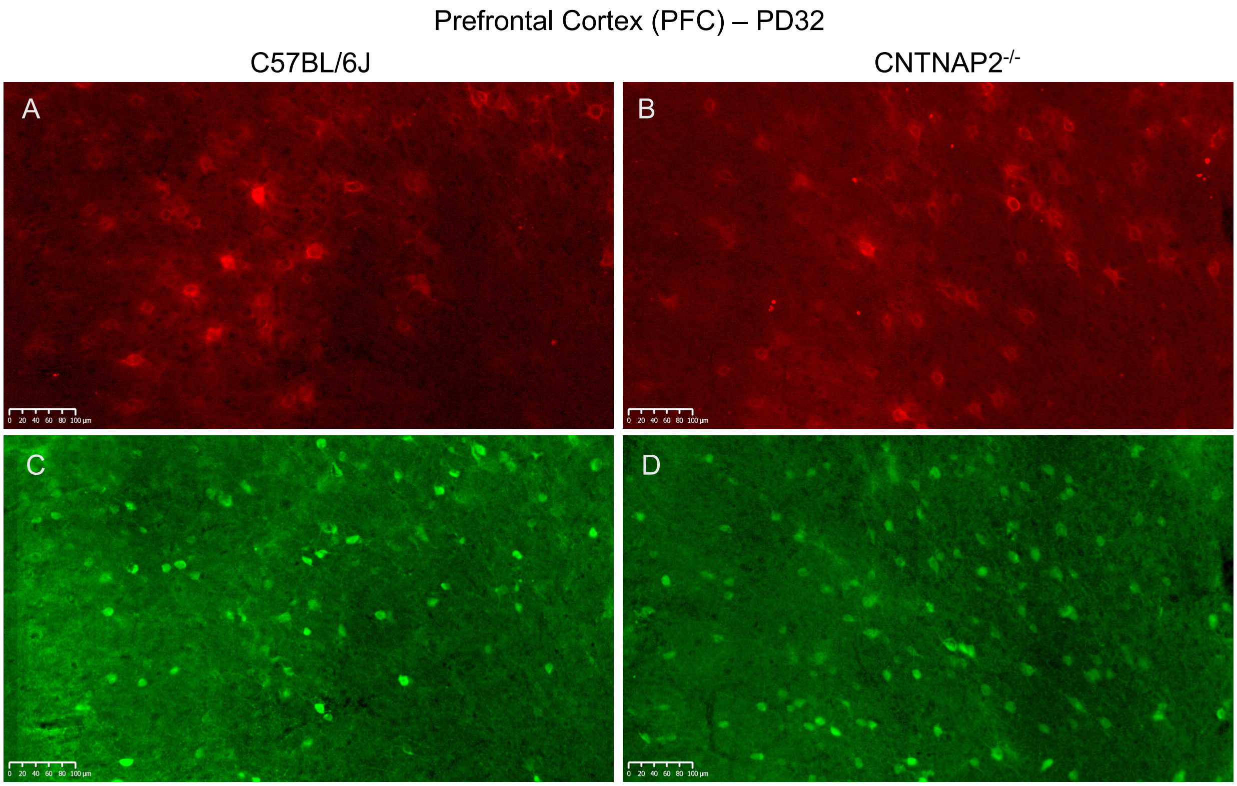

Figure 2. Immunofluorescence images depicting (A, B) PNNs (red) and, (C, D) PV-positive neurons (green) in prefrontal cortex (PFC) of C57BL/6J and CNTNAP2$^{-/-}$ mice at PD 32. Scale bar 100 μm.

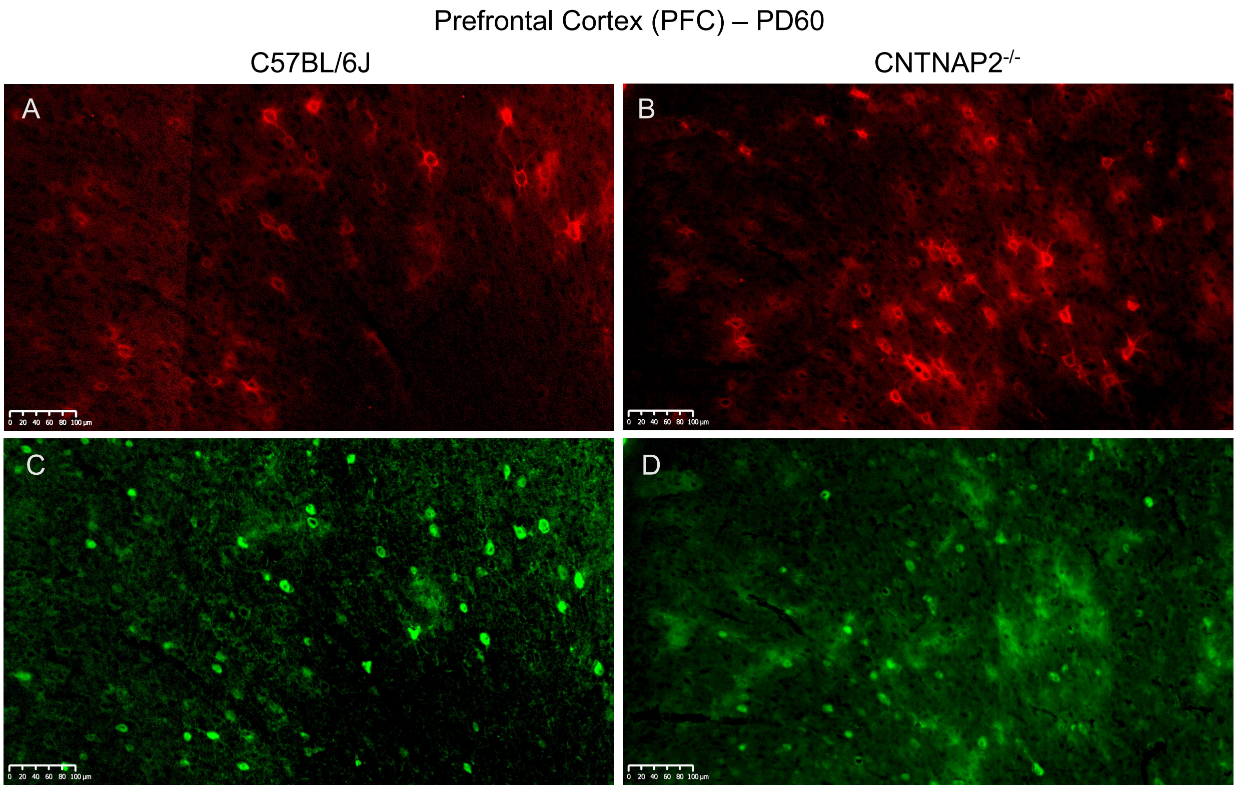

Figure 3. Immunofluorescence images depicting (A, B) PNNs (red) and, (C, D) PV-positive neurons (green) in prefrontal cortex (PFC) of C57BL/6J and CNTNAP2$^{-/-}$ mice at PD 60. Scale bar 100 µm.

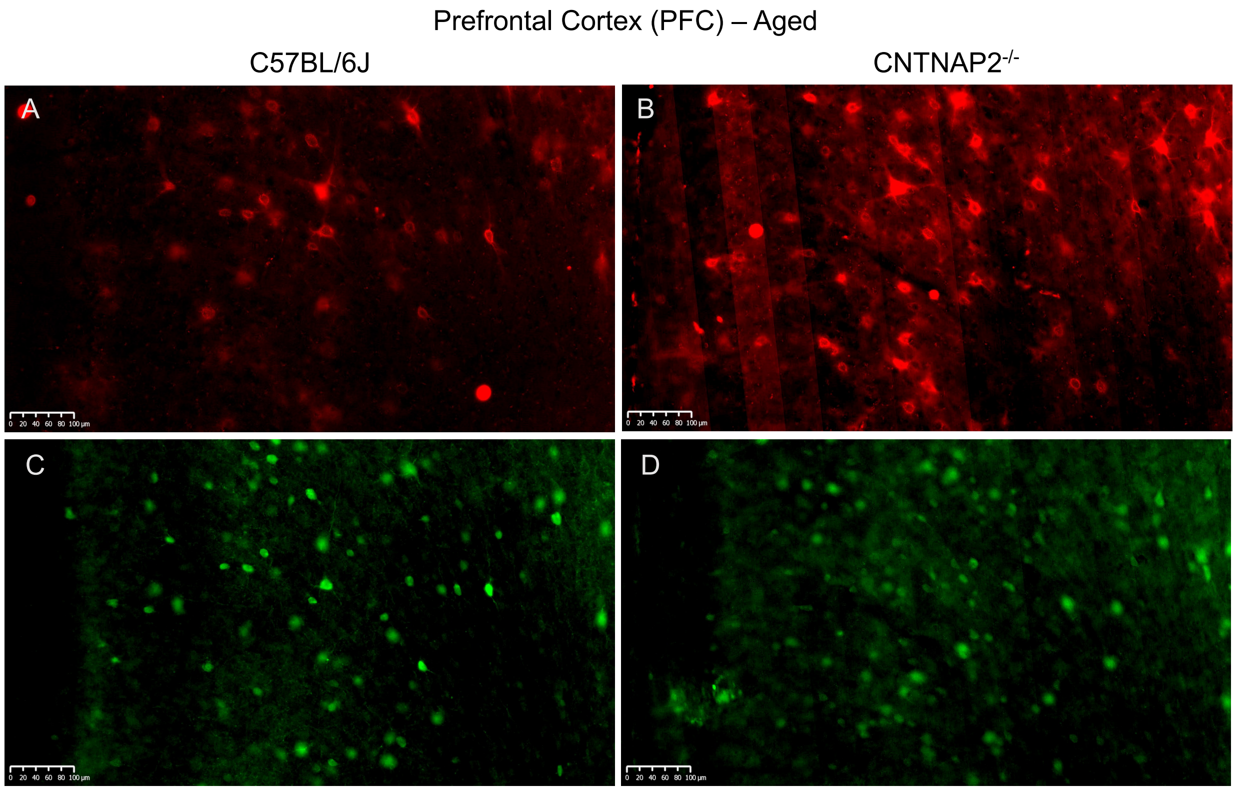

Figure 4. Immunofluorescence images depicting (A, B) PNNs (red) and, (C, D) PV-positive neurons (green) in prefrontal cortex (PFC) of aged C57BL/6J and CNTNAP2$^{-/-}$ mice. Scale bar 100 μm.

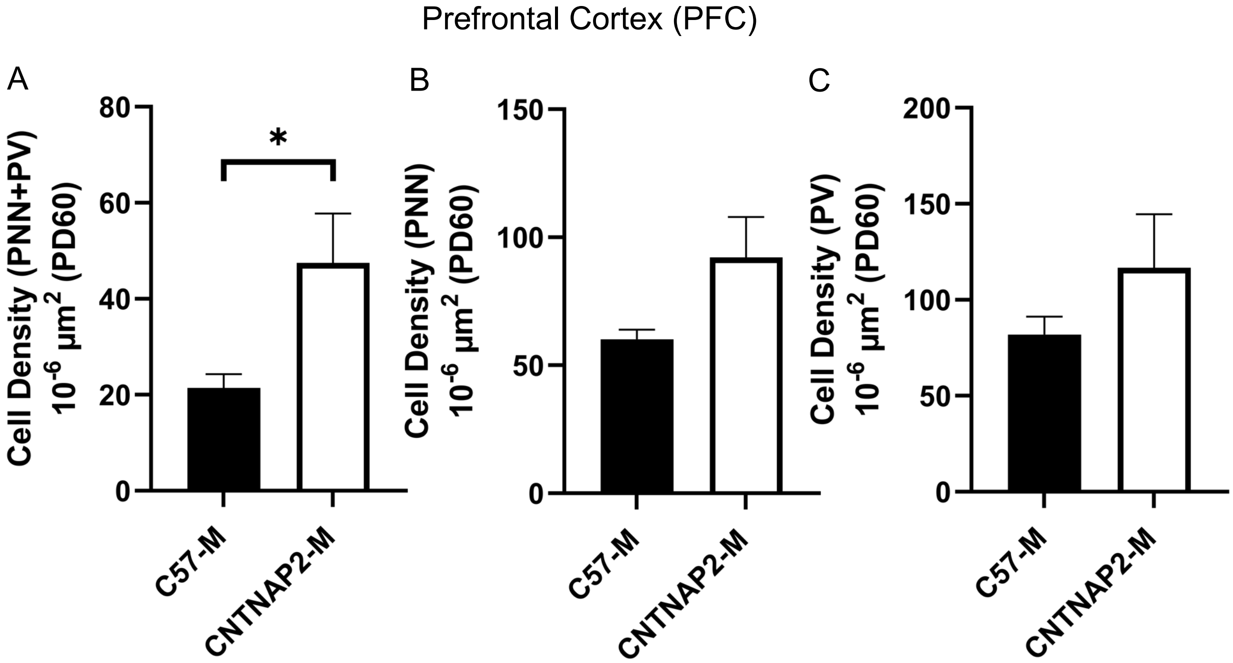

Figure 5. For *male* PD 60 mice, quantitative estimation of (A) PNNs co-localized with PV+ neurons, (B) PNNs and (C) PV+ cells in prefrontal cortex (PFC) of male C57BL/6J (n=4) and CNTNAP2$^{-/-}$ (n=3) mice at PD 60. Significant increase in density of co-localized cells in male CNTNAP2$^{-/-}$ mice in PFC region at PD60. Data expressed as mean ± SEM ($p<0.05$) ($p<0.1$).

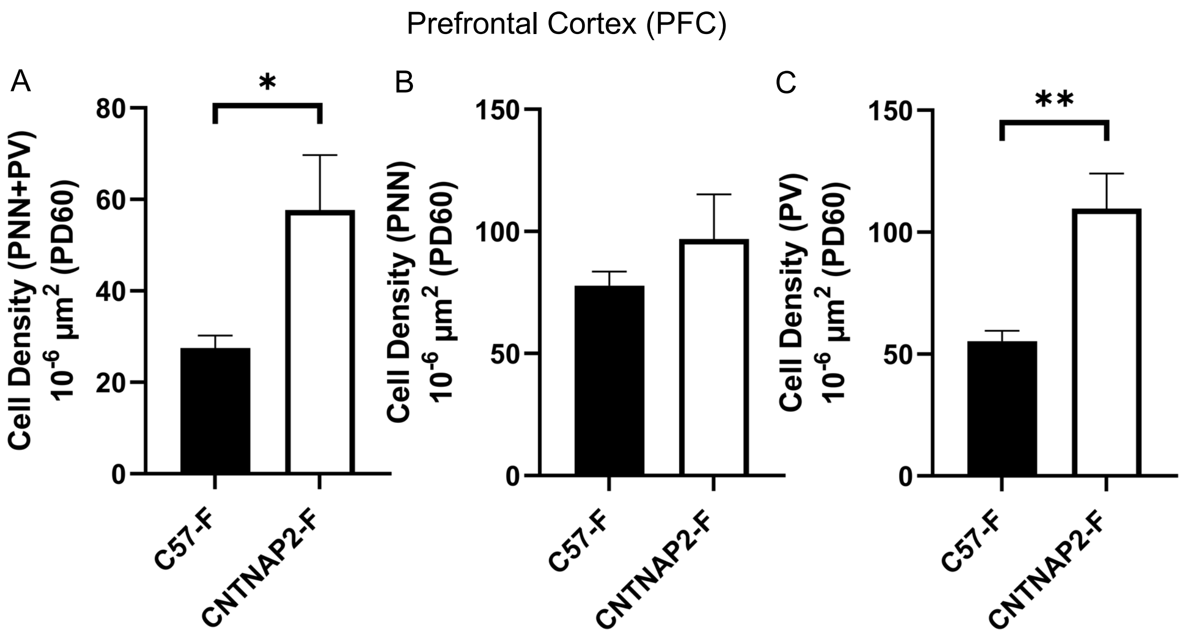

Figure 6. For *female* PD 60 mice, quantitative estimation of (A) PNNs co-localized with PV+ neurons, (B) PNNs and (C) PV+ cells in prefrontal cortex (PFC) of female C57BL/6J (n=4) and CNTNAP2$^{-/-}$ (n=3) mice at PD 60. Significant increase in density of co-localized cells in female CNTNAP2$^{-/-}$ mice in PFC region at PD60. Data expressed as mean ± SEM ($p<0.05$).

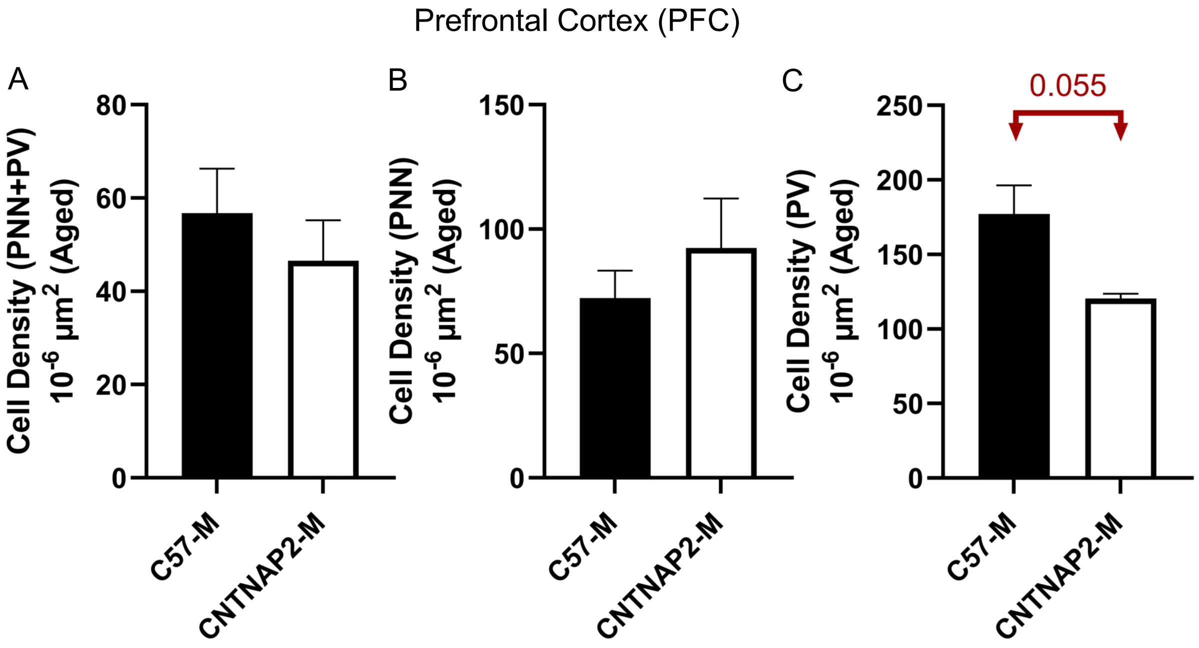

Figure 7. For *male* aged mice, quantitative estimation of (A) PNNs co-localized with PV+ neurons, (B) PNNs and (C) PV+ cells in prefrontal cortex (PFC) of aged male C57BL/6J (n=3) and CNTNAP2$^{-/-}$ (n=3) mice. Data expressed as mean ± SEM ($p<0.05$) ($p<0.1$).

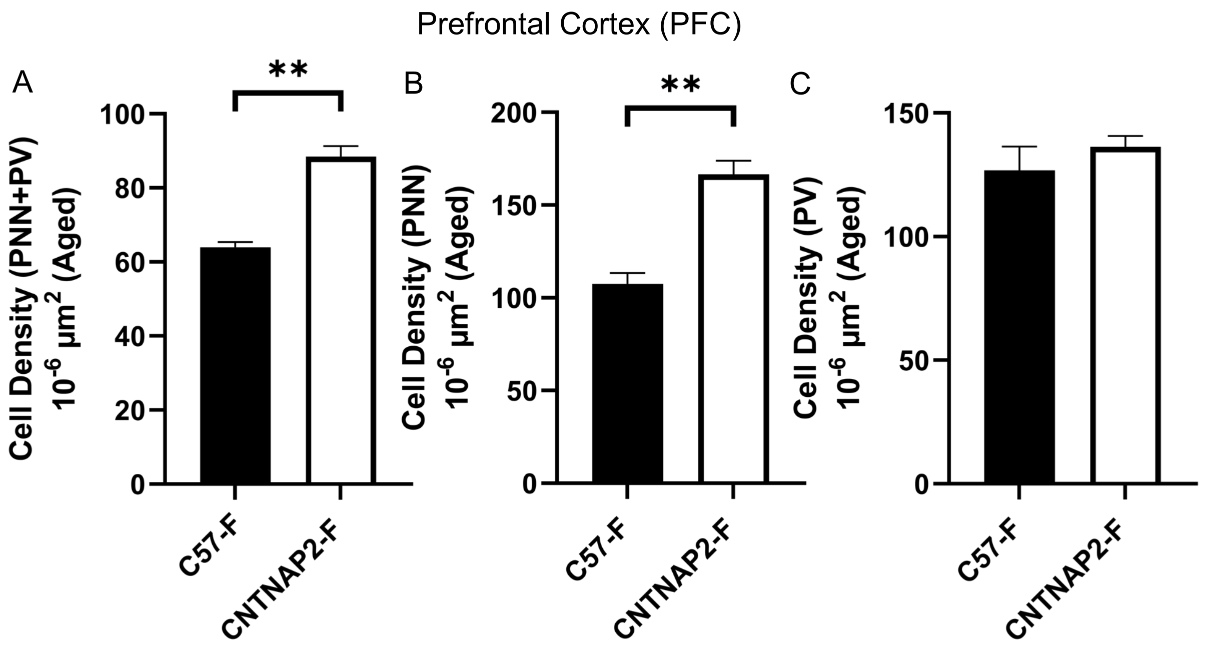

Figure 8. For *female* aged mice, quantitative estimation of (A) PNNs co-localized with PV+ neurons, (B) PNNs and (C) PV+ cells in prefrontal cortex (PFC) of aged female C57BL/6J (n=3) and CNTNAP2$^{-/-}$ (n=3) mice. Data expressed as mean ± SEM (p<0.05) (p<0.1).

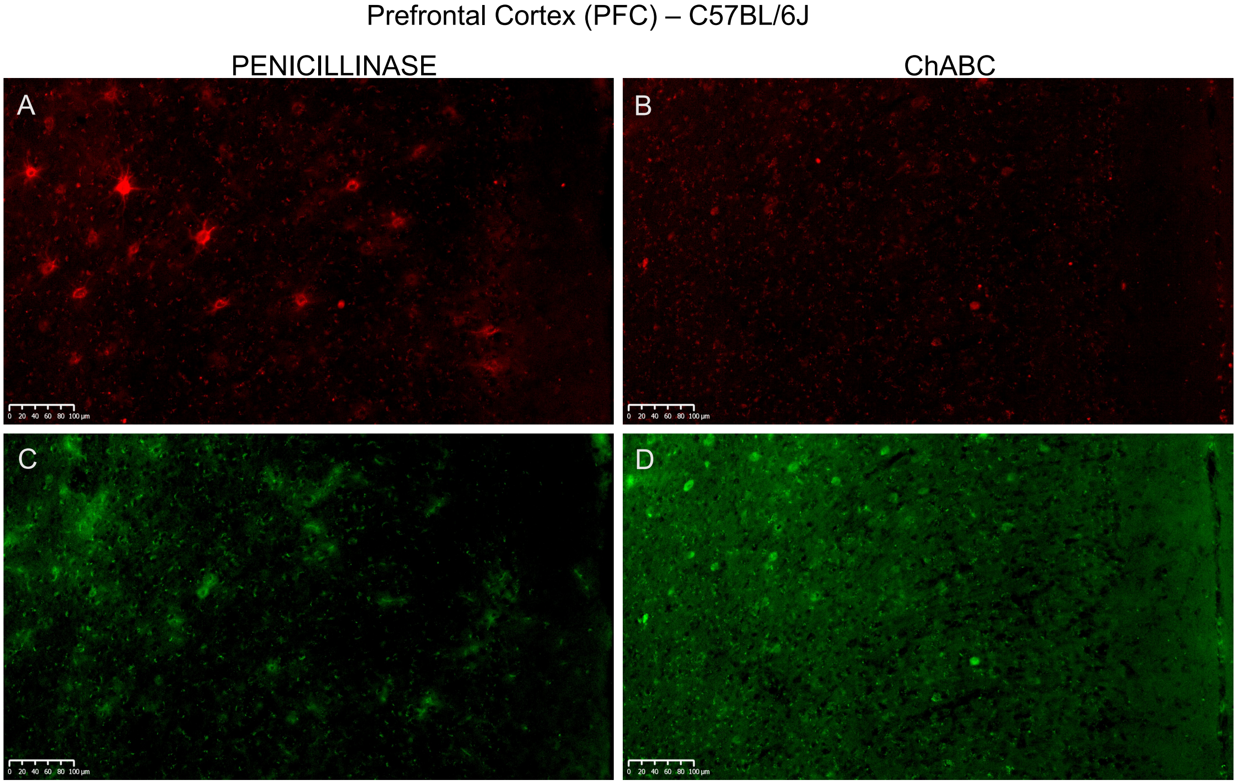

Figure 9. Immunofluorescence images depicting distribution of (A, B) PNNs (red) and, (C, D) PV-positive neurons (green) in prefrontal cortex (PFC) of C57BL/6J mice after penicillinase and ChABC injections. ChABC treatment digested PNNs in the PFC region. Scale bar 100 µm.

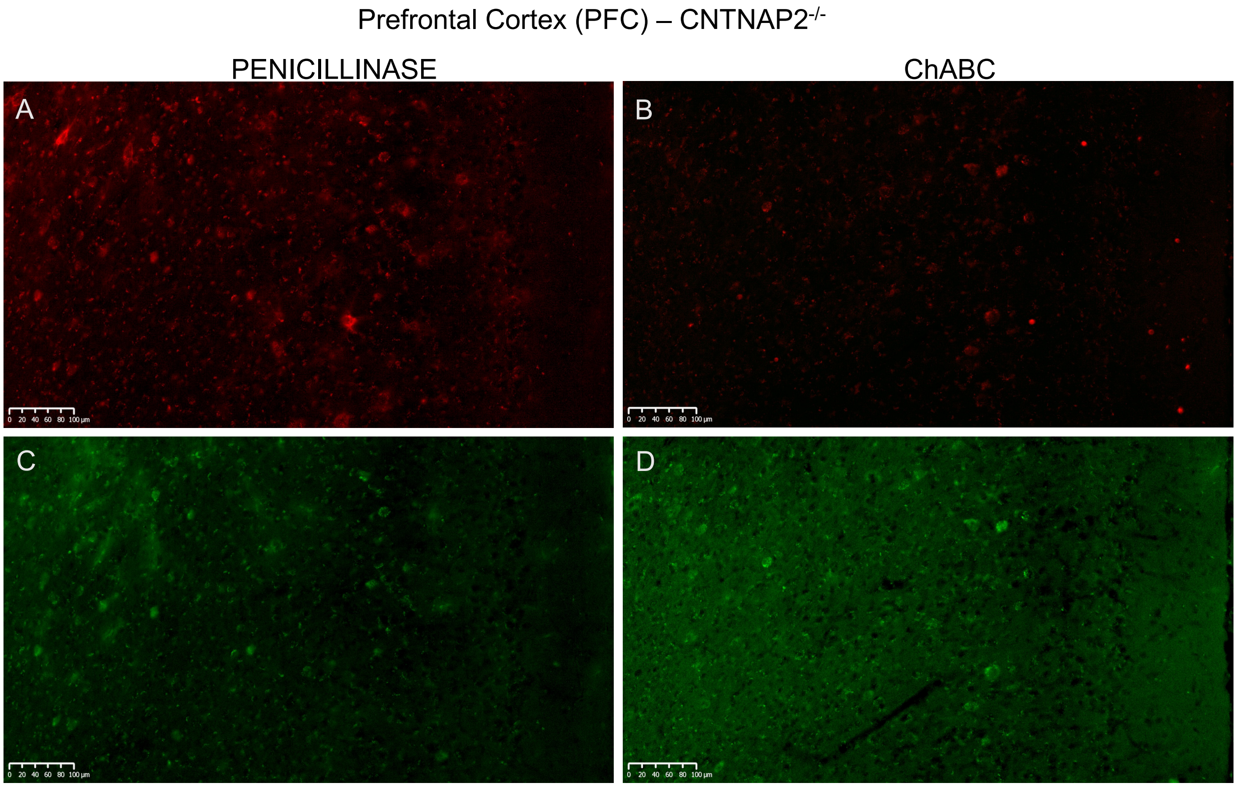

Figure 10. Immunofluorescence images depicting distribution of (A, B) PNNs (red) and, (C, D) PV-positive neurons (green) in prefrontal cortex (PFC) of CNTNAP2$^{-/-}$ mice after penicillinase and ChABC injections. ChABC treatment digested PNNs in the PFC region. Scale bar 100 µm.

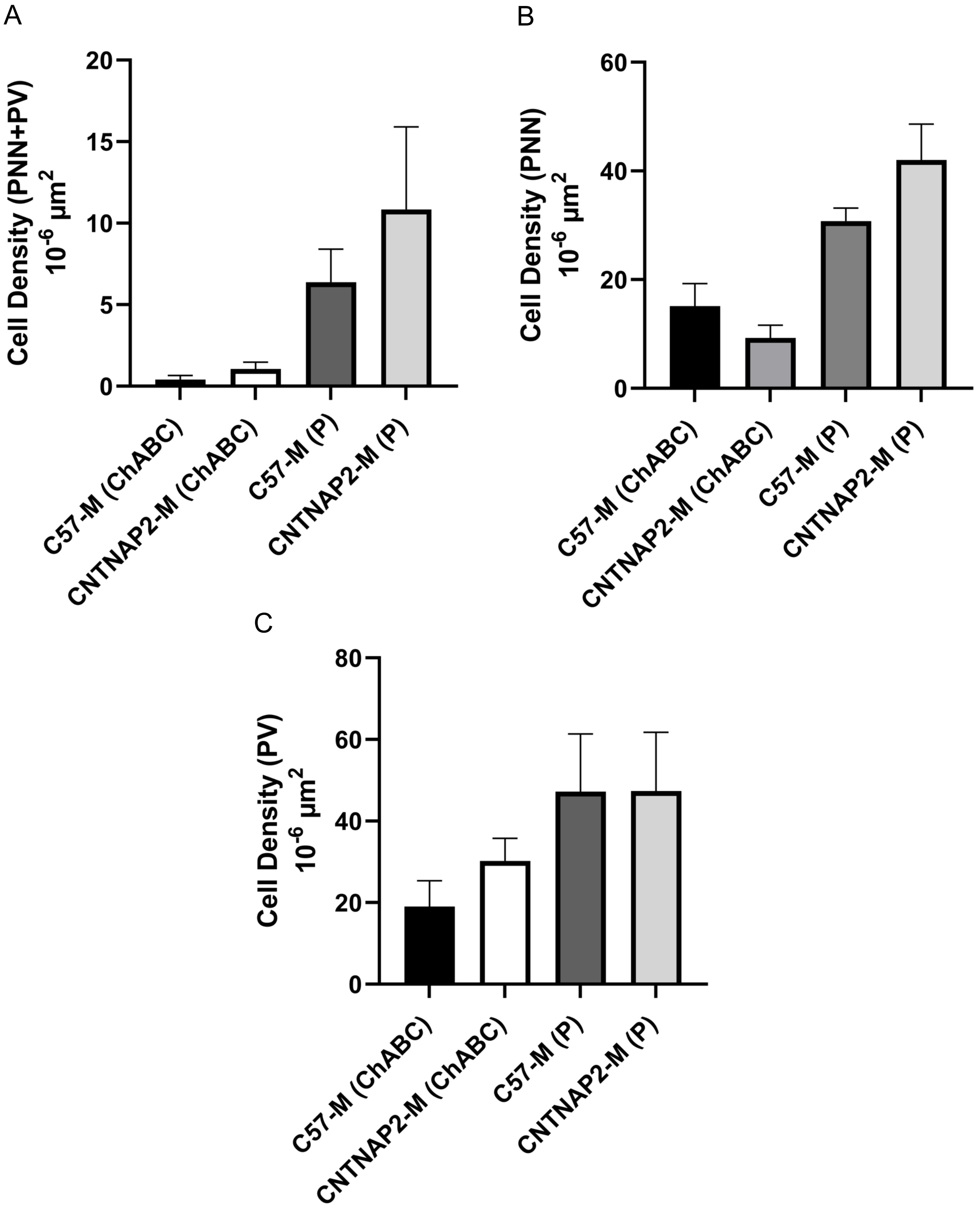

Figure 11. In *male* PD 60 mice, quantitative estimation of (A) PNNs co-localized with PV+ neurons, (B) PNNs and (C) PV+ cells in prefrontal cortex (PFC) of male C57BL/6J and CNTNAP2$^{-/-}$ mice treated with chondroitinase (C57BL/6J, n=5; CNTNAP2$^{-/-}$, n=7) and penicillinase (C57BL/6J, n=6; CNTNAP2$^{-/-}$, n=4). ChABC treatment reduced density of PNNs and co-localized cells in the PFC region in male mice. Data expressed as mean ± SEM ($p<0.05$) ($p<0.1$). Two-way ANOVA and Fisher LSD post hoc comparisons were performed with significant treatment effects (A: $F_{1, 18} = 26.68$, $p < .0001$; B: $F_{1, 18} = 43.91$, $p < .0001$; C: $F_{1, 18} = 5.44$, $p = 0.0315$) on all three.

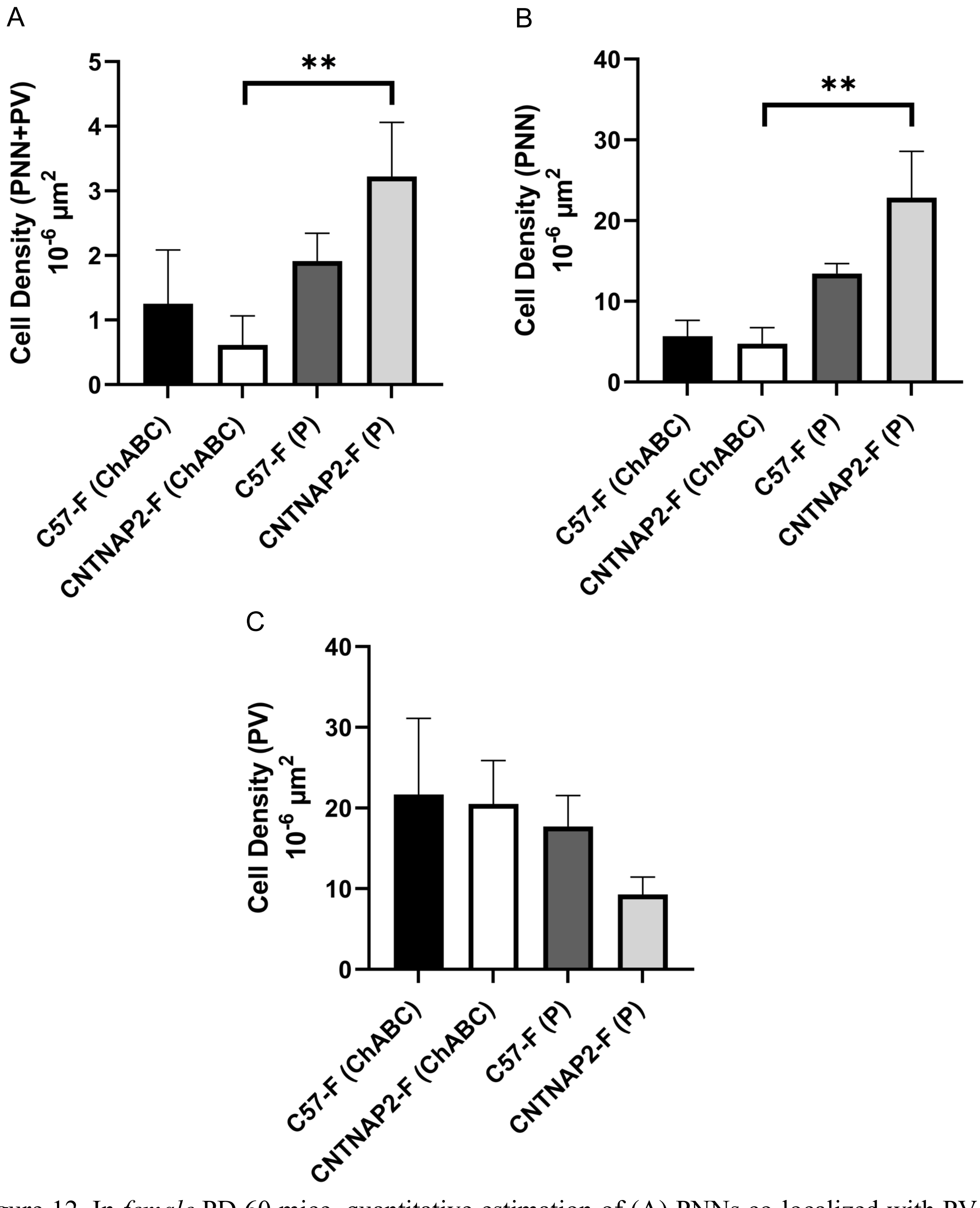

Figure 12. In *female* PD 60 mice, quantitative estimation of (A) PNNs co-localized with PV+ neurons, (B) PNNs and (C) PV+ cells in prefrontal cortex (PFC) of female C57BL/6J and CNTNAP2$^{-/-}$ mice treated with chondroitinase (C57BL/6J, n=6; CNTNAP2$^{-/-}$, n=6) and penicillinase (C57BL/6J, n=4; CNTNAP2$^{-/-}$, n=6). ChABC treatment reduced density of PNNs and co-localized cells in the PFC region in female mice. Data expressed as mean ± SEM ($p<0.05$) ($p<0.1$). Two-way ANOVA and Fisher LSD post hoc comparisons were performed with significant treatment effects (A: $F_{1, 18} = 9.21$, p =0.0071; B: $F_{1, 18} = 12.99$, p =0.002; C: $F_{1, 18} = 0.74$, p = 0.402) on (A) and (B).

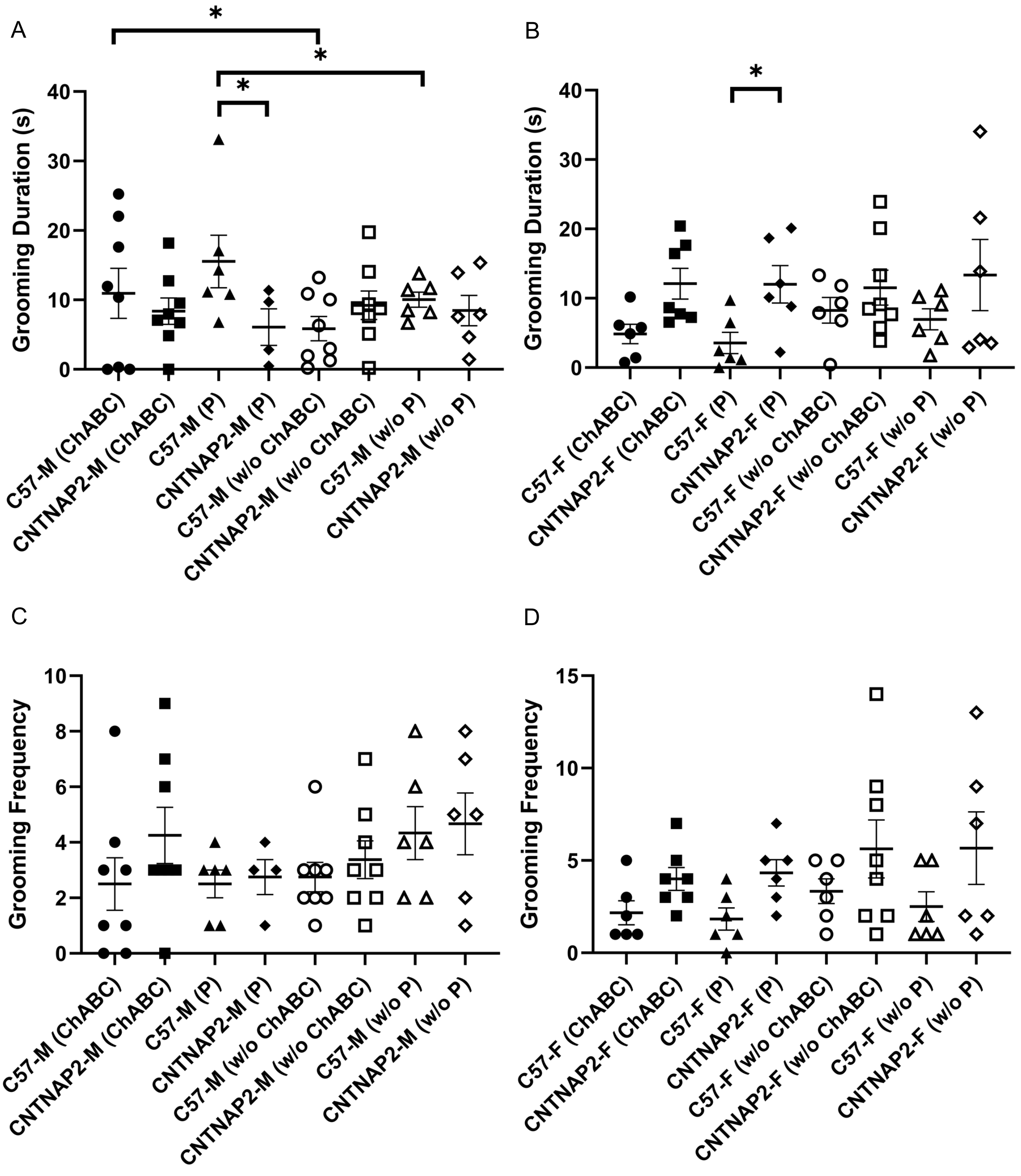

Figure 13. Gender analyses of (A, B) grooming duration and (C, D) frequency in male and female C57BL/6J and CNTNAP2$^{-/-}$ mice. C57BL/6J mice treated with chondroitinase (n=8 males; n=6 females) and penicillinase (n=6 males; n=6 females). CNTNAP2$^{-/-}$ mice treated with chondroitinase (n=8 males; n=7 females) and penicillinase (n=4 males; n=6 females). Data expressed as mean ± SEM (p<0.05) (p<0.1). Mixed ANOVA and Fisher LSD post hoc comparisons were performed with 3-way interactions (A: $F_{1, 23.2} = 0.10$, p = 0.7593; B: $F_{1, 21.8} = 0.07$, p = 0.7912; C: $F_{1, 22.5} = 0.59$, p = 0.4498; D: $F_{1, 21.9} = 0.004$, p = 0.9475).

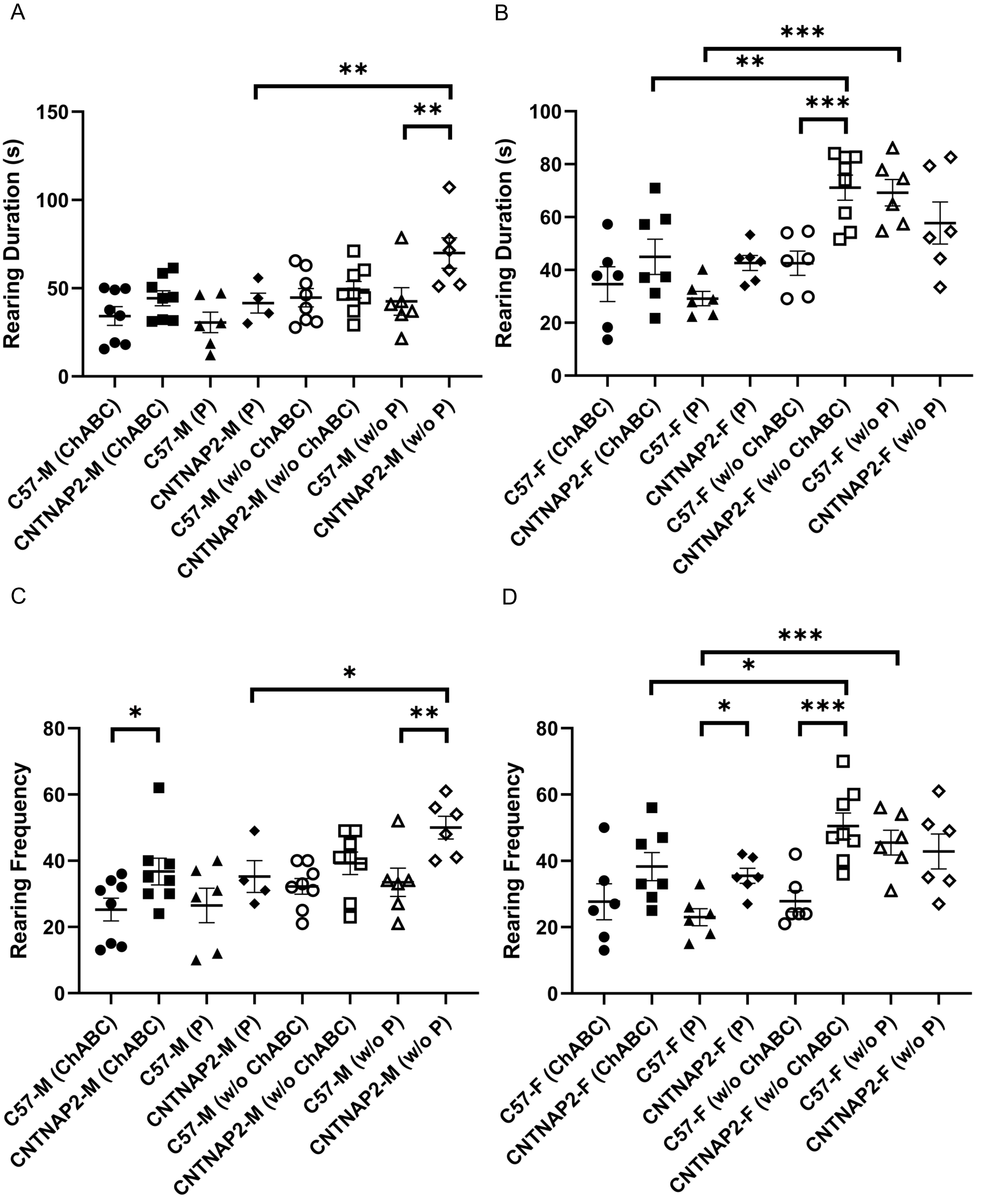

Figure 14. Gender analyses of (A, B) rearing duration and (C, D) frequency in male and female C57BL/6J and CNTNAP2$^{-/-}$ mice. C57BL/6J mice treated with chondroitinase (n=8 males; n=6 females) and penicillinase (n=6 males; n=6 females). CNTNAP2$^{-/-}$ mice treated with chondroitinase (n=8 males; n=7 females) and penicillinase (n=4 males; n=6 females). Data expressed as mean ± SEM (p<0.05) (p<0.1). Mixed ANOVA and Fisher LSD post hoc comparisons were performed with 3-way interactions (A: $F_{1, 24.8} = 1.61$, $p = 0.2167$; B: $F_{1, 22.1} = 6.51$, $p = 0.0181$; C: $F_{1, 24.7} = 1.35$, $p = 0.2571$; D: $F_{1, 21.9} = 5.77$, $p = 0.0252$).

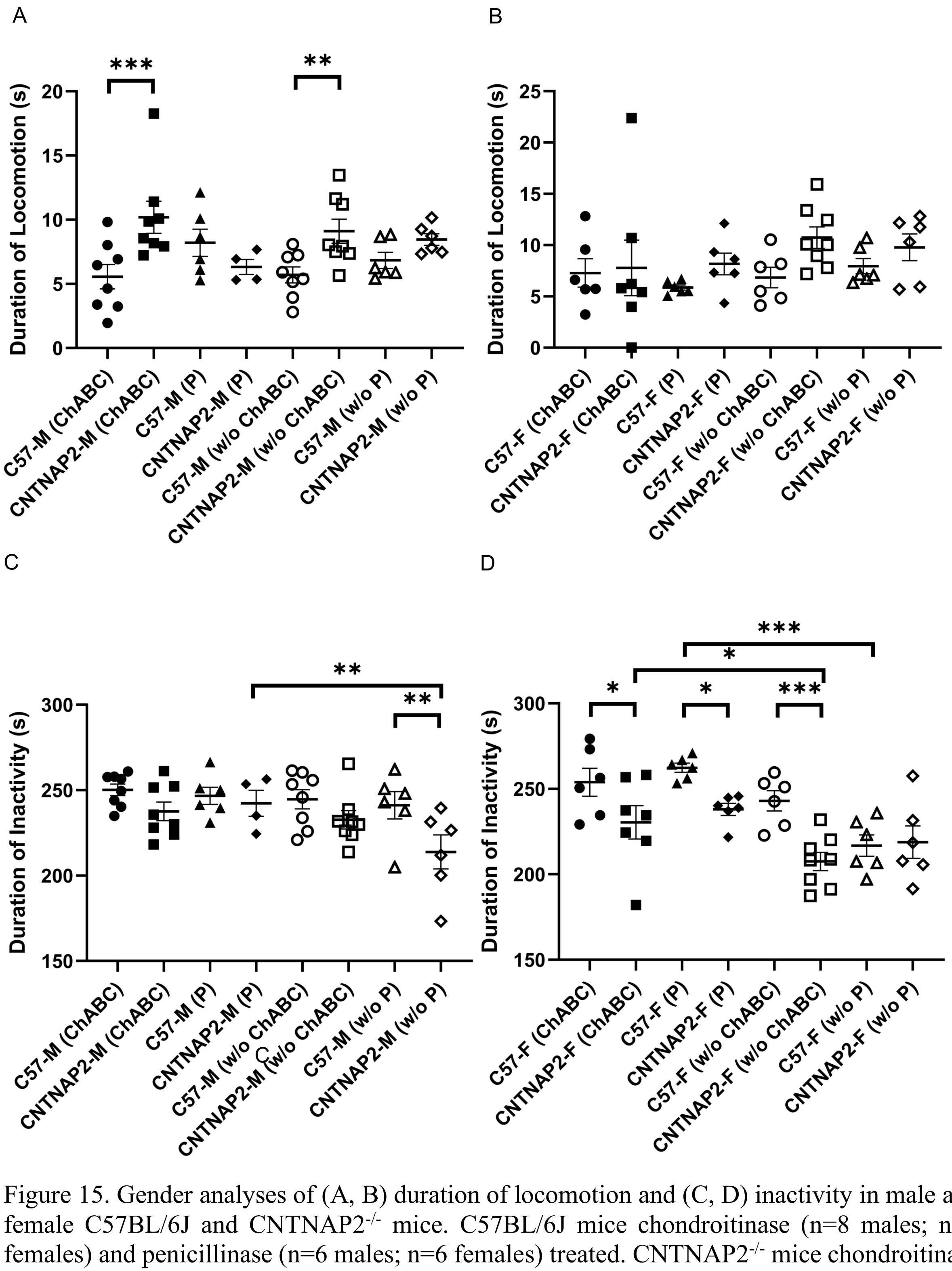

Figure 15. Gender analyses of (A, B) duration of locomotion and (C, D) inactivity in male and female C57BL/6J and CNTNAP2$^{-/-}$ mice. C57BL/6J mice chondroitinase (n=8 males; n=6 females) and penicillinase (n=6 males; n=6 females) treated. CNTNAP2$^{-/-}$ mice chondroitinase (n=8 males; n=7 females) and penicillinase (n=4 males; n=6 females) treated. Data expressed as mean ± SEM ($p<0.05$) ($p<0.1$). Mixed ANOVA and Fisher LSD post hoc comparisons were performed with 3-way interactions (A: $F_{1, 24.3} = 4.70$, p = 0.0402; B: $F_{1, 22.1} = 0.82$, p = 0.03751; C: $F_{1, 24.2} = 1.96$, p = 0.1737; D: $F_{1, 22.1} = 3.37$, p = 0.0798).

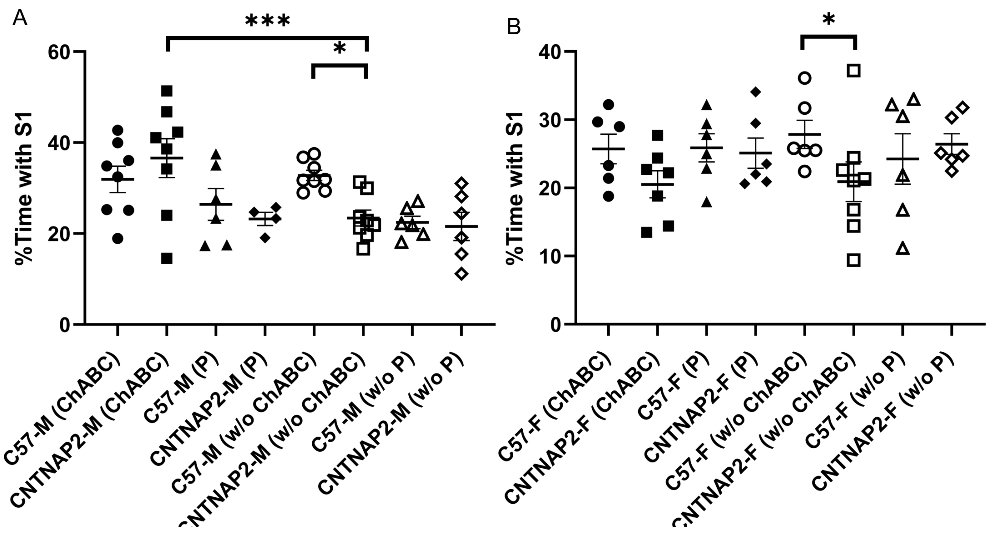

Figure 16. Three-chamber sociability performance (A) percentage time spent with stranger mice in male and, (B) female C57BL/6J and CNTNAP2$^{-/-}$ mice. C57BL/6J mice treated with chondroitinase (n=8 males; n=6 females) and penicillinase (n=6 males; n=6 females). CNTNAP2$^{-/-}$ mice treated with chondroitinase (n=8 males; n=7 females) and penicillinase (n=4 males; n=6 females). Data expressed as mean ± SEM (p<0.05) (p<0.1). Mixed ANOVA and Fisher LSD post hoc comparisons were performed with 3-way interactions (A: $F_{1, 23.7} = 4.71$, p = 0.0402; B: $F_{1, 22.6} = 0.63$, p = 0.4338).

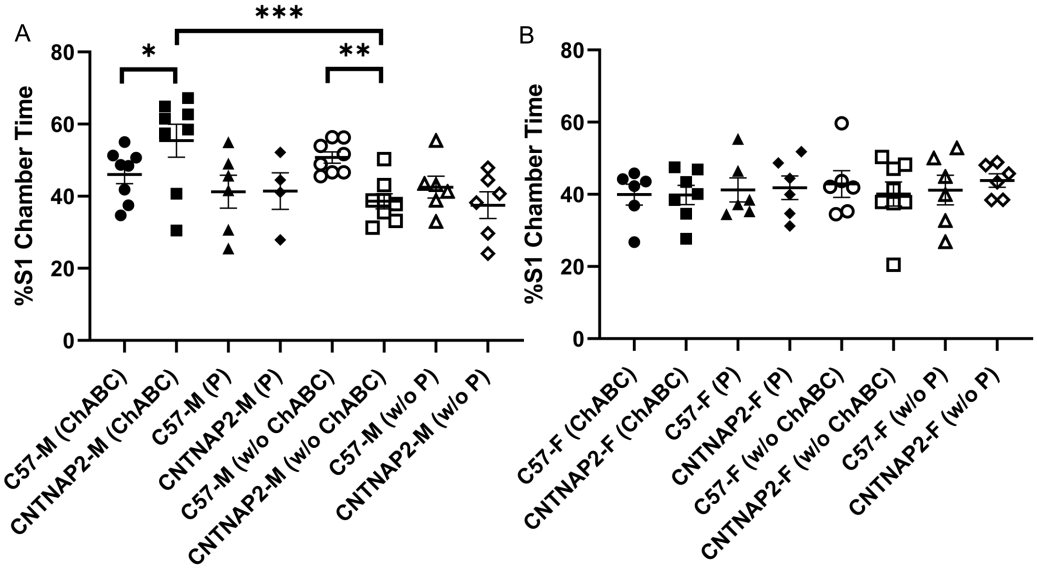

Figure 17. Gender analysis of three-chamber sociability performance percentage time spent in chamber housing stranger mice in (A) male and (B) female C57BL/6J and CNTNAP2$^{-/-}$ mice. C57BL/6J mice treated with chondroitinase (n=8 males; n=6 females) and penicillinase (n=6 males; n=6 females). CNTNAP2$^{-/-}$ mice treated with chondroitinase (n=8 males; n=7 females) and penicillinase (n=4 males; n=6 females). Data expressed as mean ± SEM (p<0.05) (p<0.1). Mixed ANOVA and Fisher LSD post hoc comparisons were performed with 3-way interactions (A: $F_{1, 23.3} = 3.41$, p = 0.0775; B: $F_{1, 22.5} = 0.31$, p = 0.5845).

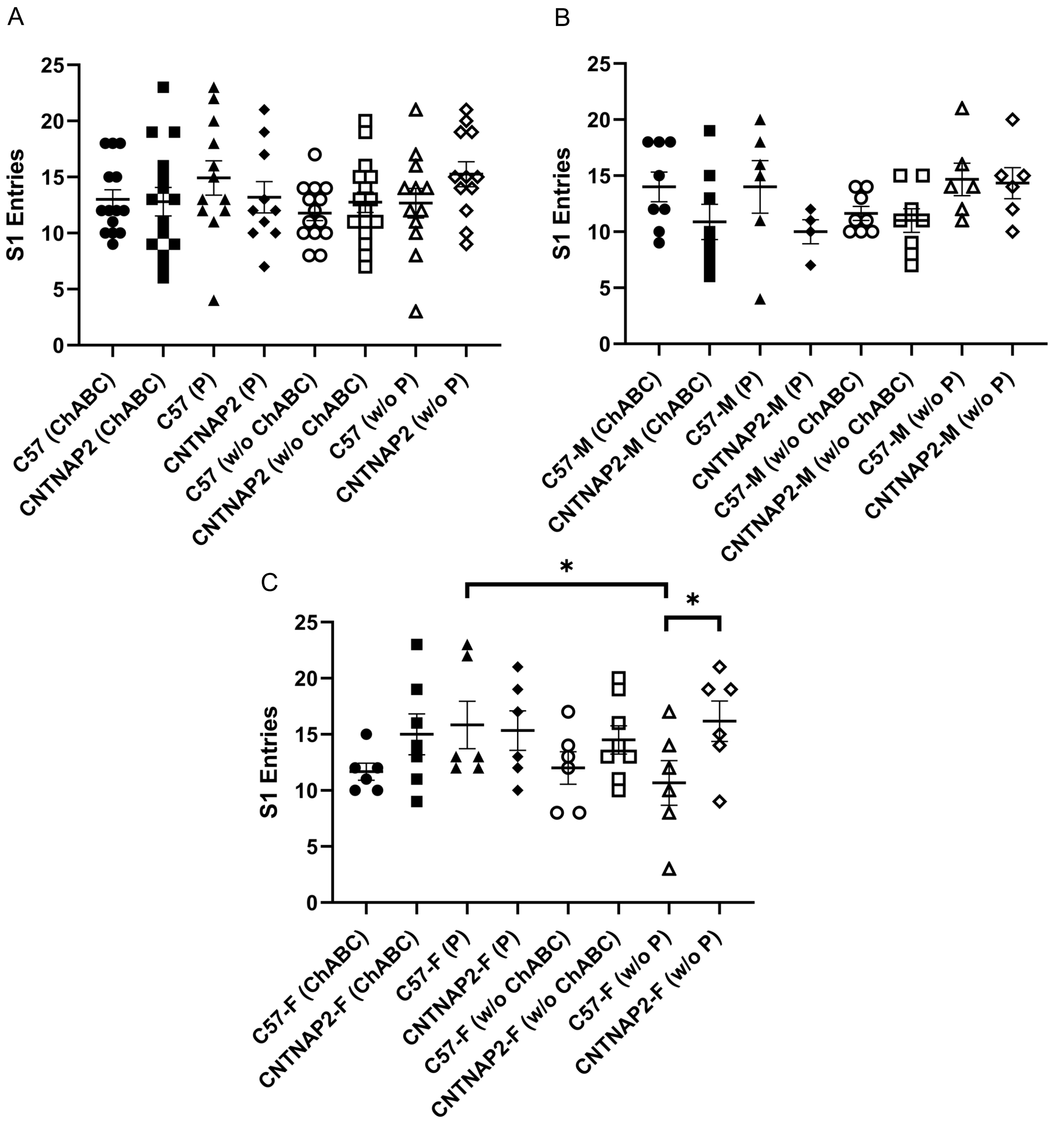

Figure 18. Gender analysis of S1 entries in the three-chamber sociability performance. (A) Entries in chamber housing stranger mice in combined male and female, (B) male and (C) female C57BL/6J and $CNTNAP2^{-/-}$ mice. C57BL/6J mice treated with chondroitinase (n=8 males; n=6 females) and penicillinase (n=6 males; n=6 females). $CNTNAP2^{-/-}$ mice treated with chondroitinase (n=8 males; n=7 females) and penicillinase (n=4 males; n=6 females). Data expressed as mean ± SEM (p<0.05) (p<0.1). Mixed ANOVA and Fisher LSD post hoc comparisons were performed with 3-way interactions (A: $F_{1, 49.0} = 1.01$, p = 0.3194; B: $F_{1, 24.4} = 0.06$, p = 0.8081; C: $F_{1, 22} = 3.64$, p = 0.0695).

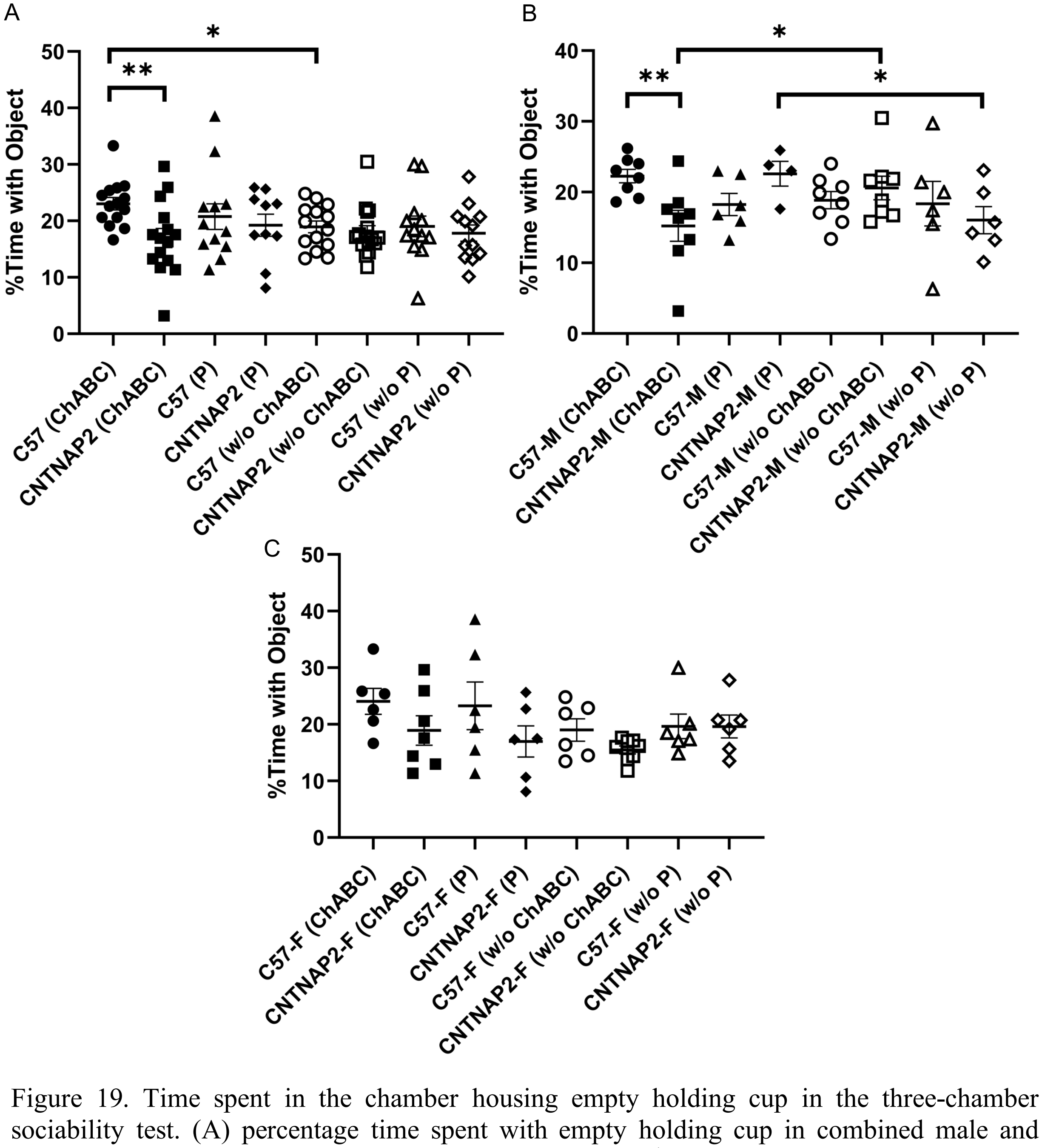

Figure 19. Time spent in the chamber housing empty holding cup in the three-chamber sociability test. (A) percentage time spent with empty holding cup in combined male and female, (B) male and (C) female C57BL/6J and CNTNAP2$^{-/-}$ mice. C57BL/6J mice treated with chondroitinase (n=8 males; n=6 females) and penicillinase (n=6 males; n=6 females). CNTNAP2$^{-/-}$ mice treated with chondroitinase (n=8 males; n=7 females) and penicillinase (n=4 males; n=6 females). Decreased time spent interacting with the empty holding cup after the ChABC treatment in male CNTNAP2$^{-/-}$ mice. Data expressed as mean ± SEM ($p<0.05$) ($p<0.1$). Mixed ANOVA and Fisher LSD post hoc comparisons were performed with 3-way interactions (A: $F_{1, 48.2} = 1.44$, $p = 0.2355$; B: $F_{1, 24.5} = 8.66$, $p = 0.0070$; C: $F_{1, 21.6} = 0.37$, $p = 0.5479$).

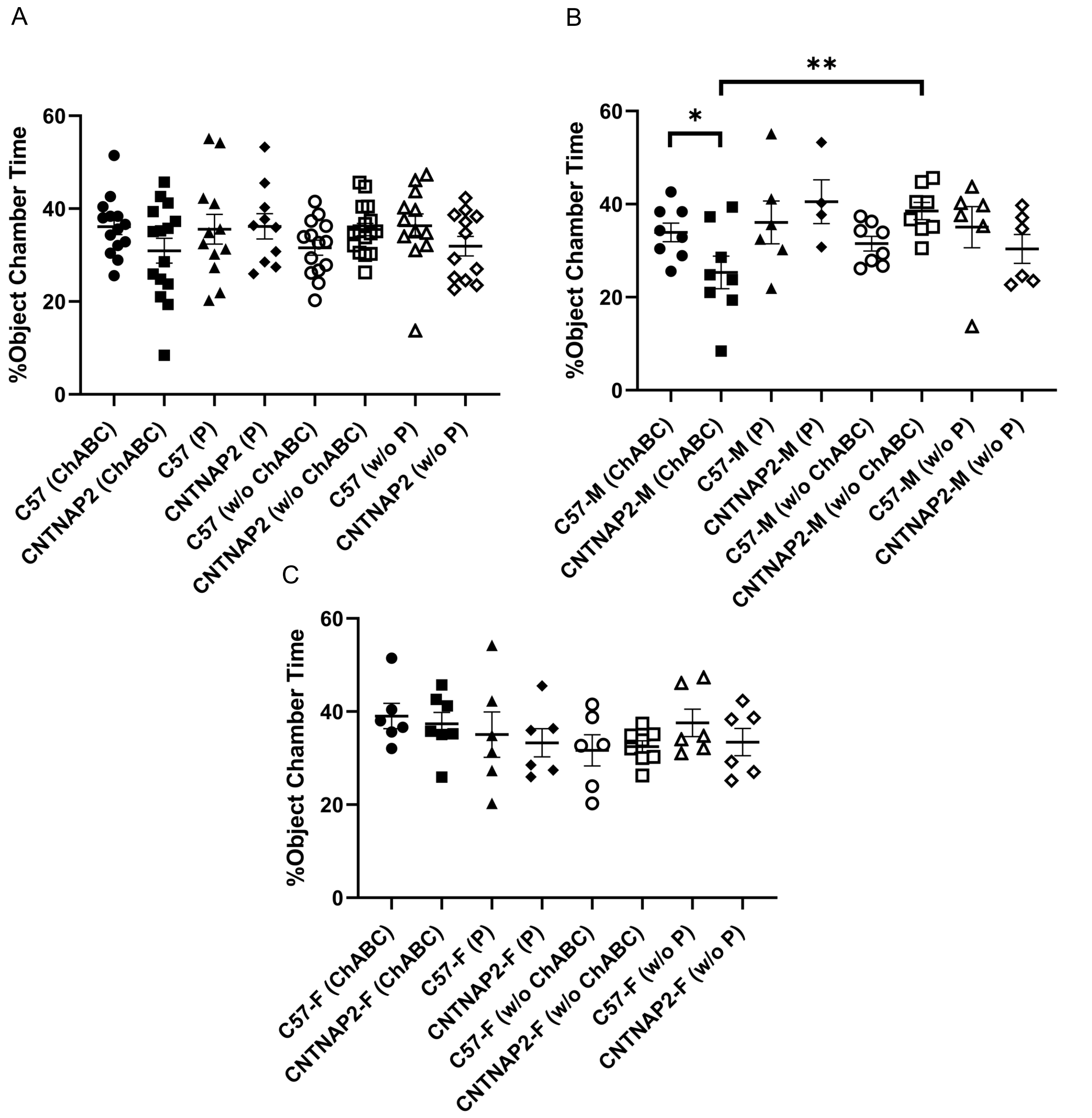

Figure 20. Analysis of empty holding cup chamber time in the three-chamber sociability performance. (A) Percentage time spent in chamber housing the empty cup in combined male and female, (B) male and (C) female C57BL/6J and CNTNAP2$^{-/-}$ mice. C57BL/6J mice treated with chondroitinase (n=8 males; n=6 females) and penicillinase (n=6 males; n=6 females). CNTNAP2$^{-/-}$ mice treated with chondroitinase (n=8 males; n=7 females) and penicillinase (n=4 males; n=6 females). Decreased time spent in the chamber housing the empty cup after ChABC treatment in male CNTNAP2$^{-/-}$ mice. Data expressed as mean ± SEM (p<0.05) (p<0.1). Mixed ANOVA and Fisher LSD post hoc comparisons were performed with 3-way interactions (A: $F_{1,48.8} = 4.75$, p = 0.0342; B: $F_{1,24.5} = 6.97$, p = 0.0142; C: $F_{1,21.8} = 0.74$, p = 0.3972).

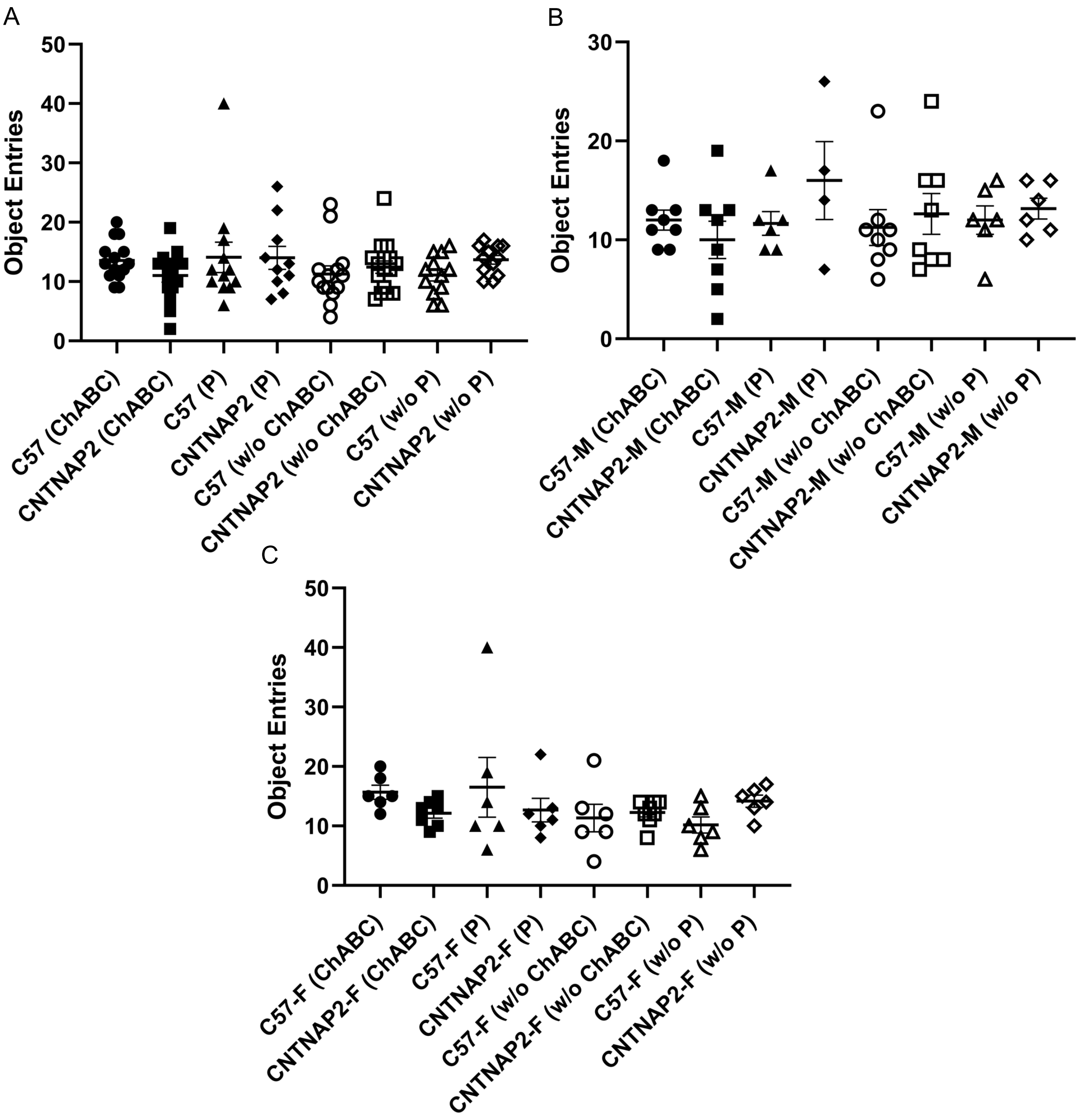

Figure 21. Empty holding cup chamber entries in the three-chamber sociability performance. (A) Entries in chamber housing the empty cup in combined male and female, (B) male and (C) female C57BL/6J and CNTNAP2$^{-/-}$ mice. C57BL/6J mice treated with chondroitinase (n=8 males; n=6 females) and penicillinase (n=6 males; n=6 females). CNTNAP2$^{-/-}$ mice treated with chondroitinase (n=8 males; n=7 females) and penicillinase (n=4 males; n=6 females). No change in empty cup chamber entries after the ChABC treatment. Data expressed as mean ± SEM ($p<0.05$) ($p<0.1$). Mixed ANOVA and Fisher LSD post hoc comparisons were performed with 3-way interactions (A: $F_{1, 50.2} = 0.07$, p = 0.7892; B: $F_{1, 24.2} = 2.36$, p = 0.1376; C: $F_{1, 24} = 0.29$, p = 0.5984).

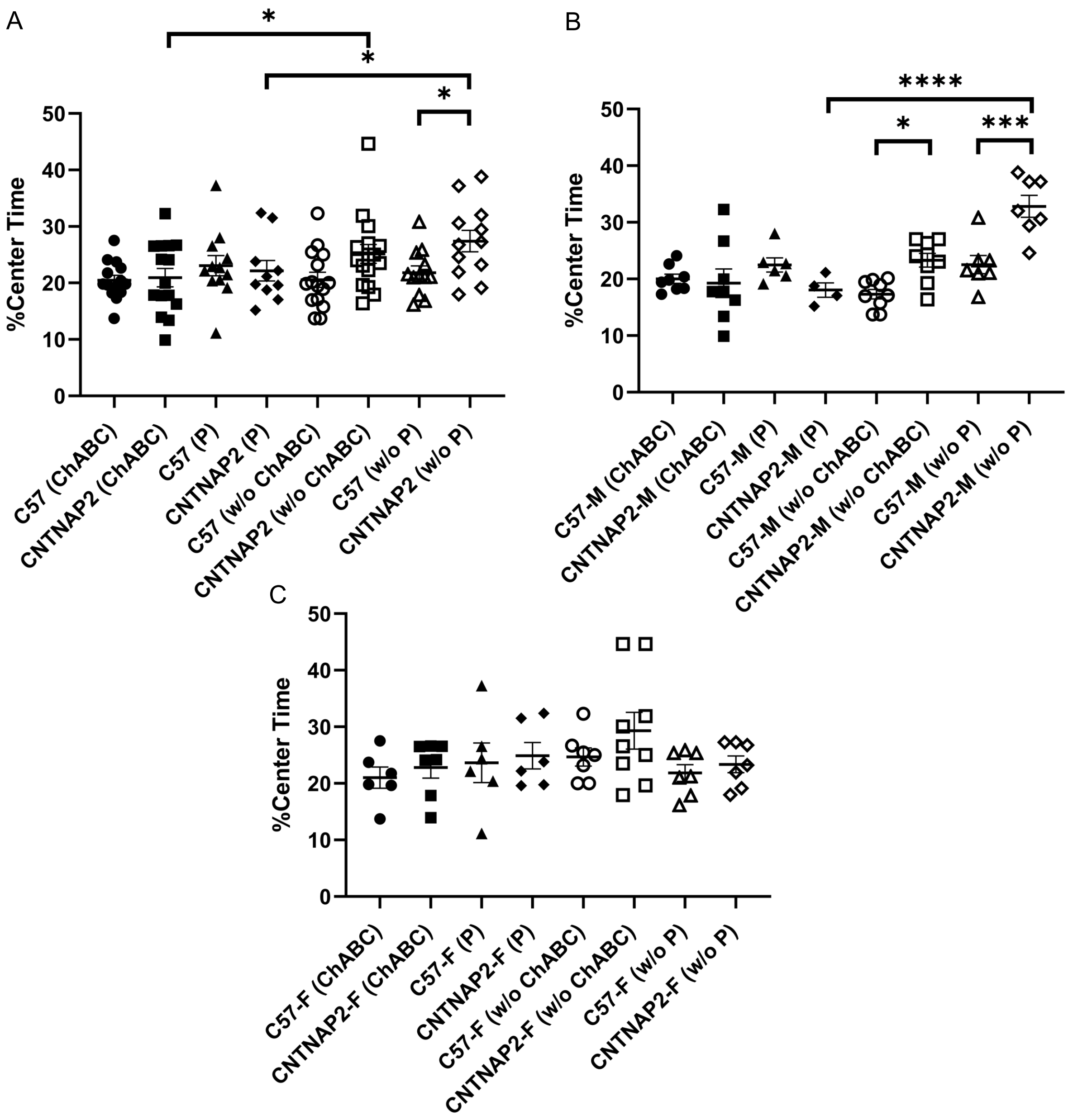

Figure 22. Time spent in center chamber in the three-chamber sociability task. (A) Percentage time spent in the center chamber in combined male and female, (B) male and (C) female C57BL/6J and CNTNAP2$^{-/-}$ mice. C57BL/6J mice treated with chondroitinase (n=8 males; n=6 females) and penicillinase (n=6 males; n=6 females). CNTNAP2$^{-/-}$ mice treated with chondroitinase (n=8 males; n=7 females) and penicillinase (n=4 males; n=6 females). Decreased time spent in the center chamber after the ChABC treatment in CNTNAP2$^{-/-}$ mice. Data expressed as mean ± SEM ($p<0.05$). Mixed ANOVA and Fisher LSD post hoc comparisons were performed with 3-way interactions (A: $F_{1, 47.5} = 0.63$, $p = 0.4292$; B: $F_{1, 23.4} = 3.13$, $p = 0.0898$; C: $F_{1, 22.8} = 0.0.1$, $p = 0.9324$).

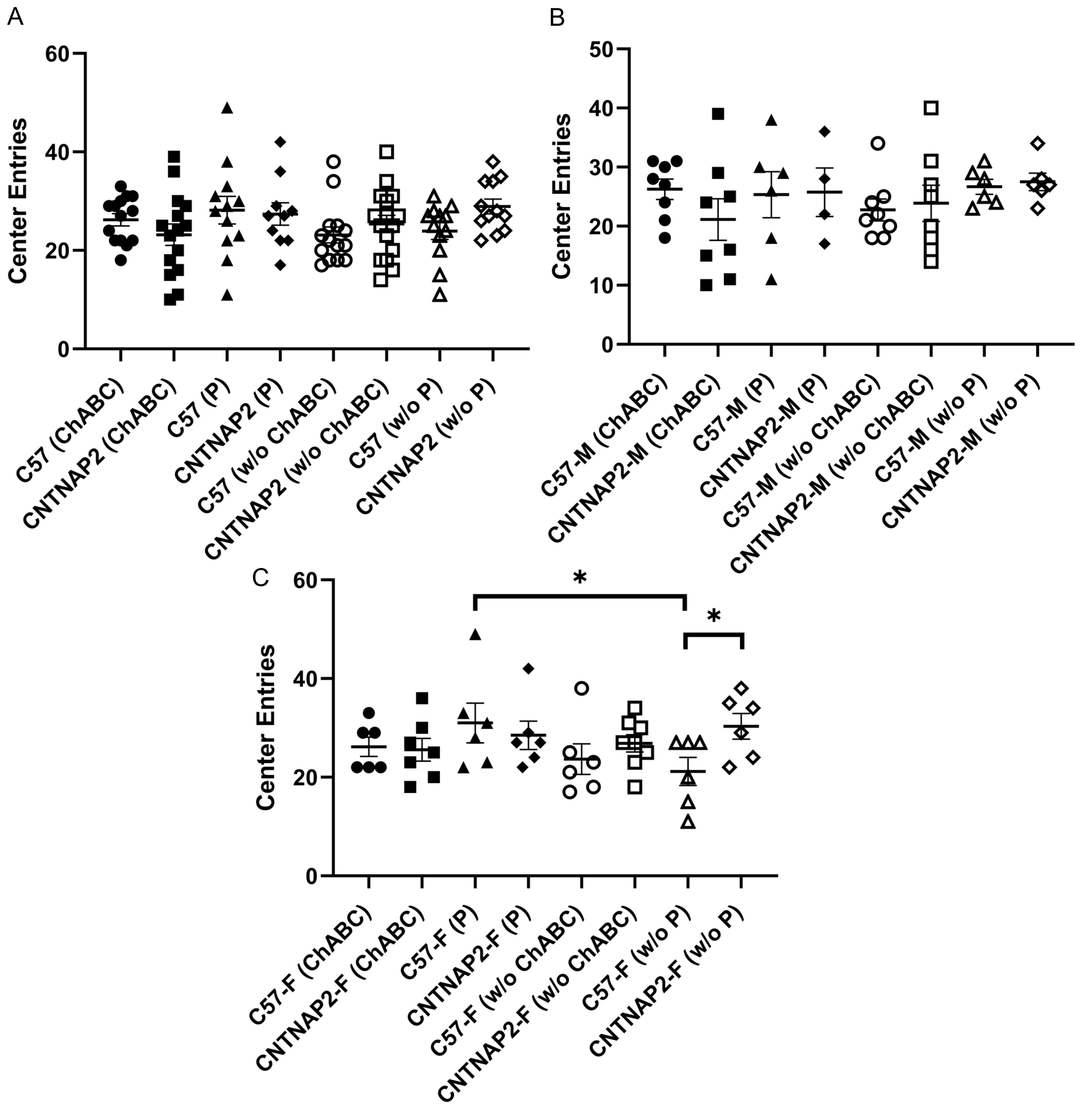

Figure 23. Center entries in the three-chamber sociability task. (A) Entries in the center chamber in combined male and female, (B) male and (C) female C57BL/6J and CNTNAP2$^{-/-}$ mice. C57BL/6J mice treated with chondroitinase (n=8 males; n=6 females) and penicillinase (n=6 males; n=6 females). CNTNAP2$^{-/-}$ mice treated with chondroitinase (n=8 males; n=7 females) and penicillinase (n=4 males; n=6 females). No change in center entries after the ChABC treatment. Data expressed as mean ± SEM ($p<0.05$). Mixed ANOVA and Fisher LSD post hoc comparisons were performed with 3-way interactions (A: $F_{1, 49.1} = 0.01$, $p = 0.9053$; B: $F_{1, 24.1} = 0.86$, $p = 0.3627$; C: $F_{1, 23.8} = 0.94$, $p = 0.343$).

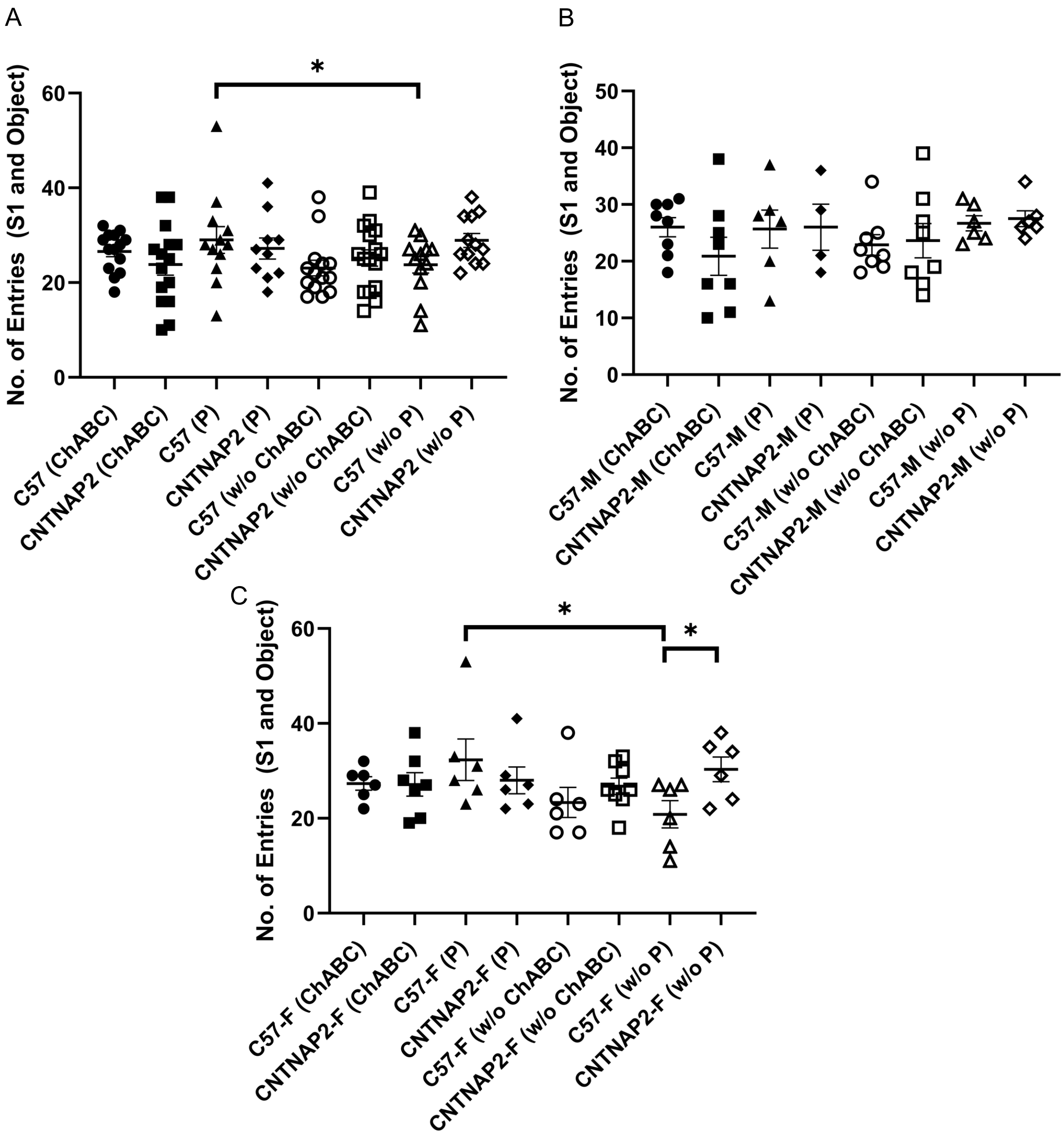

Figure 24. Three-chamber sociability performance (A) total number of entries in the empty cup and stranger mice chambers in combined male and female, (B) male and (C) female C57BL/6J and CNTNAP2$^{-/-}$ mice. C57BL/6J mice treated with chondroitinase (n=8 males; n=6 females) and penicillinase (n=6 males; n=6 females). CNTNAP2$^{-/-}$ mice treated with chondroitinase (n=8 males; n=7 females) and penicillinase (n=4 males; n=6 females). Data expressed as mean ± SEM ($p<0.05$). Mixed ANOVA and Fisher LSD post hoc comparisons were performed with 3-way interactions (A: $F_{1, 49.3} = 0.19$, $p = 0.6666$; B: $F_{1, 24} = 0.83$, $p = 0.3722$; C: $F_{1, 23.9} = 1.59$, $p = 0.2195$).

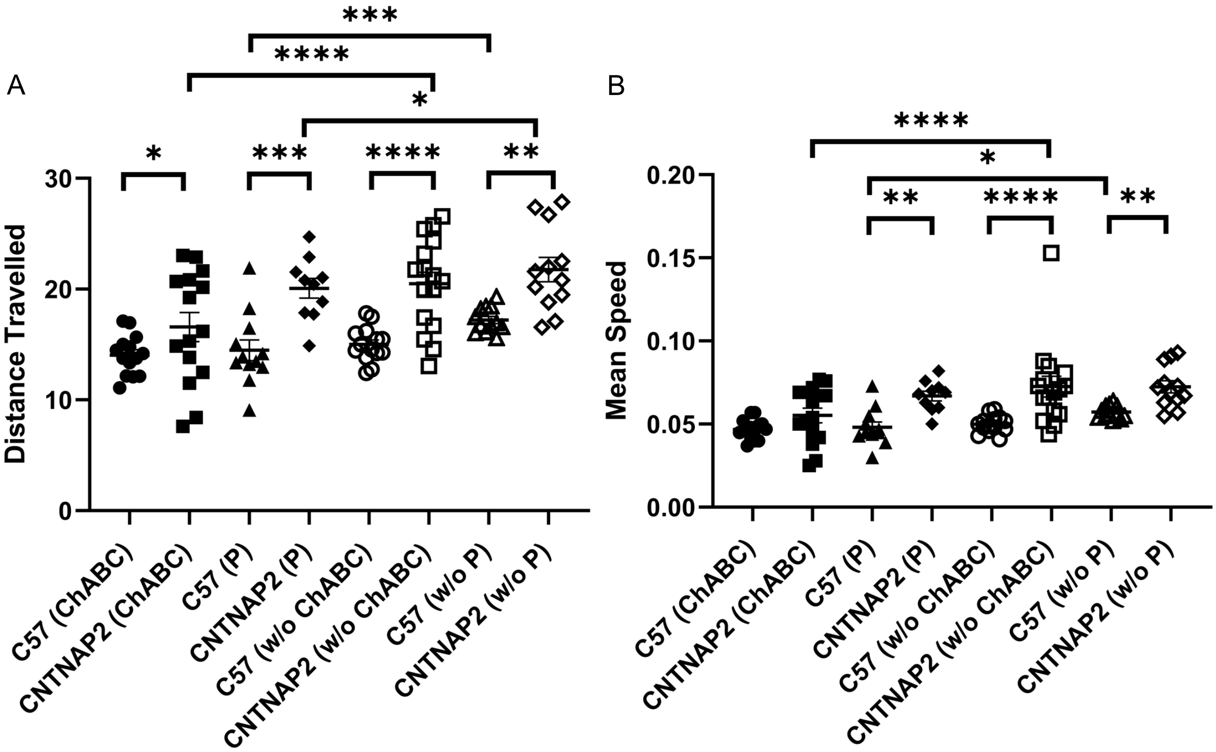

Figure 25. Distance and speed in the three-chamber sociability task. (A) Total distance travelled and (B) mean speed in C57BL/6J and CNTNAP2$^{-/-}$ mice. C57BL/6J mice treated with chondroitinase (n=14) and penicillinase (n=12). CNTNAP2$^{-/-}$ mice treated with chondroitinase (n=15) and penicillinase (n=10). Decrease in distance travelled after the ChABC and penicillinase injections. Data expressed as mean ± SEM (p<0.05) (p<0.1). Mixed ANOVA and Fisher LSD post hoc comparisons were performed with 3-way interactions (A: $F_{1, 47.6} = 5.53$, p = 0.0228; B: $F_{1, 48.6} = 5.44$, p = 0.0239).

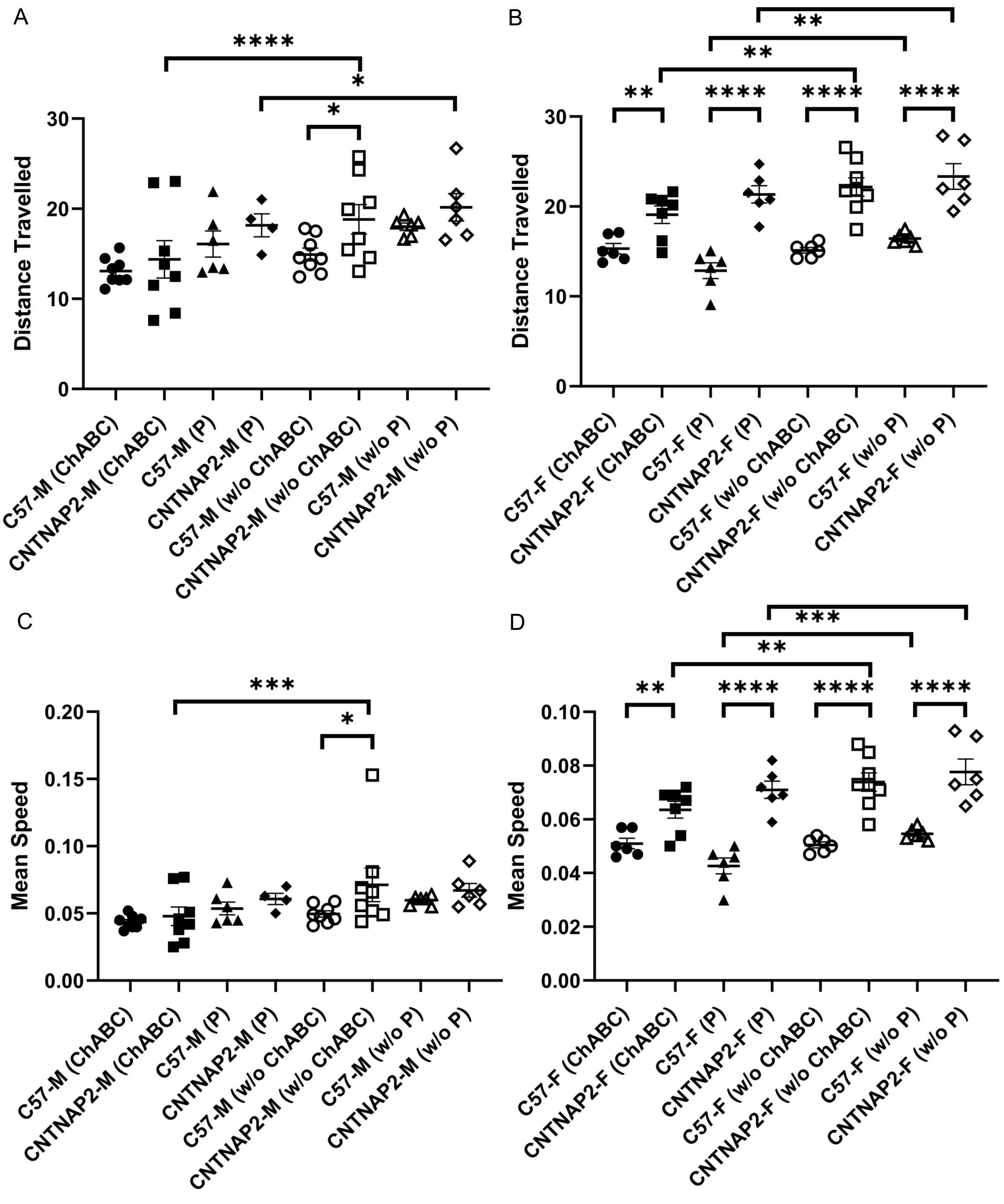

Figure 26. Gender analysis of three-chamber sociability performance in terms of total distance travelled and mean speed in (A, C) male and (B, D) female C57BL/6J and CNTNAP2$^{-/-}$ mice. C57BL/6J mice treated with chondroitinase (n=8 males; n=6 females) and penicillinase (n=6 males; n=6 females). CNTNAP2$^{-/-}$ mice treated with chondroitinase (n=8 males; n=7 females) and penicillinase (n=4 males; n=6 females). Decrease in distance travelled after the ChABC and penicillinase injections. Data expressed as mean ± SEM ($p<0.05$). Mixed ANOVA and Fisher LSD post hoc comparisons were performed with 3-way interactions (A: $F_{1, 22.7} = 0.60$, p = 0.4458; B: $F_{1, 19.5} = 4.67$, p = 0.0423; C: $F_{1, 23.3} = 1.53$, p = 0.2287; D: $F_{1, 19.6} = 4.67$, p = 0.0431).

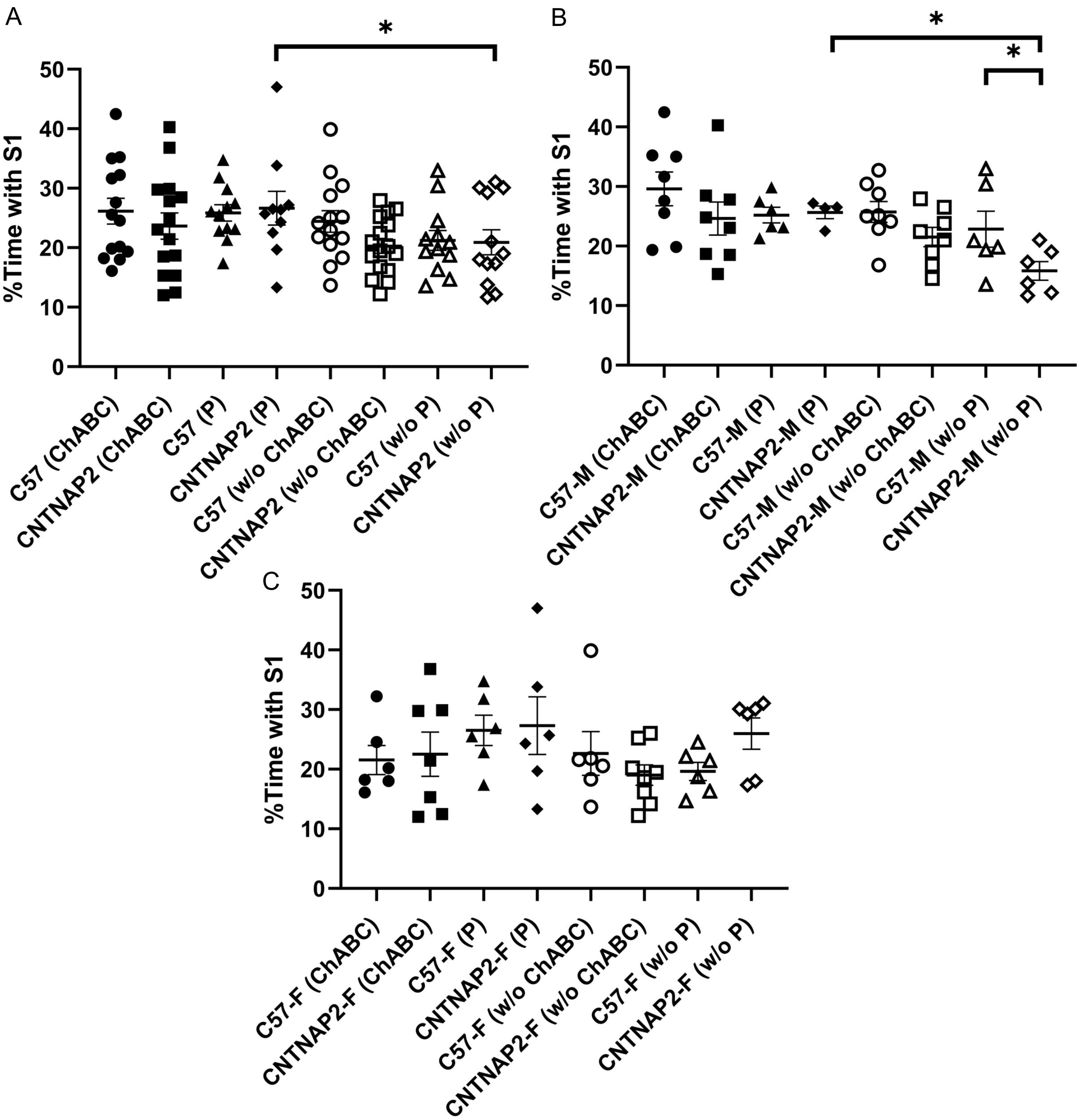

Figure 27. Three-chamber social novelty performance. (A) Percentage time spent with (familiar) stranger mice in combined male and female, (B) male and (C) female C57BL/6J and CNTNAP2$^{-/-}$ mice. C57BL/6J mice treated with chondroitinase (n=8 males; n=6 females) and penicillinase (n=6 males; n=6 females). CNTNAP2$^{-/-}$ mice treated with chondroitinase (n=8 males; n=7 females) and penicillinase (n=4 males; n=6 females). No change in time spent interacting with the familiar mouse (previous stranger mouse) after the ChABC treatment during social novelty testing. Data expressed as mean ± SEM (p<0.05) (p<0.1). Mixed ANOVA and Fisher LSD post hoc comparisons were performed with 3-way interactions (A: $F_{1, 49.7} = 0.01$, p = 0.9289; B: $F_{1, 25} = 1.24$, p = 0.2767; C: $F_{1, 22.9} = 2.03$, p = 0.1675).

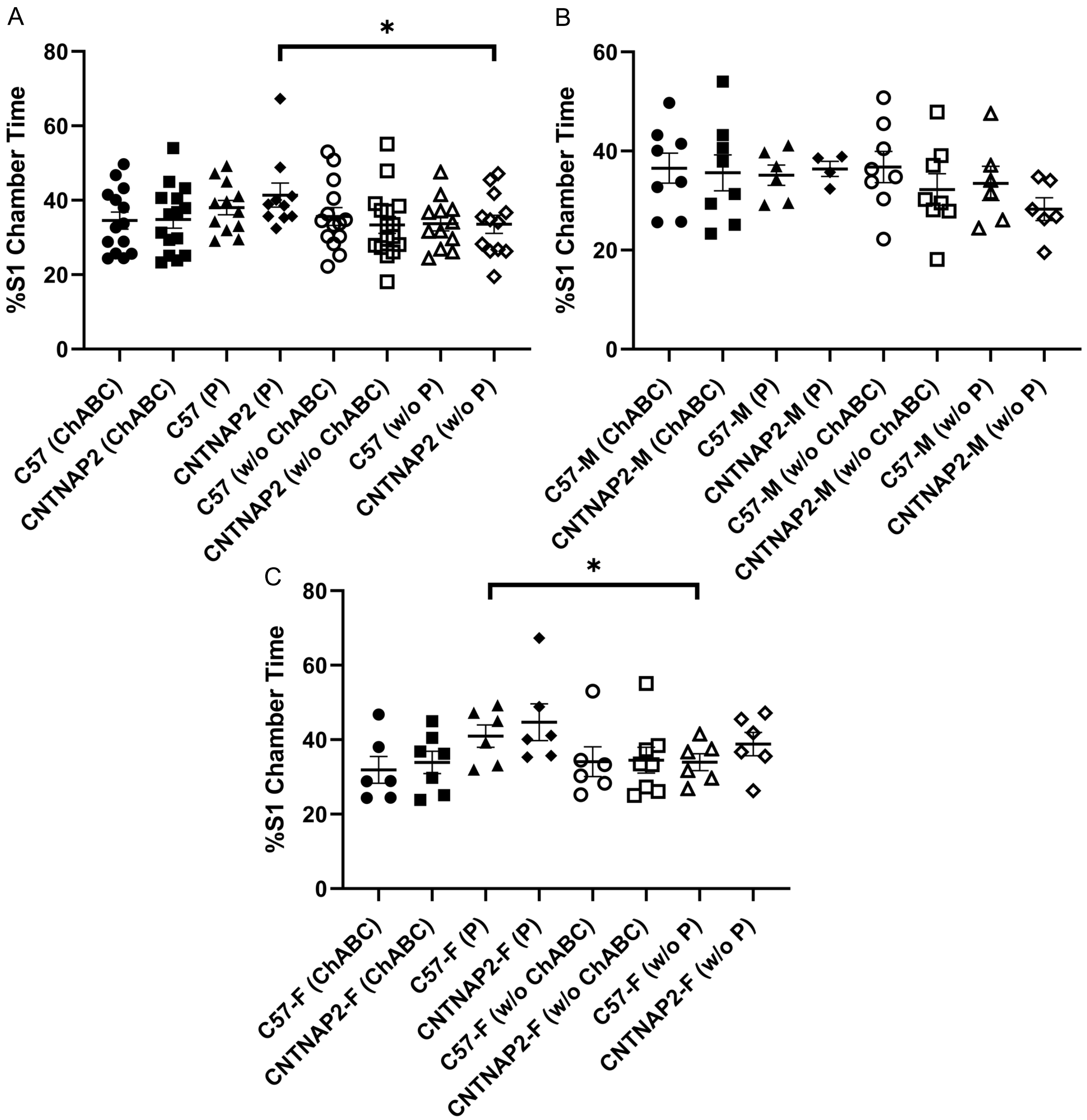

Figure 28. Three-chamber social novelty performance (A) percentage time spent in chamber housing the (familiar) stranger mice in combined male and female, (B) male and (C) female C57BL/6J and CNTNAP2$^{-/-}$ mice. C57BL/6J mice treated with chondroitinase (n=8 males; n=6 females) and penicillinase (n=6 males; n=6 females). CNTNAP2$^{-/-}$ mice treated with chondroitinase (n=8 males; n=7 females) and penicillinase (n=4 males; n=6 females). Data expressed as mean ± SEM (p<0.05) (p<0.1). Mixed ANOVA and Fisher LSD post hoc comparisons were performed with 3-way interactions (A: $F_{1, 48.8} = 0.04$, $p = 0.8461$; B: $F_{1, 24.9} = 0.08$, $p = 0.7723$; C: $F_{1, 21.2} = 0.10$, $p = 0.7594$)

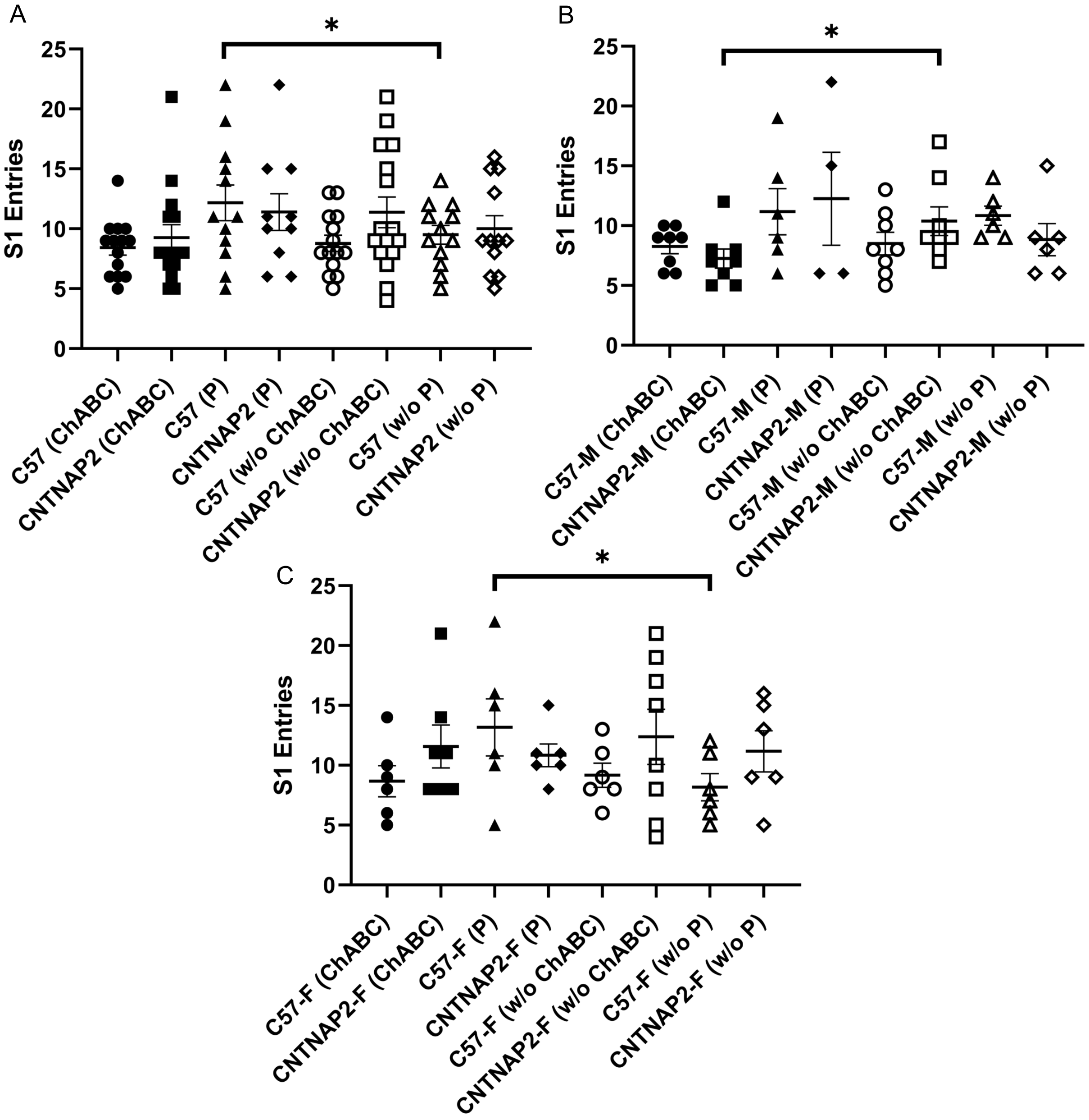

Figure 29. Three-chamber social novelty performance (A) number of entries in chamber housing the (familiar) stranger mice in combined male and female, (B) male and (C) female C57BL/6J and CNTNAP2$^{-/-}$ mice. C57BL/6J mice treated with chondroitinase (n=8 males; n=6 females) and penicillinase (n=6 males; n=6 females). CNTNAP2$^{-/-}$ mice treated with chondroitinase (n=8 males; n=7 females) and penicillinase (n=4 males; n=6 females). Data expressed as mean ± SEM (p<0.05) (p<0.1). Mixed ANOVA and Fisher LSD post hoc comparisons were performed with 3-way interactions (A: $F_{1, 48.7} = 0.02$, p = 0.8461; B: $F_{1, 23.6} = 4.42$, p = 0.0465; C: $F_{1, 22.2} = 1.81$, p = 0.1922).

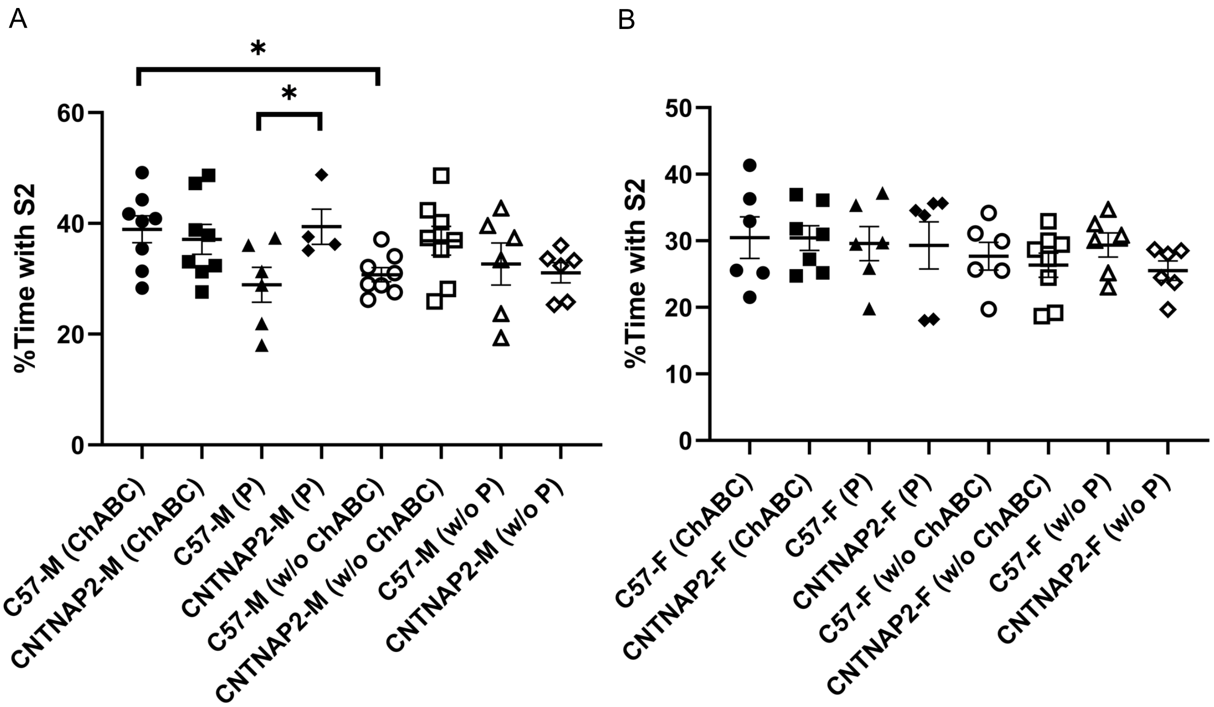

Figure 30. Three-chamber social novelty performance (A) percentage time spent with stranger mice in male and, (B) female C57BL/6J and CNTNAP2$^{-/-}$ mice. C57BL/6J mice treated with chondroitinase (n=8 males; n=6 females) and penicillinase (n=6 males; n=6 females). CNTNAP2$^{-/-}$ mice treated with chondroitinase (n=8 males; n=7 females) and penicillinase (n=4 males; n=6 females). Data expressed as mean ± SEM ($p<0.05$) ($p<0.1$). Mixed ANOVA and Fisher LSD post hoc comparisons were performed with 3-way interactions (A: $F_{1, 24} = 7.39$, p = 0.012; B: $F_{1, 21.4} = 0.10$, p = 0.7537).

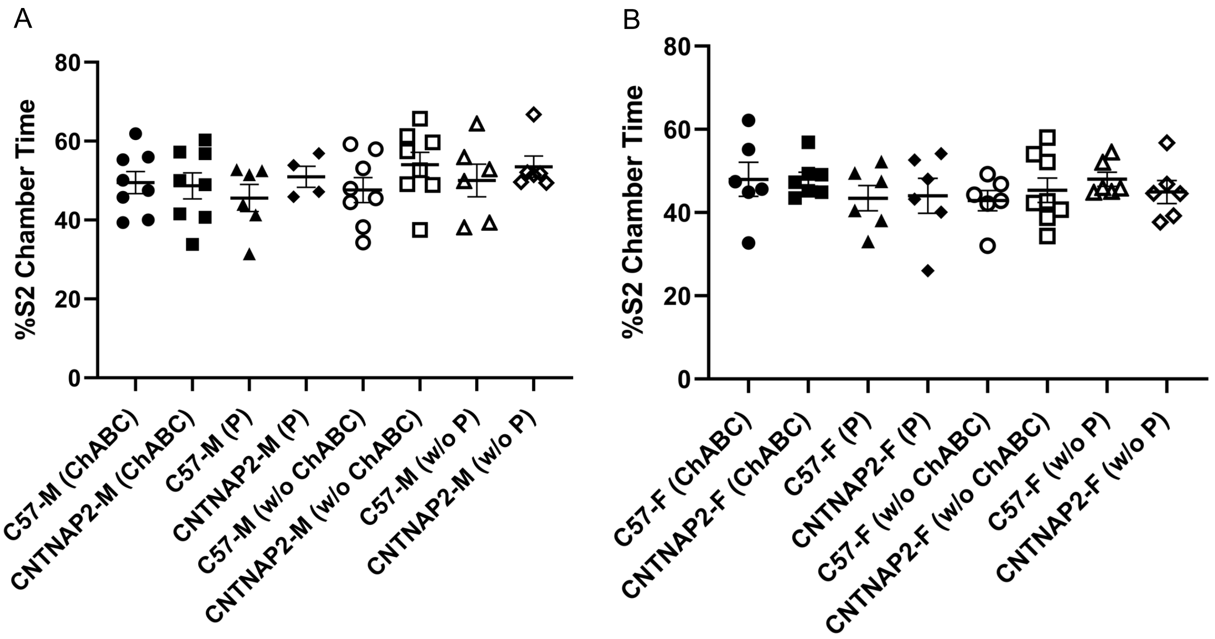

Figure 31. Three-chamber social novelty performance (A) percentage time spent in chamber housing stranger mice in male and, (B) female C57BL/6J and CNTNAP2$^{-/-}$ mice. C57BL/6J mice treated with chondroitinase (n=8 males; n=6 females) and penicillinase (n=6 males; n=6 females). CNTNAP2$^{-/-}$ mice treated with chondroitinase (n=8 males; n=7 females) and penicillinase (n=4 males; n=6 females). Data expressed as mean ± SEM (p<0.05) (p<0.1). Mixed ANOVA and Fisher LSD post hoc comparisons were performed with 3-way interactions (A: $F_{1, 25} = 0.87$, p = 0.3572; B: $F_{1, 21.1} = 0.82$, p = 0.3745).

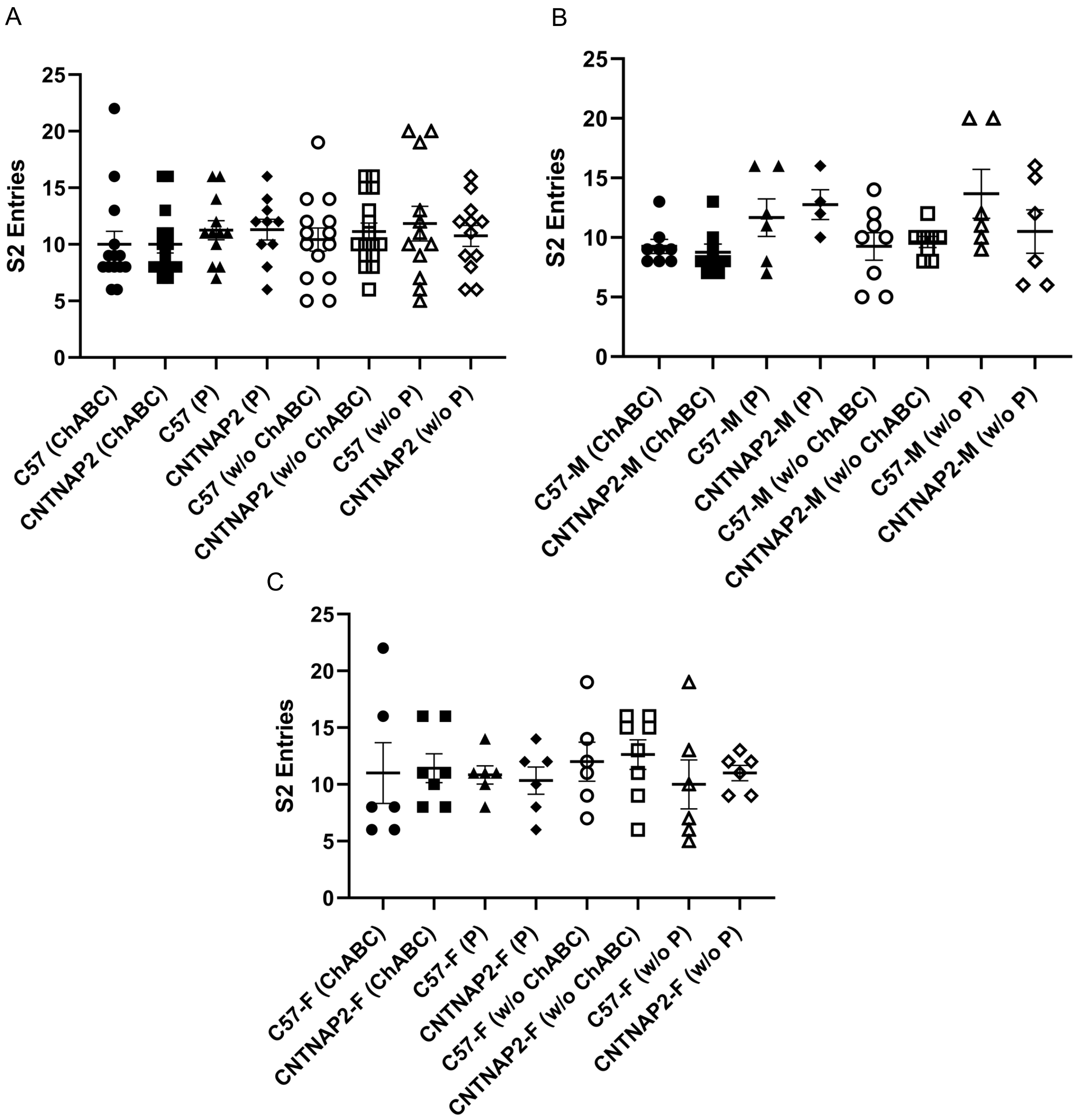

Figure 32. Three-chamber social novelty performance (A) number of entries in chamber housing the stranger mice in combined male and female, (B) male and (C) female C57BL/6J and CNTNAP2$^{-/-}$ mice. C57BL/6J mice treated with chondroitinase (n=8 males; n=6 females) and penicillinase (n=6 males; n=6 females). CNTNAP2$^{-/-}$ mice treated with chondroitinase (n=8 males; n=7 females) and penicillinase (n=4 males; n=6 females). Data expressed as mean ± SEM (p<0.05) (p<0.1). Mixed ANOVA and Fisher LSD post hoc comparisons were performed with 3-way interactions (A: $F_{1, 49.6} = 0.61$, p = 0.4370; B: $F_{1, 22.8} = 2.84$, p = 0.1054; C: $F_{1, 23} = 0.16$, p = 0.6935).

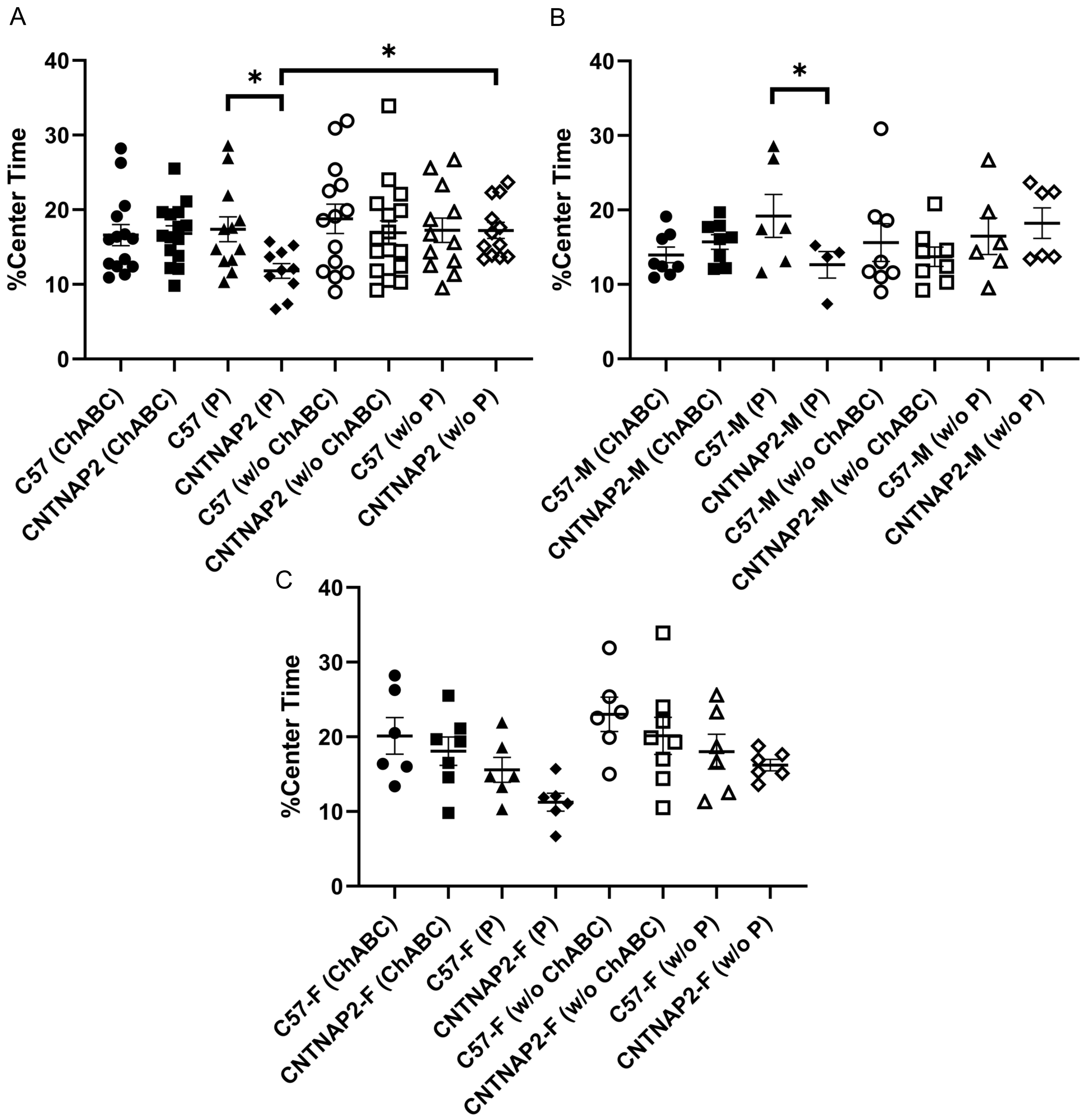

Figure 33. Three-chamber social novelty performance (A) percentage time spent in the center chamber in combined male and female, (B) male and (C) female C57BL/6J and CNTNAP2$^{-/-}$ mice. C57BL/6J mice treated with chondroitinase (n=8 males; n=6 females) and penicillinase (n=6 males; n=6 females). CNTNAP2$^{-/-}$ mice treated with chondroitinase (n=8 males; n=7 females) and penicillinase (n=4 males; n=6 females). Data expressed as mean ± SEM (p<0.05) (p<0.1). Mixed ANOVA and Fisher LSD post hoc comparisons were performed with 3-way interactions (A: $F_{1, 50.1} = 3.80$, $p = 0.0569$; B: $F_{1, 24.3} = 3.73$, $p = 0.0652$; C: $F_{1, 24.3} = 0.44$, $p = 0.5097$).

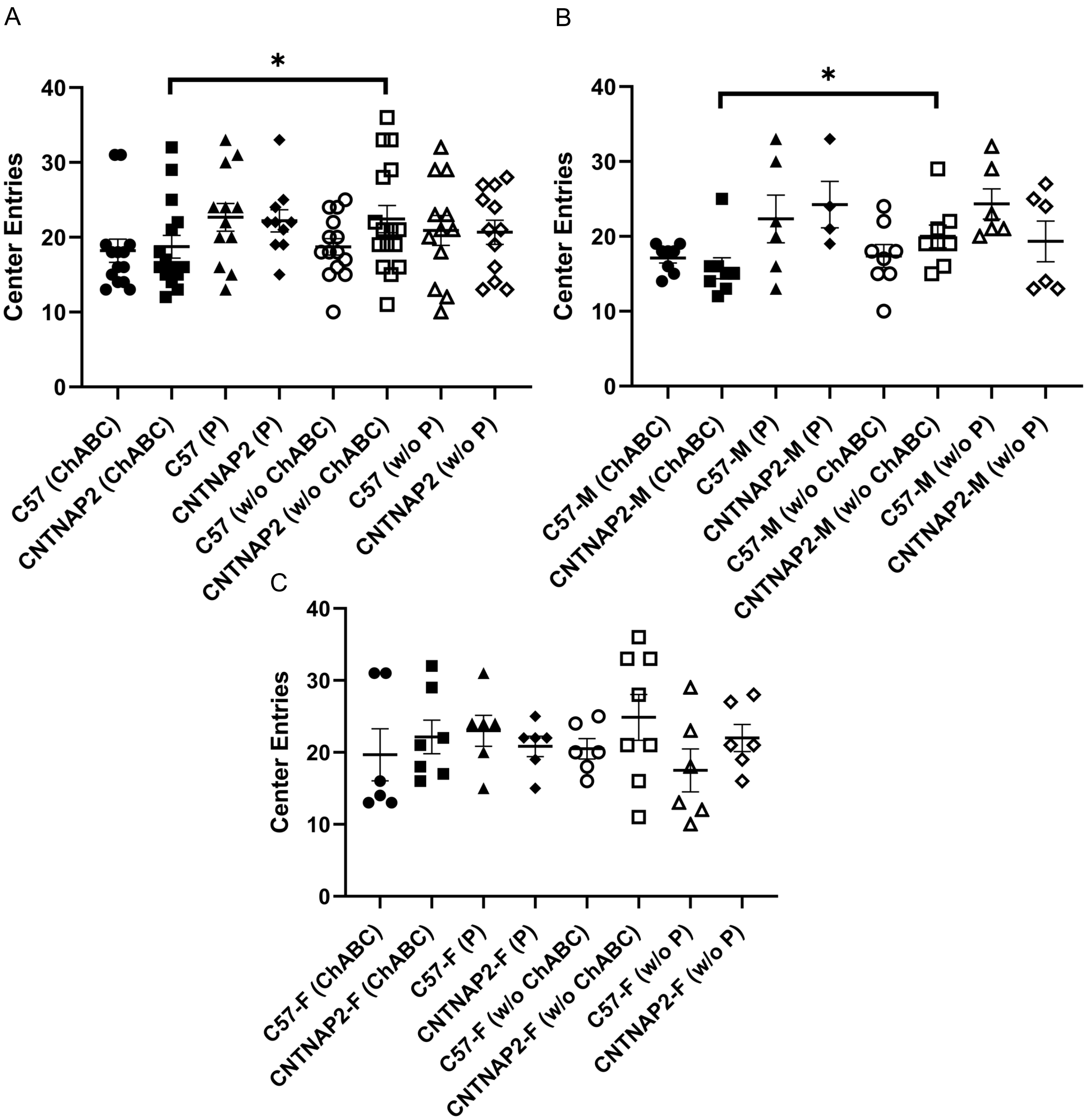

Figure 34. Three-chamber social novelty performance (A) number of entries in the center chamber in combined male and female, (B) male and (C) female C57BL/6J and CNTNAP2$^{-/-}$ mice. C57BL/6J mice treated with chondroitinase (n=8 males; n=6 females) and penicillinase (n=6 males; n=6 females). CNTNAP2$^{-/-}$ mice treated with chondroitinase (n=8 males; n=7 females) and penicillinase (n=4 males; n=6 females). Data expressed as mean ± SEM ($p<0.05$). Mixed ANOVA and Fisher LSD post hoc comparisons were performed with 3-way interactions (A: $F_{1, 49.1} = 0.55$, $p = 0.4609$; B: $F_{1, 23.2} = 6.47$, $p = 0.0181$; C: $F_{1, 22.7} = 1.18$, $p = 0.2883$).

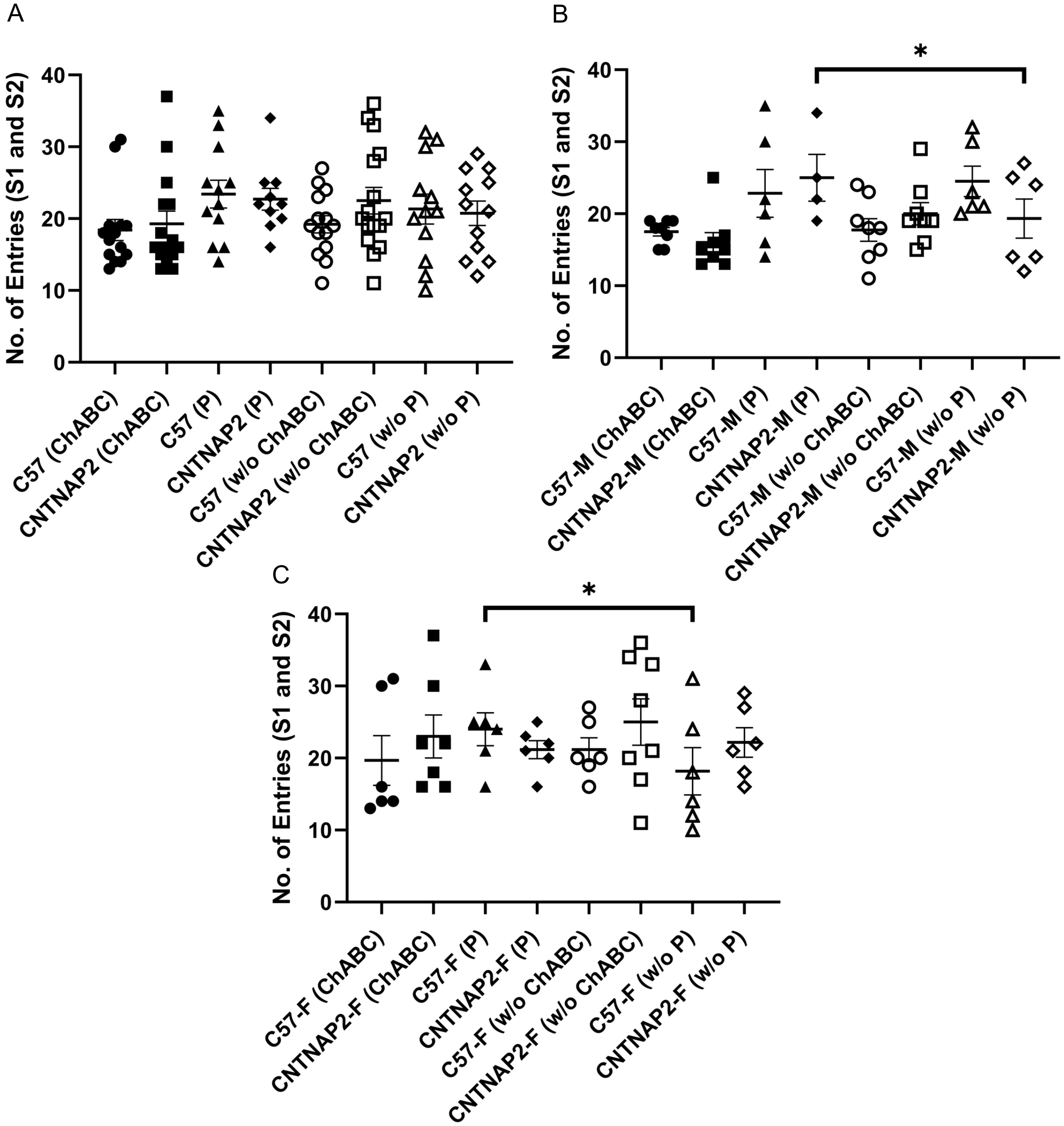

Figure 35. Three-chamber sociability performance (A) total number of entries in the object and stranger mice chambers in combined male and female, (B) male and (C) female C57BL/6J and CNTNAP2$^{-/-}$ mice. C57BL/6J mice treated with chondroitinase (n=8 males; n=6 females) and penicillinase (n=6 males; n=6 females). CNTNAP2$^{-/-}$ mice treated with chondroitinase (n=8 males; n=7 females) and penicillinase (n=4 males; n=6 females). Data expressed as mean ± SEM ($p<0.05$). Mixed ANOVA and Fisher LSD post hoc comparisons were performed with 3-way interactions (A: $F_{1,49.1} = 0.29$, $p = 0.5896$; B: $F_{1,23.3} = 5.91$, $p = 0.0231$; C: $F_{1,22.6} = 1.83$, $p = 0.1890$).

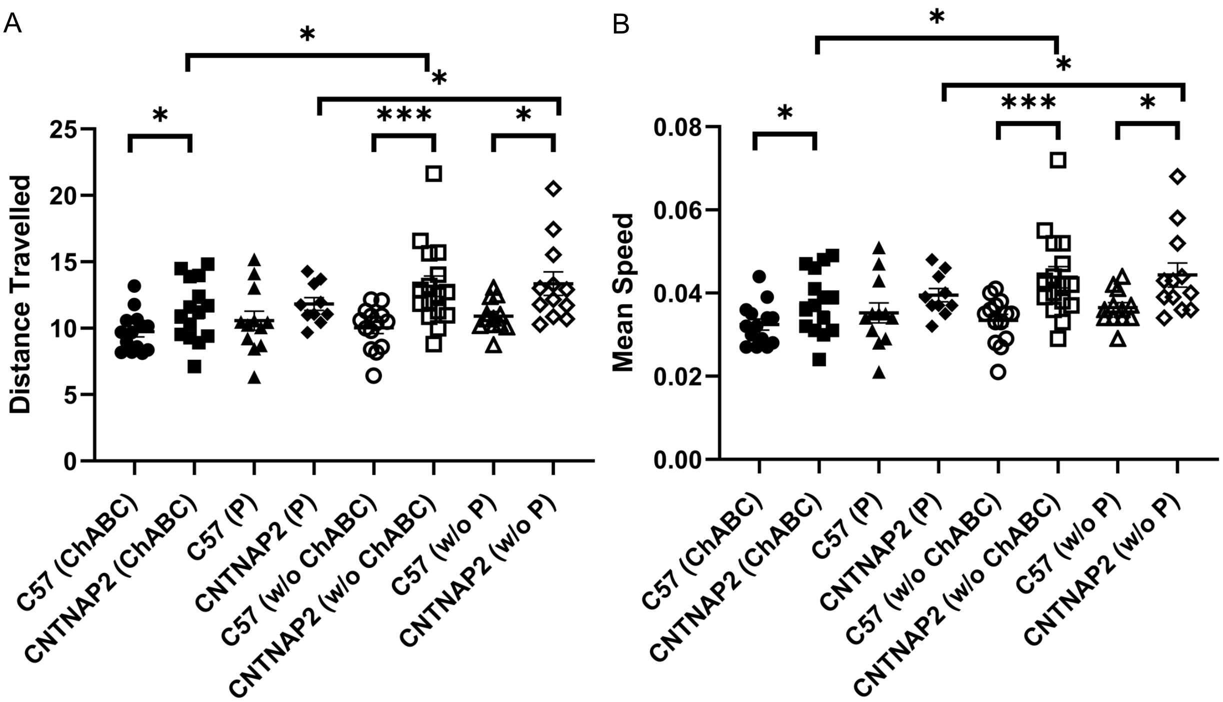

Figure 36. Three-chamber sociability performance (A) total distance travelled and (B) mean speed in C57BL/6J and CNTNAP2$^{-/-}$ mice. C57BL/6J mice treated with chondroitinase (n=14) and penicillinase (n=12). CNTNAP2$^{-/-}$ mice treated with chondroitinase (n=15) and penicillinase (n=10). Data expressed as mean ± SEM ($p<0.05$). Mixed ANOVA and Fisher LSD post hoc comparisons were performed with 3-way interactions (A: $F_{1, 42.2} = 0.0002$, p = 0.9901; B: $F_{1, 42.2} = 0.003$, p = 0.9587).

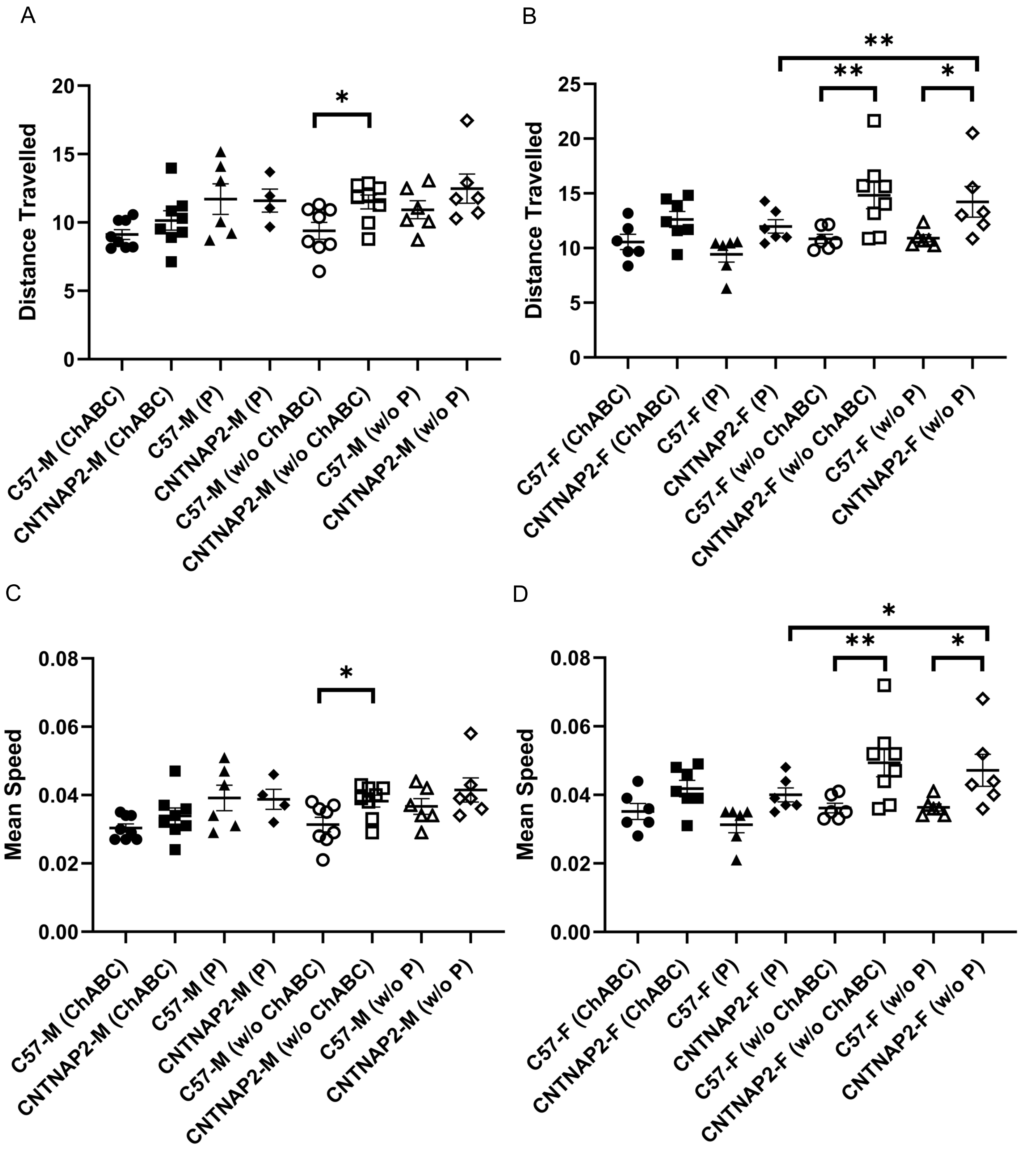

Figure 37. Three-chamber sociability performance. Total distance travelled and mean speed in (A, C) male and (B, D) female C57BL/6J and CNTNAP2$^{-/-}$ mice. C57BL/6J mice treated with chondroitinase (n=8 males; n=6 females) and penicillinase (n=6 males; n=6 females). CNTNAP2$^{-/-}$ mice treated with chondroitinase (n=8 males; n=7 females) and penicillinase (n=4 males; n=6 females). Data expressed as mean ± SEM (p<0.05) (p<0.1). Mixed ANOVA and Fisher LSD post hoc comparisons were performed with 3-way interactions (A: $F_{1, 23.0}$ = 0.23, p = 0.6362; B: $F_{1, 16.5}$ = 0.001, p = 0.9721; C: $F_{1, 23.1}$= 0.21, p = 0.6547; D: $F_{1, 16.5}$= 0.004, p = 0.9528)
