# Supplementary material for: Behavioral regulation by perineuronal nets in the prefrontal cortex of the CNTNAP2 mouse model of autism spectrum disorder
Source: Front Behav Neurosci. 2023 Mar 14;17:1114789. doi: 10.3389/fnbeh.2023.1114789 (PMC10043266; doi:10.3389/fnbeh.2023.1114789)
Supplement: Supplementary file 2 [file Data_Sheet_2.PDF]

use log (x+1) to fullfill normality assumption **FIG. 2E**

Source	Nparm	DFNum	DFDen	F Ratio	Prob > F
mouse	1	1	40	0.530824	0.4705
trt	1	1	40	30.88772	<.0001
mouse*trt	1	1	40	0.295069	0.59

mouse	trt	-mouse	-trt	Difference	Std Error	t Ratio	Prob>|t|	Lower 95%	Upper 95%
C57	ChABC	C57	P	-1.01602	0.291943	-3.48	0.0012	-1.60606	-0.42599
C57	ChABC	CNTNAP2	ChABC	-0.03756	0.27373	-0.14	0.8915	-0.59079	0.51567
C57	ChABC	CNTNAP2	P	-1.27371	0.291943	-4.36	<.0001	-1.86375	-0.68367
C57	P	CNTNAP2	ChABC	0.97846	0.281045	3.48	0.0012	0.41045	1.54648
C57	P	CNTNAP2	P	-0.25769	0.298813	-0.86	0.3936	-0.86161	0.34624
CNTNAP2	ChABC	CNTNAP2	P	-1.23615	0.281045	-4.4	<.0001	-1.80416	-0.66814

mouse	trt			Least Squares Mean (log transformed)
CNTNAP2	P	A		1.702889
C57	P	A		1.445203
CNTNAP2	ChABC		B	0.466739
C57	ChABC		B	0.429179

raw data **FIG. 2F**

Source	Nparm	DFNum	DFDen	F Ratio	Prob > F
mouse	1	1	40	0.374119	0.5442
trt	1	1	40	34.11959	<.0001
mouse*trt	1	1	40	2.210845	0.1449

mouse	trt	-mouse	-trt	Difference	Std Error	t Ratio	Prob>|t|	Lower 95%	Upper 95%
CNTNAP2	ChABC	CNTNAP2	P	-23.3305	4.416017	-5.28	<.0001	-32.2556	-14.4054
C57	ChABC	CNTNAP2	P	-20.544	4.587244	-4.48	<.0001	-29.8152	-11.2728
C57	P	CNTNAP2	ChABC	16.6494	4.416017	3.77	0.0005	7.7242	25.5745
C57	ChABC	C57	P	-13.8628	4.587244	-3.02	0.0044	-23.134	-4.5917
C57	P	CNTNAP2	P	-6.6812	4.695194	-1.42	0.1625	-16.1705	2.8082
C57	ChABC	CNTNAP2	ChABC	2.7865	4.301066	0.65	0.5208	-5.9063	11.4793

mouse	trt			Least Squares Mean
CNTNAP2	P	A		30.52569
C57	P	A		23.84454
C57	ChABC		B	9.981691
CNTNAP2	ChABC		B	7.195187

use log (x+1) to fullfill normality assumption **FIG. 2G**

Source	Nparm	DFNum	DFDen	F Ratio	Prob > F
mouse	1	1	40	0.048649	0.8266
trt	1	1	40	0.63353	0.4308
mouse*trt	1	1	40	3.907139	0.055

mouse	trt	-mouse	-trt	Difference	Std Error	t Ratio	Prob>|t|	Lower 95%	Upper 95%
C57	ChABC	C57	P	-0.70298	0.36532	-1.92	0.0615	-1.44132	0.035362
C57	ChABC	CNTNAP2	ChABC	-0.44525	0.342529	-1.3	0.2011	-1.13752	0.247032
C57	ChABC	CNTNAP2	P	-0.14589	0.36532	-0.4	0.6918	-0.88422	0.592454
C57	P	CNTNAP2	ChABC	0.257731	0.351684	0.73	0.4679	-0.45305	0.96851
C57	P	CNTNAP2	P	0.557092	0.373917	1.49	0.1441	-0.19862	1.312806
CNTNAP2	ChABC	CNTNAP2	P	0.299361	0.351684	0.85	0.3997	-0.41142	1.01014

mouse	trt		Least Squares Mean (log transformed)
C57	P	A	3.371429
CNTNAP2	ChABC	A	3.113698
CNTNAP2	P	A	2.814337
C57	ChABC	A	2.668452

FIG. 3A

Source	Nparm	DFNum	DFDen	F Ratio	Prob > F
trt	1	1	50.6	0.195103	0.6606
mouse	1	1	50.6	1.404834	0.2415
trt*mouse	1	1	50.6	0.185856	0.6682
time	1	1	49.3	0.038599	0.8451
trt*time	1	1	49.3	0.109724	0.7419
mouse*time	1	1	49.3	0.913743	0.3438
trt*mouse*tim	1	1	49.3	0.020475	0.8868

trt	mouse	time	-trt	-mouse	-time	Difference	Std Error	t Ratio	Prob>|t|	Lower 95%	Upper 95%
ChABC	C57	after	ChABC	C57	before	1.44357	2.049096	0.7	0.4845	-2.67643	5.563574
ChABC	C57	after	ChABC	CNTNAP2	after	-1.73567	2.587812	-0.67	0.5042	-6.88033	3.408987
ChABC	C57	after	ChABC	CNTNAP2	before	-2.04268	2.553401	-0.8	0.426	-7.11989	3.034537
ChABC	C57	after	P	C57	after	-1.20726	2.744823	-0.44	0.6612	-6.6651	4.250581
ChABC	C57	after	P	C57	before	-0.16893	2.744823	-0.06	0.9511	-5.62677	5.288914
ChABC	C57	after	P	CNTNAP2	after	-1.24198	2.870839	-0.43	0.6663	-6.94706	4.463102
ChABC	C57	after	P	CNTNAP2	before	-2.5706	2.744823	-0.94	0.3517	-8.02844	2.887247
ChABC	C57	before	ChABC	CNTNAP2	after	-3.17924	2.587812	-1.23	0.2226	-8.3239	1.965416
ChABC	C57	before	ChABC	CNTNAP2	before	-3.48625	2.553401	-1.37	0.1758	-8.56347	1.590965
ChABC	C57	before	P	C57	after	-2.65083	2.744823	-0.97	0.3369	-8.10868	2.807009
ChABC	C57	before	P	C57	before	-1.6125	2.744823	-0.59	0.5585	-7.07034	3.845343
ChABC	C57	before	P	CNTNAP2	after	-2.68555	2.870839	-0.94	0.3521	-8.39064	3.019531
ChABC	C57	before	P	CNTNAP2	before	-4.01417	2.744823	-1.46	0.1473	-9.47201	1.443676
ChABC	CNTNAP2	after	ChABC	CNTNAP2	before	-0.30701	1.962361	-0.16	0.8763	-4.24992	3.635905
ChABC	CNTNAP2	after	P	C57	after	0.52841	2.697464	0.2	0.8452	-4.83432	5.891136
ChABC	CNTNAP2	after	P	C57	before	1.56674	2.697464	0.58	0.5629	-3.79599	6.92947
ChABC	CNTNAP2	after	P	CNTNAP2	after	0.49369	2.825593	0.17	0.8617	-5.12064	6.108017
ChABC	CNTNAP2	after	P	CNTNAP2	before	-0.83493	2.697464	-0.31	0.7577	-6.19765	4.527803
ChABC	CNTNAP2	before	P	C57	after	0.83542	2.66447	0.31	0.7546	-4.46265	6.133483

ChABC	CNTNAP2	before	P	C57	before	1.87375	2.66447	0.7	0.4838	-3.42432	7.171816
ChABC	CNTNAP2	before	P	CNTNAP2	after	0.8007	2.794111	0.29	0.7751	-4.75175	6.353141
ChABC	CNTNAP2	before	P	CNTNAP2	before	-0.52792	2.66447	-0.2	0.8434	-5.82598	4.770149
P	C57	after	P	C57	before	1.03833	2.213277	0.47	0.6411	-3.41178	5.488444
P	C57	after	P	CNTNAP2	after	-0.03472	2.97006	-0.01	0.9907	-5.93719	5.867749
P	C57	after	P	CNTNAP2	before	-1.36333	2.848438	-0.48	0.6334	-7.0272	4.300538
P	C57	before	P	CNTNAP2	after	-1.07305	2.97006	-0.36	0.7187	-6.97552	4.829416
P	C57	before	P	CNTNAP2	before	-2.40167	2.848438	-0.84	0.4015	-8.06554	3.262204
P	CNTNAP2	after	P	CNTNAP2	before	-1.32861	2.367753	-0.56	0.5771	-6.08059	3.423358

trt	mouse	time		Least Squares Mean
P	CNTNAP2	before	A	10.90417
ChABC	CNTNAP2	before	A	10.37625
ChABC	CNTNAP2	after	A	10.06924
P	CNTNAP2	after	A	9.575552
P	C57	after	A	9.540833
P	C57	before	A	8.5025
ChABC	C57	after	A	8.333571
ChABC	C57	before	A	6.89

FIG. 3B

Source	Nparm	DFNum	DFDen	F Ratio	Prob > F
trt	1	1	49.4	0.03989	0.8425
mouse	1	1	49.4	9.336409	0.0036
trt*mouse	1	1	49.4	0.000155	0.9901
time	1	1	48.9	4.345206	0.0424
trt*time	1	1	48.9	0.925951	0.3407
mouse*tim	1	1	48.9	6.51E-05	0.9936
trt*mouse*	1	1	48.9	0.084715	0.7722

trt	mouse	time	-trt	-mouse	-time	Difference	Std Error	t Ratio	Prob>|t|	Lower 95%	Upper 95%
**P**	C57	after	P	CNTNAP2	before	-3	1.023246	-2.93	0.0042	-5.03157	-0.96843
ChABC	C57	after	P	CNTNAP2	before	-2.80952	0.986025	-2.85	0.0054	-4.7672	-0.85185
ChABC	CNTNAP2	before	P	C57	after	2.33333	0.957159	2.44	0.0167	0.43297	4.2337
ChABC	C57	after	ChABC	CNTNAP2	before	-2.14286	0.91726	-2.34	0.0216	-3.964	-0.32171
ChABC	C57	before	P	CNTNAP2	before	-2.16667	0.986025	-2.2	0.0304	-4.12434	-0.20899
ChABC	CNTNAP2	after	P	C57	after	1.96105	0.9709	2.02	0.0462	0.03348	3.88862
ChABC	C57	after	ChABC	CNTNAP2	after	-1.77057	0.931589	-1.9	**0.0604**	-3.62009	0.07894
P	C57	before	P	CNTNAP2	before	-1.75	1.023246	-1.71	0.0905	-3.78157	0.28157
ChABC	C57	before	ChABC	CNTNAP2	before	-1.5	0.91726	-1.64	0.1053	-3.32114	0.32114
P	CNTNAP2	after	P	CNTNAP2	before	-1.50598	0.988222	-1.52	0.1336	-3.48929	0.47732
P	C57	after	P	CNTNAP2	after	-1.49402	1.07378	-1.39	0.1674	-3.62567	0.63764
P	C57	after	P	C57	before	-1.25	0.933066	-1.34	0.1868	-3.12678	0.62678
ChABC	C57	after	P	CNTNAP2	after	-1.30354	1.038371	-1.26	0.2124	-3.36489	0.75781
ChABC	C57	before	ChABC	CNTNAP2	after	-1.12772	0.931589	-1.21	0.2291	-2.97724	0.7218
ChABC	CNTNAP2	before	P	C57	before	1.08333	0.957159	1.13	0.2606	-0.81703	2.9837
ChABC	C57	after	P	C57	before	-1.05952	0.986025	-1.07	0.2853	-3.0172	0.89815
ChABC	CNTNAP2	after	P	CNTNAP2	before	-1.03895	0.9709	-1.07	0.2873	-2.96652	0.88862
ChABC	C57	before	P	C57	after	0.83333	0.986025	0.85	0.4002	-1.12434	2.79101
ChABC	CNTNAP2	before	P	CNTNAP2	after	0.83932	1.011002	0.83	0.4085	-1.16769	2.84632
ChABC	C57	after	ChABC	C57	before	-0.64286	0.863851	-0.74	0.4605	-2.38042	1.0947
ChABC	CNTNAP2	after	P	C57	before	0.71105	0.9709	0.73	0.4658	-1.21652	2.63862
ChABC	CNTNAP2	before	P	CNTNAP2	before	-0.66667	0.957159	-0.7	0.4878	-2.56703	1.2337
ChABC	C57	before	P	CNTNAP2	after	-0.66068	1.038371	-0.64	0.5261	-2.72203	1.40066

ChABC	CNTNAP2	after	P	CNTNAP2	after	0.46703	1.02402	0.46	0.6494	-1.56576	2.49983
ChABC	CNTNAP2	after	ChABC	CNTNAP2	before	-0.37228	0.824289	-0.45	0.6535	-2.02891	1.28434
ChABC	C57	before	P	C57	before	-0.41667	0.986025	-0.42	0.6736	-2.37434	1.54101
P	C57	before	P	CNTNAP2	after	-0.24402	1.07378	-0.23	0.8207	-2.37567	1.88764
ChABC	C57	after	P	C57	after	0.19048	0.986025	0.19	0.8472	-1.7672	2.14815

trt	mouse	time					Least Squares Mean
P	CNTNAP2	before	A				5.166667
ChABC	CNTNAP2	before	A	B			4.5
ChABC	CNTNAP2	after	A	B	C		4.127717
P	CNTNAP2	after	A	B	C	D	3.660684
P	C57	before	A	B	C	D	3.416667
ChABC	C57	before		B	C	D	3
ChABC	C57	after			C	D	2.357143
P	C57	after				D	2.166667

FIG. 3C

Source	Nparm	DFNum	DFDen	F Ratio	Prob > F
trt	1	1	49.9	0.570078	0.4538
mouse	1	1	49.9	15.88222	0.0002
trt*mouse	1	1	49.9	0.294962	0.5895
time	1	1	50.2	32.19243	<.0001
trt*time	1	1	50.2	3.178823	0.0806
mouse*tim	1	1	50.2	0.0248	0.8755
trt*mouse*	1	1	50.2	0.669621	0.4171

trt	mouse	time	-trt	-mouse	-time	Difference	Std Error	t Ratio	Prob>|t|	Lower 95%	Upper 95%
P	C57	after	P	CNTNAP2	before	-33.9661	6.350719	-5.35	<.0001	-46.5718	-21.3604
ChABC	CNTNAP2	before	P	C57	after	30.2554	5.940554	5.09	<.0001	18.4639	42.047
ChABC	C57	after	P	CNTNAP2	before	-29.4343	6.119706	-4.81	<.0001	-41.5815	-17.2872
ChABC	C57	after	ChABC	CNTNAP2	before	-25.7237	5.692921	-4.52	<.0001	-37.0237	-14.4237
P	C57	after	P	C57	before	-26.004	6.638943	-3.92	0.0003	-39.3487	-12.6593
ChABC	C57	after	P	C57	before	-21.4723	6.119706	-3.51	0.0007	-33.6194	-9.3251
ChABC	C57	before	P	CNTNAP2	before	-20.096	6.119706	-3.28	0.0014	-32.2431	-7.9488
ChABC	CNTNAP2	after	P	CNTNAP2	before	-19.3007	6.027507	-3.2	0.0018	-31.2647	-7.3367
P	CNTNAP2	after	P	CNTNAP2	before	-21.7702	6.945371	-3.13	0.0028	-35.7	-7.8405
ChABC	C57	before	ChABC	CNTNAP2	before	-16.3853	5.692921	-2.88	0.0049	-27.6853	-5.0853
ChABC	CNTNAP2	before	P	CNTNAP2	after	18.0596	6.281146	2.88	0.005	5.5924	30.5267
ChABC	CNTNAP2	after	ChABC	CNTNAP2	before	-15.5901	5.839292	-2.67	0.0102	-27.318	-3.8622
ChABC	CNTNAP2	after	P	C57	after	14.6654	6.027507	2.43	0.0168	2.7013	26.6294
ChABC	C57	before	P	C57	after	13.8701	6.119706	2.27	0.0257	1.7229	26.0173
P	C57	before	P	CNTNAP2	after	13.8082	6.670401	2.07	0.0411	0.5684	27.048
ChABC	C57	before	P	C57	before	-12.1339	6.119706	-1.98	0.0502	-24.2811	0.0133
ChABC	CNTNAP2	after	P	C57	before	-11.3386	6.027507	-1.88	0.063	-23.3027	0.6254
P	C57	after	P	CNTNAP2	after	-12.1958	6.670401	-1.83	0.0706	-25.4356	1.044
ChABC	C57	after	ChABC	CNTNAP2	after	-10.1336	5.783598	-1.75	0.0829	-21.6135	1.3462
ChABC	C57	after	ChABC	C57	before	-9.3384	6.146467	-1.52	0.1352	-21.6932	3.0164
P	C57	before	P	CNTNAP2	before	-7.9621	6.350719	-1.25	0.213	-20.5678	4.6437

ChABC	C57	after	P	CNTNAP2	after	-7.6641	6.450846	-1.19	0.2377	-20.4681	5.1399
ChABC	C57	after	P	C57	after	4.5317	6.119706	0.74	0.4608	-7.6154	16.6789
ChABC	CNTNAP2	before	P	C57	before	4.2514	5.940554	0.72	0.4759	-7.5402	16.043
ChABC	CNTNAP2	before	P	CNTNAP2	before	-3.7106	5.940554	-0.62	0.5337	-15.5022	8.0809
ChABC	CNTNAP2	after	P	CNTNAP2	after	2.4695	6.363446	0.39	0.6988	-10.1609	15.0999
ChABC	C57	before	P	CNTNAP2	after	1.6743	6.450846	0.26	0.7958	-11.1297	14.4783
ChABC	C57	before	ChABC	CNTNAP2	after	-0.7953	5.783598	-0.14	0.8909	-12.2751	10.6846

trt	mouse	time					Least Squares Mean
P	CNTNAP2	before	A				63.8402
ChABC	CNTNAP2	before	A				60.12956
P	C57	before	A	B			55.87814
ChABC	CNTNAP2	after		B	C		44.5395
ChABC	C57	before		B	C		43.74424
P	CNTNAP2	after			C	D	42.06997
ChABC	C57	after			C	D	34.40587
P	C57	after				D	29.87413

FIG. 3D

Source	Nparm	DFNum	DFDen	F Ratio	Prob > F
trt	1	1	50.4	0.750394	0.3905
mouse	1	1	50.4	27.73661	<.0001
trt*mouse	1	1	50.4	0.967598	0.33
time	1	1	50.3	22.12141	<.0001
trt*time	1	1	50.3	3.243976	0.0777
mouse*tim	1	1	50.3	0.003161	0.9554
trt*mouse*	1	1	50.3	0.808731	0.3728

trt	mouse	time	-trt	-mouse	-time	Difference	Std Error	t Ratio	Prob>|t|	Lower 95%	Upper 95%
P	C57	after	P	CNTNAP2	before	-21.6667	4.173592	-5.19	<.0001	-29.9503	-13.3831
ChABC	CNTNAP2	before	P	C57	after	20.125	3.904038	5.15	<.0001	12.3764	27.8736
ChABC	C57	after	P	CNTNAP2	before	-20.131	4.021774	-5.01	<.0001	-28.1132	-12.1487
ChABC	C57	after	ChABC	CNTNAP2	before	-18.5893	3.741297	-4.97	<.0001	-26.0149	-11.1637
ChABC	C57	before	P	CNTNAP2	before	-16.0595	4.021774	-3.99	0.0001	-24.0418	-8.0773
ChABC	C57	before	ChABC	CNTNAP2	before	-14.5179	3.741297	-3.88	0.0002	-21.9434	-7.0923
P	C57	after	P	C57	before	-14.75	4.088216	-3.61	0.0007	-22.967	-6.533
ChABC	C57	after	P	C57	before	-13.2143	4.021774	-3.29	0.0014	-21.1965	-5.232
ChABC	CNTNAP2	after	P	C57	after	12.7197	3.961565	3.21	0.0018	4.857	20.5824
ChABC	C57	after	ChABC	CNTNAP2	after	-11.184	3.801288	-2.94	0.0041	-18.7286	-3.6394
P	CNTNAP2	after	P	CNTNAP2	before	-10.9714	4.303888	-2.55	0.0137	-19.603	-2.3399
P	C57	after	P	CNTNAP2	after	-10.6952	4.385067	-2.44	0.0165	-19.3985	-1.992
ChABC	CNTNAP2	before	P	CNTNAP2	after	9.4298	4.129341	2.28	0.0246	1.2341	17.6255
ChABC	C57	before	P	C57	before	-9.1429	4.021774	-2.27	0.0252	-17.1251	-1.1606
ChABC	CNTNAP2	after	P	CNTNAP2	before	-8.947	3.961565	-2.26	0.0262	-16.8097	-1.0842
ChABC	C57	after	P	CNTNAP2	after	-9.1595	4.240826	-2.16	0.0333	-17.5765	-0.7425
ChABC	CNTNAP2	after	ChABC	CNTNAP2	before	-7.4053	3.603834	-2.05	0.0451	-14.6429	-0.1677
ChABC	C57	before	ChABC	CNTNAP2	after	-7.1126	3.801288	-1.87	0.0644	-14.6572	0.4321
P	C57	before	P	CNTNAP2	before	-6.9167	4.173592	-1.66	0.1007	-15.2003	1.3669
ChABC	C57	before	P	C57	after	5.6071	4.021774	1.39	0.1664	-2.3751	13.5894
ChABC	CNTNAP2	before	P	C57	before	5.375	3.904038	1.38	0.1718	-2.3736	13.1236
ChABC	C57	before	P	CNTNAP2	after	-5.0881	4.240826	-1.2	0.2331	-13.505	3.3289

ChABC	C57	after	ChABC	C57	before	-4.0714	3.784953	-1.08	0.2874	-11.6789	3.536
P	C57	before	P	CNTNAP2	after	4.0548	4.385067	0.92	0.3574	-4.6485	12.758
ChABC	CNTNAP2	after	P	C57	before	-2.0303	3.961565	-0.51	0.6095	-9.893	5.8324
ChABC	CNTNAP2	after	P	CNTNAP2	after	2.0245	4.183771	0.48	0.6296	-6.2792	10.3282
ChABC	CNTNAP2	before	P	CNTNAP2	before	-1.5417	3.904038	-0.39	0.6938	-9.2902	6.2069
ChABC	C57	after	P	C57	after	1.5357	4.021774	0.38	0.7034	-6.4465	9.518

trt	mouse	time					Least Squares Mean
P	CNTNAP2	before	A				46.41667
ChABC	CNTNAP2	before	A				44.875
P	C57	before	A	B			39.5
ChABC	CNTNAP2	after		B	C		37.4697
P	CNTNAP2	after		B	C		35.44522
ChABC	C57	before			C	D	30.35714
ChABC	C57	after				D	26.28571
P	C57	after				D	24.75

FIG. 3E

Source	Nparm	DFNum	DFDen	F Ratio	Prob > F
trt	1	1	50.7	0.041662	0.8391
mouse	1	1	50.7	12.53604	0.0009
trt*mouse	1	1	50.7	3.246632	0.0775
time	1	1	50.7	1.370502	0.2472
trt*time	1	1	50.7	0.299201	0.5868
mouse*tim	1	1	50.7	0.919224	0.3422
trt*mouse*	1	1	50.7	0.024997	0.875

trt	mouse	time	-trt	-mouse	-time	Difference	Std Error	t Ratio	Prob>|t|	Lower 95%	Upper 95%
ChABC	C57	before	ChABC	CNTNAP2	before	-3.72791	1.118734	-3.33	0.0012	-5.94829	-1.50752
ChABC	C57	after	ChABC	CNTNAP2	before	-3.62206	1.118734	-3.24	0.0017	-5.84245	-1.40168
ChABC	C57	before	ChABC	CNTNAP2	after	-2.876	1.136689	-2.53	0.013	-5.13202	-0.61998
ChABC	CNTNAP2	before	P	C57	after	2.883	1.167397	2.47	0.0153	0.56603	5.19996
ChABC	C57	after	ChABC	CNTNAP2	after	-2.77015	1.136689	-2.44	0.0166	-5.02617	-0.51413
ChABC	C57	before	P	CNTNAP2	before	-2.9297	1.202603	-2.44	0.0167	-5.31655	-0.54286
ChABC	C57	after	P	CNTNAP2	before	-2.82386	1.202603	-2.35	0.0209	-5.2107	-0.43702
ChABC	CNTNAP2	before	P	C57	before	2.52365	1.167397	2.16	0.0331	0.20668	4.84062
ChABC	CNTNAP2	before	P	CNTNAP2	after	2.49355	1.234827	2.02	0.0462	0.04276	4.94435
ChABC	CNTNAP2	after	P	C57	after	2.03109	1.184614	1.71	0.0896	-0.32005	4.38223
P	C57	after	P	CNTNAP2	before	-2.0848	1.248	-1.67	0.098	-4.56174	0.39215
ChABC	CNTNAP2	after	P	C57	before	1.67174	1.184614	1.41	0.1614	-0.6794	4.02288
P	C57	before	P	CNTNAP2	before	-1.72545	1.248	-1.38	0.17	-4.20239	0.7515
ChABC	CNTNAP2	after	P	CNTNAP2	after	1.64165	1.251117	1.31	0.1926	-0.84148	4.12477
P	CNTNAP2	after	P	CNTNAP2	before	-1.69535	1.301003	-1.3	0.1981	-4.30415	0.91344
ChABC	C57	before	P	C57	before	-1.20426	1.202603	-1	0.3191	-3.5911	1.18259
ChABC	C57	before	P	CNTNAP2	after	-1.23435	1.268162	-0.97	0.3328	-3.75131	1.28261
ChABC	C57	after	P	C57	before	-1.09841	1.202603	-0.91	0.3633	-3.48525	1.28843
ChABC	C57	after	P	CNTNAP2	after	-1.12851	1.268162	-0.89	0.3757	-3.64546	1.38845
ChABC	CNTNAP2	after	ChABC	CNTNAP2	before	-0.85191	1.090168	-0.78	0.4382	-3.04093	1.33711
ChABC	C57	before	P	C57	after	-0.84491	1.202603	-0.7	0.484	-3.23175	1.54193
ChABC	CNTNAP2	before	P	CNTNAP2	before	0.7982	1.167397	0.68	0.4958	-1.51877	3.11517
ChABC	C57	after	P	C57	after	-0.73907	1.202603	-0.61	0.5403	-3.12591	1.64778

P	C57	after	P	CNTNAP2	after	-0.38944	1.311292	-0.3	0.7671	-2.992	2.21311
P	C57	after	P	C57	before	-0.35935	1.237185	-0.29	0.7727	-2.84555	2.12685
ChABC	C57	after	ChABC	C57	before	0.10584	1.145411	0.09	0.9268	-2.19593	2.40762
ChABC	CNTNAP2	after	P	CNTNAP2	before	-0.05371	1.184614	-0.05	0.9639	-2.40485	2.29743
P	C57	before	P	CNTNAP2	after	-0.03009	1.311292	-0.02	0.9817	-2.63265	2.57246

trt	mouse	time				Least Squares Mean
ChABC	CNTNAP2	before	A			9.919704
P	CNTNAP2	before	A	B		9.121503
ChABC	CNTNAP2	after	A	B		9.067795
P	CNTNAP2	after		B	C	7.42615
P	C57	before		B	C	7.396055
P	C57	after		B	C	7.036708
ChABC	C57	after			C	6.297642
ChABC	C57	before			C	6.191799

FIG. 3F

Source	Nparm	DFNum	DFDen	F Ratio	Prob > F
trt	1	1	50	0.585149	0.4479
mouse	1	1	50	24.9437	<.0001
trt*mouse	1	1	50	1.026735	0.3158
time	1	1	50.2	24.85139	<.0001
trt*time	1	1	50.2	3.583808	0.0641
mouse*tim	1	1	50.2	0.092822	0.7619
trt*mouse*	1	1	50.2	0.343417	0.5605

trt	mouse	time	-trt	-mouse	-time	Difference	Std Error	t Ratio	Prob>|t|	Lower 95%	Upper 95%
P	C57	after	P	CNTNAP2	before	38.2008	7.24752	5.27	<.0001	23.8163	52.5853
ChABC	C57	after	P	CNTNAP2	before	35.497	6.983885	5.08	<.0001	21.6358	49.3583
ChABC	CNTNAP2	before	P	C57	after	-34.4018	6.779434	-5.07	<.0001	-47.8573	-20.9463
ChABC	C57	after	ChABC	CNTNAP2	before	31.6981	6.496832	4.88	<.0001	18.8035	44.5927
ChABC	C57	before	P	CNTNAP2	before	27.6587	6.983885	3.96	0.0001	13.7975	41.52
ChABC	C57	before	ChABC	CNTNAP2	before	23.8598	6.496832	3.67	0.0004	10.9652	36.7544
P	C57	after	P	C57	before	25.493	7.371876	3.46	0.0011	10.6748	40.3113
ChABC	C57	after	P	C57	before	22.7893	6.983885	3.26	0.0015	8.928	36.6506
P	CNTNAP2	after	P	CNTNAP2	before	23.4894	7.73353	3.04	0.0037	7.9788	39.0001
ChABC	CNTNAP2	after	P	C57	after	-20.2206	6.879423	-2.94	0.0041	-33.8745	-6.5667
ChABC	CNTNAP2	before	P	CNTNAP2	after	-19.6905	7.171028	-2.75	0.0072	-33.9231	-5.4579
ChABC	C57	after	ChABC	CNTNAP2	after	17.5169	6.601103	2.65	0.0093	4.4154	30.6184
ChABC	CNTNAP2	after	P	CNTNAP2	before	17.9802	6.879423	2.61	0.0104	4.3262	31.6341
ChABC	CNTNAP2	after	ChABC	CNTNAP2	before	14.1812	6.490312	2.18	0.0336	1.1456	27.2168
ChABC	C57	before	P	C57	before	14.951	6.983885	2.14	0.0348	1.0897	28.8123
P	C57	after	P	CNTNAP2	after	14.7114	7.615081	1.93	0.0563	-0.4026	29.8253
P	C57	before	P	CNTNAP2	before	12.7077	7.24752	1.75	0.0827	-1.6768	27.0923
ChABC	C57	after	P	CNTNAP2	after	12.0076	7.364616	1.63	0.1063	-2.6092	26.6245
ChABC	C57	before	P	C57	after	-10.542	6.983885	-1.51	0.1344	-24.4033	3.3192
ChABC	C57	before	ChABC	CNTNAP2	after	9.6786	6.601103	1.47	0.1458	-3.4229	22.7801
P	C57	before	P	CNTNAP2	after	-10.7817	7.615081	-1.42	0.16	-25.8957	4.3323
ChABC	CNTNAP2	before	P	C57	before	-8.9088	6.779434	-1.31	0.1919	-22.3643	4.5467

ChABC	C57	after	ChABC	C57	before	7.8383	6.825031	1.15	0.2564	-5.8807	21.5573
ChABC	CNTNAP2	after	P	C57	before	5.2724	6.879423	0.77	0.4453	-8.3815	18.9264
ChABC	CNTNAP2	after	P	CNTNAP2	after	-5.5093	7.26563	-0.76	0.4501	-19.9296	8.9111
ChABC	C57	before	P	CNTNAP2	after	4.1693	7.364616	0.57	0.5726	-10.4475	18.7862
ChABC	CNTNAP2	before	P	CNTNAP2	before	3.7989	6.779434	0.56	0.5765	-9.6565	17.2544
ChABC	C57	after	P	C57	after	-2.7037	6.983885	-0.39	0.6995	-16.565	11.1575

trt	mouse	time					Least Squares Mean
P	C57	after	A				254.5069
ChABC	C57	after	A				251.8032
ChABC	C57	before	A	B			243.9649
P	CNTNAP2	after	A	B	C		239.7956
ChABC	CNTNAP2	after		B	C		234.2863
P	C57	before			C	D	229.0139
ChABC	CNTNAP2	before				D	220.1051
P	CNTNAP2	before				D	216.3061

use log (x+1) to fullfill normality assumption **S-FIG11A**

Source	Nparm	DFNum	DFDen	F Ratio	Prob > F
mouse	1	1	18	1.904832	0.1844
trt	1	1	18	26.67911	<.0001
mouse*trt	1	1	18	0.141917	0.7108

mouse	trt	-mouse	-trt	Difference	Std Error	t Ratio	Prob>|t|	Lower 95%	Upper 95%
C57	ChABC	CNTNAP2	P	-1.96836	0.462954	-4.25	0.0005	-2.94099	-0.99573
CNTNAP2	ChABC	CNTNAP2	P	-1.6666	0.432562	-3.85	0.0012	-2.57538	-0.75782
C57	ChABC	C57	P	-1.44002	0.417895	-3.45	0.0029	-2.31798	-0.56205
C57	P	CNTNAP2	ChABC	1.13826	0.383953	2.96	0.0083	0.3316	1.94491
C57	P	CNTNAP2	P	-0.52834	0.445477	-1.19	0.251	-1.46425	0.40757
C57	ChABC	CNTNAP2	ChABC	-0.30176	0.404099	-0.75	0.4649	-1.15074	0.54722

mouse	trt			Least Squares Mean
CNTNAP2	P	A		2.248718
C57	P	A		1.720379
CNTNAP2	ChABC		B	0.58212
C57	ChABC		B	0.280359

S-FIG11B

Source	Nparm	DFNum	DFDen	F Ratio	Prob > F
mouse	1	1	18	0.54366	0.4704
trt	1	1	18	43.90765	<.0001
mouse*trt	1	1	18	5.504163	0.0306

mouse	trt	-mouse	-trt	Difference	Std Error	t Ratio	Prob>|t|	Lower 95%	Upper 95%
CNTNAP2	ChABC	CNTNAP2	P	-32.7367	5.248124	-6.24	<.0001	-43.7626	-21.7108
C57	ChABC	CNTNAP2	P	-26.867	5.616852	-4.78	0.0001	-38.6675	-15.0664
C57	P	CNTNAP2	ChABC	21.4865	4.658368	4.61	0.0002	11.6996	31.2734
C57	ChABC	C57	P	-15.6167	5.070169	-3.08	0.0065	-26.2688	-4.9647
C57	P	CNTNAP2	P	-11.2502	5.404819	-2.08	0.0519	-22.6053	0.1049
C57	ChABC	CNTNAP2	ChABC	5.8697	4.902791	1.2	0.2467	-4.4306	16.1701

S-FIG11

mouse	trt			Least Squares Mean
CNTNAP2	P	A		42.01474
C57	P	A		30.76452
C57	ChABC		B	15.14777
CNTNAP2	ChABC		B	9.278026

use log (x+1) to fullfill normality assumption

S-FIG11C

Source	Nparm	DFNum	DFDen	F Ratio	Prob > F
mouse	1	1	18	0.905661	0.3539
trt	1	1	18	5.43757	0.0315
mouse*trt	1	1	18	0.642175	0.4334

mouse	trt	-mouse	-trt	Difference	Std Error	t Ratio	Prob>|t|	Lower 95%	Upper 95%
C57	ChABC	C57	P	-1.02301	0.453716	-2.25	0.0368	-1.97623	-0.06979
C57	ChABC	CNTNAP2	P	-1.07208	0.502637	-2.13	0.047	-2.12809	-0.01608
C57	ChABC	CNTNAP2	ChABC	-0.57237	0.438738	-1.3	0.2085	-1.49412	0.34938
C57	P	CNTNAP2	ChABC	0.45064	0.416865	1.08	0.294	-0.42516	1.32644
CNTNAP2	ChABC	CNTNAP2	P	-0.49972	0.469641	-1.06	0.3014	-1.48639	0.48696
C57	P	CNTNAP2	P	-0.04907	0.483663	-0.1	0.9203	-1.06521	0.96706

mouse	trt			Least Squares Mean
CNTNAP2	P	A		3.760779
C57	P	A		3.711704
CNTNAP2	ChABC	A	B	3.261064
C57	ChABC		B	2.688695

use log (x+1) to fullfill normality assumption

S-FIG12A

Source	Nparm	DFNum	DFDen	F Ratio	Prob > F
mouse	1	1	18	0.030468	0.8634
trt	1	1	18	9.205772	0.0071
mouse*trt	1	1	18	1.160409	0.2956

mouse	trt	-mouse	-trt	Difference	Std Error	t Ratio	Prob>|t|	Lower 95%	Upper 95%
CNTNAP2	ChABC	CNTNAP2	P	-1.00687	0.326537	-3.08	0.0064	-1.6929	-0.32085
C57	ChABC	CNTNAP2	P	-0.78581	0.326537	-2.41	0.0271	-1.47184	-0.09978
C57	P	CNTNAP2	ChABC	0.70031	0.365079	1.92	0.0711	-0.06669	1.46731
C57	ChABC	C57	P	-0.47924	0.365079	-1.31	0.2058	-1.24625	0.28776
C57	P	CNTNAP2	P	-0.30656	0.365079	-0.84	0.4121	-1.07357	0.46044
C57	ChABC	CNTNAP2	ChABC	0.22107	0.326537	0.68	0.507	-0.46496	0.9071

mouse	trt			Least Squares Mean
CNTNAP2	P	A		1.339003
C57	P	A	B	1.03244
C57	ChABC		B	0.553195
CNTNAP2	ChABC		B	0.332129

S-FIG12B

Source	Nparm	DFNum	DFDen	F Ratio	Prob > F
mouse	1	1	18	1.397138	0.2526
trt	1	1	18	12.99039	0.002
mouse*trt	1	1	18	2.061454	0.1682

mouse	trt	-mouse	-trt	Difference	Std Error	t Ratio	Prob>|t|	Lower 95%	Upper 95%
CNTNAP2	ChABC	CNTNAP2	P	-18.1011	4.788659	-3.78	0.0014	-28.1617	-8.0405
C57	ChABC	CNTNAP2	P	-17.1897	4.788659	-3.59	0.0021	-27.2503	-7.1291
C57	P	CNTNAP2	P	-9.4018	5.353884	-1.76	0.0961	-20.6498	1.8463
C57	P	CNTNAP2	ChABC	8.6994	5.353884	1.62	0.1216	-2.5487	19.9475
C57	ChABC	C57	P	-7.7879	5.353884	-1.45	0.163	-19.036	3.4601
C57	ChABC	CNTNAP2	ChABC	0.9114	4.788659	0.19	0.8512	-9.1492	10.972

mouse	trt			Least Squares Mean
CNTNAP2	P	A		22.86632
C57	P	A	B	13.46457
C57	ChABC		B	5.676623
CNTNAP2	ChABC		B	4.765208

use log (x+1) to fullfill normality assumption **S-FIG12C**

Source	Nparm	DFNum	DFDen	F Ratio	Prob > F
mouse	1	1	18	0.367067	0.5522
trt	1	1	18	0.736872	0.402
mouse*trt	1	1	18	2.290353	0.1475

mouse	trt	-mouse	-trt	Difference	Std Error	t Ratio	Prob>|t|	Lower 95%	Upper 95%
C57	ChABC	C57	P	-0.20943	0.476662	-0.44	0.6656	-1.21086	0.791998
C57	ChABC	CNTNAP2	ChABC	-0.29019	0.42634	-0.68	0.5048	-1.18589	0.605519
C57	ChABC	CNTNAP2	P	0.468208	0.42634	1.1	0.2866	-0.4275	1.363915
C57	P	CNTNAP2	ChABC	-0.08076	0.476662	-0.17	0.8674	-1.08219	0.920676
C57	P	CNTNAP2	P	0.677641	0.476662	1.42	0.1722	-0.32379	1.679072
CNTNAP2	ChABC	CNTNAP2	P	0.758396	0.42634	1.78	0.0922	-0.13731	1.654102

mouse	trt		Least Squares Mean
CNTNAP2	ChABC	A	2.941771
C57	P	A	2.861016
C57	ChABC	A	2.651583
CNTNAP2	P	A	2.183375

S-FIG13A

Source	Nparm	DFNum	DFDen	F Ratio	Prob > F
trt	1	1	24.5	0.453302	0.5071
mouse	1	1	24.5	1.13323	0.2975
trt*mouse	1	1	24.5	1.534208	0.2272
time	1	1	23.2	2.602301	0.1202
trt*time	1	1	23.2	0.01922	0.8909
mouse*tim	1	1	23.2	7.588484	0.0112
trt*mouse*	1	1	23.2	0.096129	0.7593

trt	mouse	time	-trt	-mouse	-time	Difference	Std Error	t Ratio	Prob>|t|	Lower 95%	Upper 95%
ChABC	C57	before	P	C57	after	-9.67333	3.581366	-2.7	0.0106	-16.946	-2.4006
ChABC	C57	after	ChABC	C57	before	5.08	2.117788	2.4	0.0253	0.6909	9.4691
P	C57	after	P	C57	before	5.495	2.445411	2.25	0.0349	0.4269	10.5631
P	C57	after	P	CNTNAP2	after	8.99338	4.14426	2.17	0.0361	0.6146	17.3722
ChABC	CNTNAP2	after	P	C57	after	-7.15333	3.581366	-2	0.0537	-14.426	0.1194
P	C57	after	P	CNTNAP2	before	7.06833	3.828641	1.85	0.0734	-0.7065	14.8432
ChABC	CNTNAP2	before	P	C57	after	-6.31458	3.581366	-1.76	0.0867	-13.5873	0.9581
ChABC	C57	after	P	C57	after	-4.59333	3.581366	-1.28	0.2081	-11.866	2.6794
ChABC	C57	before	P	C57	before	-4.17833	3.581366	-1.17	0.2513	-11.451	3.0944
ChABC	C57	after	P	CNTNAP2	after	4.40005	3.91696	1.12	0.268	-3.5158	12.3159
ChABC	C57	before	ChABC	CNTNAP2	before	-3.35875	3.3157	-1.01	0.3181	-10.092	3.3745
P	C57	before	P	CNTNAP2	after	3.49838	4.14426	0.84	0.4037	-4.8804	11.8772
ChABC	C57	after	ChABC	CNTNAP2	after	2.56	3.3157	0.77	0.4453	-4.1732	9.2932
ChABC	C57	before	ChABC	CNTNAP2	after	-2.52	3.3157	-0.76	0.4524	-9.2532	4.2132
ChABC	C57	before	P	CNTNAP2	before	-2.605	3.581366	-0.73	0.4719	-9.8777	4.6677
ChABC	C57	after	P	CNTNAP2	before	2.475	3.581366	0.69	0.4941	-4.7977	9.7477
ChABC	CNTNAP2	before	P	CNTNAP2	after	2.6788	3.91696	0.68	0.498	-5.2371	10.5947
P	CNTNAP2	after	P	CNTNAP2	before	-1.92505	2.914864	-0.66	0.515	-7.9281	4.078
ChABC	C57	after	ChABC	CNTNAP2	before	1.72125	3.3157	0.52	0.607	-5.012	8.4545
ChABC	CNTNAP2	after	P	CNTNAP2	after	1.84005	3.91696	0.47	0.6411	-6.0758	9.7559
ChABC	CNTNAP2	after	P	C57	before	-1.65833	3.581366	-0.46	0.6462	-8.931	5.6144
P	C57	before	P	CNTNAP2	before	1.57333	3.828641	0.41	0.6836	-6.2015	9.3482

ChABC	CNTNAP2	after	ChABC	CNTNAP2	before	-0.83875	2.117788	-0.4	0.6958	-5.2278	3.5503
ChABC	C57	after	P	C57	before	0.90167	3.581366	0.25	0.8027	-6.371	8.1744
ChABC	CNTNAP2	before	P	C57	before	-0.81958	3.581366	-0.23	0.8203	-8.0923	6.4531
ChABC	CNTNAP2	before	P	CNTNAP2	before	0.75375	3.581366	0.21	0.8345	-6.519	8.0265
ChABC	C57	before	P	CNTNAP2	after	-0.67995	3.91696	-0.17	0.8631	-8.5958	7.2359
ChABC	CNTNAP2	after	P	CNTNAP2	before	-0.085	3.581366	-0.02	0.9812	-7.3577	7.1877

trt	mouse	time				Least Squares Mean
P	C57	after	A			15.53833
ChABC	C57	after	A	B		10.945
P	C57	before		B	C	10.04333
ChABC	CNTNAP2	before	A	B	C	9.22375
P	CNTNAP2	before	A	B	C	8.47
ChABC	CNTNAP2	after	A	B	C	8.385
P	CNTNAP2	after		B	C	6.544953
ChABC	C57	before			C	5.865

S-FIG13B

Source	Nparm	DFNum	DFDen	F Ratio	Prob > F
trt	1	1	22	0.009794	0.9221
mouse	1	1	22	9.827452	0.0048
trt*mouse	1	1	22	0.298544	0.5903
time	1	1	21.8	1.297588	0.267
trt*time	1	1	21.8	0.074014	0.7881
mouse*tim	1	1	21.8	0.796192	0.382
trt*mouse*	1	1	21.8	0.07187	0.7912

trt	mouse	time	-trt	-mouse	-time	Difference	Std Error	t Ratio	Prob>|t|	Lower 95%	Upper 95%
P	C57	after	P	CNTNAP2	before	-9.795	3.805043	-2.57	0.0137	-17.4757	-2.1143
ChABC	CNTNAP2	after	P	C57	after	8.47803	3.672134	2.31	0.026	1.0671	15.889
ChABC	CNTNAP2	before	P	C57	after	7.98542	3.559292	2.24	0.0302	0.8008	15.17
ChABC	C57	after	P	CNTNAP2	before	-8.48667	3.805043	-2.23	0.0312	-16.1673	-0.806
P	C57	after	P	CNTNAP2	after	-8.47333	3.805043	-2.23	0.0314	-16.154	-0.7927
ChABC	C57	after	ChABC	CNTNAP2	after	-7.1697	3.672134	-1.95	0.0576	-14.5806	0.2412
ChABC	C57	after	P	CNTNAP2	after	-7.165	3.805043	-1.88	0.0667	-14.8457	0.5157
ChABC	C57	after	ChABC	CNTNAP2	before	-6.67708	3.559292	-1.88	0.0677	-13.8617	0.5075
P	C57	before	P	CNTNAP2	before	-6.37667	3.805043	-1.68	0.1013	-14.0573	1.304
ChABC	CNTNAP2	after	P	C57	before	5.0597	3.672134	1.38	0.1756	-2.3512	12.4706
ChABC	C57	before	P	CNTNAP2	before	-5.08167	3.805043	-1.34	0.189	-12.7623	2.599
P	C57	before	P	CNTNAP2	after	-5.055	3.805043	-1.33	0.1912	-12.7357	2.6257
ChABC	CNTNAP2	before	P	C57	before	4.56708	3.559292	1.28	0.2065	-2.6175	11.7517
ChABC	C57	before	P	C57	after	4.71333	3.805043	1.24	0.2224	-2.9673	12.394
ChABC	C57	before	ChABC	CNTNAP2	after	-3.7647	3.672134	-1.03	0.3111	-11.1756	3.6462
ChABC	C57	before	P	CNTNAP2	after	-3.76	3.805043	-0.99	0.3288	-11.4407	3.9207
P	C57	after	P	C57	before	-3.41833	3.437561	-0.99	0.3311	-10.5564	3.7197
ChABC	C57	after	ChABC	C57	before	-3.405	3.437561	-0.99	0.3329	-10.5431	3.7331
ChABC	C57	before	ChABC	CNTNAP2	before	-3.27208	3.559292	-0.92	0.3632	-10.4567	3.9125
ChABC	C57	after	P	C57	before	-2.11	3.805043	-0.55	0.5822	-9.7907	5.5707
ChABC	CNTNAP2	before	P	CNTNAP2	before	-1.80958	3.559292	-0.51	0.6138	-8.9942	5.375

P	CNTNAP2	after	P	CNTNAP2	before	-1.32167	3.437561	-0.38	0.7044	-8.4597	5.8164
ChABC	CNTNAP2	after	P	CNTNAP2	before	-1.31697	3.672134	-0.36	0.7217	-8.7279	6.094
ChABC	C57	after	P	C57	after	1.30833	3.805043	0.34	0.7327	-6.3723	8.989
ChABC	C57	before	P	C57	before	1.295	3.805043	0.34	0.7353	-6.3857	8.9757
ChABC	CNTNAP2	after	ChABC	CNTNAP2	before	0.49261	3.111049	0.16	0.8756	-5.9441	6.9293
ChABC	CNTNAP2	before	P	CNTNAP2	after	-0.48792	3.559292	-0.14	0.8916	-7.6725	6.6967
ChABC	CNTNAP2	after	P	CNTNAP2	after	0.0047	3.672134	0	0.999	-7.4062	7.4156

S-FIG13B

trt	mouse	time				Least Squares Mean
P	CNTNAP2	before	A			13.33833
ChABC	CNTNAP2	after	A	B		12.02137
P	CNTNAP2	after	A	B		12.01667
ChABC	CNTNAP2	before	A	B		11.52875
ChABC	C57	before	A	B	C	8.256667
P	C57	before	A	B	C	6.961667
ChABC	C57	after		B	C	4.851667
P	C57	after			C	3.543333

S-FIG13C

Source	Nparm	DFNum	DFDen	F Ratio	Prob > F
trt	1	1	23.6	0.16127	0.6916
mouse	1	1	23.6	0.861865	0.3626
trt*mouse	1	1	23.6	0.425081	0.5207
time	1	1	22.5	3.387149	0.079
trt*time	1	1	22.5	6.446711	0.0185
mouse*tim	1	1	22.5	0.238438	0.6301
trt*mouse*	1	1	22.5	0.591682	0.4498

trt	mouse	time	-trt	-mouse	-time	Difference	Std Error	t Ratio	Prob>|t|	Lower 95%	Upper 95%
P	C57	after	P	C57	before	-1.83333	0.905684	-2.02	0.0556	-3.71486	0.048191
P	CNTNAP2	after	P	CNTNAP2	before	-2.08464	1.072735	-1.94	0.0635	-4.29566	0.12638
ChABC	C57	after	P	CNTNAP2	before	-2.16667	1.202832	-1.8	0.0799	-4.60472	0.271385
P	C57	after	P	CNTNAP2	before	-2.16667	1.285882	-1.68	0.1005	-4.77305	0.43972
ChABC	C57	before	P	CNTNAP2	before	-1.91667	1.202832	-1.59	0.1197	-4.35472	0.521385
ChABC	C57	after	ChABC	CNTNAP2	after	-1.75	1.113606	-1.57	0.1247	-4.0072	0.507197
ChABC	C57	after	P	C57	before	-1.83333	1.202832	-1.52	0.1361	-4.27138	0.604718
ChABC	CNTNAP2	after	P	C57	after	1.75	1.202832	1.45	0.1542	-0.68805	4.188052
ChABC	C57	before	ChABC	CNTNAP2	after	-1.5	1.113606	-1.35	0.1863	-3.7572	0.757197
ChABC	C57	before	P	C57	before	-1.58333	1.202832	-1.32	0.1962	-4.02138	0.854718
ChABC	CNTNAP2	after	P	CNTNAP2	after	1.66797	1.333155	1.25	0.2179	-1.02298	4.358931
P	C57	before	P	CNTNAP2	after	1.75131	1.408541	1.24	0.2208	-1.09281	4.595427
ChABC	CNTNAP2	after	ChABC	CNTNAP2	before	0.875	0.784345	1.12	0.277	-0.75445	2.504448
ChABC	CNTNAP2	before	P	CNTNAP2	before	-1.29167	1.202832	-1.07	0.2899	-3.72972	1.146385
ChABC	CNTNAP2	before	P	C57	before	-0.95833	1.202832	-0.8	0.4307	-3.39638	1.479718
ChABC	C57	after	ChABC	CNTNAP2	before	-0.875	1.113606	-0.79	0.4371	-3.1322	1.382197
ChABC	CNTNAP2	before	P	C57	after	0.875	1.202832	0.73	0.4716	-1.56305	3.313052
ChABC	CNTNAP2	before	P	CNTNAP2	after	0.79297	1.333155	0.59	0.5552	-1.89798	3.483931
ChABC	C57	before	ChABC	CNTNAP2	before	-0.625	1.113606	-0.56	0.5781	-2.8822	1.632197
ChABC	CNTNAP2	after	P	CNTNAP2	before	-0.41667	1.202832	-0.35	0.731	-2.85472	2.021385
ChABC	C57	after	ChABC	C57	before	-0.25	0.784345	-0.32	0.753	-1.87945	1.379448
P	C57	before	P	CNTNAP2	before	-0.33333	1.285882	-0.26	0.7969	-2.93972	2.273053

ChABC	C57	before	P	C57	after	0.25	1.202832	0.21	0.8365	-2.18805	2.688052
ChABC	C57	before	P	CNTNAP2	after	0.16797	1.333155	0.13	0.9003	-2.52298	2.858931
ChABC	CNTNAP2	after	P	C57	before	-0.08333	1.202832	-0.07	0.9451	-2.52138	2.354718
ChABC	C57	after	P	CNTNAP2	after	-0.08203	1.333155	-0.06	0.9512	-2.77298	2.608931
P	C57	after	P	CNTNAP2	after	-0.08203	1.408541	-0.06	0.9538	-2.92615	2.762094
ChABC	C57	after	P	C57	after	8.88E-16	1.202832	0	1	-2.43805	2.438052

trt	mouse	time		Least Squares Mean
P	CNTNAP2	before	A	4.666667
P	C57	before	A	4.333333
ChABC	CNTNAP2	after	A	4.25
ChABC	CNTNAP2	before	A	3.375
ChABC	C57	before	A	2.75
P	CNTNAP2	after	A	2.582026
ChABC	C57	after	A	2.5
P	C57	after	A	2.5

S-FIG13D

Source	Nparm	DFNum	DFDen	F Ratio	Prob > F
trt	1	1	21.9	0.064686	0.8016
mouse	1	1	21.9	9.767183	0.005
trt*mouse	1	1	21.9	0.240077	0.629
time	1	1	21.9	2.221821	0.1504
trt*time	1	1	21.9	0.059928	0.8089
mouse*tim	1	1	21.9	0.121534	0.7307
trt*mouse*	1	1	21.9	0.004441	0.9475

trt	mouse	time	-trt	-mouse	-time	Difference	Std Error	t Ratio	Prob>|t|	Lower 95%	Upper 95%
ChABC	CNTNAP2	before	P	C57	after	3.79167	1.520937	2.49	0.0166	0.72436	6.85898
P	C57	after	P	CNTNAP2	before	-3.83333	1.62595	-2.36	0.023	-7.11243	-0.55424
ChABC	C57	after	ChABC	CNTNAP2	before	-3.45833	1.520937	-2.27	0.028	-6.52564	-0.39102
ChABC	C57	after	P	CNTNAP2	before	-3.5	1.62595	-2.15	0.037	-6.77909	-0.22091
ChABC	CNTNAP2	before	P	C57	before	3.125	1.520937	2.05	0.046	0.05769	6.19231
P	C57	before	P	CNTNAP2	before	-3.16667	1.62595	-1.95	0.058	-6.44576	0.11243
P	C57	after	P	CNTNAP2	after	-2.5	1.62595	-1.54	0.1315	-5.77909	0.77909
ChABC	C57	before	ChABC	CNTNAP2	before	-2.29167	1.520937	-1.51	0.1392	-5.35898	0.77564
ChABC	C57	before	P	CNTNAP2	before	-2.33333	1.62595	-1.44	0.1585	-5.61243	0.94576
ChABC	CNTNAP2	after	P	C57	after	2.17231	1.570948	1.38	0.1739	-0.99585	5.34047
ChABC	C57	after	P	CNTNAP2	after	-2.16667	1.62595	-1.33	0.1897	-5.44576	1.11243
ChABC	C57	after	ChABC	CNTNAP2	after	-1.83898	1.570948	-1.17	0.2482	-5.00714	1.32918
P	C57	before	P	CNTNAP2	after	-1.83333	1.62595	-1.13	0.2658	-5.11243	1.44576
ChABC	CNTNAP2	after	ChABC	CNTNAP2	before	-1.61936	1.478383	-1.1	0.2847	-4.67722	1.43851
ChABC	CNTNAP2	after	P	CNTNAP2	before	-1.66102	1.570948	-1.06	0.2963	-4.82918	1.50714
ChABC	CNTNAP2	after	P	C57	before	1.50564	1.570948	0.96	0.3432	-1.66252	4.6738
ChABC	C57	before	P	C57	after	1.5	1.62595	0.92	0.3614	-1.77909	4.77909
ChABC	CNTNAP2	before	P	CNTNAP2	after	1.29167	1.520937	0.85	0.4004	-1.77564	4.35898
P	CNTNAP2	after	P	CNTNAP2	before	-1.33333	1.645595	-0.81	0.4266	-4.75002	2.08336
ChABC	C57	after	ChABC	C57	before	-1.16667	1.645595	-0.71	0.4859	-4.58336	2.25002
ChABC	C57	before	P	CNTNAP2	after	-1	1.62595	-0.62	0.5418	-4.27909	2.27909
ChABC	C57	before	P	C57	before	0.83333	1.62595	0.51	0.6109	-2.44576	4.11243

ChABC	C57	before	ChABC	CNTNAP2	after	-0.67231	1.570948	-0.43	0.6708	-3.84047	2.49585
P	C57	after	P	C57	before	-0.66667	1.645595	-0.41	0.6894	-4.08336	2.75002
ChABC	CNTNAP2	after	P	CNTNAP2	after	-0.32769	1.570948	-0.21	0.8358	-3.49585	2.84047
ChABC	C57	after	P	C57	after	0.33333	1.62595	0.21	0.8385	-2.94576	3.61243
ChABC	C57	after	P	C57	before	-0.33333	1.62595	-0.21	0.8385	-3.61243	2.94576
ChABC	CNTNAP2	before	P	CNTNAP2	before	-0.04167	1.520937	-0.03	0.9783	-3.10898	3.02564

S-FIG11A

trt	mouse	time				Least Squares Mean
P	CNTNAP2	before	A	B		5.666667
ChABC	CNTNAP2	before	A			5.625
P	CNTNAP2	after	A	B	C	4.333333
ChABC	CNTNAP2	after	A	B	C	4.005644
ChABC	C57	before	A	B	C	3.333333
P	C57	before		B	C	2.5
ChABC	C57	after			C	2.166667
P	C57	after			C	1.833333

S-FIG14A

Source	Nparm	DFNum	DFDen	F Ratio	Prob > F
trt	1	1	24.7	0.529262	0.4738
mouse	1	1	24.7	9.850824	0.0044
trt*mouse	1	1	24.7	1.987169	0.1711
time	1	1	24.8	10.19222	0.0038
trt*time	1	1	24.8	2.095911	0.1602
mouse*tim	1	1	24.8	0.388325	0.5389
trt*mouse*	1	1	24.8	1.607101	0.2167

trt	mouse	time	-trt	-mouse	-time	Difference	Std Error	t Ratio	Prob>|t|	Lower 95%	Upper 95%
P	C57	after	P	CNTNAP2	before	-39.3689	8.82507	-4.46	<.0001	-57.1333	-21.6044
ChABC	C57	after	P	CNTNAP2	before	-35.7085	8.2551	-4.33	<.0001	-52.3256	-19.0914
P	C57	before	P	CNTNAP2	before	-27.4092	8.82507	-3.11	0.0032	-45.1736	-9.6448
ChABC	CNTNAP2	after	P	CNTNAP2	before	-25.6123	8.2551	-3.1	0.0033	-42.2294	-8.9952
ChABC	C57	before	P	CNTNAP2	before	-25.315	8.2551	-3.07	0.0036	-41.9321	-8.6979
P	CNTNAP2	after	P	CNTNAP2	before	-28.41	10.07875	-2.82	0.0087	-49.0461	-7.7739
ChABC	CNTNAP2	before	P	CNTNAP2	before	-20.8263	8.2551	-2.52	0.0152	-37.4434	-4.2092
ChABC	CNTNAP2	before	P	C57	after	18.5425	8.2551	2.25	0.0295	1.9254	35.1596
ChABC	C57	after	ChABC	CNTNAP2	before	-14.8822	7.64273	-1.95	0.0576	-30.2666	0.5022
ChABC	C57	before	P	C57	after	14.0539	8.2551	1.7	0.0954	-2.5632	30.671
ChABC	CNTNAP2	after	P	C57	after	13.7565	8.2551	1.67	0.1024	-2.8606	30.3736
ChABC	C57	after	ChABC	CNTNAP2	after	-10.0962	7.64273	-1.32	0.193	-25.4806	5.2882
ChABC	C57	after	ChABC	C57	before	-10.3936	7.7686	-1.34	0.1939	-26.4562	5.6691
P	C57	after	P	C57	before	-11.9596	8.9704	-1.33	0.1954	-30.5072	6.588
P	C57	after	P	CNTNAP2	after	-10.9589	9.94962	-1.1	0.2764	-30.9866	9.0689
ChABC	C57	after	P	C57	before	-8.2993	8.2551	-1.01	0.32	-24.9164	8.3178
ChABC	CNTNAP2	before	P	CNTNAP2	after	7.5837	9.44774	0.8	0.4263	-11.4338	26.6011
ChABC	CNTNAP2	before	P	C57	before	6.5829	8.2551	0.8	0.4293	-10.0342	23.2
ChABC	C57	after	P	CNTNAP2	after	-7.2985	9.44774	-0.77	0.4438	-26.316	11.7189
ChABC	CNTNAP2	after	ChABC	CNTNAP2	before	-4.786	7.7686	-0.62	0.5438	-20.8487	11.2767
ChABC	C57	before	ChABC	CNTNAP2	before	-4.4886	7.64273	-0.59	0.5599	-19.8731	10.8958

ChABC	C57	after	P	C57	after	3.6603	8.2551	0.44	0.6596	-12.9568	20.2774
ChABC	C57	before	P	CNTNAP2	after	3.095	9.44774	0.33	0.7447	-15.9224	22.1125
ChABC	CNTNAP2	after	P	CNTNAP2	after	2.7977	9.44774	0.3	0.7685	-16.2198	21.8151
ChABC	C57	before	P	C57	before	2.0943	8.2551	0.25	0.8009	-14.5228	18.7113
ChABC	CNTNAP2	after	P	C57	before	1.7969	8.2551	0.22	0.8286	-14.8202	18.414
P	C57	before	P	CNTNAP2	after	1.0008	9.94962	0.1	0.9203	-19.027	21.0285
ChABC	C57	before	ChABC	CNTNAP2	after	0.2973	7.64273	0.04	0.9691	-15.0871	15.6818

trt	mouse	time				Least Squares Mean
P	CNTNAP2	before	A			69.95087
ChABC	CNTNAP2	before		B		49.12454
ChABC	C57	before		B	C	44.6359
ChABC	CNTNAP2	after		B	C	44.33855
P	C57	before		B	C	42.54165
P	CNTNAP2	after		B	C	41.54088
ChABC	C57	after		B	C	34.24234
P	C57	after			C	30.58201

S-FIG14B

Source	Nparm	DFNum	DFDen	F Ratio	Prob > F
trt	1	1	21.9	0.155588	0.6971
mouse	1	1	21.9	8.506566	0.008
trt*mouse	1	1	21.9	6.963996	0.015
time	1	1	22.1	27.83303	<.0001
trt*time	1	1	22.1	1.535751	0.2282
mouse*tim	1	1	22.1	0.150972	0.7013
trt*mouse*	1	1	22.1	6.514259	0.0181

trt	mouse	time	-trt	-mouse	-time	Difference	Std Error	t Ratio	Prob>|t|	Lower 95%	Upper 95%
ChABC	CNTNAP2	before	P	C57	after	41.9683	7.446617	5.64	<.0001	26.9357	57.001
ChABC	C57	after	ChABC	CNTNAP2	before	-36.5107	7.446617	-4.9	<.0001	-51.5433	-21.478
ChABC	C57	after	P	C57	before	-34.5907	7.960768	-4.35	<.0001	-50.6613	-18.5202
P	C57	after	P	C57	before	-40.0484	8.694078	-4.61	0.0001	-58.0878	-22.009
ChABC	C57	before	ChABC	CNTNAP2	before	-28.5792	7.446617	-3.84	0.0004	-43.6118	-13.5466
ChABC	CNTNAP2	before	P	CNTNAP2	after	28.5425	7.446617	3.83	0.0004	13.5099	43.5751
P	C57	after	P	CNTNAP2	before	-28.5633	7.960768	-3.59	0.0009	-44.6338	-12.4927
ChABC	C57	before	P	C57	before	-26.6593	7.960768	-3.35	0.0017	-42.7298	-10.5887
P	C57	before	P	CNTNAP2	after	26.6226	7.960768	3.34	0.0018	10.552	42.6931
ChABC	CNTNAP2	after	ChABC	CNTNAP2	before	-26.2598	7.761841	-3.38	0.0025	-42.3065	-10.2132
ChABC	CNTNAP2	after	P	C57	before	-24.3399	7.681668	-3.17	0.0029	-39.8437	-8.8361
ChABC	C57	after	P	CNTNAP2	before	-23.1056	7.960768	-2.9	0.0059	-39.1762	-7.0351
ChABC	CNTNAP2	after	P	C57	after	15.7085	7.681668	2.04	0.0472	0.2047	31.2123
ChABC	C57	before	P	CNTNAP2	before	-15.1742	7.960768	-1.91	0.0636	-31.2447	0.8964
ChABC	CNTNAP2	before	P	CNTNAP2	before	13.4051	7.446617	1.8	0.0791	-1.6276	28.4377
P	CNTNAP2	after	P	CNTNAP2	before	-15.1375	8.694078	-1.74	0.0958	-33.1769	2.902
P	C57	after	P	CNTNAP2	after	-13.4258	7.960768	-1.69	0.0992	-29.4964	2.6447
ChABC	C57	before	P	C57	after	13.3891	7.960768	1.68	0.1001	-2.6814	29.4597
ChABC	CNTNAP2	after	P	CNTNAP2	before	-12.8548	7.681668	-1.67	0.1017	-28.3586	2.649
P	C57	before	P	CNTNAP2	before	11.4851	7.960768	1.44	0.1566	-4.5854	27.5557
ChABC	C57	after	ChABC	CNTNAP2	after	-10.2508	7.681668	-1.33	0.1893	-25.7546	5.253
ChABC	C57	after	P	CNTNAP2	after	-7.9682	7.960768	-1	0.3227	-24.0387	8.1024

ChABC	C57	after	ChABC	C57	before	-7.9315	8.694078	-0.91	0.3716	-25.9709	10.108
ChABC	C57	after	P	C57	after	5.4577	7.960768	0.69	0.4968	-10.6129	21.5282
ChABC	C57	before	ChABC	CNTNAP2	after	-2.3194	7.681668	-0.3	0.7642	-17.8232	13.1844
ChABC	CNTNAP2	after	P	CNTNAP2	after	2.2827	7.681668	0.3	0.7678	-13.2211	17.7865
ChABC	CNTNAP2	before	P	C57	before	1.9199	7.446617	0.26	0.7978	-13.1127	16.9526
ChABC	C57	before	P	CNTNAP2	after	-0.0367	7.960768	0	0.9963	-16.1073	16.0339

trt	mouse	time					Least Squares Mean
ChABC	CNTNAP2	before	A				71.13458
P	C57	before	A				69.21464
P	CNTNAP2	before	A	B			57.72953
ChABC	CNTNAP2	after		B	C		44.87473
P	CNTNAP2	after		B	C	D	42.59206
ChABC	C57	before		B	C	D	42.55536
ChABC	C57	after			C	D	34.6239
P	C57	after				D	29.16624

S-FIG14C

Source	Nparm	DFNum	DFDen	F Ratio	Prob > F
trt	1	1	25	1.030351	0.3198
mouse	1	1	25	14.07437	0.0009
trt*mouse	1	1	25	0.345134	0.5622
time	1	1	24.7	8.862445	0.0064
trt*time	1	1	24.7	1.345628	0.2571
mouse*tim	1	1	24.7	0.089605	0.7672
trt*mouse*	1	1	24.7	1.345628	0.2571

trt	mouse	time	-trt	-mouse	-time	Difference	Std Error	t Ratio	Prob>|t|	Lower 95%	Upper 95%
ChABC	C57	after	P	CNTNAP2	before	-24.75	5.346082	-4.63	<.0001	-35.5148	-13.9852
P	C57	after	P	CNTNAP2	before	-23.5	5.715202	-4.11	0.0002	-35.0081	-11.9919
ChABC	C57	before	P	CNTNAP2	before	-17.75	5.346082	-3.32	0.0018	-28.5148	-6.9852
P	C57	before	P	CNTNAP2	before	-16.5	5.715202	-2.89	0.0059	-28.0081	-4.9919
ChABC	C57	after	ChABC	CNTNAP2	before	-14	4.94951	-2.83	0.0069	-23.9663	-4.0337
ChABC	CNTNAP2	after	P	CNTNAP2	before	-13.25	5.346082	-2.48	0.017	-24.0148	-2.4852
ChABC	CNTNAP2	before	P	C57	after	12.75	5.346082	2.38	0.0213	1.9852	23.5148
P	CNTNAP2	after	P	CNTNAP2	before	-14.6302	6.121491	-2.39	0.0238	-27.1703	-2.0901
ChABC	C57	after	ChABC	CNTNAP2	after	-11.5	4.94951	-2.32	0.0247	-21.4663	-1.5337
ChABC	CNTNAP2	before	P	CNTNAP2	before	-10.75	5.346082	-2.01	0.0503	-21.5148	0.0148
ChABC	CNTNAP2	after	P	C57	after	10.25	5.346082	1.92	0.0615	-0.5148	21.0148
ChABC	C57	after	P	CNTNAP2	after	-10.1198	6.107635	-1.66	0.1044	-22.4151	2.1755
ChABC	C57	after	P	C57	before	-8.25	5.346082	-1.54	0.1297	-19.0148	2.5148
ChABC	C57	after	ChABC	C57	before	-7	4.643548	-1.51	0.1452	-16.6025	2.6025
ChABC	C57	before	ChABC	CNTNAP2	before	-7	4.94951	-1.41	0.1641	-16.9663	2.9663
P	C57	after	P	CNTNAP2	after	-8.8698	6.433206	-1.38	0.1747	-21.8207	4.0811
P	C57	after	P	C57	before	-7	5.361907	-1.31	0.2045	-18.088	4.088
ChABC	CNTNAP2	before	P	C57	before	5.75	5.346082	1.08	0.2878	-5.0148	16.5148
ChABC	C57	before	P	C57	after	5.75	5.346082	1.08	0.2878	-5.0148	16.5148
ChABC	C57	before	ChABC	CNTNAP2	after	-4.5	4.94951	-0.91	0.3681	-14.4663	5.4663
ChABC	CNTNAP2	before	P	CNTNAP2	after	3.8802	6.107635	0.64	0.5284	-8.4151	16.1755

ChABC	CNTNAP2	after	P	C57	before	3.25	5.346082	0.61	0.5463	-7.5148	14.0148
ChABC	CNTNAP2	after	ChABC	CNTNAP2	before	-2.5	4.643548	-0.54	0.5955	-12.1025	7.1025
ChABC	C57	before	P	CNTNAP2	after	-3.1198	6.107635	-0.51	0.6119	-15.4151	9.1755
P	C57	before	P	CNTNAP2	after	-1.8698	6.433206	-0.29	0.7726	-14.8207	11.0811
ChABC	C57	after	P	C57	after	-1.25	5.346082	-0.23	0.8162	-12.0148	9.5148
ChABC	C57	before	P	C57	before	-1.25	5.346082	-0.23	0.8162	-12.0148	9.5148
ChABC	CNTNAP2	after	P	CNTNAP2	after	1.3802	6.107635	0.23	0.8222	-10.9151	13.6755

trt	mouse	time					Least Squares Mean
P	CNTNAP2	before	A				50
ChABC	CNTNAP2	before	A	B			39.25
ChABC	CNTNAP2	after		B	C		36.75
P	CNTNAP2	after		B	C	D	35.36981
P	C57	before		B	C	D	33.5
ChABC	C57	before		B	C	D	32.25
P	C57	after			C	D	26.5
ChABC	C57	after				D	25.25

S-FIG14D

Source	Nparm	DFNum	DFDen	F Ratio	Prob > F
trt	1	1	21.9	0.049619	0.8258
mouse	1	1	21.9	14.11612	0.0011
trt*mouse	1	1	21.9	4.174555	0.0532
time	1	1	21.9	13.89033	0.0012
trt*time	1	1	21.9	2.370922	0.1379
mouse*tim	1	1	21.9	0.075395	0.7862
trt*mouse*	1	1	21.9	5.774069	0.0252

trt	mouse	time	-trt	-mouse	-time	Difference	Std Error	t Ratio	Prob>|t|	Lower 95%	Upper 95%
ChABC	CNTNAP2	before	P	C57	after	27.5	5.465174	5.03	<.0001	16.4784	38.5216
ChABC	C57	after	ChABC	CNTNAP2	before	-22.8333	5.465174	-4.18	0.0001	-33.855	-11.8117
ChABC	C57	before	ChABC	CNTNAP2	before	-22.6667	5.465174	-4.15	0.0002	-33.6883	-11.645
P	C57	after	P	C57	before	-22.5	5.804558	-3.88	0.0008	-34.5499	-10.4501
P	C57	after	P	CNTNAP2	before	-19.8333	5.842517	-3.39	0.0015	-31.6159	-8.0507
ChABC	C57	after	P	C57	before	-17.8333	5.842517	-3.05	0.0039	-29.6159	-6.0507
ChABC	C57	before	P	C57	before	-17.6667	5.842517	-3.02	0.0042	-29.4493	-5.8841
ChABC	CNTNAP2	before	P	CNTNAP2	after	15	5.465174	2.74	0.0088	3.9784	26.0216
ChABC	CNTNAP2	after	P	C57	after	15.2774	5.644855	2.71	0.0097	3.8934	26.6614
ChABC	C57	after	P	CNTNAP2	before	-15.1667	5.842517	-2.6	0.0129	-26.9493	-3.3841
ChABC	C57	before	P	CNTNAP2	before	-15	5.842517	-2.57	0.0138	-26.7826	-3.2174
ChABC	CNTNAP2	after	ChABC	CNTNAP2	before	-12.2226	5.221679	-2.34	0.0282	-23.0217	-1.4235
P	C57	after	P	CNTNAP2	after	-12.5	5.842517	-2.14	0.0381	-24.2826	-0.7174
ChABC	C57	after	ChABC	CNTNAP2	after	-10.6107	5.644855	-1.88	0.0669	-21.9947	0.7733
ChABC	C57	before	ChABC	CNTNAP2	after	-10.4441	5.644855	-1.85	0.0712	-21.828	0.9399
P	C57	before	P	CNTNAP2	after	10	5.842517	1.71	0.0942	-1.7826	21.7826
ChABC	CNTNAP2	before	P	CNTNAP2	before	7.6667	5.465174	1.4	0.1679	-3.355	18.6883
ChABC	C57	after	P	CNTNAP2	after	-7.8333	5.842517	-1.34	0.187	-19.6159	3.9493
ChABC	C57	before	P	CNTNAP2	after	-7.6667	5.842517	-1.31	0.1964	-19.4493	4.1159
ChABC	CNTNAP2	after	P	C57	before	-7.2226	5.644855	-1.28	0.2076	-18.6066	4.1614
P	CNTNAP2	after	P	CNTNAP2	before	-7.3333	5.804558	-1.26	0.2199	-19.3832	4.7165
ChABC	CNTNAP2	before	P	C57	before	5	5.465174	0.91	0.3654	-6.0216	16.0216

ChABC	C57	before	P	C57	after	4.8333	5.842517	0.83	0.4127	-6.9493	16.6159
ChABC	CNTNAP2	after	P	CNTNAP2	before	-4.5559	5.644855	-0.81	0.4241	-15.9399	6.828
ChABC	C57	after	P	C57	after	4.6667	5.842517	0.8	0.4288	-7.1159	16.4493
ChABC	CNTNAP2	after	P	CNTNAP2	after	2.7774	5.644855	0.49	0.6252	-8.6066	14.1614
P	C57	before	P	CNTNAP2	before	2.6667	5.842517	0.46	0.6504	-9.1159	14.4493
ChABC	C57	after	ChABC	C57	before	-0.1667	5.804558	-0.03	0.9774	-12.2165	11.8832

trt	mouse	time					Least Squares Mean
ChABC	CNTNAP2	before	A				50.5
P	C57	before	A	B			45.5
P	CNTNAP2	before	A	B			42.83333
ChABC	CNTNAP2	after		B	C		38.27739
P	CNTNAP2	after		B	C		35.5
ChABC	C57	before			C	D	27.83333
ChABC	C57	after			C	D	27.66667
P	C57	after				D	23

S-FIG15A

Source	Nparm	DFNum	DFDen	F Ratio	Prob > F
trt	1	1	25.1	0.069475	0.7943
mouse	1	1	25.1	6.299184	0.0189
trt*mouse	1	1	25.1	7.409238	0.0116
time	1	1	24.3	0.00191	0.9655
trt*time	1	1	24.3	0.642514	0.4306
mouse*tim	1	1	24.3	1.126038	0.299
trt*mouse*	1	1	24.3	4.700161	0.0402

trt	mouse	time	-trt	-mouse	-time	Difference	Std Error	t Ratio	Prob>|t|	Lower 95%	Upper 95%
ChABC	C57	after	ChABC	CNTNAP2	after	-4.62821	1.198917	-3.86	0.0004	-7.04811	-2.20832
ChABC	C57	before	ChABC	CNTNAP2	after	-4.48341	1.198917	-3.74	0.0006	-6.9033	-2.06352
ChABC	C57	after	ChABC	CNTNAP2	before	-3.5475	1.198917	-2.96	0.0051	-5.9674	-1.12761
ChABC	C57	before	ChABC	CNTNAP2	before	-3.4027	1.198917	-2.84	0.007	-5.8226	-0.98281
ChABC	CNTNAP2	after	P	CNTNAP2	after	3.93542	1.460656	2.69	0.0099	0.9926	6.87825
ChABC	CNTNAP2	after	P	C57	before	3.35302	1.294978	2.59	0.0132	0.73924	5.9668
ChABC	C57	after	P	CNTNAP2	before	-2.90002	1.294978	-2.24	0.0305	-5.51381	-0.28624
ChABC	C57	before	P	CNTNAP2	before	-2.75522	1.294978	-2.13	0.0393	-5.36901	-0.14144
ChABC	C57	after	P	C57	after	-2.64331	1.294978	-2.04	0.0476	-5.25709	-0.02953
ChABC	CNTNAP2	before	P	CNTNAP2	after	2.85472	1.460656	1.95	0.057	-0.08811	5.79754
ChABC	C57	before	P	C57	after	-2.49851	1.294978	-1.93	0.0605	-5.11229	0.11527
ChABC	CNTNAP2	before	P	C57	before	2.27231	1.294978	1.75	0.0866	-0.34147	4.8861
P	CNTNAP2	after	P	CNTNAP2	before	-2.20724	1.314219	-1.68	0.1046	-4.90353	0.48906
ChABC	CNTNAP2	after	P	C57	after	1.9849	1.294978	1.53	0.1329	-0.62888	4.59868
ChABC	CNTNAP2	after	P	CNTNAP2	before	1.72819	1.294978	1.33	0.1893	-0.8856	4.34197
P	C57	after	P	CNTNAP2	after	1.95052	1.540481	1.27	0.2121	-1.15356	5.05461
P	C57	after	P	C57	before	1.36812	1.127219	1.21	0.2372	-0.96379	3.70002
P	C57	before	P	CNTNAP2	before	-1.62483	1.38439	-1.17	0.2472	-4.41908	1.16942
ChABC	CNTNAP2	after	ChABC	CNTNAP2	before	1.08071	0.976201	1.11	0.2797	-0.93878	3.1002
ChABC	C57	after	P	C57	before	-1.27519	1.294978	-0.98	0.3304	-3.88897	1.33859
ChABC	C57	before	P	C57	before	-1.13039	1.294978	-0.87	0.3877	-3.74417	1.48339
ChABC	CNTNAP2	before	P	C57	after	0.90419	1.294978	0.7	0.4889	-1.70959	3.51798
ChABC	CNTNAP2	before	P	CNTNAP2	before	0.64748	1.294978	0.5	0.6197	-1.9663	3.26126

ChABC	C57	after	P	CNTNAP2	after	-0.69279	1.460656	-0.47	0.6376	-3.63561	2.25004
P	C57	before	P	CNTNAP2	after	0.5824	1.540481	0.38	0.7072	-2.52168	3.68649
ChABC	C57	before	P	CNTNAP2	after	-0.54799	1.460656	-0.38	0.7093	-3.49081	2.39484
P	C57	after	P	CNTNAP2	before	-0.25671	1.38439	-0.19	0.8538	-3.05096	2.53754
ChABC	C57	after	ChABC	C57	before	-0.1448	0.976201	-0.15	0.8834	-2.16429	1.87469

trt	mouse	time					Least Squares Mean
ChABC	CNTNAP2	after	A				10.18686
ChABC	CNTNAP2	before	A	B			9.10615
P	CNTNAP2	before	A	B			8.45867
P	C57	after	A	B	C		8.201957
P	C57	before		B	C	D	6.833837
P	CNTNAP2	after		B	C	D	6.251433
ChABC	C57	before			C	D	5.703447
ChABC	C57	after				D	5.558645

S-FIG15B

Source	Nparm	DFNum	DFDen	F Ratio	Prob > F
trt	1	1	22	0.050921	0.8236
mouse	1	1	22	4.832711	0.0388
trt*mouse	1	1	22	0.005321	0.9425
time	1	1	22.1	2.129524	0.1586
trt*time	1	1	22.1	0.080886	0.7788
mouse*tim	1	1	22.1	0.467808	0.5011
trt*mouse*	1	1	22.1	0.819511	0.3751

trt	mouse	time	-trt	-mouse	-time	Difference	Std Error	t Ratio	Prob>|t|	Lower 95%	Upper 95%
ChABC	CNTNAP2	before	P	C57	after	4.8618	1.949866	2.49	0.0166	0.92862	8.79498
ChABC	C57	before	ChABC	CNTNAP2	before	-3.89032	1.949866	-2	0.0524	-7.8235	0.042857
P	C57	after	P	CNTNAP2	before	-3.91288	2.084495	-1.88	0.0673	-8.11762	0.29187
ChABC	C57	after	ChABC	CNTNAP2	before	-3.45029	1.949866	-1.77	0.084	-7.38347	0.482893
ChABC	CNTNAP2	after	ChABC	CNTNAP2	before	-2.93548	1.952019	-1.5	0.1461	-6.97114	1.100188
ChABC	CNTNAP2	before	P	C57	before	2.77499	1.949866	1.42	0.162	-1.15819	6.708166
ChABC	C57	before	P	CNTNAP2	before	-2.9414	2.084495	-1.41	0.1655	-7.14615	1.263346
ChABC	CNTNAP2	before	P	CNTNAP2	after	2.56569	1.949866	1.32	0.1953	-1.36749	6.498871
ChABC	C57	after	P	CNTNAP2	before	-2.50136	2.084495	-1.2	0.2368	-6.70611	1.703382
P	C57	after	P	CNTNAP2	after	-2.29611	2.084495	-1.1	0.2768	-6.50086	1.908638
ChABC	CNTNAP2	after	P	CNTNAP2	before	-1.98655	2.013363	-0.99	0.3294	-6.04761	2.074505
ChABC	CNTNAP2	after	P	C57	after	1.92632	2.013363	0.96	0.3441	-2.13473	5.987382
P	C57	after	P	C57	before	-2.08681	2.178295	-0.96	0.3486	-6.60709	2.433459
P	C57	before	P	CNTNAP2	before	-1.82606	2.084495	-0.88	0.3859	-6.03081	2.378683
P	CNTNAP2	after	P	CNTNAP2	before	-1.61677	2.178295	-0.74	0.4659	-6.13704	2.903505
ChABC	C57	after	P	C57	after	1.41151	2.084495	0.68	0.502	-2.79323	5.616259
ChABC	C57	before	P	CNTNAP2	after	-1.32463	2.084495	-0.64	0.5285	-5.52938	2.880114
ChABC	C57	before	P	C57	before	-1.11534	2.084495	-0.54	0.5954	-5.32008	3.089409
ChABC	CNTNAP2	before	P	CNTNAP2	before	0.94892	1.949866	0.49	0.629	-2.98426	4.882103
ChABC	C57	before	ChABC	CNTNAP2	after	-0.95485	2.013363	-0.47	0.6377	-5.01591	3.10621
ChABC	C57	before	P	C57	after	0.97148	2.084495	0.47	0.6436	-3.23327	5.176223
ChABC	C57	after	P	CNTNAP2	after	-0.8846	2.084495	-0.42	0.6734	-5.08934	3.32015

ChABC	C57	after	P	C57	before	-0.6753	2.084495	-0.32	0.7475	-4.88005	3.529445
ChABC	C57	after	ChABC	CNTNAP2	after	-0.51481	2.013363	-0.26	0.7994	-4.57587	3.546246
ChABC	C57	after	ChABC	C57	before	0.44004	2.178295	0.2	0.8418	-4.08024	4.960309
ChABC	CNTNAP2	after	P	CNTNAP2	after	-0.36978	2.013363	-0.18	0.8551	-4.43084	3.691273
P	C57	before	P	CNTNAP2	after	-0.2093	2.084495	-0.1	0.9205	-4.41404	3.995451
ChABC	CNTNAP2	after	P	C57	before	-0.16049	2.013363	-0.08	0.9368	-4.22155	3.900568

S-FIG11A

trt	mouse	time			Least Squares Mean
ChABC	CNTNAP2	before	A		10.73326
P	CNTNAP2	before	A	B	9.784336
P	CNTNAP2	after	A	B	8.167568
P	C57	before	A	B	7.958272
ChABC	CNTNAP2	after	A	B	7.797783
ChABC	C57	after	A	B	7.282971
ChABC	C57	before	A	B	6.842935
P	C57	after		B	5.871459

S-FIG15C

Source	Nparm	DFNum	DFDen	F Ratio	Prob > F
trt	1	1	24.5	1.222876	0.2795
mouse	1	1	24.5	8.59652	0.0072
trt*mouse	1	1	24.5	0.136222	0.7152
time	1	1	24.2	6.917212	0.0146
trt*time	1	1	24.2	1.93498	0.1769
mouse*tim	1	1	24.2	1.770422	0.1957
trt*mouse*	1	1	24.2	1.964204	0.1737

trt	mouse	time	-trt	-mouse	-time	Difference	Std Error	t Ratio	Prob>|t|	Lower 95%	Upper 95%
ChABC	C57	after	P	CNTNAP2	before	36.4111	8.71699	4.18	0.0001	18.8563	53.96582
ChABC	C57	before	P	CNTNAP2	before	30.8811	8.71699	3.54	0.0009	13.3263	48.43585
P	C57	after	P	CNTNAP2	before	32.8286	9.31885	3.52	0.001	14.0617	51.59542
P	C57	before	P	CNTNAP2	before	27.387	9.31885	2.94	0.0052	8.6201	46.1538
P	CNTNAP2	after	P	CNTNAP2	before	28.5086	9.89934	2.88	0.0076	8.2132	48.80394
ChABC	CNTNAP2	after	P	CNTNAP2	before	23.8032	8.71699	2.73	0.009	6.2484	41.35797
ChABC	C57	after	ChABC	CNTNAP2	before	17.539	8.07036	2.17	0.035	1.2864	33.79153
ChABC	CNTNAP2	before	P	CNTNAP2	before	18.8721	8.71699	2.16	0.0357	1.3173	36.42685
ChABC	CNTNAP2	before	P	C57	after	-13.9565	8.71699	-1.6	0.1163	-31.5113	3.59826
ChABC	C57	after	ChABC	CNTNAP2	after	12.6078	8.07036	1.56	0.1252	-3.6447	28.86041
ChABC	C57	before	ChABC	CNTNAP2	before	12.009	8.07036	1.49	0.1437	-4.2436	28.26156
ChABC	CNTNAP2	after	P	C57	after	-9.0254	8.71699	-1.04	0.306	-26.5801	8.52939
ChABC	C57	after	P	C57	before	9.0241	8.71699	1.04	0.3061	-8.5307	26.57885
ChABC	CNTNAP2	before	P	C57	before	-8.5149	8.71699	-0.98	0.3339	-26.0697	9.03988
ChABC	CNTNAP2	before	P	CNTNAP2	after	-9.6365	9.95623	-0.97	0.3382	-29.6801	10.40719
ChABC	C57	before	ChABC	CNTNAP2	after	7.0779	8.07036	0.88	0.3851	-9.1747	23.33043
ChABC	C57	after	P	CNTNAP2	after	7.9025	9.95623	0.79	0.4315	-12.1412	27.94616
ChABC	C57	after	ChABC	C57	before	5.53	7.49282	0.74	0.4681	-9.9818	21.04172
ChABC	CNTNAP2	after	ChABC	CNTNAP2	before	4.9311	7.49282	0.66	0.5171	-10.5806	20.44287
P	C57	after	P	C57	before	5.4416	8.65196	0.63	0.5357	-12.4698	23.35304
ChABC	CNTNAP2	after	P	CNTNAP2	after	-4.7053	9.95623	-0.47	0.6387	-24.749	15.33831
P	C57	after	P	CNTNAP2	after	4.32	10.48721	0.41	0.6823	-16.7931	25.43318

ChABC	CNTNAP2	after	P	C57	before	-3.5838	8.71699	-0.41	0.6829	-21.1385	13.971
ChABC	C57	after	P	C57	after	3.5825	8.71699	0.41	0.683	-13.9723	21.13724
ChABC	C57	before	P	C57	before	3.4941	8.71699	0.4	0.6904	-14.0607	21.04888
ChABC	C57	before	P	CNTNAP2	after	2.3725	9.95623	0.24	0.8127	-17.6711	22.41619
ChABC	C57	before	P	C57	after	-1.9475	8.71699	-0.22	0.8242	-19.5023	15.60726
P	C57	before	P	CNTNAP2	after	-1.1216	10.48721	-0.11	0.9153	-22.2347	19.99156

trt	mouse	time				Least Squares Mean
ChABC	C57	after	A			250.231
P	C57	after	A	B		246.6485
ChABC	C57	before	A	B		244.701
P	CNTNAP2	after	A	B		242.3285
P	C57	before	A	B		241.2069
ChABC	CNTNAP2	after	A	B		237.6231
ChABC	CNTNAP2	before		B		232.692
P	CNTNAP2	before			C	213.8199

S-FIG15D

Source	Nparm	DFNum	DFDen	F Ratio	Prob > F
trt	1	1	22	0.004459	0.9474
mouse	1	1	22	19.30707	0.0002
trt*mouse	1	1	22	3.917997	0.0604
time	1	1	22.1	22.40701	<.0001
trt*time	1	1	22.1	2.216784	0.1506
mouse*tim	1	1	22.1	0.472901	0.4988
trt*mouse*	1	1	22.1	3.371251	0.0798

trt	mouse	time	-trt	-mouse	-time	Difference	Std Error	t Ratio	Prob>|t|	Lower 95%	Upper 95%
ChABC	CNTNAP2	before	P	C57	after	-54.8472	9.44454	-5.81	<.0001	-73.9012	-35.7931
ChABC	C57	after	ChABC	CNTNAP2	before	46.3813	9.44454	4.91	<.0001	27.3272	65.4353
P	C57	after	P	CNTNAP2	before	43.573	10.09663	4.32	<.0001	23.2033	63.9426
P	C57	after	P	C57	before	45.5445	10.68321	4.26	0.0003	23.3771	67.7119
ChABC	C57	before	ChABC	CNTNAP2	before	35.4652	9.44454	3.76	0.0005	16.4111	54.5193
ChABC	C57	after	P	C57	before	37.0786	10.09663	3.67	0.0007	16.7089	57.4482
ChABC	C57	after	P	CNTNAP2	before	35.1071	10.09663	3.48	0.0012	14.7374	55.4767
ChABC	CNTNAP2	after	P	C57	after	-31.9708	9.75013	-3.28	0.0021	-51.6397	-12.3018
ChABC	CNTNAP2	before	P	CNTNAP2	after	-30.5345	9.44454	-3.23	0.0024	-49.5886	-11.4805
ChABC	C57	before	P	C57	before	26.1625	10.09663	2.59	0.0131	5.7929	46.5322
ChABC	C57	after	ChABC	CNTNAP2	after	23.5049	9.75013	2.41	0.0203	3.8359	43.1738
P	C57	after	P	CNTNAP2	after	24.3126	10.09663	2.41	0.0205	3.943	44.6823
ChABC	C57	before	P	CNTNAP2	before	24.191	10.09663	2.4	0.0211	3.8214	44.5607
ChABC	CNTNAP2	after	ChABC	CNTNAP2	before	22.8764	9.56368	2.39	0.0252	3.1053	42.6475
P	C57	before	P	CNTNAP2	after	-21.2319	10.09663	-2.1	0.0414	-41.6015	-0.8622
ChABC	C57	before	P	C57	after	-19.382	10.09663	-1.92	0.0616	-39.7516	0.9877
P	CNTNAP2	after	P	CNTNAP2	before	19.2604	10.68321	1.8	0.0853	-2.907	41.4277
ChABC	C57	after	P	CNTNAP2	after	15.8467	10.09663	1.57	0.124	-4.5229	36.2164
ChABC	CNTNAP2	after	P	C57	before	13.5737	9.75013	1.39	0.1711	-6.0952	33.2427
ChABC	C57	before	ChABC	CNTNAP2	after	12.5888	9.75013	1.29	0.2036	-7.0801	32.2577
ChABC	CNTNAP2	before	P	CNTNAP2	before	-11.2742	9.44454	-1.19	0.2392	-30.3283	7.7799

ChABC	CNTNAP2	after	P	CNTNAP2	before	11.6022	9.75013	1.19	0.2407	-8.0667	31.2712
ChABC	C57	after	ChABC	C57	before	10.9161	10.68321	1.02	0.3181	-11.2513	33.0835
ChABC	CNTNAP2	before	P	C57	before	-9.3027	9.44454	-0.98	0.3302	-28.3568	9.7514
ChABC	C57	after	P	C57	after	-8.4659	10.09663	-0.84	0.4065	-28.8356	11.9038
ChABC	CNTNAP2	after	P	CNTNAP2	after	-7.6581	9.75013	-0.79	0.4365	-27.3271	12.0108
ChABC	C57	before	P	CNTNAP2	after	4.9307	10.09663	0.49	0.6278	-15.439	25.3003
P	C57	before	P	CNTNAP2	before	-1.9715	10.09663	-0.2	0.8461	-22.3412	18.3982

trt	mouse	time							Least Squares Mean
P	C57	after	A						262.3653
ChABC	C57	after	A	B					253.8994
ChABC	C57	before	A	B	C				242.9833
P	CNTNAP2	after		B	C	D			238.0527
ChABC	CNTNAP2	after			C	D	E		230.3945
P	CNTNAP2	before				D	E	F	218.7923
P	C57	before					E	F	216.8208
ChABC	CNTNAP2	before						F	207.5181
