# Supplementary material for: Behavioral regulation by perineuronal nets in the prefrontal cortex of the CNTNAP2 mouse model of autism spectrum disorder
Source: Front Behav Neurosci. 2023 Mar 14;17:1114789. doi: 10.3389/fnbeh.2023.1114789 (PMC10043266; doi:10.3389/fnbeh.2023.1114789)
Supplement: Supplementary file 3 [file Data_Sheet_3.PDF]

Fixed Effect test **FIG. 4I**

Source	Nparm	DFNum	DFDen	F Ratio	Prob > F
Mouse	1	1	50.9	1.943456	0.1693
treatment	1	1	50.9	5.268608	0.0259
Mouse*trea	1	1	50.9	1.039325	0.3128
Time	1	1	50.2	3.393486	0.0714
Mouse*Tim	1	1	50.2	1.725657	0.1949
treatment*	1	1	50.2	0.435197	0.5125
Mouse*trea	1	1	50.2	5.021931	0.0295

Fisher LSD

**Mouse**	**treatment**	**Time**	**-Mouse**	**-treatment**	**-Time**	**Difference**	**Std Error**	**t Ratio**	**Prob>|t|**	**Lower 95%**	**Upper 95%**
**CNTNAP2**	**ChABC**	**After**	**CNTNAP2**	**ChABC**	**Before**	**8.02112**	**2.455798**	**3.27**	**0.0019**	**3.0918**	**12.9504**
**C57**	**ChABC**	**Before**	**CNTNAP2**	**ChABC**	**Before**	**8.50241**	**2.687367**	**3.16**	**0.0021**	**3.1661**	**13.8387**
C57	ChABC	After	CNTNAP2	ChABC	Before	7.10813	2.687367	2.65	0.0096	1.7718	12.4444
C57	ChABC	Before	C57	P	Before	7.30595	2.888833	2.53	0.0131	1.5696	13.0423
C57	P	Before	CNTNAP2	ChABC	After	-6.82466	2.889391	-2.36	0.0202	-12.5617	-1.0877
C57	ChABC	Before	CNTNAP2	P	Before	6.66679	2.888833	2.31	0.0232	0.9304	12.4031
CNTNAP2	ChABC	After	CNTNAP2	P	Before	6.18549	2.889391	2.14	0.0349	0.4485	11.9225
C57	ChABC	Before	CNTNAP2	P	After	6.3289	3.041398	2.08	0.0402	0.2904	12.3674
C57	ChABC	After	C57	P	Before	5.91167	2.888833	2.05	0.0435	0.1753	11.648
CNTNAP2	ChABC	After	CNTNAP2	P	After	5.84761	3.041928	1.92	0.0576	-0.1916	11.8868
C57	ChABC	After	CNTNAP2	P	Before	5.2725	2.888833	1.83	0.0712	-0.4638	11.0088
C57	ChABC	After	CNTNAP2	P	After	4.93462	3.041398	1.62	0.108	-1.1039	10.9731
C57	ChABC	Before	C57	P	After	4.51179	2.888833	1.56	0.1217	-1.2246	10.2481
C57	P	After	CNTNAP2	ChABC	Before	3.99063	2.804264	1.42	0.1581	-1.5778	9.559
C57	P	After	CNTNAP2	ChABC	After	-4.03049	2.889391	-1.39	0.1663	-9.7675	1.7065
C57	ChABC	After	C57	P	After	3.1175	2.888833	1.08	0.2833	-2.6188	8.8538
C57	P	After	C57	P	Before	2.79417	2.719376	1.03	0.3093	-2.6727	8.261
CNTNAP2	ChABC	Before	CNTNAP2	P	After	-2.17351	2.961189	-0.73	0.4648	-8.0527	3.7057
C57	P	After	CNTNAP2	P	Before	2.155	2.997884	0.72	0.474	-3.7979	8.1079
CNTNAP2	ChABC	Before	CNTNAP2	P	Before	-1.83563	2.804264	-0.65	0.5143	-7.404	3.7328
C57	P	After	CNTNAP2	P	After	1.81712	3.145164	0.58	0.5648	-4.4275	8.0617

C57	ChABC	After	C57	ChABC	Before	-1.39429	2.517653	-0.55	0.5823	-6.4556	3.6671
C57	P	Before	CNTNAP2	ChABC	Before	1.19646	2.804264	0.43	0.6706	-4.372	6.7649
C57	ChABC	After	CNTNAP2	ChABC	After	-0.91299	2.776082	-0.33	0.743	-6.425	4.599
C57	P	Before	CNTNAP2	P	After	-0.97705	3.145164	-0.31	0.7568	-7.2216	5.2675
C57	P	Before	CNTNAP2	P	Before	-0.63917	2.997884	-0.21	0.8316	-6.5921	5.3137
C57	ChABC	Before	CNTNAP2	ChABC	After	0.48129	2.776082	0.17	0.8627	-5.0307	5.9933
CNTNAP2	P	After	CNTNAP2	P	Before	0.33788	2.88093	0.12	0.9071	-5.4416	6.1174

All pairwise connecting letters

Mouse	treatment	Time					Least Squares Mean
C57	ChABC	Before	A				30.66429
CNTNAP2	ChABC	After	A	B			30.18299
C57	ChABC	After	A	B	C		29.27
C57	P	After	A	B	C	D	26.1525
CNTNAP2	P	After		B	C	D	24.33538
CNTNAP2	P	Before			C	D	23.9975
C57	P	Before				D	23.35833
CNTNAP2	ChABC	Before				D	22.16188

Fixed Effect test **FIG. 4J**

Source	Nparm	DFNum	DFDen	F Ratio	Prob > F
Mouse	1	1	50.8	0.024469	0.8763
treatment	1	1	50.8	5.019565	0.0295
Mouse*trea	1	1	50.8	0.00375	0.9514
Time	1	1	50.4	1.544842	0.2197
Mouse*Tim	1	1	50.4	6.974576	0.011
treatment*'	1	1	50.4	1.283879	0.2625
Mouse*trea	1	1	50.4	4.646484	0.0359

Fisher LSD

Mouse	treatment	Time	-Mouse	-treatment	-Time	Difference	Std Error	t Ratio	Prob>|t|	Lower 95%	Upper 95%
**CNTNAP2**	**ChABC**	**After**	**CNTNAP2**	**ChABC**	**Before**	**11.7301**	**3.08203**	**3.81**	**0.0004**	**5.5485**	**17.9117**
CNTNAP2	ChABC	After	CNTNAP2	P	Before	10.4093	3.426489	3.04	0.0031	3.6065	17.212
C57	P	After	CNTNAP2	ChABC	After	-9.846	3.426489	-2.87	0.005	-16.6487	-3.0432
C57	P	Before	CNTNAP2	ChABC	After	-9.2318	3.426489	-2.69	0.0083	-16.0345	-2.429
CNTNAP2	ChABC	After	CNTNAP2	P	After	9.4359	3.605511	2.62	0.0103	2.2778	16.5939
C57	ChABC	Before	CNTNAP2	ChABC	Before	8.0121	3.127604	2.56	0.012	1.8026	14.2217
C57	ChABC	After	CNTNAP2	ChABC	After	-7.6766	3.297169	-2.33	0.022	-14.2225	-1.1306
**C57**	**ChABC**	**Before**	**CNTNAP2**	**P**	**Before**	**6.6913**	**3.362073**	**1.99**	**0.0495**	**0.0163**	**13.3663**
C57	ChABC	Before	C57	P	After	6.128	3.362073	1.82	0.0715	-0.547	12.803
C57	ChABC	Before	C57	P	Before	5.5138	3.362073	1.64	0.1043	-1.1612	12.1888
C57	ChABC	Before	CNTNAP2	P	After	5.7179	3.54435	1.61	0.11	-1.3188	12.7546
C57	ChABC	After	CNTNAP2	ChABC	Before	4.0536	3.127604	1.3	0.1981	-2.1559	10.2631
C57	ChABC	After	C57	ChABC	Before	-3.9586	3.100137	-1.28	0.2078	-10.1918	2.2747
C57	ChABC	Before	CNTNAP2	ChABC	After	-3.718	3.297169	-1.13	0.2623	-10.264	2.828
C57	ChABC	After	CNTNAP2	P	Before	2.7327	3.362073	0.81	0.4184	-3.9423	9.4078
C57	P	Before	CNTNAP2	ChABC	Before	2.4983	3.263649	0.77	0.4459	-3.9813	8.978
CNTNAP2	ChABC	Before	CNTNAP2	P	After	-2.2942	3.451129	-0.66	0.5078	-9.1459	4.5574
C57	ChABC	After	C57	P	After	2.1694	3.362073	0.65	0.5203	-4.5056	8.8444
C57	P	After	CNTNAP2	ChABC	Before	1.8842	3.263649	0.58	0.5651	-4.5955	8.3638
C57	ChABC	After	CNTNAP2	P	After	1.7593	3.54435	0.5	0.6208	-5.2774	8.7961

C57	ChABC	After	C57	P	Before	1.5552	3.362073	0.46	0.6447	-5.1198	8.2303
CNTNAP2	ChABC	Before	CNTNAP2	P	Before	-1.3208	3.263649	-0.4	0.6866	-7.8005	5.1588
C57	P	Before	CNTNAP2	P	Before	1.1775	3.488988	0.34	0.7365	-5.7495	8.1045
CNTNAP2	P	After	CNTNAP2	P	Before	0.9734	3.531506	0.28	0.7839	-6.1117	8.0585
C57	P	After	C57	P	Before	-0.6142	3.34853	-0.18	0.8552	-7.3468	6.1185
C57	P	After	CNTNAP2	P	Before	0.5633	3.488988	0.16	0.8721	-6.3637	7.4903
C57	P	After	CNTNAP2	P	After	-0.4101	3.664958	-0.11	0.9111	-7.6863	6.8661
C57	P	Before	CNTNAP2	P	After	0.2041	3.664958	0.06	0.9557	-7.0721	7.4803

All pairwise connecting letters

Mouse	treatment	Time				Least Squares Mean
CNTNAP2	ChABC	After	A			51.09012
C57	ChABC	Before	A	B		47.37214
C57	ChABC	After		B	C	43.41357
C57	P	Before		B	C	41.85833
CNTNAP2	P	After		B	C	41.65425
C57	P	After		B	C	41.24417
CNTNAP2	P	Before			C	40.68083
CNTNAP2	ChABC	Before			C	39.36

FIG. 5I

Source	Nparm	DFNum	DFDen	F Ratio	Prob > F
Mouse	1	1	49	0.064109	0.8012
treatment	1	1	49	1.685892	0.2002
Mouse*trea	1	1	49	0.006373	0.9367
Time	1	1	47.9	5.773527	0.0202
Mouse*Tim	1	1	47.9	0.358333	0.5523
treatment*	1	1	47.9	1.196494	0.2795
Mouse*trea	1	1	47.9	5.07743	0.0289

Mouse	treatment	Time	-Mouse	-treatment	-Time	Difference	Std Error	t Ratio	Prob>|t|	Lower 95%	Upper 95%
C57	ChABC	After	C57	ChABC	Before	5.88714	2.176288	2.71	0.0095	1.50767	10.26661
C57	ChABC	After	CNTNAP2	P	Before	7.01369	2.800211	2.5	0.0141	1.44765	12.57973
C57	ChABC	After	C57	P	After	6.05702	2.800211	2.16	0.0333	0.49098	11.62307
CNTNAP2	ChABC	After	CNTNAP2	P	Before	5.40338	2.753522	1.96	0.0529	-0.06904	10.8758
CNTNAP2	P	After	CNTNAP2	P	Before	4.7552	2.509562	1.89	0.0639	-0.28457	9.79498
C57	ChABC	Before	CNTNAP2	ChABC	After	-4.27683	2.641726	-1.62	0.1091	-9.527	0.97334
C57	P	After	CNTNAP2	ChABC	After	-4.44671	2.753522	-1.61	0.1099	-9.91913	1.02571
C57	ChABC	After	C57	P	Before	4.29369	2.800211	1.53	0.1288	-1.27235	9.85973
C57	ChABC	After	CNTNAP2	ChABC	Before	3.71536	2.604926	1.43	0.1574	-1.46251	8.89323
C57	P	After	CNTNAP2	P	After	-3.79854	3.035894	-1.25	0.2141	-9.83026	2.23319
C57	ChABC	Before	CNTNAP2	P	After	-3.62866	2.934874	-1.24	0.2195	-9.4595	2.20219
CNTNAP2	ChABC	Before	CNTNAP2	P	Before	3.29833	2.718236	1.21	0.2283	-2.10476	8.70143
CNTNAP2	ChABC	After	CNTNAP2	ChABC	Before	2.10504	2.082613	1.01	0.3172	-2.08272	6.29281
C57	P	Before	CNTNAP2	ChABC	After	-2.68338	2.753522	-0.97	0.3325	-8.1558	2.78904
C57	P	Before	CNTNAP2	P	Before	2.72	2.905917	0.94	0.3519	-3.05616	8.49616
C57	P	After	CNTNAP2	ChABC	Before	-2.34167	2.718236	-0.86	0.3914	-7.74476	3.06143
C57	ChABC	Before	CNTNAP2	ChABC	Before	-2.17179	2.604926	-0.83	0.4067	-7.34966	3.00608
C57	ChABC	After	CNTNAP2	P	After	2.25849	2.934874	0.77	0.4436	-3.57236	8.08933
C57	P	After	C57	P	Before	-1.76333	2.35066	-0.75	0.457	-6.4937	2.96703
C57	P	Before	CNTNAP2	P	After	-2.0352	3.035894	-0.67	0.5043	-8.06693	3.99652
C57	ChABC	After	CNTNAP2	ChABC	After	1.61031	2.641726	0.61	0.5437	-3.63985	6.86048
C57	ChABC	Before	C57	P	Before	-1.59345	2.800211	-0.57	0.5708	-7.15949	3.97259
CNTNAP2	ChABC	Before	CNTNAP2	P	After	-1.45687	2.856765	-0.51	0.6113	-7.13239	4.21865

C57	ChABC	Before	CNTNAP2	P	Before	1.12655	2.800211	0.4	0.6884	-4.43949	6.69259
C57	P	After	CNTNAP2	P	Before	0.95667	2.905917	0.33	0.7428	-4.81949	6.73282
CNTNAP2	ChABC	After	CNTNAP2	P	After	0.64817	2.89036	0.22	0.8231	-5.09352	6.38987
C57	P	Before	CNTNAP2	ChABC	Before	-0.57833	2.718236	-0.21	0.832	-5.98143	4.82476
C57	ChABC	Before	C57	P	After	0.16988	2.800211	0.06	0.9518	-5.39616	5.73592

Mouse	treatment	Time			Least Squares Mean
C57	ChABC	After	A		35.31786
CNTNAP2	ChABC	After	A	B	33.70754
CNTNAP2	P	After	A	B	33.05937
CNTNAP2	ChABC	Before	A	B	31.6025
C57	P	Before	A	B	31.02417
C57	ChABC	Before		B	29.43071
C57	P	After		B	29.26083
CNTNAP2	P	Before		B	28.30417

FIG. 5J

Source	Nparm	DFNum	DFDen	F Ratio	Prob > F
Mouse	1	1	49.4	0.706916	0.4045
treatment	1	1	49.4	0.163059	0.6881
Mouse*tre	1	1	49.4	0.023902	0.8778
Time	1	1	48.7	0.81462	0.3712
Mouse*Tim	1	1	48.7	0.219804	0.6413
treatment*	1	1	48.7	2.362106	0.1308
Mouse*tre	1	1	48.7	1.384047	0.2451

Mouse	treatment	Time	-Mouse	-treatment	-Time	Difference	Std Error	t Ratio	Prob>|t|	Lower 95%	Upper 95%
C57	P	After	CNTNAP2	ChABC	Before	-5.185	3.120673	-1.66	0.1	-11.3822	1.01221
C57	P	After	C57	P	Before	-4.51333	2.95632	-1.53	0.1335	-10.4603	1.4336
C57	P	After	CNTNAP2	P	Before	-4.71333	3.336139	-1.41	0.1611	-11.3384	1.91176
C57	ChABC	Before	CNTNAP2	ChABC	Before	-4.12179	2.990587	-1.38	0.1714	-10.0607	1.81709
C57	ChABC	After	C57	P	After	4.3325	3.214784	1.35	0.181	-2.0516	10.7166
C57	ChABC	After	C57	ChABC	Before	3.26929	2.737021	1.19	0.2383	-2.2365	8.77508
C57	P	After	CNTNAP2	ChABC	After	-3.73519	3.164635	-1.18	0.2409	-10.0193	2.54894
C57	ChABC	Before	CNTNAP2	P	Before	-3.65012	3.214784	-1.14	0.2591	-10.0342	2.73398
C57	ChABC	Before	C57	P	Before	-3.45012	3.214784	-1.07	0.286	-9.8342	2.93398
CNTNAP2	ChABC	Before	CNTNAP2	P	After	2.95536	3.293004	0.9	0.3718	-3.5827	9.49344
C57	ChABC	Before	CNTNAP2	ChABC	After	-2.67198	3.036434	-0.88	0.3811	-8.7015	3.35755
CNTNAP2	P	After	CNTNAP2	P	Before	-2.48369	3.137691	-0.79	0.4323	-8.7817	3.81429
C57	P	Before	CNTNAP2	P	After	2.28369	3.497871	0.65	0.5154	-4.6613	9.22867
C57	P	After	CNTNAP2	P	After	-2.22964	3.497871	-0.64	0.5254	-9.1746	4.71534
C57	ChABC	After	CNTNAP2	P	After	2.10286	3.382324	0.62	0.5356	-4.6126	8.81834
CNTNAP2	ChABC	After	CNTNAP2	ChABC	Before	-1.44981	2.613655	-0.55	0.5816	-6.7032	3.80357
CNTNAP2	ChABC	After	CNTNAP2	P	After	1.50555	3.334696	0.45	0.6527	-5.115	8.12614
C57	ChABC	Before	CNTNAP2	P	After	-1.16642	3.382324	-0.34	0.731	-7.8819	5.54906
C57	ChABC	Before	C57	P	After	1.06321	3.214784	0.33	0.7416	-5.3209	7.44732
CNTNAP2	ChABC	After	CNTNAP2	P	Before	-0.97814	3.164635	-0.31	0.7579	-7.2623	5.30599
C57	ChABC	After	CNTNAP2	ChABC	Before	-0.8525	2.990587	-0.29	0.7762	-6.7914	5.08638

C57	P	Before	CNTNAP2	ChABC	After	0.77814	3.164635	0.25	0.8063	-5.506	7.06227
C57	P	Before	CNTNAP2	ChABC	Before	-0.67167	3.120673	-0.22	0.8301	-6.8689	5.52554
C57	ChABC	After	CNTNAP2	ChABC	After	0.59731	3.036434	0.2	0.8445	-5.4322	6.62683
CNTNAP2	ChABC	Before	CNTNAP2	P	Before	0.47167	3.120673	0.15	0.8802	-5.7255	6.66888
C57	ChABC	After	CNTNAP2	P	Before	-0.38083	3.214784	-0.12	0.906	-6.7649	6.00327
C57	P	Before	CNTNAP2	P	Before	-0.2	3.336139	-0.06	0.9523	-6.8251	6.4251
C57	ChABC	After	C57	P	Before	-0.18083	3.214784	-0.06	0.9553	-6.5649	6.20327

Mouse	treatment	Time		Least Squares Mean
CNTNAP2	ChABC	Before	A	49.7025
CNTNAP2	P	Before	A	49.23083
C57	P	Before	A	49.03083
C57	ChABC	After	A	48.85
CNTNAP2	ChABC	After	A	48.25269
CNTNAP2	P	After	A	46.74714
C57	ChABC	Before	A	45.58071
C57	P	After	A	44.5175

S-FIG-16A

Source	Nparm	DFNum	DFDen	F Ratio	Prob > F
Mouse	1	1	24	1.062007	0.313
treatment	1	1	24	12.77301	0.0015
Mouse*trea	1	1	24	0.001436	0.9701
Time	1	1	23.7	5.333835	0.03
Mouse*Tim	1	1	23.7	2.244966	0.1473
treatment*	1	1	23.7	0.838236	0.3691
Mouse*trea	1	1	23.7	4.71164	0.0402

Mouse	treatment	Time	-Mouse	-treatment	-Time	Difference	Std Error	t Ratio	Prob>|t|	Lower 95%	Upper 95%
CNTNAP2	ChABC	After	CNTNAP2	P	Before	15.0421	3.967561	3.79	**0.0004**	7.0514	23.0328
CNTNAP2	ChABC	After	CNTNAP2	ChABC	Before	13.2013	3.397462	3.89	0.0008	6.158	20.2445
C57	P	Before	CNTNAP2	ChABC	After	-14.1538	3.967561	-3.57	0.0009	-22.1444	-6.1631
CNTNAP2	ChABC	After	CNTNAP2	P	After	13.6602	4.532547	3.01	0.0042	4.5352	22.7852
C57	ChABC	Before	CNTNAP2	P	Before	11.2083	3.967561	2.82	**0.007**	3.2177	19.199
C57	ChABC	After	CNTNAP2	P	Before	10.3583	3.967561	2.61	0.0122	2.3677	18.349
C57	ChABC	Before	C57	P	Before	10.32	3.967561	2.6	0.0125	2.3293	18.3107
C57	P	After	CNTNAP2	ChABC	After	-10.1971	3.967561	-2.57	0.0135	-18.1878	-2.2064
C57	ChABC	Before	CNTNAP2	ChABC	Before	9.3675	3.673248	2.55	0.0142	1.9696	16.7654
C57	ChABC	After	C57	P	Before	9.47	3.967561	2.39	0.0212	1.4793	17.4607
C57	ChABC	After	CNTNAP2	ChABC	Before	8.5175	3.673248	2.32	0.025	1.1196	15.9154
C57	ChABC	Before	CNTNAP2	P	After	9.8265	4.532547	2.17	0.0354	0.7014	18.9515
C57	ChABC	After	CNTNAP2	P	After	8.9765	4.532547	1.98	0.0537	-0.1486	18.1015
C57	ChABC	Before	C57	P	After	6.3633	3.967561	1.6	0.1157	-1.6273	14.354
C57	ChABC	After	C57	P	After	5.5133	3.967561	1.39	0.1715	-2.4773	13.504
C57	ChABC	After	CNTNAP2	ChABC	After	-4.6838	3.673248	-1.28	0.2088	-12.0817	2.7142
C57	P	After	CNTNAP2	P	Before	4.845	4.241501	1.14	0.2594	-3.6974	13.3874
C57	ChABC	Before	CNTNAP2	ChABC	After	-3.8338	3.673248	-1.04	0.3022	-11.2317	3.5642
C57	P	After	C57	P	Before	3.9567	3.923051	1.01	0.3241	-4.1761	12.0895
C57	P	After	CNTNAP2	ChABC	Before	3.0042	3.967561	0.76	0.4529	-4.9865	10.9948
C57	P	After	CNTNAP2	P	After	3.4631	4.774178	0.73	0.4719	-6.1486	13.0749

CNTNAP2	ChABC	Before	CNTNAP2	P	Before	1.8408	3.967561	0.46	0.6449	-6.1498	9.8315
CNTNAP2	P	After	CNTNAP2	P	Before	1.3819	4.493637	0.31	0.7608	-7.8395	10.6032
C57	ChABC	After	C57	ChABC	Before	-0.85	3.397462	-0.25	0.8047	-7.8932	6.1932
C57	P	Before	CNTNAP2	ChABC	Before	-0.9525	3.967561	-0.24	0.8114	-8.9432	7.0382
C57	P	Before	CNTNAP2	P	Before	0.8883	4.241501	0.21	0.835	-7.6541	9.4307
C57	P	Before	CNTNAP2	P	After	-0.4935	4.774178	-0.1	0.9181	-10.1053	9.1182
CNTNAP2	ChABC	Before	CNTNAP2	P	After	0.459	4.532547	0.1	0.9198	-8.6661	9.584

Mouse	treatment	Time					Least Squares Mean
CNTNAP2	ChABC	After	A				36.61375
C57	ChABC	Before	A	B			32.78
C57	ChABC	After	A	B	C		31.93
C57	P	After		B	C	D	26.41667
CNTNAP2	ChABC	Before				D	23.4125
CNTNAP2	P	After			C	D	22.95354
C57	P	Before				D	22.46
CNTNAP2	P	Before				D	21.57167

S-FIG-16B

Source	Nparm	DFNum	DFDen	F Ratio	Prob > F
Mouse	1	1	23.3	2.012657	0.1692
treatment	1	1	23.3	0.773612	0.3881
Mouse*trea	1	1	23.3	3.128799	0.09
Time	1	1	22.6	0.195086	0.6629
Mouse*Tim	1	1	22.6	0.081581	0.7778
treatment*	1	1	22.6	0.161682	0.6914
Mouse*trea	1	1	22.6	0.63499	0.4338

Mouse	treatment	Time	-Mouse	-treatment	-Time	Difference	Std Error	t Ratio	Prob>|t|	Lower 95%	Upper 95%
C57	ChABC	Before	CNTNAP2	ChABC	After	7.45236	3.464167	2.15	0.0373	0.4593	14.44543
C57	ChABC	Before	CNTNAP2	ChABC	Before	6.93208	3.359111	2.06	0.0454	0.1491	13.71509
CNTNAP2	ChABC	After	CNTNAP2	P	Before	-6.23295	3.472531	-1.79	0.0798	-13.2394	0.77348
CNTNAP2	ChABC	Before	CNTNAP2	P	Before	-5.71267	3.367736	-1.7	0.0972	-12.5089	1.0836
C57	P	After	CNTNAP2	ChABC	After	5.49736	3.464167	1.59	0.1201	-1.4957	12.49043
C57	ChABC	After	CNTNAP2	ChABC	After	5.33236	3.464167	1.54	0.1313	-1.6607	12.32543
C57	P	After	CNTNAP2	ChABC	Before	4.97708	3.359111	1.48	0.146	-1.8059	11.76009
C57	ChABC	After	CNTNAP2	ChABC	Before	4.81208	3.359111	1.43	0.1595	-1.9709	11.59509
CNTNAP2	ChABC	After	CNTNAP2	P	After	-4.47741	3.472531	-1.29	0.2043	-11.4838	2.52903
CNTNAP2	ChABC	Before	CNTNAP2	P	After	-3.95712	3.367736	-1.18	0.2466	-10.7534	2.83914
C57	P	Before	CNTNAP2	ChABC	After	3.8657	3.464167	1.12	0.2709	-3.1274	10.85876
C57	ChABC	Before	C57	P	Before	3.58667	3.591041	1	0.3237	-3.6647	10.83801
C57	P	Before	CNTNAP2	ChABC	Before	3.34542	3.359111	1	0.3251	-3.4376	10.12842
C57	ChABC	Before	CNTNAP2	P	After	2.97496	3.59911	0.83	0.4132	-4.2887	10.23863
C57	ChABC	After	C57	ChABC	Before	-2.12	3.153867	-0.67	0.5091	-8.6952	4.45523
C57	P	Before	CNTNAP2	P	Before	-2.36725	3.59911	-0.66	0.5143	-9.6309	4.89642
C57	ChABC	Before	C57	P	After	1.955	3.591041	0.54	0.5891	-5.2963	9.20634
CNTNAP2	P	After	CNTNAP2	P	Before	-1.75554	3.330824	-0.53	0.6022	-8.5718	5.06074
C57	P	After	C57	P	Before	1.63167	3.153867	0.52	0.6105	-4.9436	8.2069
C57	ChABC	After	C57	P	Before	1.46667	3.591041	0.41	0.6851	-5.7847	8.71801
C57	ChABC	Before	CNTNAP2	P	Before	1.21942	3.59911	0.34	0.7364	-6.0443	8.48309

C57	P	After	CNTNAP2	P	After	1.01996	3.59911	0.28	0.7783	-6.2437	8.28363
C57	ChABC	After	CNTNAP2	P	Before	-0.90058	3.59911	-0.25	0.8036	-8.1643	6.36309
C57	ChABC	After	CNTNAP2	P	After	0.85496	3.59911	0.24	0.8134	-6.4087	8.11863
C57	P	After	CNTNAP2	P	Before	-0.73558	3.59911	-0.2	0.839	-7.9993	6.52809
CNTNAP2	ChABC	After	CNTNAP2	ChABC	Before	-0.52028	2.859542	-0.18	0.8573	-6.4583	5.41776
C57	P	Before	CNTNAP2	P	After	-0.61171	3.59911	-0.17	0.8659	-7.8754	6.65196
C57	ChABC	After	C57	P	After	-0.165	3.591041	-0.05	0.9636	-7.4163	7.08634

Mouse	treatment	Time			Least Squares Mean
C57	ChABC	Before	A		27.84333
CNTNAP2	P	Before	A	B	26.62392
C57	P	After	A	B	25.88833
C57	ChABC	After	A	B	25.72333
CNTNAP2	P	After	A	B	24.86837
C57	P	Before	A	B	24.25667
CNTNAP2	ChABC	Before		B	20.91125
CNTNAP2	ChABC	After		B	20.39097

Fixed Effect test

S-FIG-17A

Source	Nparm	DFNum	DFDen	F Ratio	Prob > F
Mouse	1	1	23.7	0.550095	0.4656
treatment	1	1	23.7	7.185678	0.0132
Mouse*trea	1	1	23.7	0.051152	0.823
Time	1	1	23.3	2.561727	0.123
Mouse*Tim	1	1	23.3	8.614738	0.0074
treatment*	1	1	23.3	1.172079	0.2901
Mouse*trea	1	1	23.3	3.411251	0.0775

Mouse	treatment	Time	-Mouse	-treatment	-Time	Difference	Std Error	t Ratio	Prob>|t|	Lower 95%	Upper 95%
**CNTNAP2**	**ChABC**	**After**	**CNTNAP2**	**ChABC**	**Before**	**16.8013**	**3.988696**	**4.21**	**0.0004**	**8.5239**	**25.0786**
CNTNAP2	ChABC	After	CNTNAP2	P	Before	17.9046	4.73845	3.78	0.0005	8.3588	27.4504
C57	P	After	CNTNAP2	ChABC	After	-14.1763	4.73845	-2.99	0.0045	-23.722	-4.6305
C57	ChABC	Before	CNTNAP2	P	Before	13.2308	4.73845	2.79	0.0077	3.6851	22.7766
**C57**	**ChABC**	**Before**	**CNTNAP2**	**ChABC**	**Before**	**12.1275**	**4.386952**	**2.76**	**0.0083**	**3.2898**	**20.9652**
C57	P	Before	CNTNAP2	ChABC	After	-12.8929	4.73845	-2.72	0.0092	-22.4387	-3.3471
CNTNAP2	ChABC	After	CNTNAP2	P	After	14.29	5.40845	2.64	0.0112	3.4007	25.1793
**C57**	**ChABC**	**After**	**CNTNAP2**	**ChABC**	**After**	**-9.3975**	**4.386952**	**-2.14**	**0.0377**	**-18.2352**	**-0.5598**
C57	ChABC	Before	C57	P	After	9.5025	4.73845	2.01	0.051	-0.0433	19.0483
C57	ChABC	After	CNTNAP2	P	Before	8.5071	4.73845	1.8	0.0794	-1.0387	18.0529
C57	ChABC	Before	CNTNAP2	P	After	9.6163	5.40845	1.78	0.0821	-1.273	20.5056
C57	ChABC	Before	C57	P	Before	8.2192	4.73845	1.73	0.0897	-1.3266	17.7649
C57	ChABC	After	CNTNAP2	ChABC	Before	7.4038	4.386952	1.69	0.0984	-1.4339	16.2414
C57	ChABC	After	C57	ChABC	Before	-4.7237	3.988696	-1.18	0.2491	-13.0011	3.5536
C57	ChABC	Before	CNTNAP2	ChABC	After	-4.6737	4.386952	-1.07	0.2924	-13.5114	4.1639
C57	ChABC	After	C57	P	After	4.7788	4.73845	1.01	0.3186	-4.767	14.3245
C57	P	Before	CNTNAP2	P	Before	5.0117	5.065616	0.99	0.3278	-5.1932	15.2165
C57	ChABC	After	CNTNAP2	P	After	4.8925	5.40845	0.9	0.3704	-5.9968	15.7818
C57	P	Before	CNTNAP2	ChABC	Before	3.9083	4.73845	0.82	0.4139	-5.6374	13.4541
C57	ChABC	After	C57	P	Before	3.4954	4.73845	0.74	0.4646	-6.0504	13.0412

C57	P	After	CNTNAP2	P	Before	3.7283	5.065616	0.74	0.4656	-6.4765	13.9332
CNTNAP2	P	After	CNTNAP2	P	Before	3.6146	5.292575	0.68	0.5006	-7.2552	14.4843
C57	P	After	CNTNAP2	ChABC	Before	2.625	4.73845	0.55	0.5824	-6.9208	12.1708
CNTNAP2	ChABC	Before	CNTNAP2	P	After	-2.5112	5.40845	-0.46	0.6446	-13.4005	8.3781
C57	P	After	C57	P	Before	-1.2833	4.605749	-0.28	0.7832	-10.8412	8.2746
C57	P	Before	CNTNAP2	P	After	1.3971	5.69727	0.25	0.8074	-10.0742	12.8684
CNTNAP2	ChABC	Before	CNTNAP2	P	Before	1.1033	4.73845	0.23	0.8169	-8.4424	10.6491
C57	P	After	CNTNAP2	P	After	0.1138	5.69727	0.02	0.9842	-11.3575	11.585

Mouse	treatment	Time				Least Squares Mean
CNTNAP2	ChABC	After	A			55.42125
C57	ChABC	Before	A	B		50.7475
C57	ChABC	After		B	C	46.02375
C57	P	Before		B	C	42.52833
C57	P	After		B	C	41.245
CNTNAP2	P	After		B	C	41.13124
CNTNAP2	ChABC	Before			C	38.62
CNTNAP2	P	Before			C	37.51667

S-FIG-17B

Source	Nparm	DFNum	DFDen	F Ratio	Prob > F
Mouse	1	1	22.8	0.001151	0.9732
treatment	1	1	22.8	0.295052	0.5923
Mouse*trea	1	1	22.8	0.385991	0.5406
Time	1	1	22.5	0.345981	0.5623
Mouse*Tim	1	1	22.5	0.00411	0.9495
treatment*	1	1	22.5	0.014991	0.9036
Mouse*trea	1	1	22.5	0.307755	0.5845

Mouse	treatment	Time	-Mouse	-treatment	-Time	Difference	Std Error	t Ratio	Prob>|t|	Lower 95%	Upper 95%
CNTNAP2	ChABC	After	CNTNAP2	P	Before	-3.94166	4.571796	-0.86	0.3934	-13.1622	5.27891
CNTNAP2	ChABC	Before	CNTNAP2	P	Before	-3.73882	4.427619	-0.84	0.4031	-12.6689	5.19123
C57	ChABC	After	CNTNAP2	P	Before	-3.90549	4.729813	-0.83	0.4135	-13.4451	5.63415
C57	ChABC	Before	CNTNAP2	ChABC	After	2.9745	4.546289	0.65	0.5164	-6.1953	12.14434
C57	ChABC	After	C57	ChABC	Before	-2.93833	4.492835	-0.65	0.5205	-12.3056	6.42898
C57	ChABC	Before	CNTNAP2	ChABC	Before	2.77167	4.401276	0.63	0.5322	-6.1061	11.64944
C57	P	Before	CNTNAP2	P	Before	-2.65049	4.729813	-0.56	0.5781	-12.1901	6.88915
C57	P	After	CNTNAP2	P	Before	-2.59549	4.729813	-0.55	0.586	-12.1351	6.94415
CNTNAP2	P	After	CNTNAP2	P	Before	-2.11377	4.620055	-0.46	0.6507	-11.567	7.33942
CNTNAP2	ChABC	After	CNTNAP2	P	After	-1.82789	4.571796	-0.4	0.6913	-11.0485	7.39268
C57	ChABC	After	CNTNAP2	P	After	-1.79172	4.729813	-0.38	0.7067	-11.3314	7.74792
CNTNAP2	ChABC	Before	CNTNAP2	P	After	-1.62505	4.427619	-0.37	0.7154	-10.5551	7.305
C57	ChABC	Before	C57	P	Before	1.68333	4.705162	0.36	0.7223	-7.8074	11.17407
C57	ChABC	Before	C57	P	After	1.62833	4.705162	0.35	0.731	-7.8624	11.11907
C57	P	After	CNTNAP2	ChABC	After	1.34617	4.546289	0.3	0.7686	-7.8237	10.51601
C57	P	Before	CNTNAP2	ChABC	After	1.29117	4.546289	0.28	0.7778	-7.8787	10.46101
C57	ChABC	After	C57	P	After	-1.31	4.705162	-0.28	0.782	-10.8007	8.18074
C57	ChABC	After	C57	P	Before	-1.255	4.705162	-0.27	0.791	-10.7457	8.23574
C57	P	After	CNTNAP2	ChABC	Before	1.14333	4.401276	0.26	0.7963	-7.7344	10.02111
C57	P	Before	CNTNAP2	ChABC	Before	1.08833	4.401276	0.25	0.8059	-7.7894	9.96611
C57	ChABC	Before	CNTNAP2	P	After	1.14661	4.729813	0.24	0.8096	-8.393	10.68625
C57	ChABC	Before	CNTNAP2	P	Before	-0.96716	4.729813	-0.2	0.8389	-10.5068	8.57248

C57	P	Before	CNTNAP2	P	After	-0.53672	4.729813	-0.11	0.9102	-10.0764	9.00292
C57	P	After	CNTNAP2	P	After	-0.48172	4.729813	-0.1	0.9194	-10.0214	9.05792
CNTNAP2	ChABC	After	CNTNAP2	ChABC	Before	-0.20283	4.054218	-0.05	0.9606	-8.6198	8.21415
C57	ChABC	After	CNTNAP2	ChABC	Before	-0.16667	4.401276	-0.04	0.97	-9.0444	8.71111
C57	P	After	C57	P	Before	0.055	4.492835	0.01	0.9904	-9.3123	9.42231
C57	ChABC	After	CNTNAP2	ChABC	After	0.03617	4.546289	0.01	0.9937	-9.1337	9.20601

Mouse	treatment	Time		Least Squares Mean
CNTNAP2	P	Before	A	43.83882
C57	ChABC	Before	A	42.87167
CNTNAP2	P	After	A	41.72505
C57	P	After	A	41.24333
C57	P	Before	A	41.18833
CNTNAP2	ChABC	Before	A	40.1
C57	ChABC	After	A	39.93333
CNTNAP2	ChABC	After	A	39.89717

S-FIG-18A

Source	Nparm	DFNum	DFDen	F Ratio	Prob > F
Mouse	1	1	49.3	0.25009	0.6192
treatment	1	1	49.3	2.865106	0.0968
Mouse*trea	1	1	49.3	0.000721	0.9787
Time	1	1	49	0.233768	0.6309
Mouse*Tim	1	1	49	2.994657	0.0898
treatment*	1	1	49	0.118327	0.7323
Mouse*trea	1	1	49	1.011845	0.3194

Mouse	treatment	Time	-Mouse	-treatment	-Time	Difference	Std Error	t Ratio	Prob>|t|	Lower 95%	Upper 95%
C57	ChABC	Before	CNTNAP2	P	Before	-3.46429	1.618194	-2.14	0.0348	-6.67617	-0.25241
C57	ChABC	Before	C57	P	After	-3.13095	1.618194	-1.93	0.0559	-6.34283	0.08093
CNTNAP2	ChABC	Before	CNTNAP2	P	Before	-2.5	1.570822	-1.59	0.1148	-5.61785	0.61785
C57	P	Before	CNTNAP2	P	Before	-2.58333	1.67928	-1.54	0.1272	-5.91646	0.74979
CNTNAP2	ChABC	After	CNTNAP2	P	Before	-2.41951	1.593926	-1.52	0.1323	-5.5832	0.74418
C57	ChABC	After	CNTNAP2	P	Before	-2.25	1.618194	-1.39	0.1676	-5.46188	0.96188
C57	P	After	C57	P	Before	2.25	1.616674	1.39	0.1705	-1.00158	5.50158
C57	P	After	CNTNAP2	ChABC	Before	2.16667	1.570822	1.38	0.171	-0.95119	5.28452
C57	P	After	CNTNAP2	ChABC	After	2.08618	1.593926	1.31	0.1937	-1.07751	5.24987
CNTNAP2	P	After	CNTNAP2	P	Before	-2.03171	1.704729	-1.19	0.2388	-5.45255	1.38914
C57	ChABC	After	C57	P	After	-1.91667	1.618194	-1.18	0.2391	-5.12855	1.29521
C57	P	After	CNTNAP2	P	After	1.69837	1.764212	0.96	0.3381	-1.80325	5.2
C57	ChABC	Before	CNTNAP2	P	After	-1.43258	1.706171	-0.84	0.4032	-4.819	1.95384
C57	ChABC	After	C57	ChABC	Before	1.21429	1.496749	0.81	0.4213	-1.79609	4.22466
C57	ChABC	Before	CNTNAP2	ChABC	After	-1.04477	1.529435	-0.68	0.4962	-4.08046	1.99091
C57	ChABC	Before	CNTNAP2	ChABC	Before	-0.96429	1.505342	-0.64	0.5233	-3.95217	2.0236
C57	ChABC	Before	C57	P	Before	-0.88095	1.618194	-0.54	0.5874	-4.09283	2.33093
C57	P	Before	CNTNAP2	P	After	-0.55163	1.764212	-0.31	0.7552	-4.05325	2.95
CNTNAP2	ChABC	Before	CNTNAP2	P	After	-0.46829	1.661309	-0.28	0.7786	-3.76567	2.82908
CNTNAP2	ChABC	After	CNTNAP2	P	After	-0.3878	1.683171	-0.23	0.8183	-3.72855	2.95295
C57	ChABC	After	C57	P	Before	0.33333	1.618194	0.21	0.8372	-2.87855	3.54521
C57	P	After	CNTNAP2	P	Before	-0.33333	1.67928	-0.2	0.8431	-3.66646	2.99979

C57	ChABC	After	CNTNAP2	ChABC	Before	0.25	1.505342	0.17	0.8684	-2.73789	3.23789
C57	ChABC	After	CNTNAP2	P	After	-0.21829	1.706171	-0.13	0.8985	-3.60471	3.16813
C57	ChABC	After	CNTNAP2	ChABC	After	0.16951	1.529435	0.11	0.912	-2.86617	3.20519
C57	P	Before	CNTNAP2	ChABC	After	-0.16382	1.593926	-0.1	0.9184	-3.32751	2.99987
CNTNAP2	ChABC	After	CNTNAP2	ChABC	Before	0.08049	1.425953	0.06	0.9552	-2.78509	2.94607
C57	P	Before	CNTNAP2	ChABC	Before	-0.08333	1.570822	-0.05	0.9578	-3.20119	3.03452

Mouse	treatment	Time			Least Squares Mean
CNTNAP2	P	Before	A		15.25
C57	P	After	A	B	14.91667
CNTNAP2	P	After	A	B	13.21829
C57	ChABC	After	A	B	13
CNTNAP2	ChABC	After	A	B	12.83049
CNTNAP2	ChABC	Before	A	B	12.75
C57	P	Before	A	B	12.66667
C57	ChABC	Before		B	11.78571

S-FIG-18B

Source	Nparm	DFNum	DFDen	F Ratio	Prob > F
Mouse	1	1	24.1	4.191649	0.0517
treatment	1	1	24.1	2.066678	0.1634
Mouse*trea	1	1	24.1	0.015166	0.903
Time	1	1	24.4	0.373685	0.5467
Mouse*Tim	1	1	24.4	1.96124	0.174
treatment*	1	1	24.4	2.724677	0.1116
Mouse*trea	1	1	24.4	0.060318	0.8081

Mouse	treatment	Time	-Mouse	-treatment	-Time	Difference	Std Error	t Ratio	Prob>|t|	Lower 95%	Upper 95%
C57	P	Before	CNTNAP2	ChABC	After	3.79167	1.984889	1.91	0.0624	-0.205	7.788333
C57	P	Before	CNTNAP2	P	After	4.56333	2.390022	1.91	0.0625	-0.24807	9.374729
C57	P	Before	CNTNAP2	ChABC	Before	3.66667	1.984889	1.85	0.0712	-0.33	7.663333
CNTNAP2	ChABC	After	CNTNAP2	P	Before	-3.45833	1.984889	-1.74	0.0882	-7.455	0.538333
C57	ChABC	After	CNTNAP2	P	After	3.89666	2.269224	1.72	0.0927	-0.67148	8.464806
C57	ChABC	After	CNTNAP2	ChABC	After	3.125	1.83765	1.7	0.0959	-0.57519	6.825194
CNTNAP2	ChABC	Before	CNTNAP2	P	Before	-3.33333	1.984889	-1.68	0.0999	-7.33	0.663333
CNTNAP2	P	After	CNTNAP2	P	Before	-4.23	2.49395	-1.7	0.101	-9.3393	0.879304
C57	ChABC	After	CNTNAP2	ChABC	Before	3	1.83765	1.63	0.1095	-0.70019	6.700194
C57	P	After	CNTNAP2	P	After	3.89666	2.390022	1.63	0.1099	-0.91474	8.708063
C57	P	After	CNTNAP2	ChABC	After	3.125	1.984889	1.57	0.1223	-0.87167	7.121666
C57	ChABC	Before	C57	P	Before	-3.04167	1.984889	-1.53	0.1324	-7.03833	0.954999
C57	P	After	CNTNAP2	ChABC	Before	3	1.984889	1.51	0.1376	-0.99667	6.996666
C57	ChABC	Before	CNTNAP2	P	Before	-2.70833	1.984889	-1.36	0.1791	-6.705	1.288333
C57	ChABC	After	C57	ChABC	Before	2.375	1.938465	1.23	0.233	-1.6372	6.387201
C57	ChABC	Before	C57	P	After	-2.375	1.984889	-1.2	0.2377	-6.37167	1.621666
C57	ChABC	Before	CNTNAP2	P	After	1.52166	2.269224	0.67	0.5059	-3.04648	6.089806
C57	ChABC	Before	CNTNAP2	ChABC	After	0.75	1.83765	0.41	0.6851	-2.95019	4.450194
CNTNAP2	ChABC	Before	CNTNAP2	P	After	0.89666	2.269224	0.4	0.6946	-3.67148	5.464806

C57	ChABC	Before	CNTNAP2	ChABC	Before	0.625	1.83765	0.34	0.7353	-3.07519	4.325194
CNTNAP2	ChABC	After	CNTNAP2	P	After	0.77166	2.269224	0.34	0.7354	-3.79648	5.339806
C57	ChABC	After	C57	P	Before	-0.66667	1.984889	-0.34	0.7385	-4.66333	3.329999
C57	P	After	C57	P	Before	-0.66667	2.238346	-0.3	0.7685	-5.29956	3.966224
C57	ChABC	After	CNTNAP2	P	Before	-0.33333	1.984889	-0.17	0.8674	-4.33	3.663333
C57	P	After	CNTNAP2	P	Before	-0.33333	2.121936	-0.16	0.8759	-4.60595	3.939282
C57	P	Before	CNTNAP2	P	Before	0.33333	2.121936	0.16	0.8759	-3.93928	4.605949
CNTNAP2	ChABC	After	CNTNAP2	ChABC	Before	-0.125	1.938465	-0.06	0.9491	-4.1372	3.887201
C57	ChABC	After	C57	P	After	0	1.984889	0	1	-3.99667	3.996666

Mouse	treatment	Time		Least Squares Mean
C57	P	Before	A	14.66667
CNTNAP2	P	Before	A	14.33333
C57	ChABC	After	A	14
C57	P	After	A	14
C57	ChABC	Before	A	11.625
CNTNAP2	ChABC	Before	A	11
CNTNAP2	ChABC	After	A	10.875
CNTNAP2	P	After	A	10.10334

S-FIG-18C

Source	Nparm	DFNum	DFDen	F Ratio	Prob > F
Mouse	1	1	23.1	4.552647	0.0437
treatment	1	1	23.1	0.874397	0.3594
Mouse*trea	1	1	23.1	0.014412	0.9055
Time	1	1	22	1.200119	0.2851
Mouse*Tim	1	1	22	1.881136	0.184
treatment*	1	1	22	0.807966	0.3785
Mouse*trea	1	1	22	3.642209	0.0695

Mouse	treatment	Time	-Mouse	-treatment	-Time	Difference	Std Error	t Ratio	Prob>|t|	Lower 95%	Upper 95%
C57	P	After	C57	P	Before	5.16667	1.960445	2.64	0.016	1.073	9.26037
C57	P	Before	CNTNAP2	P	Before	-5.91661	2.409	-2.46	0.0184	-10.7832	-1.05002
C57	ChABC	After	CNTNAP2	P	Before	-4.91661	2.409	-2.04	0.0478	-9.7832	-0.05002
C57	P	Before	CNTNAP2	ChABC	After	-4.5519	2.322952	-1.96	0.0571	-9.2472	0.14344
C57	P	Before	CNTNAP2	P	After	-4.65723	2.409	-1.93	0.0602	-9.5238	0.20936
C57	ChABC	Before	CNTNAP2	P	Before	-4.58328	2.409	-1.9	0.0642	-9.4499	0.28331
C57	ChABC	After	C57	P	After	-4.16667	2.413117	-1.73	0.0921	-9.0473	0.71401
C57	P	Before	CNTNAP2	ChABC	Before	-3.83333	2.257265	-1.7	0.0974	-8.3988	0.73212
C57	ChABC	Before	C57	P	After	-3.83333	2.413117	-1.59	0.1202	-8.714	1.04734
C57	ChABC	After	CNTNAP2	ChABC	After	-3.5519	2.322952	-1.53	0.1341	-8.2472	1.14344
C57	ChABC	After	CNTNAP2	P	After	-3.65723	2.409	-1.52	0.1367	-8.5238	1.20936
C57	ChABC	Before	CNTNAP2	ChABC	After	-3.21856	2.322952	-1.39	0.1736	-7.9139	1.47677
C57	ChABC	Before	CNTNAP2	P	After	-3.3239	2.409	-1.38	0.1752	-8.1905	1.54269
C57	ChABC	After	CNTNAP2	ChABC	Before	-2.83333	2.257265	-1.26	0.2169	-7.3988	1.73212
C57	ChABC	Before	CNTNAP2	ChABC	Before	-2.5	2.257265	-1.11	0.2748	-7.0655	2.06546
CNTNAP2	ChABC	Before	CNTNAP2	P	Before	-2.08328	2.252863	-0.92	0.3605	-6.6338	2.46722
CNTNAP2	P	After	CNTNAP2	P	Before	-1.25938	2.117347	-0.59	0.5568	-5.5981	3.07931
C57	P	After	CNTNAP2	ChABC	Before	1.33333	2.257265	0.59	0.5581	-3.2321	5.89879
CNTNAP2	ChABC	After	CNTNAP2	P	Before	-1.36471	2.318675	-0.59	0.5593	-6.0462	3.31679
C57	ChABC	Before	C57	P	Before	1.33333	2.413117	0.55	0.5837	-3.5473	6.21401
C57	ChABC	After	C57	P	Before	1	2.413117	0.41	0.6808	-3.8807	5.88068

CNTNAP2	ChABC	After	CNTNAP2	ChABC	Before	0.71856	1.784201	0.4	0.6912	-2.9929	4.43006
CNTNAP2	ChABC	Before	CNTNAP2	P	After	-0.8239	2.252863	-0.37	0.7165	-5.3744	3.7266
C57	P	After	CNTNAP2	P	Before	-0.74994	2.409	-0.31	0.7572	-5.6165	4.11664
C57	P	After	CNTNAP2	ChABC	After	0.61477	2.322952	0.26	0.7926	-4.0806	5.31011
C57	P	After	CNTNAP2	P	After	0.50944	2.409	0.21	0.8336	-4.3571	5.37602
C57	ChABC	After	C57	ChABC	Before	-0.33333	1.960445	-0.17	0.8667	-4.427	3.76037
CNTNAP2	ChABC	After	CNTNAP2	P	After	-0.10533	2.318675	-0.05	0.964	-4.7868	4.57617

Mouse	treatment	Time				Least Squares Mean
CNTNAP2	P	Before	A			16.58328
C57	P	After	A	B		15.83333
CNTNAP2	P	After	A	B	C	15.3239
CNTNAP2	ChABC	After	A	B	C	15.21856
CNTNAP2	ChABC	Before	A	B	C	14.5
C57	ChABC	Before	A	B	C	12
C57	ChABC	After		B	C	11.66667
C57	P	Before			C	10.66667

S-FIG-19A

Source	Nparm	DFNum	DFDen	F Ratio	Prob > F
Mouse	1	1	48.5	4.266477	0.0442
treatment	1	1	48.5	6.77E-05	0.9935
Mouse*trea	1	1	48.5	0.904333	0.3463
Time	1	1	48.2	2.250834	0.1401
Mouse*Tim	1	1	48.2	1.728684	0.1948
treatment*	1	1	48.2	0.005406	0.9417
Mouse*trea	1	1	48.2	1.443117	0.2355

Mouse	treatment	Time	-Mouse	-treatment	-Time	Difference	Std Error	t Ratio	Prob>|t|	Lower 95%	Upper 95%
C57	ChABC	After	CNTNAP2	ChABC	After	6.13137	2.08154	2.95	0.004	1.99954	10.26321
C57	ChABC	After	CNTNAP2	ChABC	Before	4.99348	2.048928	2.44	0.0166	0.92631	9.06066
C57	ChABC	After	CNTNAP2	P	Before	5.21119	2.202531	2.37	0.02	0.83911	9.58327
C57	ChABC	After	C57	ChABC	Before	4.11857	1.997094	2.06	0.0448	0.09993	8.13721
C57	ChABC	After	C57	P	Before	4.03536	2.202531	1.83	0.07	-0.33672	8.40744
C57	P	After	CNTNAP2	ChABC	After	3.85851	2.169326	1.78	0.0785	-0.44758	8.16461
C57	ChABC	After	CNTNAP2	P	After	3.6858	2.321629	1.59	0.1157	-0.92244	8.29404
C57	P	After	CNTNAP2	P	Before	2.93833	2.285675	1.29	0.2017	-1.59879	7.47546
C57	P	After	CNTNAP2	ChABC	Before	2.72062	2.138053	1.27	0.2063	-1.52347	6.96472
CNTNAP2	ChABC	After	CNTNAP2	P	After	-2.44557	2.290151	-1.07	0.2882	-6.99127	2.10013
C57	ChABC	After	C57	P	After	2.27286	2.202531	1.03	0.3047	-2.09922	6.64494
C57	ChABC	Before	CNTNAP2	ChABC	After	2.0128	2.08154	0.97	0.336	-2.11904	6.14464
C57	P	Before	CNTNAP2	ChABC	After	2.09601	2.169326	0.97	0.3364	-2.21008	6.40211
C57	ChABC	Before	C57	P	After	-1.84571	2.202531	-0.84	0.4041	-6.2178	2.52637
C57	P	After	C57	P	Before	1.7625	2.157108	0.82	0.4181	-2.57813	6.10313
CNTNAP2	P	After	CNTNAP2	P	Before	1.52539	2.278581	0.67	0.5062	-3.04893	6.09971
CNTNAP2	ChABC	After	CNTNAP2	ChABC	Before	-1.13789	1.903823	-0.6	0.5529	-4.96554	2.68976
C57	P	After	CNTNAP2	P	After	1.41294	2.400651	0.59	0.5575	-3.35217	6.17805
CNTNAP2	ChABC	Before	CNTNAP2	P	After	-1.30768	2.26055	-0.58	0.5643	-5.79468	3.17931
C57	P	Before	CNTNAP2	P	Before	1.17583	2.285675	0.51	0.6081	-3.36129	5.71296
C57	ChABC	Before	CNTNAP2	P	Before	1.09262	2.202531	0.5	0.621	-3.27946	5.4647
C57	P	Before	CNTNAP2	ChABC	Before	0.95813	2.138053	0.45	0.6551	-3.28597	5.20222
C57	ChABC	Before	CNTNAP2	ChABC	Before	0.87491	2.048928	0.43	0.6703	-3.19226	4.94209

CNTNAP2	ChABC	After	CNTNAP2	P	Before	-0.92018	2.169326	-0.42	0.6724	-5.22628	3.38591
C57	ChABC	Before	CNTNAP2	P	After	-0.43277	2.321629	-0.19	0.8525	-5.04101	4.17547
C57	P	Before	CNTNAP2	P	After	-0.34956	2.400651	-0.15	0.8845	-5.11467	4.41555
CNTNAP2	ChABC	Before	CNTNAP2	P	Before	0.21771	2.138053	0.1	0.9191	-4.02638	4.4618
C57	ChABC	Before	C57	P	Before	-0.08321	2.202531	-0.04	0.9699	-4.4553	4.28887

Mouse	treatment	Time			Least Squares Mean
C57	ChABC	After	A		23.03786
C57	P	After	A	B	20.765
CNTNAP2	P	After	A	B	19.35206
C57	P	Before	A	B	19.0025
C57	ChABC	Before		B	18.91929
CNTNAP2	ChABC	Before		B	18.04438
CNTNAP2	P	Before		B	17.82667
CNTNAP2	ChABC	After		B	16.90649

S-FIG-19B

Source	Nparm	DFNum	DFDen	F Ratio	Prob > F
Mouse	1	1	24.7	0.345689	0.5619
treatment	1	1	24.7	0.090365	0.7662
Mouse*trea	1	1	24.7	1.746318	0.1985
Time	1	1	24.5	0.737552	0.3988
Mouse*Tim	1	1	24.5	0.158404	0.6941
treatment*	1	1	24.5	2.568352	0.1218
Mouse*trea	1	1	24.5	8.655099	0.007

Mouse	treatment	Time	-Mouse	-treatment	-Time	Difference	Std Error	t Ratio	Prob>|t|	Lower 95%	Upper 95%
C57	ChABC	After	CNTNAP2	ChABC	After	7.03625	2.410793	2.92	0.0054	2.1831	11.8894
CNTNAP2	ChABC	After	CNTNAP2	P	After	-7.38297	2.979574	-2.48	0.017	-13.3807	-1.3852
C57	ChABC	After	CNTNAP2	P	Before	6.22292	2.603954	2.39	0.021	0.9809	11.4649
CNTNAP2	ChABC	After	CNTNAP2	ChABC	Before	-5.36	2.33602	-2.29	**0.0312**	-10.1931	-0.5269
CNTNAP2	P	After	CNTNAP2	P	Before	6.56964	3.06158	2.15	0.0407	0.2972	12.8421
CNTNAP2	ChABC	Before	CNTNAP2	P	Before	4.54667	2.603954	1.75	0.0875	-0.6953	9.7886
C57	ChABC	After	C57	P	After	4.01125	2.603954	1.54	0.1303	-1.2307	9.2532
C57	ChABC	Before	CNTNAP2	ChABC	After	3.62875	2.410793	1.51	0.1391	-1.2244	8.4819
C57	ChABC	After	C57	P	Before	3.90292	2.603954	1.5	0.1408	-1.3391	9.1449
C57	ChABC	After	C57	ChABC	Before	3.4075	2.33602	1.46	0.1582	-1.4256	8.2406
C57	P	After	CNTNAP2	P	After	-4.35797	3.137916	-1.39	0.1716	-10.6745	1.9585
C57	P	Before	CNTNAP2	P	After	-4.24964	3.137916	-1.35	0.1823	-10.5661	2.0669
C57	ChABC	Before	CNTNAP2	P	After	-3.75422	2.979574	-1.26	0.214	-9.752	2.2435
C57	P	Before	CNTNAP2	ChABC	After	3.13333	2.603954	1.2	0.235	-2.1086	8.3753
C57	P	After	CNTNAP2	ChABC	After	3.025	2.603954	1.16	0.2514	-2.217	8.267
C57	ChABC	Before	CNTNAP2	P	Before	2.81542	2.603954	1.08	0.2853	-2.4266	8.0574
C57	P	After	CNTNAP2	ChABC	Before	-2.335	2.603954	-0.9	0.3746	-7.577	2.907
C57	P	Before	CNTNAP2	ChABC	Before	-2.22667	2.603954	-0.86	0.3969	-7.4686	3.0153
C57	P	Before	CNTNAP2	P	Before	2.32	2.783744	0.83	0.4089	-3.2839	7.9239
C57	P	After	CNTNAP2	P	Before	2.21167	2.783744	0.79	0.431	-3.3922	7.8156
C57	ChABC	Before	CNTNAP2	ChABC	Before	-1.73125	2.410793	-0.72	0.4763	-6.5844	3.1219
C57	ChABC	After	CNTNAP2	ChABC	Before	1.67625	2.410793	0.7	0.4904	-3.1769	6.5294

CNTNAP2	ChABC	Before	CNTNAP2	P	After	-2.02297	2.979574	-0.68	0.5006	-8.0207	3.9748
CNTNAP2	ChABC	After	CNTNAP2	P	Before	-0.81333	2.603954	-0.31	0.7562	-6.0553	4.4286
C57	ChABC	Before	C57	P	After	0.60375	2.603954	0.23	0.8177	-4.6382	5.8457
C57	ChABC	Before	C57	P	Before	0.49542	2.603954	0.19	0.8499	-4.7466	5.7374
C57	ChABC	After	CNTNAP2	P	After	-0.34672	2.979574	-0.12	0.9079	-6.3445	5.651
C57	P	After	C57	P	Before	-0.10833	2.697404	-0.04	0.9683	-5.6892	5.4725

Mouse	treatment	Time				Least Squares Mean
CNTNAP2	P	After	A			22.61297
C57	ChABC	After	A			22.26625
CNTNAP2	ChABC	Before	A	B		20.59
C57	ChABC	Before	A	B	C	18.85875
C57	P	Before	A	B	C	18.36333
C57	P	After	A	B	C	18.255
CNTNAP2	P	Before		B	C	16.04333
CNTNAP2	ChABC	After			C	15.23

S-FIG-19C

Source	Nparm	DFNum	DFDen	F Ratio	Prob > F
Mouse	1	1	22.5	3.87413	0.0615
treatment	1	1	22.5	0.077477	0.7833
Mouse*trea	1	1	22.5	0.100706	0.7539
Time	1	1	21.6	2.864024	0.105
Mouse*Tim	1	1	21.6	1.456419	0.2406
treatment*	1	1	21.6	1.184087	0.2885
Mouse*trea	1	1	21.6	0.372732	0.5479

Mouse	treatment	Time	-Mouse	-treatment	-Time	Difference	Std Error	t Ratio	Prob>|t|	Lower 95%	Upper 95%
C57	ChABC	After	CNTNAP2	ChABC	Before	8.56792	3.306615	2.59	0.0133	1.8863	15.24954
C57	P	After	CNTNAP2	ChABC	Before	7.77625	3.306615	2.35	0.0237	1.0946	14.45787
C57	ChABC	After	CNTNAP2	P	After	6.69373	3.539362	1.89	0.0656	-0.4523	13.83974
C57	P	After	CNTNAP2	P	After	5.90206	3.539362	1.67	0.103	-1.2439	13.04808
C57	ChABC	After	C57	ChABC	Before	5.06667	3.014964	1.68	0.109	-1.2387	11.37202
C57	ChABC	After	CNTNAP2	ChABC	After	5.29068	3.408157	1.55	0.1283	-1.593	12.17439
C57	ChABC	After	CNTNAP2	P	Before	4.87348	3.539362	1.38	0.1759	-2.2725	12.01949
C57	P	After	CNTNAP2	ChABC	After	4.49901	3.408157	1.32	0.1942	-2.3847	11.38273
C57	P	Before	CNTNAP2	ChABC	Before	4.14292	3.306615	1.25	0.2175	-2.5387	10.82454
C57	ChABC	After	C57	P	Before	4.425	3.53492	1.25	0.2179	-2.718	11.56795
C57	ChABC	Before	C57	P	After	-4.275	3.53492	-1.21	0.2336	-11.418	2.86795
C57	P	After	C57	P	Before	3.63333	3.014964	1.21	0.2428	-2.672	9.93869
CNTNAP2	ChABC	After	CNTNAP2	ChABC	Before	3.27724	2.738492	1.2	0.2451	-2.4258	8.98032
C57	P	After	CNTNAP2	P	Before	4.08181	3.539362	1.15	0.2554	-3.0642	11.22782
CNTNAP2	ChABC	Before	CNTNAP2	P	Before	-3.69444	3.311363	-1.12	0.271	-10.3794	2.99056
C57	ChABC	Before	CNTNAP2	ChABC	Before	3.50125	3.306615	1.06	0.296	-3.1804	10.18287
C57	P	Before	CNTNAP2	P	After	2.26873	3.539362	0.64	0.5251	-4.8773	9.41474
CNTNAP2	ChABC	Before	CNTNAP2	P	After	-1.87419	3.311363	-0.57	0.5745	-8.5592	4.81082
CNTNAP2	P	After	CNTNAP2	P	Before	-1.82026	3.215805	-0.57	0.5759	-8.4115	4.77095
C57	ChABC	Before	CNTNAP2	P	After	1.62706	3.539362	0.46	0.6481	-5.5189	8.77308
CNTNAP2	ChABC	After	CNTNAP2	P	After	1.40305	3.412764	0.41	0.6831	-5.4847	8.2908

C57	P	Before	CNTNAP2	ChABC	After	0.86568	3.408157	0.25	0.8008	-6.018	7.74939
C57	ChABC	After	C57	P	After	0.79167	3.53492	0.22	0.8239	-6.3513	7.93462
C57	ChABC	Before	C57	P	Before	-0.64167	3.53492	-0.18	0.8569	-7.7846	6.50129
C57	P	Before	CNTNAP2	P	Before	0.44848	3.539362	0.13	0.8998	-6.6975	7.59449
CNTNAP2	ChABC	After	CNTNAP2	P	Before	-0.4172	3.412764	-0.12	0.9033	-7.3049	6.47054
C57	ChABC	Before	CNTNAP2	ChABC	After	0.22401	3.408157	0.07	0.9479	-6.6597	7.10773
C57	ChABC	Before	CNTNAP2	P	Before	-0.19319	3.539362	-0.05	0.9567	-7.3392	6.95282

Mouse	treatment	Time			Least Squares Mean
C57	ChABC	After	A		24.06667
C57	P	After	A		23.275
C57	P	Before	A	B	19.64167
CNTNAP2	P	Before	A	B	19.19319
C57	ChABC	Before	A	B	19
CNTNAP2	ChABC	After	A	B	18.77599
CNTNAP2	P	After	A	B	17.37294
CNTNAP2	ChABC	Before		B	15.49875

S-FIG-20A

Source	Nparm	DFNum	DFDen	F Ratio	Prob > F
Mouse	1	1	48.7	0.632762	0.4302
treatment	1	1	48.7	0.820793	0.3694
Mouse*trea	1	1	48.7	0.164344	0.687
Time	1	1	48.8	0.291036	0.592
Mouse*Tim	1	1	48.8	0.406971	0.5265
treatment*	1	1	48.8	0.294362	0.5899
Mouse*trea	1	1	48.8	4.745747	0.0342

Mouse	treatment	Time	-Mouse	-treatment	-Time	Difference	Std Error	t Ratio	Prob>|t|	Lower 95%	Upper 95%
C57	ChABC	After	CNTNAP2	ChABC	After	5.16975	3.018136	1.71	0.0899	-0.8204	11.15994
C57	P	Before	CNTNAP2	ChABC	After	5.34856	3.145376	1.7	0.0923	-0.8942	11.59129
CNTNAP2	ChABC	After	CNTNAP2	P	After	-5.19101	3.322475	-1.56	0.1215	-11.7852	1.40319
CNTNAP2	ChABC	After	CNTNAP2	ChABC	Before	-4.55565	2.945131	-1.55	0.1284	-10.4749	1.3636
C57	ChABC	Before	C57	P	Before	-4.72452	3.192994	-1.48	0.1422	-11.0618	1.61271
C57	P	After	CNTNAP2	ChABC	After	4.60773	3.145376	1.46	0.1462	-1.635	10.85045
C57	ChABC	After	C57	ChABC	Before	4.54571	3.096069	1.47	0.1487	-1.6822	10.77366
C57	ChABC	Before	CNTNAP2	P	After	-4.56698	3.36759	-1.36	0.1782	-11.2507	2.11678
C57	P	Before	CNTNAP2	P	Before	4.395	3.313527	1.33	0.1878	-2.1815	10.97146
C57	ChABC	Before	CNTNAP2	ChABC	Before	-3.93161	2.970317	-1.32	0.1887	-9.8269	1.96367
C57	ChABC	After	CNTNAP2	P	Before	4.21619	3.192994	1.32	0.1898	-2.121	10.55343
C57	ChABC	Before	C57	P	After	-3.98369	3.192994	-1.25	0.2152	-10.3209	2.35355
CNTNAP2	P	After	CNTNAP2	P	Before	4.23745	3.511224	1.21	0.233	-2.809	11.28395
CNTNAP2	ChABC	Before	CNTNAP2	P	Before	3.60208	3.099521	1.16	0.248	-2.5496	9.7538
C57	P	After	CNTNAP2	P	Before	3.65417	3.313527	1.1	0.2728	-2.9223	10.23063
CNTNAP2	ChABC	After	CNTNAP2	P	Before	-0.95356	3.145376	-0.3	0.7624	-7.1963	5.28916
C57	P	Before	CNTNAP2	ChABC	Before	0.79292	3.099521	0.26	0.7986	-5.3588	6.94463
C57	P	After	C57	P	Before	-0.74083	3.344136	-0.22	0.8256	-7.4678	5.98612
C57	ChABC	Before	CNTNAP2	ChABC	After	0.62404	3.018136	0.21	0.8366	-5.3661	6.61422
C57	ChABC	After	CNTNAP2	ChABC	Before	0.61411	2.970317	0.21	0.8366	-5.2812	6.50939
CNTNAP2	ChABC	Before	CNTNAP2	P	After	-0.63537	3.279097	-0.19	0.8468	-7.1435	5.87275

C57	ChABC	After	C57	P	After	0.56202	3.192994	0.18	0.8606	-5.7752	6.89926
C57	P	After	CNTNAP2	P	After	-0.58329	3.482084	-0.17	0.8673	-7.4943	6.32771
C57	ChABC	Before	CNTNAP2	P	Before	-0.32952	3.192994	-0.1	0.918	-6.6668	6.00771
C57	ChABC	After	C57	P	Before	-0.17881	3.192994	-0.06	0.9555	-6.516	6.15843
C57	P	Before	CNTNAP2	P	After	0.15755	3.482084	0.05	0.964	-6.7534	7.06854
C57	P	After	CNTNAP2	ChABC	Before	0.05208	3.099521	0.02	0.9866	-6.0996	6.2038
C57	ChABC	After	CNTNAP2	P	After	-0.02126	3.36759	-0.01	0.995	-6.705	6.66249

Mouse	treatment	Time		Least Squares Mean
C57	P	Before	A	36.30667
CNTNAP2	P	After	A	36.14912
C57	ChABC	After	A	36.12786
C57	P	After	A	35.56583
CNTNAP2	ChABC	Before	A	35.51375
CNTNAP2	P	Before	A	31.91167
C57	ChABC	Before	A	31.58214
CNTNAP2	ChABC	After	A	30.95811

S-FIG-20B

Source	Nparm	DFNum	DFDen	F Ratio	Prob > F
Mouse	1	1	24.3	0.043335	0.8368
treatment	1	1	24.3	2.087012	0.1613
Mouse*trea	1	1	24.3	0.022944	0.8809
Time	1	1	24.5	0.001729	0.9672
Mouse*Tim	1	1	24.5	0.485792	0.4924
treatment*	1	1	24.5	5.471312	0.0278
Mouse*trea	1	1	24.5	6.96547	0.0142

Mouse	treatment	Time	-Mouse	-treatment	-Time	Difference	Std Error	t Ratio	Prob>|t|	Lower 95%	Upper 95%
CNTNAP2	ChABC	After	CNTNAP2	P	After	-15.1826	5.010028	-3.03	0.004	-25.2676	-5.0976
CNTNAP2	ChABC	After	CNTNAP2	ChABC	Before	-13.2138	4.194137	-3.15	0.0045	-21.8928	-4.5347
C57	P	After	CNTNAP2	ChABC	After	10.7517	4.378398	2.46	0.0179	1.9373	19.566
C57	P	Before	CNTNAP2	ChABC	After	9.7233	4.378398	2.22	0.0314	0.909	18.5377
C57	ChABC	After	CNTNAP2	ChABC	After	8.615	4.053609	2.13	**0.039**	0.4545	16.7755
CNTNAP2	ChABC	Before	CNTNAP2	P	Before	8.1704	4.378398	1.87	0.0684	-0.6439	16.9848
CNTNAP2	P	After	CNTNAP2	P	Before	10.1393	5.420737	1.87	**0.0719**	-0.9648	21.2433
C57	ChABC	Before	CNTNAP2	P	After	-9.0038	5.010028	-1.8	0.0789	-19.0889	1.0812
C57	ChABC	Before	CNTNAP2	ChABC	Before	-7.035	4.053609	-1.74	0.0894	-15.1955	1.1255
C57	ChABC	Before	CNTNAP2	ChABC	After	6.1788	4.053609	1.52	0.1343	-1.9818	14.3393
C57	ChABC	After	CNTNAP2	P	After	-6.5676	5.010028	-1.31	0.1964	-16.6526	3.5174
C57	P	After	CNTNAP2	P	Before	5.7083	4.680705	1.22	0.2289	-3.7146	15.1313
CNTNAP2	ChABC	After	CNTNAP2	P	Before	-5.0433	4.378398	-1.15	0.2554	-13.8577	3.771
C57	ChABC	After	CNTNAP2	ChABC	Before	-4.5987	4.053609	-1.13	0.2625	-12.7593	3.5618
C57	ChABC	Before	C57	P	After	-4.5729	4.378398	-1.04	0.3018	-13.3873	4.2414
C57	P	Before	CNTNAP2	P	After	-5.4593	5.276269	-1.03	0.3062	-16.0803	5.1618
C57	P	Before	CNTNAP2	P	Before	4.68	4.680705	1	0.3226	-4.7429	14.1029
C57	P	After	CNTNAP2	P	After	-4.4309	5.276269	-0.84	0.4054	-15.0519	6.1901
C57	ChABC	After	CNTNAP2	P	Before	3.5717	4.378398	0.82	0.4189	-5.2427	12.386
C57	ChABC	Before	C57	P	Before	-3.5446	4.378398	-0.81	0.4224	-12.3589	5.2698
C57	P	Before	CNTNAP2	ChABC	Before	-3.4904	4.378398	-0.8	0.4295	-12.3048	5.3239
C57	ChABC	After	C57	ChABC	Before	2.4362	4.194137	0.58	0.567	-6.2428	11.1153

C57	P	After	CNTNAP2	ChABC	Before	-2.4621	4.378398	-0.56	0.5766	-11.2764	6.3523
C57	ChABC	After	C57	P	After	-2.1367	4.378398	-0.49	0.6279	-10.951	6.6777
CNTNAP2	ChABC	Before	CNTNAP2	P	After	-1.9688	5.010028	-0.39	0.6962	-12.0539	8.1162
C57	ChABC	Before	CNTNAP2	P	Before	1.1354	4.378398	0.26	0.7965	-7.6789	9.9498
C57	ChABC	After	C57	P	Before	-1.1083	4.378398	-0.25	0.8013	-9.9227	7.706
C57	P	After	C57	P	Before	1.0283	4.842972	0.21	0.8337	-8.9934	11.05

Mouse	treatment	Time			Least Squares Mean
CNTNAP2	P	After	A		40.51259
CNTNAP2	ChABC	Before	A		38.54375
C57	P	After	A		36.08167
C57	P	Before	A		35.05333
C57	ChABC	After	A		33.945
C57	ChABC	Before	A	B	31.50875
CNTNAP2	P	Before	A	B	30.37333
CNTNAP2	ChABC	After		B	25.33

S-FIG-20C

Source	Nparm	DFNum	DFDen	F Ratio	Prob > F
Mouse	1	1	22.8	0.470153	0.4998
treatment	1	1	22.8	0.002399	0.9614
Mouse*trea	1	1	22.8	0.198329	0.6603
Time	1	1	21.8	2.099407	0.1616
Mouse*Tim	1	1	21.8	0.00874	0.9264
treatment*	1	1	21.8	3.592237	0.0714
Mouse*trea	1	1	21.8	0.745744	0.3972

Mouse	treatment	Time	-Mouse	-treatment	-Time	Difference	Std Error	t Ratio	Prob>|t|	Lower 95%	Upper 95%
C57	ChABC	After	C57	ChABC	Before	7.35833	3.584978	2.05	0.0538	-0.1326	14.84926
C57	ChABC	After	C57	P	After	3.98833	4.326285	0.92	0.3622	-4.7585	12.73521
C57	ChABC	After	C57	P	Before	1.47833	4.326285	0.34	0.7344	-7.2685	10.22521
C57	ChABC	After	CNTNAP2	ChABC	After	1.95076	4.167359	0.47	0.6422	-6.4701	10.3716
C57	ChABC	After	CNTNAP2	ChABC	Before	6.55458	4.046869	1.62	0.1133	-1.6274	14.73654
C57	ChABC	After	CNTNAP2	P	After	4.9756	4.324299	1.15	0.2566	-3.7582	13.70935
C57	ChABC	After	CNTNAP2	P	Before	5.8889	4.324299	1.36	0.1807	-2.8449	14.62265
C57	ChABC	Before	C57	P	After	-3.37	4.326285	-0.78	0.4406	-12.1169	5.37688
C57	ChABC	Before	C57	P	Before	-5.88	4.326285	-1.36	0.1818	-14.6269	2.86688
C57	ChABC	Before	CNTNAP2	ChABC	After	-5.40757	4.167359	-1.3	0.2018	-13.8284	3.01326
C57	ChABC	Before	CNTNAP2	ChABC	Before	-0.80375	4.046869	-0.2	0.8436	-8.9857	7.3782
C57	ChABC	Before	CNTNAP2	P	After	-2.38274	4.324299	-0.55	0.5846	-11.1165	6.35102
C57	ChABC	Before	CNTNAP2	P	Before	-1.46943	4.324299	-0.34	0.7357	-10.2032	7.26432
C57	P	After	C57	P	Before	-2.51	3.584978	-0.7	0.4921	-10.0009	4.98093
C57	P	After	CNTNAP2	ChABC	After	-2.03757	4.167359	-0.49	0.6275	-10.4584	6.38326
C57	P	After	CNTNAP2	ChABC	Before	2.56625	4.046869	0.63	0.5296	-5.6157	10.7482
C57	P	After	CNTNAP2	P	After	0.98726	4.324299	0.23	0.8205	-7.7465	9.72102
C57	P	After	CNTNAP2	P	Before	1.90057	4.324299	0.44	0.6626	-6.8332	10.63432
C57	P	Before	CNTNAP2	ChABC	After	0.47243	4.167359	0.11	0.9103	-7.9484	8.89326
C57	P	Before	CNTNAP2	ChABC	Before	5.07625	4.046869	1.25	0.2171	-3.1057	13.2582
C57	P	Before	CNTNAP2	P	After	3.49726	4.324299	0.81	0.4233	-5.2365	12.23102

C57	P	Before	CNTNAP2	P	Before	4.41057	4.324299	1.02	0.3137	-4.3232	13.14432
CNTNAP2	ChABC	After	CNTNAP2	ChABC	Before	4.60382	3.260182	1.41	0.1727	-2.1814	11.38902
CNTNAP2	ChABC	After	CNTNAP2	P	After	3.02483	4.165298	0.73	0.4718	-5.3837	11.43333
CNTNAP2	ChABC	After	CNTNAP2	P	Before	3.93814	4.165298	0.95	0.3499	-4.4704	12.34664
CNTNAP2	ChABC	Before	CNTNAP2	P	After	-1.57899	4.044746	-0.39	0.6983	-9.7471	6.58911
CNTNAP2	ChABC	Before	CNTNAP2	P	Before	-0.66568	4.044746	-0.16	0.8701	-8.8338	7.50242
CNTNAP2	P	After	CNTNAP2	P	Before	0.91331	3.852889	0.24	0.8144	-6.9826	8.80917

Mouse	treatment	Time		Least Squares Mean
C57	ChABC	After	A	39.03833
C57	P	Before	A	37.56
CNTNAP2	ChABC	After	A	37.08757
C57	P	After	A	35.05
CNTNAP2	P	After	A	34.06274
CNTNAP2	P	Before	A	33.14943
CNTNAP2	ChABC	Before	A	32.48375
C57	ChABC	Before	A	31.68

S-FIG-21A

Source	Nparm	DFNum	DFDen	F Ratio	Prob > F
Mouse	1	1	50.5	0.075547	0.7845
treatment	1	1	50.5	1.333364	0.2536
Mouse*trea	1	1	50.5	0.99445	0.3234
Time	1	1	50.5	1.131086	0.2926
Mouse*Tim	1	1	50.5	2.641217	0.1104
treatment*	1	1	50.5	0.399521	0.5302
Mouse*trea	1	1	50.5	0.07222	0.7892

Mouse	treatment	Time	-Mouse	-treatment	-Time	Difference	Std Error	t Ratio	Prob>|t|	Lower 95%	Upper 95%
C57	P	After	CNTNAP2	ChABC	After	3.08336	1.930271	1.6	0.1134	-0.74769	6.914418
CNTNAP2	ChABC	After	CNTNAP2	P	After	-3.00007	2.038702	-1.47	0.1444	-7.04633	1.046188
C57	P	After	C57	P	Before	3	2.033843	1.48	0.1466	-1.08756	7.087559
C57	ChABC	Before	C57	P	After	-2.79762	1.959563	-1.43	0.1566	-6.68681	1.091571
C57	ChABC	After	CNTNAP2	ChABC	After	2.57146	1.85218	1.39	0.1682	-1.1046	6.247524
CNTNAP2	ChABC	After	CNTNAP2	P	Before	-2.6667	1.930271	-1.38	0.1703	-6.49775	1.164355
C57	P	Before	CNTNAP2	P	After	-2.91671	2.136732	-1.37	0.1754	-7.15753	1.324115
C57	ChABC	Before	CNTNAP2	P	After	-2.71433	2.066458	-1.31	0.1921	-6.81567	1.387021
C57	P	Before	CNTNAP2	P	Before	-2.58333	2.033535	-1.27	0.207	-6.61934	1.45267
C57	ChABC	After	C57	P	Before	2.4881	1.959563	1.27	0.2072	-1.40109	6.377285
C57	ChABC	Before	CNTNAP2	P	Before	-2.38095	1.959563	-1.22	0.2273	-6.27014	1.508238
C57	ChABC	After	C57	ChABC	Before	2.28571	1.882973	1.21	0.2306	-1.49863	6.070058
C57	P	After	CNTNAP2	ChABC	Before	1.64583	1.902198	0.87	0.389	-2.1295	5.421169
CNTNAP2	ChABC	After	CNTNAP2	ChABC	Before	-1.43753	1.791641	-0.8	0.4261	-5.03544	2.160376
CNTNAP2	ChABC	Before	CNTNAP2	P	After	-1.56254	2.012142	-0.78	0.4393	-5.55609	2.431005
C57	P	Before	CNTNAP2	ChABC	Before	-1.35417	1.902198	-0.71	0.4782	-5.1295	2.421169
CNTNAP2	ChABC	Before	CNTNAP2	P	Before	-1.22917	1.902198	-0.65	0.5197	-5.0045	2.546169
C57	ChABC	Before	CNTNAP2	ChABC	Before	-1.15179	1.822904	-0.63	0.529	-4.76975	2.466174
C57	ChABC	After	CNTNAP2	ChABC	Before	1.13393	1.822904	0.62	0.5354	-2.48403	4.751889
C57	ChABC	After	C57	P	After	-0.5119	1.959563	-0.26	0.7945	-4.40109	3.377285
C57	ChABC	After	CNTNAP2	P	After	-0.42861	2.066458	-0.21	0.8361	-4.52996	3.672735
C57	P	After	CNTNAP2	P	Before	0.41667	2.033535	0.2	0.8381	-3.61934	4.45267
CNTNAP2	P	After	CNTNAP2	P	Before	0.33337	2.137025	0.16	0.8766	-3.95217	4.618922

C57	ChABC	Before	CNTNAP2	ChABC	After	0.28575	1.85218	0.15	0.8777	-3.39032	3.96181
C57	ChABC	Before	C57	P	Before	0.20238	1.959563	0.1	0.918	-3.68681	4.091571
C57	ChABC	After	CNTNAP2	P	Before	-0.09524	1.959563	-0.05	0.9613	-3.98443	3.793952
C57	P	Before	CNTNAP2	ChABC	After	0.08336	1.930271	0.04	0.9656	-3.74769	3.914418
C57	P	After	CNTNAP2	P	After	0.08329	2.136732	0.04	0.969	-4.15753	4.324115

Mouse	treatment	Time		Least Squares Mean
C57	P	After	A	14.08333
CNTNAP2	P	After	A	14.00004
CNTNAP2	P	Before	A	13.66667
C57	ChABC	After	A	13.57143
CNTNAP2	ChABC	Before	A	12.4375
C57	ChABC	Before	A	11.28571
C57	P	Before	A	11.08333
CNTNAP2	ChABC	After	A	10.99997

S-FIG-21B

Source	Nparm	DFNum	DFDen	F Ratio	Prob > F
Mouse	1	1	25	0.677421	0.4183
treatment	1	1	25	1.370581	0.2528
Mouse*trea	1	1	25	1.064308	0.3121
Time	1	1	24.2	0.025191	0.8752
Mouse*Tim	1	1	24.2	0.001239	0.9722
treatment*	1	1	24.2	1.063632	0.3126
Mouse*trea	1	1	24.2	2.357758	0.1376

Mouse	treatment	Time	-Mouse	-treatment	-Time	Difference	Std Error	t Ratio	Prob>|t|	Lower 95%	Upper 95%
C57	ChABC	After	C57	ChABC	Before	0.75	1.894059	0.4	0.6958	-3.1697	4.6697
C57	ChABC	After	C57	P	After	0.33333	2.522986	0.13	0.8955	-4.7597	5.42636
C57	ChABC	After	C57	P	Before	-3.55E-15	2.522986	0	1	-5.093	5.09303
C57	ChABC	After	CNTNAP2	ChABC	After	2	2.335831	0.86	0.3968	-2.7152	6.71523
C57	ChABC	After	CNTNAP2	ChABC	Before	-0.625	2.335831	-0.27	0.7904	-5.3402	4.09023
C57	ChABC	After	CNTNAP2	P	After	-4.05708	2.844672	-1.43	0.1608	-9.7886	1.67442
C57	ChABC	After	CNTNAP2	P	Before	-1.16667	2.522986	-0.46	0.6462	-6.2597	3.92636
C57	ChABC	Before	C57	P	After	-0.41667	2.522986	-0.17	0.8696	-5.5097	4.67636
C57	ChABC	Before	C57	P	Before	-0.75	2.522986	-0.3	0.7677	-5.843	4.34303
C57	ChABC	Before	CNTNAP2	ChABC	After	1.25	2.335831	0.54	0.5954	-3.4652	5.96523
C57	ChABC	Before	CNTNAP2	ChABC	Before	-1.375	2.335831	-0.59	0.5593	-6.0902	3.34023
C57	ChABC	Before	CNTNAP2	P	After	-4.80708	2.844672	-1.69	0.0981	-10.5386	0.92442
C57	ChABC	Before	CNTNAP2	P	Before	-1.91667	2.522986	-0.76	0.4517	-7.0097	3.17636
C57	P	After	C57	P	Before	-0.33333	2.18707	-0.15	0.8802	-4.8594	4.19274
C57	P	After	CNTNAP2	ChABC	After	1.66667	2.522986	0.66	0.5125	-3.4264	6.75969
C57	P	After	CNTNAP2	ChABC	Before	-0.95833	2.522986	-0.38	0.706	-6.0514	4.13469
C57	P	After	CNTNAP2	P	After	-4.39041	3.000252	-1.46	0.1504	-10.4363	1.65545
C57	P	After	CNTNAP2	P	Before	-1.5	2.697185	-0.56	0.5811	-6.9447	3.94468
C57	P	Before	CNTNAP2	ChABC	After	2	2.522986	0.79	0.4324	-3.093	7.09303
C57	P	Before	CNTNAP2	ChABC	Before	-0.625	2.522986	-0.25	0.8056	-5.718	4.46803
C57	P	Before	CNTNAP2	P	After	-4.05708	3.000252	-1.35	0.1832	-10.1029	1.98878
C57	P	Before	CNTNAP2	P	Before	-1.16667	2.697185	-0.43	0.6676	-6.6113	4.27801

CNTNAP2	ChABC	After	CNTNAP2	ChABC	Before	-2.625	1.894059	-1.39	0.1792	-6.5447	1.2947
CNTNAP2	ChABC	After	CNTNAP2	P	After	-6.05708	2.844672	-2.13	0.0388	-11.7886	-0.32558
CNTNAP2	ChABC	After	CNTNAP2	P	Before	-3.16667	2.522986	-1.26	0.2164	-8.2597	1.92636
CNTNAP2	ChABC	Before	CNTNAP2	P	After	-3.43208	2.844672	-1.21	0.234	-9.1636	2.29942
CNTNAP2	ChABC	Before	CNTNAP2	P	Before	-0.54167	2.522986	-0.21	0.8311	-5.6347	4.55136
CNTNAP2	P	After	CNTNAP2	P	Before	2.89041	2.551466	1.13	0.2673	-2.3458	8.12662

Mouse	treatment	Time			Least Squares Mean
CNTNAP2	P	After	A		16.05708
CNTNAP2	P	Before	A	B	13.16667
CNTNAP2	ChABC	Before	A	B	12.625
C57	P	Before	A	B	12
C57	ChABC	After	A	B	12
C57	P	After	A	B	11.66667
C57	ChABC	Before	A	B	11.25
CNTNAP2	ChABC	After		B	10

S-FIG-21C

Source	Nparm	DFNum	DFDen	F Ratio	Prob > F
Mouse	1	1	23.3	0.153786	0.6985
treatment	1	1	23.3	0.222042	0.6419
Mouse*trea	1	1	23.3	0.353799	0.5577
Time	1	1	24	1.807626	0.1914
Mouse*Tim	1	1	24	3.617324	0.0692
treatment*	1	1	24	0.005605	0.9409
Mouse*trea	1	1	24	0.284976	0.5984

Mouse	treatment	Time	-Mouse	-treatment	-Time	Difference	Std Error	t Ratio	Prob>|t|	Lower 95%	Upper 95%
C57	P	After	C57	P	Before	6.33333	3.397911	1.86	0.0757	-0.7134	13.38006
C57	ChABC	After	C57	P	Before	5.5	3.078082	1.79	0.0813	-0.7143	11.71434
C57	ChABC	Before	C57	P	After	-5.16667	3.078082	-1.68	0.1008	-11.381	1.04767
C57	P	After	CNTNAP2	ChABC	After	4.41179	2.969256	1.49	0.1448	-1.5813	10.4049
C57	P	After	CNTNAP2	ChABC	Before	4.25	2.879282	1.48	0.1475	-1.563	10.06298
C57	P	Before	CNTNAP2	P	Before	-4.27484	3.084781	-1.39	0.1731	-10.4998	1.95012
C57	ChABC	After	C57	ChABC	Before	4.33333	3.397911	1.28	0.2155	-2.7134	11.38006
C57	P	After	CNTNAP2	P	After	3.72829	3.084781	1.21	0.2336	-2.4967	9.95326
C57	ChABC	After	CNTNAP2	ChABC	After	3.57846	2.969256	1.21	0.2349	-2.4146	9.57157
C57	ChABC	After	CNTNAP2	ChABC	Before	3.41667	2.879282	1.19	0.2421	-2.3963	9.22965
C57	ChABC	Before	CNTNAP2	P	Before	-3.10817	3.084781	-1.01	0.3194	-9.3331	3.11679
C57	ChABC	After	CNTNAP2	P	After	2.89496	3.084781	0.94	0.3534	-3.33	9.11992
C57	P	Before	CNTNAP2	P	After	-2.60504	3.084781	-0.84	0.4032	-8.83	3.61992
CNTNAP2	ChABC	After	CNTNAP2	P	Before	-2.3533	2.976199	-0.79	0.4335	-8.3579	3.65125
CNTNAP2	ChABC	Before	CNTNAP2	P	Before	-2.19151	2.886442	-0.76	0.4519	-8.0159	3.63289
C57	P	Before	CNTNAP2	ChABC	Before	-2.08333	2.879282	-0.72	0.4734	-7.8963	3.72965
C57	P	After	CNTNAP2	P	Before	2.05849	3.084781	0.67	0.5082	-4.1665	8.28346
C57	P	Before	CNTNAP2	ChABC	After	-1.92154	2.969256	-0.65	0.5211	-7.9146	4.07157
CNTNAP2	P	After	CNTNAP2	P	Before	-1.6698	3.302382	-0.51	0.6169	-8.4201	5.08046
C57	ChABC	Before	CNTNAP2	P	After	-1.43837	3.084781	-0.47	0.6434	-7.6633	4.78659
C57	ChABC	After	CNTNAP2	P	Before	1.22516	3.084781	0.4	0.6933	-4.9998	7.45012
C57	ChABC	Before	C57	P	Before	1.16667	3.078082	0.38	0.7066	-5.0477	7.38101
C57	ChABC	Before	CNTNAP2	ChABC	Before	-0.91667	2.879282	-0.32	0.7518	-6.7297	4.89632

C57	ChABC	After	C57	P	After	-0.83333	3.078082	-0.27	0.7879	-7.0477	5.38101
C57	ChABC	Before	CNTNAP2	ChABC	After	-0.75487	2.969256	-0.25	0.8006	-6.748	5.23823
CNTNAP2	ChABC	After	CNTNAP2	P	After	-0.6835	2.976199	-0.23	0.8195	-6.6881	5.32105
CNTNAP2	ChABC	Before	CNTNAP2	P	After	-0.52171	2.886442	-0.18	0.8574	-6.3461	5.30269
CNTNAP2	ChABC	After	CNTNAP2	ChABC	Before	-0.16179	3.03077	-0.05	0.9579	-6.4245	6.1009

Mouse	treatment	Time		Least Squares Mean
C57	P	After	A	16.5
C57	ChABC	After	A	15.66667
CNTNAP2	P	Before	A	14.44151
CNTNAP2	P	After	A	12.77171
CNTNAP2	ChABC	Before	A	12.25
CNTNAP2	ChABC	After	A	12.08821
C57	ChABC	Before	A	11.33333
C57	P	Before	A	10.16667

S-FIG-22A

Source	Nparm	DFNum	DFDen	F Ratio	Prob > F
Mouse	1	1	48.1	3.434793	0.07
treatment	1	1	48.1	1.861067	0.1789
Mouse*trea	1	1	48.1	0.001846	0.9659
Time	1	1	47.5	5.123033	0.0282
Mouse*Tim	1	1	47.5	6.669346	0.0129
treatment*	1	1	47.5	0.011312	0.9157
Mouse*trea	1	1	47.5	0.635884	0.4292

Mouse	treatment	Time	-Mouse	-treatment	-Time	Difference	Std Error	t Ratio	Prob>|t|	Lower 95%	Upper 95%
C57	ChABC	After	CNTNAP2	P	Before	-6.95226	2.212656	-3.14	0.0022	-11.3456	-2.55889
CNTNAP2	ChABC	After	CNTNAP2	P	Before	-6.47213	2.178649	-2.97	0.0038	-10.7978	-2.14648
C57	ChABC	Before	CNTNAP2	P	Before	-6.36298	2.212656	-2.88	0.005	-10.7564	-1.9696
CNTNAP2	P	After	CNTNAP2	P	Before	-5.58056	2.203107	-2.53	0.0145	-10.005	-1.15611
C57	P	Before	CNTNAP2	P	Before	-5.5825	2.296182	-2.43	0.0169	-10.1417	-1.02328
C57	ChABC	After	CNTNAP2	ChABC	Before	-4.66768	2.058347	-2.27	0.0256	-8.7547	-0.5807
CNTNAP2	ChABC	After	CNTNAP2	ChABC	Before	-4.18755	1.836853	-2.28	0.0272	-7.8819	-0.49319
C57	ChABC	Before	CNTNAP2	ChABC	Before	-4.07839	2.058347	-1.98	0.0505	-8.1654	0.00859
C57	P	After	CNTNAP2	P	Before	-4.35	2.296182	-1.89	0.0612	-8.9092	0.20922
C57	P	Before	CNTNAP2	ChABC	Before	-3.29792	2.147881	-1.54	0.128	-7.5627	0.96684
CNTNAP2	ChABC	Before	CNTNAP2	P	After	3.29598	2.268448	1.45	0.1495	-1.2075	7.79943
C57	ChABC	After	C57	P	After	-2.60226	2.212656	-1.18	0.2425	-6.9956	1.79111
CNTNAP2	ChABC	Before	CNTNAP2	P	Before	-2.28458	2.147881	-1.06	0.2902	-6.5493	1.98018
C57	P	After	CNTNAP2	ChABC	After	2.12213	2.178649	0.97	0.3325	-2.2035	6.44779
C57	P	After	CNTNAP2	ChABC	Before	-2.06542	2.147881	-0.96	0.3387	-6.3302	2.19934
C57	ChABC	Before	C57	P	After	-2.01298	2.212656	-0.91	0.3653	-6.4064	2.3804
C57	ChABC	After	C57	P	Before	-1.36976	2.212656	-0.62	0.5374	-5.7631	3.02361
C57	P	After	C57	P	Before	1.2325	2.078754	0.59	0.5562	-2.952	5.417
C57	ChABC	After	CNTNAP2	P	After	-1.3717	2.329874	-0.59	0.5574	-5.9971	3.25373
C57	P	After	CNTNAP2	P	After	1.23056	2.409339	0.51	0.6107	-3.5527	6.01379
C57	P	Before	CNTNAP2	ChABC	After	0.88963	2.178649	0.41	0.6839	-3.436	5.21529

CNTNAP2	ChABC	After	CNTNAP2	P	After	-0.89157	2.297602	-0.39	0.6988	-5.4528	3.66962
C57	ChABC	Before	C57	P	Before	-0.78048	2.212656	-0.35	0.7251	-5.1739	3.6129
C57	ChABC	Before	CNTNAP2	P	After	-0.78242	2.329874	-0.34	0.7377	-5.4078	3.84301
C57	ChABC	After	C57	ChABC	Before	-0.58929	1.924552	-0.31	0.7608	-4.4634	3.28481
C57	ChABC	After	CNTNAP2	ChABC	After	-0.48013	2.090432	-0.23	0.8188	-4.6306	3.67036
C57	ChABC	Before	CNTNAP2	ChABC	After	0.10916	2.090432	0.05	0.9585	-4.0413	4.25964
C57	P	Before	CNTNAP2	P	After	-0.00194	2.409339	0	0.9994	-4.7852	4.78129

Mouse	treatment	Time				Least Squares Mean
CNTNAP2	P	Before	A			27.41083
CNTNAP2	ChABC	Before	A	B		25.12625
C57	P	After	A	B	C	23.06083
CNTNAP2	P	After		B	C	21.83027
C57	P	Before		B	C	21.82833
C57	ChABC	Before		B	C	21.04786
CNTNAP2	ChABC	After			C	20.9387
C57	ChABC	After			C	20.45857

S-FIG-22B

Source	Nparm	DFNum	DFDen	F Ratio	Prob > F
Mouse	1	1	23.6	3.866981	0.0611
treatment	1	1	23.6	9.580214	0.005
Mouse*trea	1	1	23.6	0.046619	0.8309
Time	1	1	23.4	10.92278	0.003
Mouse*Tim	1	1	23.4	18.74398	0.0002
treatment*	1	1	23.4	7.493617	0.0116
Mouse*trea	1	1	23.4	3.131557	0.0898

Mouse	treatment	Time	-Mouse	-treatment	-Time	Difference	Std Error	t Ratio	Prob>|t|	Lower 95%	Upper 95%
C57	ChABC	Before	CNTNAP2	P	Before	-14.3588	2.296181	-6.25	<.0001	-18.9815	-9.736
CNTNAP2	ChABC	After	CNTNAP2	P	Before	-12.8487	2.296181	-5.6	<.0001	-17.4715	-8.226
C57	ChABC	After	CNTNAP2	P	Before	-12.0725	2.296181	-5.26	<.0001	-16.6952	-7.44975
CNTNAP2	P	After	CNTNAP2	P	Before	-13.9329	2.681572	-5.2	<.0001	-19.437	-8.42876
CNTNAP2	ChABC	Before	CNTNAP2	P	Before	-9.26	2.296181	-4.03	0.0002	-13.8827	-4.63725
C57	P	Before	CNTNAP2	P	Before	-9.6933	2.454721	-3.95	0.0003	-14.6353	-4.75141
C57	P	After	CNTNAP2	P	Before	-9.6283	2.454721	-3.92	0.0003	-14.5703	-4.68641
C57	ChABC	Before	CNTNAP2	ChABC	Before	-5.0988	2.12585	-2.4	0.0206	-9.3786	-0.81892
C57	ChABC	Before	C57	P	After	-4.7304	2.296181	-2.06	0.0451	-9.3532	-0.10767
C57	ChABC	Before	C57	P	Before	-4.6654	2.296181	-2.03	0.048	-9.2882	-0.04267
CNTNAP2	ChABC	Before	CNTNAP2	P	After	4.6729	2.62901	1.78	0.0821	-0.6193	9.96506
CNTNAP2	ChABC	After	CNTNAP2	ChABC	Before	-3.5887	2.040511	-1.76	0.0926	-7.8223	0.64482
C57	P	After	CNTNAP2	P	After	4.3045	2.768556	1.55	0.1269	-1.2686	9.87768
C57	P	Before	CNTNAP2	P	After	4.2395	2.768556	1.53	0.1326	-1.3336	9.81268
C57	P	After	CNTNAP2	ChABC	After	3.2204	2.296181	1.4	0.1675	-1.4023	7.84317
C57	P	Before	CNTNAP2	ChABC	After	3.1554	2.296181	1.37	0.1761	-1.4673	7.77817
C57	ChABC	After	CNTNAP2	ChABC	Before	-2.8125	2.12585	-1.32	0.1924	-7.0923	1.46733
C57	ChABC	After	C57	ChABC	Before	2.2863	2.040511	1.12	0.2747	-1.9473	6.51982
C57	ChABC	After	C57	P	After	-2.4442	2.296181	-1.06	0.2927	-7.0669	2.17858
C57	ChABC	After	C57	P	Before	-2.3792	2.296181	-1.04	0.3056	-7.0019	2.24358
C57	ChABC	Before	CNTNAP2	ChABC	After	-1.51	2.12585	-0.71	0.4811	-5.7898	2.76983
C57	ChABC	After	CNTNAP2	P	After	1.8604	2.62901	0.71	0.4827	-3.4318	7.15256

CNTNAP2	ChABC	After	CNTNAP2	P	After	1.0841	2.62901	0.41	0.682	-4.208	6.37631
C57	ChABC	After	CNTNAP2	ChABC	After	0.7762	2.12585	0.37	0.7167	-3.5036	5.05608
C57	P	Before	CNTNAP2	ChABC	Before	-0.4333	2.296181	-0.19	0.8511	-5.0561	4.18942
C57	ChABC	Before	CNTNAP2	P	After	-0.4259	2.62901	-0.16	0.872	-5.718	4.86631
C57	P	After	CNTNAP2	ChABC	Before	-0.3683	2.296181	-0.16	0.8733	-4.9911	4.25442
C57	P	After	C57	P	Before	0.065	2.356179	0.03	0.9782	-4.8235	4.95351

Mouse	treatment	Time				Least Squares Mean
CNTNAP2	P	Before	A			32.105
CNTNAP2	ChABC	Before		B		22.845
C57	P	After		B		22.47667
C57	P	Before		B		22.41167
C57	ChABC	After		B	C	20.0325
CNTNAP2	ChABC	After		B	C	19.25625
CNTNAP2	P	After		B	C	18.17212
C57	ChABC	Before			C	17.74625

S-FIG-22C

Source	Nparm	DFNum	DFDen	F Ratio	Prob > F
Mouse	1	1	23.4	0.76142	0.3917
treatment	1	1	23.4	0.35079	0.5593
Mouse*trea	1	1	23.4	0.024083	0.878
Time	1	1	22.8	0.664537	0.4234
Mouse*Tim	1	1	22.8	0.021499	0.8847
treatment*	1	1	22.8	4.703768	0.0408
Mouse*trea	1	1	22.8	0.007363	0.9324

Mouse	treatment	Time	-Mouse	-treatment	-Time	Difference	Std Error	t Ratio	Prob>|t|	Lower 95%	Upper 95%
C57	ChABC	After	CNTNAP2	ChABC	Before	-6.38083	3.199768	-1.99	0.0527	-12.8402	0.0785
C57	P	Before	CNTNAP2	ChABC	Before	-6.1625	3.199768	-1.93	0.061	-12.6218	0.29683
CNTNAP2	ChABC	After	CNTNAP2	ChABC	Before	-4.60739	2.767785	-1.66	0.1103	-10.3509	1.13615
C57	ChABC	After	C57	ChABC	Before	-4.42333	3.055607	-1.45	0.1629	-10.7892	1.9425
CNTNAP2	ChABC	Before	CNTNAP2	P	Before	4.50866	3.210344	1.4	0.1675	-1.9689	10.98627
C57	ChABC	Before	C57	P	Before	4.205	3.420696	1.23	0.2259	-2.7003	11.11031
C57	P	After	CNTNAP2	ChABC	Before	-3.7625	3.199768	-1.18	0.2463	-10.2218	2.69683
C57	ChABC	After	CNTNAP2	P	After	-3.56875	3.430591	-1.04	0.3041	-10.4911	3.35362
C57	P	Before	CNTNAP2	P	After	-3.35041	3.430591	-0.98	0.3343	-10.2728	3.57195
CNTNAP2	ChABC	Before	CNTNAP2	P	After	2.81209	3.210344	0.88	0.386	-3.6655	9.2897
C57	ChABC	Before	CNTNAP2	ChABC	After	2.64989	3.301	0.8	0.4266	-4.0123	9.31203
C57	P	After	C57	P	Before	2.4	3.055607	0.79	0.4412	-3.9658	8.76583
C57	ChABC	After	C57	P	After	-2.61833	3.420696	-0.77	0.4483	-9.5236	4.28698
C57	ChABC	Before	CNTNAP2	P	Before	2.55116	3.430591	0.74	0.4612	-4.3712	9.47353
C57	ChABC	Before	CNTNAP2	ChABC	Before	-1.9575	3.199768	-0.61	0.544	-8.4168	4.50183
C57	ChABC	After	CNTNAP2	P	Before	-1.87217	3.430591	-0.55	0.5881	-8.7945	5.05019
CNTNAP2	ChABC	After	CNTNAP2	P	After	-1.7953	3.311253	-0.54	0.5905	-8.4756	4.88495
C57	ChABC	After	CNTNAP2	ChABC	After	-1.77344	3.301	-0.54	0.5939	-8.4356	4.8887
C57	ChABC	Before	C57	P	After	1.805	3.420696	0.53	0.6005	-5.1003	8.71031
CNTNAP2	P	After	CNTNAP2	P	Before	1.69658	3.209617	0.53	0.6011	-4.8691	8.26226
C57	P	Before	CNTNAP2	P	Before	-1.65384	3.430591	-0.48	0.6322	-8.5762	5.26853
C57	P	Before	CNTNAP2	ChABC	After	-1.55511	3.301	-0.47	0.64	-8.2173	5.10703

C57	P	After	CNTNAP2	P	After	-0.95041	3.430591	-0.28	0.7831	-7.8728	5.97195
C57	P	After	CNTNAP2	ChABC	After	0.84489	3.301	0.26	0.7992	-5.8173	7.50703
C57	ChABC	Before	CNTNAP2	P	After	0.85459	3.430591	0.25	0.8045	-6.0678	7.77695
C57	P	After	CNTNAP2	P	Before	0.74616	3.430591	0.22	0.8289	-6.1762	7.66853
C57	ChABC	After	C57	P	Before	-0.21833	3.420696	-0.06	0.9494	-7.1236	6.68698
CNTNAP2	ChABC	After	CNTNAP2	P	Before	-0.09873	3.311253	-0.03	0.9764	-6.779	6.58152

Mouse	treatment	Time		Least Squares Mean
CNTNAP2	ChABC	Before	A	27.4075
C57	ChABC	Before	A	25.45
CNTNAP2	P	After	A	24.59541
C57	P	After	A	23.645
CNTNAP2	P	Before	A	22.89884
CNTNAP2	ChABC	After	A	22.80011
C57	P	Before	A	21.245
C57	ChABC	After	A	21.02667

S-FIG-23A

Source	Nparm	DFNum	DFDen	F Ratio	Prob > F
Mouse	1	1	49.7	0.364173	0.5489
treatment	1	1	49.7	3.084804	0.0852
Mouse*trea	1	1	49.7	0.69658	0.4079
Time	1	1	49.1	0.560782	0.4575
Mouse*Tim	1	1	49.1	4.737639	0.0343
treatment*	1	1	49.1	0.123416	0.7269
Mouse*trea	1	1	49.1	0.01431	0.9053

Mouse	treatment	Time	-Mouse	-treatment	-Time	Difference	Std Error	t Ratio	Prob>|t|	Lower 95%	Upper 95%
C57	ChABC	Before	CNTNAP2	P	Before	-5.77381	2.732557	-2.11	0.0372	-11.1991	-0.3485
CNTNAP2	ChABC	After	CNTNAP2	P	Before	-5.61817	2.690586	-2.09	0.0395	-10.9599	-0.2764
C57	ChABC	Before	C57	P	After	-5.02381	2.732557	-1.84	0.0691	-10.4491	0.4015
C57	P	After	CNTNAP2	ChABC	After	4.86817	2.690586	1.81	0.0736	-0.4736	10.2099
C57	P	Before	CNTNAP2	P	Before	-5	2.835708	-1.76	0.0811	-10.6301	0.6301
C57	P	After	C57	P	Before	4.25	2.581433	1.65	0.1063	-0.9416	9.4416
C57	ChABC	Before	CNTNAP2	P	After	-4.27713	2.87742	-1.49	0.1405	-9.9893	1.4351
CNTNAP2	ChABC	After	CNTNAP2	P	After	-4.12149	2.837594	-1.45	0.1497	-9.7545	1.5115
CNTNAP2	ChABC	Before	CNTNAP2	P	Before	-3.54167	2.652562	-1.34	0.185	-8.8082	1.7248
C57	ChABC	After	C57	ChABC	Before	3.07143	2.389942	1.29	0.205	-1.7351	7.8779
C57	P	Before	CNTNAP2	P	After	-3.50332	2.975554	-1.18	0.242	-9.4104	2.4037
C57	ChABC	After	CNTNAP2	ChABC	After	2.91579	2.581643	1.13	0.2616	-2.2097	8.0413
C57	P	After	CNTNAP2	ChABC	Before	2.79167	2.652562	1.05	0.2953	-2.4748	8.0582
C57	ChABC	After	CNTNAP2	P	Before	-2.70238	2.732557	-0.99	0.3252	-8.1277	2.7229
CNTNAP2	ChABC	After	CNTNAP2	ChABC	Before	-2.0765	2.280574	-0.91	0.367	-6.6593	2.5063
C57	ChABC	Before	CNTNAP2	ChABC	Before	-2.23214	2.541989	-0.88	0.3821	-7.2791	2.8148
C57	ChABC	After	C57	P	Before	2.29762	2.732557	0.84	0.4026	-3.1277	7.7229
CNTNAP2	ChABC	Before	CNTNAP2	P	After	-2.04498	2.801565	-0.73	0.4672	-7.6066	3.5166
C57	ChABC	After	C57	P	After	-1.95238	2.732557	-0.71	0.4767	-7.3777	3.4729
C57	P	Before	CNTNAP2	ChABC	Before	-1.45833	2.652562	-0.55	0.5838	-6.7248	3.8082

CNTNAP2	P	After	CNTNAP2	P	Before	-1.49668	2.734315	-0.55	0.5865	-6.9837	3.9903
C57	ChABC	After	CNTNAP2	P	After	-1.2057	2.87742	-0.42	0.6761	-6.9179	4.5065
C57	ChABC	After	CNTNAP2	ChABC	Before	0.83929	2.541989	0.33	0.742	-4.2077	5.8862
C57	ChABC	Before	C57	P	Before	-0.77381	2.732557	-0.28	0.7777	-6.1991	4.6515
C57	P	After	CNTNAP2	P	Before	-0.75	2.835708	-0.26	0.792	-6.3801	4.8801
C57	P	After	CNTNAP2	P	After	0.74668	2.975554	0.25	0.8024	-5.1604	6.6537
C57	P	Before	CNTNAP2	ChABC	After	0.61817	2.690586	0.23	0.8188	-4.7236	5.9599
C57	ChABC	Before	CNTNAP2	ChABC	After	-0.15564	2.581643	-0.06	0.9521	-5.2811	4.9698

Mouse	treatment	Time			Least Squares Mean
CNTNAP2	P	Before	A		28.91667
C57	P	After	A	B	28.16667
CNTNAP2	P	After	A	B	27.41999
C57	ChABC	After	A	B	26.21429
CNTNAP2	ChABC	Before	A	B	25.375
C57	P	Before	A	B	23.91667
CNTNAP2	ChABC	After		B	23.2985
C57	ChABC	Before		B	23.14286

S-FIG-23B

Source	Nparm	DFNum	DFDen	F Ratio	Prob > F
Mouse	1	1	25.1	0.099325	0.7552
treatment	1	1	25.1	1.368555	0.253
Mouse*trea	1	1	25.1	0.284536	0.5984
Time	1	1	24.1	0.178347	0.6765
Mouse*Tim	1	1	24.1	1.29664	0.266
treatment*	1	1	24.1	0.449292	0.509
Mouse*trea	1	1	24.1	0.860827	0.3627

Mouse	treatment	Time	-Mouse	-treatment	-Time	Difference	Std Error	t Ratio	Prob>|t|	Lower 95%	Upper 95%
CNTNAP2	ChABC	After	CNTNAP2	P	Before	-6.375	3.844072	-1.66	0.1052	-14.1491	1.39909
C57	P	Before	CNTNAP2	ChABC	After	5.54167	3.844072	1.44	0.1574	-2.2324	13.31576
C57	ChABC	After	CNTNAP2	ChABC	After	5.125	3.558919	1.44	0.1578	-2.0724	12.32241
C57	ChABC	After	C57	ChABC	Before	3.5	2.657707	1.32	0.2009	-1.9989	8.99887
C57	ChABC	Before	CNTNAP2	P	Before	-4.75	3.844072	-1.24	0.2239	-12.5241	3.02409
C57	P	After	CNTNAP2	ChABC	After	4.20833	3.844072	1.09	0.2803	-3.5658	11.98243
CNTNAP2	ChABC	After	CNTNAP2	P	After	-4.40384	4.289863	-1.03	0.3103	-13.0539	4.24624
CNTNAP2	ChABC	After	CNTNAP2	ChABC	Before	-2.75	2.657707	-1.03	0.3116	-8.2489	2.74887
C57	ChABC	Before	C57	P	Before	-3.91667	3.844072	-1.02	0.3145	-11.6908	3.85743
CNTNAP2	ChABC	Before	CNTNAP2	P	Before	-3.625	3.844072	-0.94	0.3515	-11.3991	4.14909
C57	P	Before	CNTNAP2	ChABC	Before	2.79167	3.844072	0.73	0.472	-4.9824	10.56576
C57	ChABC	Before	C57	P	After	-2.58333	3.844072	-0.67	0.5055	-10.3574	5.19076
C57	ChABC	After	CNTNAP2	ChABC	Before	2.375	3.558919	0.67	0.5085	-4.8224	9.57241
C57	ChABC	Before	CNTNAP2	P	After	-2.77884	4.289863	-0.65	0.5206	-11.4289	5.87124
CNTNAP2	P	After	CNTNAP2	P	Before	-1.97116	3.611635	-0.55	0.5898	-9.3881	5.44582
C57	P	After	CNTNAP2	P	Before	-2.16667	4.109486	-0.53	0.601	-10.4775	6.14419
C57	ChABC	Before	CNTNAP2	ChABC	After	1.625	3.558919	0.46	0.6505	-5.5724	8.82241
C57	P	After	C57	P	Before	-1.33333	3.068856	-0.43	0.668	-7.6829	5.01621
CNTNAP2	ChABC	Before	CNTNAP2	P	After	-1.65384	4.289863	-0.39	0.7017	-10.3039	6.99624
C57	P	After	CNTNAP2	ChABC	Before	1.45833	3.844072	0.38	0.7065	-6.3158	9.23243
C57	ChABC	After	CNTNAP2	P	Before	-1.25	3.844072	-0.33	0.7468	-9.0241	6.52409
C57	ChABC	Before	CNTNAP2	ChABC	Before	-1.125	3.558919	-0.32	0.7536	-8.3224	6.07241

C57	P	Before	CNTNAP2	P	After	1.13783	4.529228	0.25	0.8028	-7.9971	10.27275
C57	ChABC	After	C57	P	After	0.91667	3.844072	0.24	0.8128	-6.8574	8.69076
C57	P	Before	CNTNAP2	P	Before	-0.83333	4.109486	-0.2	0.8404	-9.1442	7.47752
C57	ChABC	After	CNTNAP2	P	After	0.72116	4.289863	0.17	0.8673	-7.9289	9.37124
C57	ChABC	After	C57	P	Before	-0.41667	3.844072	-0.11	0.9142	-8.1908	7.35743
C57	P	After	CNTNAP2	P	After	-0.1955	4.529228	-0.04	0.9658	-9.3304	8.93942

Mouse	treatment	Time		Least Squares Mean
CNTNAP2	P	Before	A	27.5
C57	P	Before	A	26.66667
C57	ChABC	After	A	26.25
CNTNAP2	P	After	A	25.52884
C57	P	After	A	25.33333
CNTNAP2	ChABC	Before	A	23.875
C57	ChABC	Before	A	22.75
CNTNAP2	ChABC	After	A	21.125

S-FIG-23C

Source	Nparm	DFNum	DFDen	F Ratio	Prob > F
Mouse	1	1	23.5	1.583584	0.2206
treatment	1	1	23.5	1.463603	0.2384
Mouse*trea	1	1	23.5	0.328486	0.572
Time	1	1	23.8	1.29193	0.267
Mouse*Tim	1	1	23.8	3.773176	0.064
treatment*	1	1	23.8	0.745289	0.3966
Mouse*trea	1	1	23.8	0.936083	0.343

Mouse	treatment	Time	-Mouse	-treatment	-Time	Difference	Std Error	t Ratio	Prob>|t|	Lower 95%	Upper 95%
C57	P	Before	CNTNAP2	P	Before	-9.18425	3.943179	-2.33	0.0246	-17.1375	-1.231
C57	P	After	C57	P	Before	9.83333	4.11637	2.39	0.0261	1.2874	18.3793
C57	ChABC	Before	C57	P	After	-7.33333	3.925122	-1.87	0.0686	-15.2509	0.5843
C57	P	Before	CNTNAP2	P	After	-7.36165	3.943179	-1.87	0.0688	-15.3149	0.5916
C57	ChABC	Before	CNTNAP2	P	Before	-6.68425	3.943179	-1.7	0.0973	-14.6375	1.269
C57	P	Before	CNTNAP2	ChABC	Before	-5.70833	3.671615	-1.55	0.1274	-13.1146	1.6979
C57	P	After	CNTNAP2	ChABC	After	5.53018	3.791321	1.46	0.152	-2.1171	13.1774
CNTNAP2	ChABC	After	CNTNAP2	P	Before	-4.88109	3.810013	-1.28	0.207	-12.5654	2.8032
C57	ChABC	After	C57	P	Before	5	3.925122	1.27	0.2096	-2.9176	12.9176
C57	ChABC	Before	CNTNAP2	P	After	-4.86165	3.943179	-1.23	0.2243	-12.8149	3.0916
C57	ChABC	After	C57	P	After	-4.83333	3.925122	-1.23	0.2249	-12.7509	3.0843
C57	P	Before	CNTNAP2	ChABC	After	-4.30316	3.791321	-1.14	0.2627	-11.9504	3.3441
C57	P	After	CNTNAP2	ChABC	Before	4.125	3.671615	1.12	0.2675	-3.2812	11.5312
C57	ChABC	After	CNTNAP2	P	Before	-4.18425	3.943179	-1.06	0.2946	-12.1375	3.769
CNTNAP2	ChABC	Before	CNTNAP2	P	Before	-3.47591	3.690913	-0.94	0.3516	-10.9202	3.9684
C57	ChABC	Before	CNTNAP2	ChABC	Before	-3.20833	3.671615	-0.87	0.3871	-10.6146	4.1979
CNTNAP2	ChABC	After	CNTNAP2	P	After	-3.05849	3.810013	-0.8	0.4265	-10.7428	4.6258
C57	ChABC	Before	C57	P	Before	2.5	3.925122	0.64	0.5276	-5.4176	10.4176
C57	P	After	CNTNAP2	P	After	2.47169	3.943179	0.63	0.5341	-5.4815	10.4249
C57	ChABC	After	C57	ChABC	Before	2.5	4.11637	0.61	0.55	-6.046	11.046
C57	ChABC	After	CNTNAP2	P	After	-2.36165	3.943179	-0.6	0.5524	-10.3149	5.5916
C57	ChABC	Before	CNTNAP2	ChABC	After	-1.80316	3.791321	-0.48	0.6368	-9.4504	5.8441

CNTNAP2	ChABC	Before	CNTNAP2	P	After	-1.65331	3.690913	-0.45	0.6565	-9.0976	5.791
CNTNAP2	P	After	CNTNAP2	P	Before	-1.8226	4.084787	-0.45	0.6587	-10.1699	6.5247
CNTNAP2	ChABC	After	CNTNAP2	ChABC	Before	-1.40518	3.688053	-0.38	0.7067	-9.0328	6.2224
C57	ChABC	After	CNTNAP2	ChABC	Before	-0.70833	3.671615	-0.19	0.8479	-8.1146	6.6979
C57	ChABC	After	CNTNAP2	ChABC	After	0.69684	3.791321	0.18	0.855	-6.9504	8.3441
C57	P	After	CNTNAP2	P	Before	0.64909	3.943179	0.16	0.87	-7.3041	8.6023

Mouse	treatment	Time			Least Squares Mean
C57	P	After	A		31
CNTNAP2	P	Before	A		30.35091
CNTNAP2	P	After	A	B	28.52831
CNTNAP2	ChABC	Before	A	B	26.875
C57	ChABC	After	A	B	26.16667
CNTNAP2	ChABC	After	A	B	25.46982
C57	ChABC	Before	A	B	23.66667
C57	P	Before		B	21.16667

S-FIG-24A

Source	Nparm	DFNum	DFDen	F Ratio	Prob > F
Mouse	1	1	49.8	0.228636	0.6346
treatment	1	1	49.8	2.959284	0.0916
Mouse*trea	1	1	49.8	0.446893	0.5069
Time	1	1	49.3	1.346787	0.2514
Mouse*Tim	1	1	49.3	5.595687	0.022
treatment*	1	1	49.3	0.073839	0.787
Mouse*trea	1	1	49.3	0.1879	0.6666

Mouse	treatment	Time	-Mouse	-treatment	-Time	Difference	Std Error	t Ratio	Prob>|t|	Lower 95%	Upper 95%
C57	ChABC	Before	C57	P	After	-5.92857	2.721267	-2.18	0.0319	-11.3316	-0.5255
C57	ChABC	Before	CNTNAP2	P	Before	-5.84524	2.721267	-2.15	0.0343	-11.2483	-0.4422
C57	P	After	C57	P	Before	5.25	2.559349	2.05	0.0458	0.1032	10.3968
C57	P	After	CNTNAP2	ChABC	After	5.10696	2.679358	1.91	0.0597	-0.2127	10.4266
CNTNAP2	ChABC	After	CNTNAP2	P	Before	-5.02362	2.679358	-1.87	0.0639	-10.3432	0.296
C57	P	Before	CNTNAP2	P	Before	-5.16667	2.823992	-1.83	0.0705	-10.7737	0.4403
C57	ChABC	After	C57	ChABC	Before	3.5	2.369496	1.48	0.1462	-1.265	8.265
C57	ChABC	Before	CNTNAP2	P	After	-4.14941	2.865114	-1.45	0.1508	-9.8373	1.5385
C57	P	After	CNTNAP2	ChABC	Before	3.8125	2.641603	1.44	0.1523	-1.4324	9.0574
CNTNAP2	ChABC	Before	CNTNAP2	P	Before	-3.72917	2.641603	-1.41	0.1613	-8.974	1.5157
CNTNAP2	ChABC	After	CNTNAP2	P	After	-3.3278	2.82534	-1.18	0.2418	-8.9365	2.2809
C57	P	Before	CNTNAP2	P	After	-3.47084	2.962856	-1.17	0.2443	-9.3528	2.4111
C57	ChABC	After	CNTNAP2	ChABC	After	2.67839	2.57086	1.04	0.3002	-2.4258	7.7826
C57	ChABC	After	C57	P	Before	2.82143	2.721267	1.04	0.3025	-2.5816	8.2245
C57	ChABC	After	C57	P	After	-2.42857	2.721267	-0.89	0.3744	-7.8316	2.9745
C57	ChABC	After	CNTNAP2	P	Before	-2.34524	2.721267	-0.86	0.391	-7.7483	3.0578
C57	ChABC	Before	CNTNAP2	ChABC	Before	-2.11607	2.531487	-0.84	0.4053	-7.1423	2.9102
CNTNAP2	ChABC	Before	CNTNAP2	P	After	-2.03334	2.789561	-0.73	0.4678	-7.5712	3.5045
CNTNAP2	P	After	CNTNAP2	P	Before	-1.69582	2.711798	-0.63	0.5345	-7.1374	3.7457
C57	P	After	CNTNAP2	P	After	1.77916	2.962856	0.6	0.5496	-4.1028	7.6611
CNTNAP2	ChABC	After	CNTNAP2	ChABC	Before	-1.29446	2.261326	-0.57	0.5696	-5.8383	3.2494
C57	ChABC	After	CNTNAP2	ChABC	Before	1.38393	2.531487	0.55	0.5859	-3.6423	6.4102

C57	P	Before	CNTNAP2	ChABC	Before	-1.4375	2.641603	-0.54	0.5876	-6.6824	3.8074
C57	ChABC	Before	CNTNAP2	ChABC	After	-0.82161	2.57086	-0.32	0.75	-5.9258	4.2826
C57	ChABC	Before	C57	P	Before	-0.67857	2.721267	-0.25	0.8036	-6.0816	4.7245
C57	ChABC	After	CNTNAP2	P	After	-0.64941	2.865114	-0.23	0.8212	-6.3373	5.0385
C57	P	Before	CNTNAP2	ChABC	After	-0.14304	2.679358	-0.05	0.9575	-5.4627	5.1766
C57	P	After	CNTNAP2	P	Before	0.08333	2.823992	0.03	0.9765	-5.5237	5.6903

Mouse	treatment	Time				Least Squares Mean
C57	P	After	A			29
CNTNAP2	P	Before	A	B		28.91667
CNTNAP2	P	After	A	B	C	27.22084
C57	ChABC	After	A	B	C	26.57143
CNTNAP2	ChABC	Before	A	B	C	25.1875
CNTNAP2	ChABC	After	A	B	C	23.89304
C57	P	Before		B	C	23.75
C57	ChABC	Before			C	23.07143

S-FIG-24B

Source	Nparm	DFNum	DFDen	F Ratio	Prob > F
Mouse	1	1	25.1	0.154555	0.6975
treatment	1	1	25.1	1.766566	0.1958
Mouse*trea	1	1	25.1	0.323493	0.5746
Time	1	1	24	0.204964	0.6548
Mouse*Tim	1	1	24	1.499586	0.2326
treatment*	1	1	24	0.346847	0.5614
Mouse*trea	1	1	24	0.826923	0.3722

Mouse	treatment	Time	-Mouse	-treatment	-Time	Difference	Std Error	t Ratio	Prob>|t|	Lower 95%	Upper 95%
C57	ChABC	After	C57	ChABC	Before	3.125	2.415727	1.29	0.2087	-1.8744	8.12439
C57	ChABC	After	C57	P	After	0.33333	3.655403	0.09	0.9278	-7.0674	7.73404
C57	ChABC	After	C57	P	Before	-0.66667	3.655403	-0.18	0.8563	-8.0674	6.73404
C57	ChABC	After	CNTNAP2	ChABC	After	5.125	3.384246	1.51	0.1382	-1.7267	11.97672
C57	ChABC	After	CNTNAP2	ChABC	Before	2.375	3.384246	0.7	0.4871	-4.4767	9.22672
C57	ChABC	After	CNTNAP2	P	After	0.36785	4.055565	0.09	0.9282	-7.8142	8.54988
C57	ChABC	After	CNTNAP2	P	Before	-1.5	3.655403	-0.41	0.6839	-8.9007	5.90071
C57	ChABC	Before	C57	P	After	-2.79167	3.655403	-0.76	0.4498	-10.1924	4.60904
C57	ChABC	Before	C57	P	Before	-3.79167	3.655403	-1.04	0.3062	-11.1924	3.60904
C57	ChABC	Before	CNTNAP2	ChABC	After	2	3.384246	0.59	0.558	-4.8517	8.85172
C57	ChABC	Before	CNTNAP2	ChABC	Before	-0.75	3.384246	-0.22	0.8258	-7.6017	6.10172
C57	ChABC	Before	CNTNAP2	P	After	-2.75715	4.055565	-0.68	0.5003	-10.9392	5.42488
C57	ChABC	Before	CNTNAP2	P	Before	-4.625	3.655403	-1.27	0.2135	-12.0257	2.77571
C57	P	After	C57	P	Before	-1	2.789441	-0.36	0.7233	-6.7728	4.77279
C57	P	After	CNTNAP2	ChABC	After	4.79167	3.655403	1.31	0.1978	-2.609	12.19237
C57	P	After	CNTNAP2	ChABC	Before	2.04167	3.655403	0.56	0.5798	-5.359	9.44237
C57	P	After	CNTNAP2	P	After	0.03452	4.284444	0.01	0.9936	-8.6118	8.68084
C57	P	After	CNTNAP2	P	Before	-1.83333	3.907791	-0.47	0.6417	-9.745	6.07836
C57	P	Before	CNTNAP2	ChABC	After	5.79167	3.655403	1.58	0.1214	-1.609	13.19237
C57	P	Before	CNTNAP2	ChABC	Before	3.04167	3.655403	0.83	0.4106	-4.359	10.44237
C57	P	Before	CNTNAP2	P	After	1.03452	4.284444	0.24	0.8104	-7.6118	9.68084
C57	P	Before	CNTNAP2	P	Before	-0.83333	3.907791	-0.21	0.8323	-8.745	7.07836

CNTNAP2	ChABC	After	CNTNAP2	ChABC	Before	-2.75	2.415727	-1.14	0.2668	-7.7494	2.24939
CNTNAP2	ChABC	After	CNTNAP2	P	After	-4.75715	4.055565	-1.17	0.2473	-12.9392	3.42488
CNTNAP2	ChABC	After	CNTNAP2	P	Before	-6.625	3.655403	-1.81	0.0779	-14.0257	0.77571
CNTNAP2	ChABC	Before	CNTNAP2	P	After	-2.00715	4.055565	-0.49	0.6232	-10.1892	6.17488
CNTNAP2	ChABC	Before	CNTNAP2	P	Before	-3.875	3.655403	-1.06	0.2958	-11.2757	3.52571
CNTNAP2	P	After	CNTNAP2	P	Before	-1.86785	3.296455	-0.57	0.5758	-8.6418	4.90614

Mouse	treatment	Time		Least Squares Mean
CNTNAP2	P	Before	A	27.5
C57	P	Before	A	26.66667
C57	ChABC	After	A	26
C57	P	After	A	25.66667
CNTNAP2	P	After	A	25.63215
CNTNAP2	ChABC	Before	A	23.625
C57	ChABC	Before	A	22.875
CNTNAP2	ChABC	After	A	20.875

S-FIG-24C

Source	Nparm	DFNum	DFDen	F Ratio	Prob > F
Mouse	1	1	23.7	1.22211	0.28
treatment	1	1	23.7	0.864866	0.3617
Mouse*trea	1	1	23.7	0.071816	0.791
Time	1	1	23.9	2.822501	0.106
Mouse*Tim	1	1	23.9	4.723758	0.0399
treatment*	1	1	23.9	0.364432	0.5517
Mouse*trea	1	1	23.9	1.589846	0.2195

Mouse	treatment	Time	-Mouse	-treatment	-Time	Difference	Std Error	t Ratio	Prob>|t|	Lower 95%	Upper 95%
C57	P	After	C57	P	Before	11.5	4.127428	2.79	0.0108	2.9339	20.0661
C57	P	Before	CNTNAP2	P	Before	-9.5005	4.007492	-2.37	0.0223	-17.5829	-1.4181
C57	ChABC	Before	C57	P	After	-9	3.988282	-2.26	0.0292	-17.0441	-0.9559
C57	P	Before	CNTNAP2	P	After	-7.1864	4.007492	-1.79	0.08	-15.2688	0.896
C57	ChABC	Before	CNTNAP2	P	Before	-7.0005	4.007492	-1.75	0.0878	-15.0829	1.0819
C57	ChABC	After	C57	P	Before	6.5	3.988282	1.63	0.1105	-1.5441	14.5441
C57	P	Before	CNTNAP2	ChABC	After	-6.2461	3.852793	-1.62	0.1123	-14.0167	1.5244
C57	P	Before	CNTNAP2	ChABC	Before	-5.9167	3.730696	-1.59	0.1201	-13.4412	1.6079
C57	P	After	CNTNAP2	ChABC	Before	5.5833	3.730696	1.5	0.1418	-1.9412	13.1079
C57	P	After	CNTNAP2	ChABC	After	5.2539	3.852793	1.36	0.1798	-2.5167	13.0244
C57	ChABC	After	C57	P	After	-5	3.988282	-1.25	0.2168	-13.0441	3.0441
C57	ChABC	Before	CNTNAP2	P	After	-4.6864	4.007492	-1.17	0.2487	-12.7688	3.396
C57	P	After	CNTNAP2	P	After	4.3136	4.007492	1.08	0.2878	-3.7688	12.396
C57	ChABC	Before	CNTNAP2	ChABC	After	-3.7461	3.852793	-0.97	0.3363	-11.5167	4.0244
C57	ChABC	After	C57	ChABC	Before	4	4.127428	0.97	0.3431	-4.5661	12.5661
CNTNAP2	ChABC	Before	CNTNAP2	P	Before	-3.5838	3.751225	-0.96	0.3447	-11.1493	3.9817
C57	ChABC	Before	CNTNAP2	ChABC	Before	-3.4167	3.730696	-0.92	0.3649	-10.9412	4.1079
CNTNAP2	ChABC	After	CNTNAP2	P	Before	-3.2543	3.872675	-0.84	0.4054	-11.0647	4.556
C57	ChABC	After	CNTNAP2	P	Before	-3.0005	4.007492	-0.75	0.4581	-11.0829	5.0819
C57	ChABC	Before	C57	P	Before	2.5	3.988282	0.63	0.5341	-5.5441	10.5441
CNTNAP2	P	After	CNTNAP2	P	Before	-2.3141	4.116287	-0.56	0.5782	-10.7233	6.0952
C57	P	After	CNTNAP2	P	Before	1.9995	4.007492	0.5	0.6204	-6.0829	10.0819
CNTNAP2	ChABC	Before	CNTNAP2	P	After	-1.2697	3.751225	-0.34	0.7366	-8.8352	6.2958

CNTNAP2	ChABC	After	CNTNAP2	P	After	-0.9403	3.872675	-0.24	0.8093	-8.7506	6.8701
C57	ChABC	After	CNTNAP2	P	After	-0.6864	4.007492	-0.17	0.8648	-8.7688	7.396
C57	ChABC	After	CNTNAP2	ChABC	Before	0.5833	3.730696	0.16	0.8765	-6.9412	8.1079
CNTNAP2	ChABC	After	CNTNAP2	ChABC	Before	0.3295	3.701711	0.09	0.9298	-7.3242	7.9831
C57	ChABC	After	CNTNAP2	ChABC	After	0.2539	3.852793	0.07	0.9478	-7.5167	8.0244

Mouse	treatment	Time				Least Squares Mean
C57	P	After	A			32.33333
CNTNAP2	P	Before	A	B		30.3338
CNTNAP2	P	After	A	B	C	28.01973
C57	ChABC	After	A	B	C	27.33333
CNTNAP2	ChABC	After	A	B	C	27.07947
CNTNAP2	ChABC	Before	A	B	C	26.75
C57	ChABC	Before		B	C	23.33333
C57	P	Before			C	20.83333

S-FIG-25A

Source	Nparm	DFNum	DFDen	F Ratio	Prob > F
Mouse	1	1	49.7	29.7181	<.0001
treatment	1	1	49.7	4.265547	0.0441
Mouse*trea	1	1	49.7	0.234089	0.6306
Time	1	1	47.6	38.08325	<.0001
Mouse*Tim	1	1	47.6	1.505718	0.2258
treatment*	1	1	47.6	0.000603	0.9805
Mouse*trea	1	1	47.6	5.532834	0.0228

Mouse	treatment	Time	-Mouse	-treatment	-Time	Difference	Std Error	t Ratio	Prob>|t|	Lower 95%	Upper 95%
C57	ChABC	After	CNTNAP2	P	Before	-7.7107	1.301601	-5.92	<.0001	-10.3086	-5.11276
C57	P	After	CNTNAP2	P	Before	-7.27883	1.350735	-5.39	<.0001	-9.9749	-4.58282
C57	ChABC	After	CNTNAP2	ChABC	Before	-6.45185	1.210828	-5.33	<.0001	-8.8686	-4.03508
C57	ChABC	Before	CNTNAP2	P	Before	-6.7497	1.301601	-5.19	<.0001	-9.3476	-4.15176
CNTNAP2	ChABC	After	CNTNAP2	ChABC	Before	-3.64058	0.682003	-5.34	<.0001	-5.0122	-2.26899
C57	P	After	CNTNAP2	ChABC	Before	-6.01998	1.263497	-4.76	<.0001	-8.5419	-3.49809
C57	ChABC	Before	CNTNAP2	ChABC	Before	-5.49085	1.210828	-4.53	<.0001	-7.9076	-3.07408
C57	ChABC	After	CNTNAP2	P	After	-5.81268	1.339733	-4.34	<.0001	-8.4835	-3.14185
C57	P	After	CNTNAP2	P	After	-5.38081	1.387518	-3.88	0.0002	-8.1471	-2.61451
CNTNAP2	ChABC	After	CNTNAP2	P	Before	-4.89944	1.273423	-3.85	0.0003	-7.4403	-2.35862
C57	ChABC	Before	CNTNAP2	P	After	-4.85168	1.339733	-3.62	0.0005	-7.5225	-2.18085
C57	P	After	C57	P	Before	-2.74033	0.765896	-3.58	0.0008	-4.2813	-1.19932
C57	P	Before	CNTNAP2	P	Before	-4.5385	1.350735	-3.36	0.0013	-7.2345	-1.84248
C57	P	Before	CNTNAP2	ChABC	Before	-3.27965	1.263497	-2.6	0.0116	-5.8015	-0.75775
C57	ChABC	After	C57	P	Before	-3.1722	1.301601	-2.44	0.0175	-5.7701	-0.57426
C57	ChABC	After	CNTNAP2	ChABC	After	-2.81127	1.221182	-2.3	0.0244	-5.2478	-0.37476
CNTNAP2	ChABC	After	CNTNAP2	P	After	-3.00141	1.312374	-2.29	0.0251	-5.6168	-0.38602
CNTNAP2	P	After	CNTNAP2	P	Before	-1.89802	0.829046	-2.29	0.0264	-3.5641	-0.23198
C57	P	Before	CNTNAP2	P	After	-2.64048	1.387518	-1.9	0.0611	-5.4068	0.12582
C57	P	After	CNTNAP2	ChABC	After	-2.3794	1.273423	-1.87	0.066	-4.9202	0.16142
C57	ChABC	Before	C57	P	Before	-2.2112	1.301601	-1.7	0.094	-4.8091	0.38674
C57	ChABC	Before	CNTNAP2	ChABC	After	-1.85027	1.221182	-1.52	0.1343	-4.2868	0.58624
C57	ChABC	After	C57	ChABC	Before	-0.961	0.709082	-1.36	0.1818	-2.3877	0.4657

CNTNAP2	ChABC	Before	CNTNAP2	P	Before	-1.25885	1.263497	-1	0.3227	-3.7807	1.26304
CNTNAP2	ChABC	Before	CNTNAP2	P	After	0.63917	1.302745	0.49	0.6252	-1.9577	3.23609
C57	ChABC	Before	C57	P	After	0.52913	1.301601	0.41	0.6857	-2.0688	3.12708
C57	ChABC	After	C57	P	After	-0.43187	1.301601	-0.33	0.7411	-3.0298	2.16608
C57	P	Before	CNTNAP2	ChABC	After	0.36094	1.273423	0.28	0.7777	-2.1799	2.90175

Mouse	treatment	Time							Least Squares Mean
CNTNAP2	P	Before	A						21.75392
CNTNAP2	ChABC	Before	A	B					20.49506
CNTNAP2	P	After		B	C				19.85589
C57	P	Before			C	D			17.21542
CNTNAP2	ChABC	After				D	E		16.85448
C57	ChABC	Before				D	E	F	15.00421
C57	P	After					E	F	14.47508
C57	ChABC	After						F	14.04321

S-FIG-25B

Source	Nparm	DFNum	DFDen	F Ratio	Prob > F
Mouse	1	1	50.4	23.33812	<.0001
treatment	1	1	50.4	1.99573	0.1639
Mouse*trea	1	1	50.4	0.016053	0.8997
Time	1	1	48.6	24.77634	<.0001
Mouse*Tim	1	1	48.6	2.251225	0.14
treatment*	1	1	48.6	0.451767	0.5047
Mouse*trea	1	1	48.6	5.436456	0.0239

Mouse	treatment	Time	-Mouse	-treatment	-Time	Difference	Std Error	t Ratio	Prob>|t|	Lower 95%	Upper 95%
C57	ChABC	After	CNTNAP2	ChABC	Before	-0.02578	0.005005	-5.15	<.0001	-0.03575	-0.0158
CNTNAP2	ChABC	After	CNTNAP2	ChABC	Before	-0.01677	0.003222	-5.2	<.0001	-0.02324	-0.01029
C57	ChABC	After	CNTNAP2	P	Before	-0.02563	0.00538	-4.76	<.0001	-0.03635	-0.01491
C57	P	After	CNTNAP2	ChABC	Before	-0.0244	0.005223	-4.67	<.0001	-0.0348	-0.01399
C57	ChABC	Before	CNTNAP2	ChABC	Before	-0.02256	0.005005	-4.51	<.0001	-0.03254	-0.01259
C57	P	After	CNTNAP2	P	Before	-0.02425	0.005583	-4.34	<.0001	-0.03538	-0.01313
C57	ChABC	Before	CNTNAP2	P	Before	-0.02242	0.00538	-4.17	<.0001	-0.03314	-0.0117
C57	ChABC	After	CNTNAP2	P	After	-0.01949	0.005575	-3.5	0.0008	-0.03058	-0.00839
CNTNAP2	ChABC	After	CNTNAP2	P	Before	-0.01662	0.005273	-3.15	0.0023	-0.02713	-0.00612
C57	P	After	CNTNAP2	P	After	-0.01811	0.005771	-3.14	0.0024	-0.0296	-0.00662
C57	P	Before	CNTNAP2	ChABC	Before	-0.01531	0.005223	-2.93	0.0045	-0.02572	-0.00491
C57	ChABC	Before	CNTNAP2	P	After	-0.01627	0.005575	-2.92	0.0046	-0.02737	-0.00518
C57	P	Before	CNTNAP2	P	Before	-0.01517	0.005583	-2.72	0.0082	-0.02629	-0.00404
C57	P	After	C57	P	Before	-0.00908	0.003624	-2.51	0.0157	-0.01637	-0.0018
C57	ChABC	After	C57	P	Before	-0.01046	0.00538	-1.95	0.0556	-0.02119	0.000256
CNTNAP2	ChABC	After	CNTNAP2	P	After	-0.01048	0.005472	-1.91	0.0591	-0.02137	0.000411
C57	ChABC	After	CNTNAP2	ChABC	After	-0.00901	0.005058	-1.78	0.0789	-0.01909	0.001065
C57	P	Before	CNTNAP2	P	After	-0.00902	0.005771	-1.56	0.122	-0.02051	0.002465
CNTNAP2	P	After	CNTNAP2	P	Before	-0.00614	0.003907	-1.57	0.1221	-0.01399	0.001702
C57	P	After	CNTNAP2	ChABC	After	-0.00763	0.005273	-1.45	0.1521	-0.01813	0.002876
C57	ChABC	Before	C57	P	Before	-0.00725	0.00538	-1.35	0.1819	-0.01797	0.003471

CNTNAP2	ChABC	Before	CNTNAP2	P	After	0.006289	0.005423	1.16	0.2496	-0.0045	0.017083
C57	ChABC	Before	CNTNAP2	ChABC	After	-0.0058	0.005058	-1.15	0.2555	-0.01587	0.00428
C57	ChABC	After	C57	ChABC	Before	-0.00321	0.003355	-0.96	0.3429	-0.00996	0.003533
C57	ChABC	Before	C57	P	After	0.001833	0.00538	0.34	0.7343	-0.00889	0.012554
C57	P	Before	CNTNAP2	ChABC	After	0.001454	0.005273	0.28	0.7835	-0.00905	0.011959
C57	ChABC	After	C57	P	After	-0.00138	0.00538	-0.26	0.7981	-0.0121	0.00934
CNTNAP2	ChABC	Before	CNTNAP2	P	Before	0.000146	0.005223	0.03	0.9778	-0.01026	0.010553

Mouse	treatment	Time					Least Squares Mean
CNTNAP2	ChABC	Before	A				0.072563
CNTNAP2	P	Before	A				0.072417
CNTNAP2	P	After	A	B			0.066273
C57	P	Before		B	C		0.05725
CNTNAP2	ChABC	After		B	C	D	0.055796
C57	ChABC	Before			C	D	0.05
C57	P	After				D	0.048167
C57	ChABC	After			C	D	0.046786

S-FIG-26A

Source	Nparm	DFNum	DFDen	F Ratio	Prob > F
Mouse	1	1	24.4	2.709636	0.1126
treatment	1	1	24.4	3.948714	0.0583
Mouse*trea	1	1	24.4	0.132488	0.719
Time	1	1	22.7	29.2004	<.0001
Mouse*Tim	1	1	22.7	3.102466	0.0916
treatment*	1	1	22.7	0.467497	0.501
Mouse*trea	1	1	22.7	0.601907	0.4458

Mouse	treatment	Time	-Mouse	-treatment	-Time	Difference	Std Error	t Ratio	Prob>|t|	Lower 95%	Upper 95%
CNTNAP2	ChABC	After	CNTNAP2	ChABC	Before	-4.435	0.895786	-4.95	<.0001	-6.2922	-2.5778
C57	ChABC	After	CNTNAP2	P	Before	-7.07171	1.93461	-3.66	0.001	-11.0214	-3.12201
C57	ChABC	After	CNTNAP2	ChABC	Before	-5.72613	1.791101	-3.2	0.0032	-9.3828	-2.06942
CNTNAP2	ChABC	After	CNTNAP2	P	Before	-5.78058	1.93461	-2.99	0.0055	-9.7303	-1.83089
C57	ChABC	Before	CNTNAP2	P	Before	-5.24608	1.93461	-2.71	0.0109	-9.1958	-1.29639
C57	ChABC	After	C57	P	Before	-4.90921	1.93461	-2.54	0.0165	-8.8589	-0.95951
CNTNAP2	P	After	CNTNAP2	P	Before	-2.93392	1.247401	-2.35	0.0273	-5.5092	-0.35868
C57	ChABC	Before	CNTNAP2	ChABC	Before	-3.9005	1.791101	-2.18	0.0374	-7.5572	-0.24379
C57	ChABC	After	CNTNAP2	P	After	-4.13779	2.056409	-2.01	0.0519	-8.3117	0.03613
C57	ChABC	After	C57	ChABC	Before	-1.82562	0.895786	-2.04	0.0537	-3.6828	0.03158
C57	P	After	CNTNAP2	P	Before	-4.08283	2.068185	-1.97	0.0576	-8.3052	0.13957
C57	P	Before	CNTNAP2	ChABC	After	3.61808	1.93461	1.87	0.0712	-0.3316	7.56778
C57	P	After	C57	P	Before	-1.92033	1.034364	-1.86	0.0768	-4.0648	0.22418
C57	ChABC	Before	C57	P	Before	-3.08358	1.93461	-1.59	0.1214	-7.0333	0.86611
C57	ChABC	After	C57	P	After	-2.98888	1.93461	-1.54	0.1328	-6.9386	0.96082
C57	P	After	CNTNAP2	ChABC	Before	-2.73725	1.93461	-1.41	0.1673	-6.6869	1.21245
CNTNAP2	ChABC	After	CNTNAP2	P	After	-2.84666	2.056409	-1.38	0.175	-7.0206	1.32725
C57	ChABC	Before	CNTNAP2	P	After	-2.31216	2.056409	-1.12	0.2685	-6.4861	1.86175
C57	P	Before	CNTNAP2	P	Before	-2.1625	2.068185	-1.05	0.304	-6.3849	2.0599
C57	P	After	CNTNAP2	ChABC	After	1.69775	1.93461	0.88	0.3871	-2.2519	5.64745
CNTNAP2	ChABC	Before	CNTNAP2	P	After	1.58834	2.056409	0.77	0.445	-2.5856	5.76225
C57	ChABC	After	CNTNAP2	ChABC	After	-1.29112	1.791101	-0.72	0.4765	-4.9478	2.36558

CNTNAP2	ChABC	Before	CNTNAP2	P	Before	-1.34558	1.93461	-0.7	0.492	-5.2953	2.60411
C57	ChABC	Before	C57	P	After	-1.16325	1.93461	-0.6	0.5521	-5.1129	2.78645
C57	P	After	CNTNAP2	P	After	-1.14891	2.182542	-0.53	0.602	-5.5814	3.28359
C57	P	Before	CNTNAP2	ChABC	Before	-0.81692	1.93461	-0.42	0.6758	-4.7666	3.13278
C57	P	Before	CNTNAP2	P	After	0.77142	2.182542	0.35	0.7259	-3.6611	5.20392
C57	ChABC	Before	CNTNAP2	ChABC	After	0.5345	1.791101	0.3	0.7674	-3.1222	4.19121

Mouse	treatment	Time					Least Squares Mean
CNTNAP2	P	Before	A				20.16683
CNTNAP2	ChABC	Before	A	B			18.82125
C57	P	Before	A	B	C		18.00433
CNTNAP2	P	After		B	C	D	17.23291
C57	P	After	A	B	C	D	16.084
C57	ChABC	Before			C	D	14.92075
CNTNAP2	ChABC	After			C	D	14.38625
C57	ChABC	After				D	13.09513

S-FIG-26B

Source	Nparm	DFNum	DFDen	F Ratio	Prob > F
Mouse	1	1	21.5	66.4834	<.0001
treatment	1	1	21.5	0.178913	0.6765
Mouse*trea	1	1	21.5	1.283042	0.2698
Time	1	1	19.5	22.51963	0.0001
Mouse*Tim	1	1	19.5	1.101904	0.3067
treatment*	1	1	19.5	3.823954	0.065
Mouse*trea	1	1	19.5	4.670862	0.0433

Mouse	treatment	Time	-Mouse	-treatment	-Time	Difference	Std Error	t Ratio	Prob>|t|	Lower 95%	Upper 95%
C57	P	After	CNTNAP2	P	Before	-10.4476	1.32406	-7.89	<.0001	-13.1334	-7.76174
C57	P	After	CNTNAP2	ChABC	Before	-9.3027	1.255765	-7.41	<.0001	-11.858	-6.74738
C57	ChABC	Before	CNTNAP2	P	Before	-8.1983	1.32406	-6.19	<.0001	-10.8841	-5.51241
C57	ChABC	After	CNTNAP2	P	Before	-8.0064	1.32406	-6.05	<.0001	-10.6923	-5.32058
C57	P	After	CNTNAP2	P	After	-7.9003	1.32406	-5.97	<.0001	-10.5862	-5.21448
C57	ChABC	Before	CNTNAP2	ChABC	Before	-7.0534	1.255765	-5.62	<.0001	-9.6087	-4.49805
C57	ChABC	After	CNTNAP2	ChABC	Before	-6.8615	1.255765	-5.46	<.0001	-9.4169	-4.30622
C57	P	Before	CNTNAP2	P	Before	-6.8873	1.32406	-5.2	<.0001	-9.5731	-4.20141
C57	P	After	CNTNAP2	ChABC	After	-6.5679	1.284376	-5.11	<.0001	-9.1774	-3.95853
C57	P	Before	CNTNAP2	ChABC	Before	-5.7424	1.255765	-4.57	<.0001	-8.2977	-3.18705
C57	ChABC	Before	CNTNAP2	P	After	-5.651	1.32406	-4.27	0.0001	-8.3368	-2.96515
C57	ChABC	After	CNTNAP2	P	After	-5.4592	1.32406	-4.12	0.0002	-8.145	-2.77332
C57	P	After	C57	P	Before	-3.5603	0.898234	-3.96	0.001	-5.451	-1.66966
C57	ChABC	Before	CNTNAP2	ChABC	After	-4.3186	1.284376	-3.36	0.0019	-6.928	-1.70919
C57	P	Before	CNTNAP2	P	After	-4.34	1.32406	-3.28	0.0023	-7.0258	-1.65415
C57	ChABC	After	CNTNAP2	ChABC	After	-4.1268	1.284376	-3.21	0.0029	-6.7362	-1.51736
CNTNAP2	ChABC	After	CNTNAP2	ChABC	Before	-2.7348	0.823283	-3.32	0.0037	-4.4615	-1.00807
CNTNAP2	ChABC	After	CNTNAP2	P	Before	-3.8796	1.265122	-3.07	0.004	-6.442	-1.31732
CNTNAP2	P	After	CNTNAP2	P	Before	-2.5473	1.015629	-2.51	0.0193	-4.6426	-0.45187
C57	P	Before	CNTNAP2	ChABC	After	-3.0076	1.284376	-2.34	0.0251	-5.617	-0.39819
C57	ChABC	After	C57	P	After	2.4412	1.34247	1.82	0.0781	-0.2906	5.17292

C57	ChABC	Before	C57	P	After	2.2493	1.34247	1.68	0.1033	-0.4824	4.98109
CNTNAP2	ChABC	Before	CNTNAP2	P	After	1.4024	1.236065	1.13	0.264	-1.1039	3.9087
CNTNAP2	ChABC	After	CNTNAP2	P	After	-1.3324	1.265122	-1.05	0.299	-3.8947	1.22993
C57	ChABC	Before	C57	P	Before	-1.311	1.34247	-0.98	0.3359	-4.0428	1.42076
CNTNAP2	ChABC	Before	CNTNAP2	P	Before	-1.1449	1.236065	-0.93	0.3605	-3.6512	1.36144
C57	ChABC	After	C57	P	Before	-1.1192	1.34247	-0.83	0.4105	-3.8509	1.61259
C57	ChABC	After	C57	ChABC	Before	0.1918	0.898234	0.21	0.8333	-1.6988	2.0825

Mouse	treatment	Time						Least Squares Mean
CNTNAP2	P	Before	A					23.31375
CNTNAP2	ChABC	Before	A	B				22.16888
CNTNAP2	P	After		B	C			20.7665
CNTNAP2	ChABC	After			C			19.43411
C57	P	Before				D		16.4265
C57	ChABC	After				D	E	15.30733
C57	ChABC	Before				D	E	15.1155
C57	P	After					E	12.86617

S-FIG-26C

Source	Nparm	DFNum	DFDen	F Ratio	Prob > F
Mouse	1	1	24.7	2.477325	0.1282
treatment	1	1	24.7	1.218879	0.2802
Mouse*trea	1	1	24.7	0.345388	0.5621
Time	1	1	23.3	14.41344	0.0009
Mouse*Tim	1	1	23.3	2.971314	0.098
treatment*	1	1	23.3	1.459068	0.2392
Mouse*trea	1	1	23.3	1.528469	0.2287

Mouse	treatment	Time	-Mouse	-treatment	-Time	Difference	Std Error	t Ratio	Prob>|t|	Lower 95%	Upper 95%
CNTNAP2	ChABC	After	CNTNAP2	ChABC	Before	-0.02338	0.005128	-4.56	0.0001	-0.034	-0.01275
C57	ChABC	After	CNTNAP2	ChABC	Before	-0.02763	0.00854	-3.23	0.0027	-0.04499	-0.01026
C57	ChABC	After	CNTNAP2	P	Before	-0.02354	0.009225	-2.55	0.0154	-0.0423	-0.00479
C57	ChABC	Before	CNTNAP2	ChABC	Before	-0.02163	0.00854	-2.53	0.0162	-0.03899	-0.00426
CNTNAP2	ChABC	After	CNTNAP2	P	Before	-0.01929	0.009225	-2.09	0.0442	-0.03805	-0.00054
C57	P	After	CNTNAP2	ChABC	Before	-0.01758	0.009225	-1.91	0.0652	-0.03634	0.001173
C57	ChABC	Before	CNTNAP2	P	Before	-0.01754	0.009225	-1.9	0.0658	-0.0363	0.001215
C57	ChABC	After	C57	P	Before	-0.01621	0.009225	-1.76	0.088	-0.03497	0.002548
C57	ChABC	After	CNTNAP2	P	After	-0.01451	0.01001	-1.45	0.1551	-0.03476	0.005734
C57	P	After	CNTNAP2	P	Before	-0.0135	0.009861	-1.37	0.1801	-0.03355	0.006551
CNTNAP2	ChABC	Before	CNTNAP2	P	After	0.013111	0.01001	1.31	0.1979	-0.00714	0.033359
C57	P	Before	CNTNAP2	ChABC	After	0.011958	0.009225	1.3	0.2037	-0.0068	0.030715
CNTNAP2	P	After	CNTNAP2	P	Before	-0.00903	0.007083	-1.27	0.2142	-0.02362	0.005563
C57	P	Before	CNTNAP2	ChABC	Before	-0.01142	0.009225	-1.24	0.2244	-0.03017	0.00734
C57	ChABC	After	C57	ChABC	Before	-0.006	0.005128	-1.17	0.2543	-0.01662	0.004623
C57	ChABC	Before	C57	P	Before	-0.01021	0.009225	-1.11	0.2763	-0.02897	0.008548
C57	ChABC	After	C57	P	After	-0.01004	0.009225	-1.09	0.2841	-0.0288	0.008715
C57	P	After	C57	P	Before	-0.00617	0.005921	-1.04	0.3088	-0.01843	0.0061
CNTNAP2	ChABC	After	CNTNAP2	P	After	-0.01026	0.01001	-1.03	0.3115	-0.03051	0.009984
C57	ChABC	Before	CNTNAP2	P	After	-0.00851	0.01001	-0.85	0.4002	-0.02876	0.011734
C57	P	Before	CNTNAP2	P	Before	-0.00733	0.009861	-0.74	0.4623	-0.02739	0.012718
C57	P	After	CNTNAP2	ChABC	After	0.005792	0.009225	0.63	0.5344	-0.01297	0.024548
C57	ChABC	After	CNTNAP2	ChABC	After	-0.00425	0.00854	-0.5	0.622	-0.02162	0.013115

CNTNAP2	ChABC	Before	CNTNAP2	P	Before	0.004083	0.009225	0.44	0.6609	-0.01467	0.02284
C57	ChABC	Before	C57	P	After	-0.00404	0.009225	-0.44	0.6641	-0.0228	0.014715
C57	P	After	CNTNAP2	P	After	-0.00447	0.0106	-0.42	0.6754	-0.02592	0.016979
C57	ChABC	Before	CNTNAP2	ChABC	After	0.00175	0.00854	0.2	0.8389	-0.01562	0.019115
C57	P	Before	CNTNAP2	P	After	0.001694	0.0106	0.16	0.8738	-0.01976	0.023146

Mouse	treatment	Time				Least Squares Mean
CNTNAP2	ChABC	Before	A			0.07125
CNTNAP2	P	Before	A	B		0.067167
C57	P	Before	A	B	C	0.059833
CNTNAP2	P	After	A	B	C	0.058139
C57	P	After	A	B	C	0.053667
C57	ChABC	Before		B	C	0.049625
CNTNAP2	ChABC	After			C	0.047875
C57	ChABC	After			C	0.043625

S-FIG-26D

Source	Nparm	DFNum	DFDen	F Ratio	Prob > F
Mouse	1	1	21.5	67.09599	<.0001
treatment	1	1	21.5	0.140431	0.7115
Mouse*trea	1	1	21.5	1.357586	0.2567
Time	1	1	19.6	22.68342	0.0001
Mouse*Tim	1	1	19.6	1.016176	0.3257
treatment*	1	1	19.6	3.679747	0.0698
Mouse*trea	1	1	19.6	4.678054	0.0431

Mouse	treatment	Time	-Mouse	-treatment	-Time	Difference	Std Error	t Ratio	Prob>|t|	Lower 95%	Upper 95%
C57	P	After	CNTNAP2	P	Before	-0.03489	0.004401	-7.93	<.0001	-0.04382	-0.02597
C57	P	After	CNTNAP2	ChABC	Before	-0.03121	0.00417	-7.48	<.0001	-0.03969	-0.02273
C57	ChABC	Before	CNTNAP2	P	Before	-0.02706	0.004401	-6.15	<.0001	-0.03598	-0.01813
C57	ChABC	After	CNTNAP2	P	Before	-0.02656	0.004401	-6.03	<.0001	-0.03548	-0.01763
C57	P	After	CNTNAP2	P	After	-0.02643	0.004401	-6.01	<.0001	-0.03535	-0.01751
C57	ChABC	Before	CNTNAP2	ChABC	Before	-0.02338	0.00417	-5.6	<.0001	-0.03186	-0.01489
C57	ChABC	After	CNTNAP2	ChABC	Before	-0.02288	0.00417	-5.49	<.0001	-0.03136	-0.01439
C57	P	Before	CNTNAP2	P	Before	-0.02289	0.004401	-5.2	<.0001	-0.03182	-0.01397
C57	P	After	CNTNAP2	ChABC	After	-0.022	0.004267	-5.15	<.0001	-0.03066	-0.01333
C57	P	Before	CNTNAP2	ChABC	Before	-0.01921	0.00417	-4.61	<.0001	-0.02769	-0.01073
C57	ChABC	Before	CNTNAP2	P	After	-0.0186	0.004401	-4.23	0.0002	-0.02752	-0.00967
C57	ChABC	After	CNTNAP2	P	After	-0.0181	0.004401	-4.11	0.0002	-0.02702	-0.00917
C57	P	After	C57	P	Before	-0.012	0.003021	-3.97	0.0009	-0.01836	-0.00564
C57	ChABC	Before	CNTNAP2	ChABC	After	-0.01416	0.004267	-3.32	0.0021	-0.02283	-0.0055
C57	P	Before	CNTNAP2	P	After	-0.01443	0.004401	-3.28	0.0023	-0.02335	-0.00551
C57	ChABC	After	CNTNAP2	ChABC	After	-0.01366	0.004267	-3.2	0.0029	-0.02233	-0.005
CNTNAP2	ChABC	After	CNTNAP2	ChABC	Before	-0.00921	0.002768	-3.33	0.0036	-0.01502	-0.00341
CNTNAP2	ChABC	After	CNTNAP2	P	Before	-0.0129	0.004207	-3.07	0.004	-0.02142	-0.00438
CNTNAP2	P	After	CNTNAP2	P	Before	-0.00846	0.003408	-2.48	0.0203	-0.01549	-0.00144
C57	P	Before	CNTNAP2	ChABC	After	-0.01	0.004267	-2.34	0.025	-0.01866	-0.00133
C57	ChABC	After	C57	P	After	0.008333	0.004458	1.87	0.0704	-0.00074	0.017402
C57	ChABC	Before	C57	P	After	0.007833	0.004458	1.76	0.0881	-0.00124	0.016902

CNTNAP2	ChABC	Before	CNTNAP2	P	After	0.004779	0.004109	1.16	0.2524	-0.00355	0.013108
CNTNAP2	ChABC	After	CNTNAP2	P	After	-0.00443	0.004207	-1.05	0.2987	-0.01295	0.004086
C57	ChABC	Before	C57	P	Before	-0.00417	0.004458	-0.93	0.3568	-0.01324	0.004902
CNTNAP2	ChABC	Before	CNTNAP2	P	Before	-0.00368	0.004109	-0.9	0.3758	-0.01201	0.004645
C57	ChABC	After	C57	P	Before	-0.00367	0.004458	-0.82	0.4167	-0.01274	0.005402
C57	ChABC	After	C57	ChABC	Before	0.0005	0.003021	0.17	0.8704	-0.00586	0.006857

Mouse	treatment	Time						Least Squares Mean
CNTNAP2	P	Before	A					0.077559
CNTNAP2	ChABC	Before	A	B				0.073875
CNTNAP2	P	After		B	C			0.069096
CNTNAP2	ChABC	After			C			0.064663
C57	P	Before				D		0.054667
C57	ChABC	After				D	E	0.051
C57	ChABC	Before				D	E	0.0505
C57	P	After					E	0.042667

S-FIG-27A

Source	Nparm	DFNum	DFDen	F Ratio	Prob > F
Mouse	1	1	50.3	1.037396	0.3133
treatment	1	1	50.3	0.002204	0.9627
Mouse*trea	1	1	50.3	1.441414	0.2355
Time	1	1	49.7	9.748031	0.003
Mouse*Tim	1	1	49.7	0.321575	0.5732
treatment*	1	1	49.7	1.16261	0.2861
Mouse*trea	1	1	49.7	0.008038	0.9289

Mouse	treatment	Time	-Mouse	-treatment	-Time	Difference	Std Error	t Ratio	Prob>|t|	Lower 95%	Upper 95%
C57	ChABC	After	CNTNAP2	ChABC	Before	5.87732	2.560014	2.3	0.0239	0.7942	10.9604
CNTNAP2	ChABC	Before	CNTNAP2	P	After	-6.46939	2.820224	-2.29	0.024	-12.0683	-0.8705
CNTNAP2	P	After	CNTNAP2	P	Before	5.82897	2.724236	2.14	0.0371	0.3635	11.2945
C57	P	After	CNTNAP2	ChABC	Before	5.60125	2.67137	2.1	0.0387	0.297	10.9055
C57	ChABC	After	CNTNAP2	P	Before	5.2369	2.751932	1.9	0.0601	-0.2273	10.7011
C57	P	Before	CNTNAP2	P	After	-5.50731	2.995517	-1.84	0.0691	-11.4543	0.4397
C57	P	After	C57	P	Before	4.63917	2.569829	1.81	0.0773	-0.5276	9.8059
C57	ChABC	After	C57	P	Before	4.91524	2.751932	1.79	0.0773	-0.5489	10.3794
C57	P	After	CNTNAP2	P	Before	4.96083	2.855815	1.74	0.0857	-0.7096	10.6313
C57	ChABC	Before	CNTNAP2	ChABC	Before	4.14875	2.560014	1.62	0.1085	-0.9344	9.2319
CNTNAP2	ChABC	After	CNTNAP2	ChABC	Before	3.36525	2.270986	1.48	0.1447	-1.197	7.9276
C57	ChABC	Before	CNTNAP2	P	Before	3.50833	2.751932	1.27	0.2055	-1.9558	8.9725
C57	ChABC	Before	C57	P	Before	3.18667	2.751932	1.16	0.2498	-2.2775	8.6508
CNTNAP2	ChABC	After	CNTNAP2	P	After	-3.10414	2.856225	-1.09	0.2799	-8.7743	2.5661
CNTNAP2	ChABC	After	CNTNAP2	P	Before	2.72484	2.70935	1.01	0.3171	-2.6545	8.1042
C57	ChABC	After	CNTNAP2	ChABC	After	2.51207	2.599621	0.97	0.3364	-2.6494	7.6736
C57	P	Before	CNTNAP2	ChABC	After	-2.40317	2.70935	-0.89	0.3773	-7.7826	2.9762
C57	P	After	CNTNAP2	ChABC	After	2.236	2.70935	0.83	0.4113	-3.1434	7.6154
C57	ChABC	Before	CNTNAP2	P	After	-2.32064	2.896649	-0.8	0.425	-8.0713	3.43
C57	ChABC	After	C57	ChABC	Before	1.72857	2.379199	0.73	0.471	-3.0549	6.512
C57	ChABC	Before	C57	P	After	-1.4525	2.751932	-0.53	0.5989	-6.9167	4.0117
C57	P	Before	CNTNAP2	ChABC	Before	0.96208	2.67137	0.36	0.7195	-4.3421	6.2663

C57	ChABC	Before	CNTNAP2	ChABC	After	0.7835	2.599621	0.3	0.7638	-4.378	5.945
C57	P	After	CNTNAP2	P	After	-0.86814	2.995517	-0.29	0.7726	-6.8151	5.0789
CNTNAP2	ChABC	Before	CNTNAP2	P	Before	-0.64042	2.67137	-0.24	0.8111	-5.9446	4.6638
C57	ChABC	After	CNTNAP2	P	After	-0.59207	2.896649	-0.2	0.8385	-6.3427	5.1586
C57	P	Before	CNTNAP2	P	Before	0.32167	2.855815	0.11	0.9106	-5.3488	5.9921
C57	ChABC	After	C57	P	After	0.27607	2.751932	0.1	0.9203	-5.1881	5.7403

Mouse	treatment	Time				Least Squares Mean
CNTNAP2	P	After	A			26.74064
C57	ChABC	After	A	B		26.14857
C57	P	After	A	B		25.8725
C57	ChABC	Before	A	B	C	24.42
CNTNAP2	ChABC	After	A	B	C	23.6365
C57	P	Before	A	B	C	21.23333
CNTNAP2	P	Before		B	C	20.91167
CNTNAP2	ChABC	Before			C	20.27125

S-FIG-27B

Source	Nparm	DFNum	DFDen	F Ratio	Prob > F
Mouse	1	1	24.5	7.348329	0.0121
treatment	1	1	24.5	4.245309	0.0501
Mouse*tre	1	1	24.5	0.195376	0.6624
Time	1	1	25	6.812852	0.0151
Mouse*Tim	1	1	25	0.82627	0.372
treatment*	1	1	25	0.500685	0.4857
Mouse*tre	1	1	25	1.23678	0.2767

Mouse	treatment	Time	-Mouse	-treatment	-Time	Difference	Std Error	t Ratio	Prob>|t|	Lower 95%	Upper 95%
C57	ChABC	After	CNTNAP2	P	Before	13.7592	3.19231	4.31	<.0001	7.3245	20.19378
C57	ChABC	Before	CNTNAP2	P	Before	9.9104	3.19231	3.1	0.0033	3.4758	16.34503
CNTNAP2	ChABC	After	CNTNAP2	P	Before	8.7917	3.19231	2.75	0.0085	2.357	15.22628
C57	P	After	CNTNAP2	P	Before	9.3717	3.412723	2.75	0.0087	2.4928	16.25056
C57	ChABC	After	CNTNAP2	ChABC	Before	8.0738	2.955505	2.73	0.009	2.1165	14.03105
CNTNAP2	P	After	CNTNAP2	P	Before	9.7584	4.170264	2.34	0.0265	1.2232	18.29354
C57	ChABC	After	C57	P	Before	6.7575	3.19231	2.12	0.04	0.3229	13.19212
C57	P	Before	CNTNAP2	P	Before	7.0017	3.412723	2.05	0.0462	0.1228	13.88056
CNTNAP2	ChABC	Before	CNTNAP2	P	Before	5.6854	3.19231	1.78	0.0819	-0.7492	12.12003
C57	ChABC	After	CNTNAP2	ChABC	After	4.9675	2.955505	1.68	0.0999	-0.9898	10.9248
C57	ChABC	Before	CNTNAP2	ChABC	Before	4.225	2.955505	1.43	0.1599	-1.7323	10.1823
C57	ChABC	After	C57	P	After	4.3875	3.19231	1.37	0.1763	-2.0471	10.82212
C57	ChABC	After	C57	ChABC	Before	3.8488	3.289208	1.17	0.2537	-2.9473	10.64484
C57	P	After	CNTNAP2	ChABC	Before	3.6863	3.19231	1.15	0.2545	-2.7484	10.12087
CNTNAP2	ChABC	Before	CNTNAP2	P	After	-4.0729	3.627224	-1.12	0.2674	-11.3773	3.23138
C57	ChABC	After	CNTNAP2	P	After	4.0008	3.627224	1.1	0.2759	-3.3035	11.30513
CNTNAP2	ChABC	After	CNTNAP2	ChABC	Before	3.1063	3.289208	0.94	0.3546	-3.6898	9.90234
C57	ChABC	Before	C57	P	Before	2.9087	3.19231	0.91	0.3672	-3.5259	9.34337
C57	P	Before	CNTNAP2	P	After	-2.7567	3.822642	-0.72	0.4745	-10.4551	4.9417
C57	P	After	C57	P	Before	2.37	3.798051	0.62	0.5386	-5.4774	10.21745
C57	P	Before	CNTNAP2	ChABC	After	-1.79	3.19231	-0.56	0.5778	-8.2246	4.64462
C57	P	Before	CNTNAP2	ChABC	Before	1.3163	3.19231	0.41	0.6821	-5.1184	7.75087

C57	ChABC	Before	CNTNAP2	ChABC	After	1.1187	2.955505	0.38	0.7069	-4.8385	7.07605
CNTNAP2	ChABC	After	CNTNAP2	P	After	-0.9667	3.627224	-0.27	0.7911	-8.271	6.33763
C57	P	After	CNTNAP2	ChABC	After	0.58	3.19231	0.18	0.8567	-5.8546	7.01462
C57	ChABC	Before	C57	P	After	0.5388	3.19231	0.17	0.8668	-5.8959	6.97337
C57	P	After	CNTNAP2	P	After	-0.3867	3.822642	-0.1	0.9199	-8.0851	7.3117
C57	ChABC	Before	CNTNAP2	P	After	0.1521	3.627224	0.04	0.9667	-7.1523	7.45638

Mouse	treatment	Time				Least Squares Mean
C57	ChABC	After	A			29.5925
C57	ChABC	Before	A	B		25.74375
CNTNAP2	P	After	A	B		25.5917
C57	P	After	A	B		25.205
CNTNAP2	ChABC	After	A	B		24.625
C57	P	Before		B		22.835
CNTNAP2	ChABC	Before		B	C	21.51875
CNTNAP2	P	Before			C	15.83333

S-FIG-27C

Source	Nparm	DFNum	DFDen	F Ratio	Prob > F
Mouse	1	1	24.1	0.141304	0.7103
treatment	1	1	24.1	1.718544	0.2023
Mouse*trea	1	1	24.1	0.832068	0.3707
Time	1	1	22.9	2.458764	0.1306
Mouse*Tim	1	1	22.9	0.009711	0.9224
treatment*	1	1	22.9	0.758701	0.3928
Mouse*trea	1	1	22.9	2.032517	0.1675

Mouse	treatment	Time	-Mouse	-treatment	-Time	Difference	Std Error	t Ratio	Prob>|t|	Lower 95%	Upper 95%
C57	ChABC	After	C57	ChABC	Before	-1.09833	3.483973	-0.32	0.7557	-8.353	6.15637
C57	ChABC	After	C57	P	After	-4.98333	4.379419	-1.14	0.2621	-13.8427	3.87604
C57	ChABC	After	C57	P	Before	1.925	4.379419	0.44	0.6627	-6.9344	10.78437
C57	ChABC	After	CNTNAP2	ChABC	After	-0.99531	4.212234	-0.24	0.8144	-9.5108	7.5202
C57	ChABC	After	CNTNAP2	ChABC	Before	2.53292	4.096571	0.62	0.54	-5.7543	10.8201
C57	ChABC	After	CNTNAP2	P	After	-5.49217	4.364753	-1.26	0.2155	-14.3109	3.32653
C57	ChABC	After	CNTNAP2	P	Before	-3.89747	4.364753	-0.89	0.3772	-12.7162	4.92123
C57	ChABC	Before	C57	P	After	-3.885	4.379419	-0.89	0.3805	-12.7444	4.97437
C57	ChABC	Before	C57	P	Before	3.02333	4.379419	0.69	0.4941	-5.836	11.8827
C57	ChABC	Before	CNTNAP2	ChABC	After	0.10302	4.212234	0.02	0.9806	-8.4125	8.61853
C57	ChABC	Before	CNTNAP2	ChABC	Before	3.63125	4.096571	0.89	0.3809	-4.6559	11.91843
C57	ChABC	Before	CNTNAP2	P	After	-4.39383	4.364753	-1.01	0.3201	-13.2125	4.42487
C57	ChABC	Before	CNTNAP2	P	Before	-2.79914	4.364753	-0.64	0.5249	-11.6178	6.01956
C57	P	After	C57	P	Before	6.90833	3.483973	1.98	0.0609	-0.3464	14.16303
C57	P	After	CNTNAP2	ChABC	After	3.98802	4.212234	0.95	0.3495	-4.5275	12.50353
C57	P	After	CNTNAP2	ChABC	Before	7.51625	4.096571	1.83	0.0742	-0.7709	15.80343
C57	P	After	CNTNAP2	P	After	-0.50883	4.364753	-0.12	0.9078	-9.3275	8.30987
C57	P	After	CNTNAP2	P	Before	1.08586	4.364753	0.25	0.8048	-7.7328	9.90456
C57	P	Before	CNTNAP2	ChABC	After	-2.92031	4.212234	-0.69	0.4922	-11.4358	5.5952
C57	P	Before	CNTNAP2	ChABC	Before	0.60792	4.096571	0.15	0.8828	-7.6793	8.8951
C57	P	Before	CNTNAP2	P	After	-7.41717	4.364753	-1.7	0.0969	-16.2359	1.40153
C57	P	Before	CNTNAP2	P	Before	-5.82247	4.364753	-1.33	0.1897	-14.6412	2.99623

CNTNAP2	ChABC	After	CNTNAP2	ChABC	Before	3.52823	3.17247	1.11	0.2782	-3.0553	10.11177
CNTNAP2	ChABC	After	CNTNAP2	P	After	-4.49686	4.196984	-1.07	0.2902	-12.9716	3.97787
CNTNAP2	ChABC	After	CNTNAP2	P	Before	-2.90216	4.196984	-0.69	0.4931	-11.3769	5.57257
CNTNAP2	ChABC	Before	CNTNAP2	P	After	-8.02508	4.080889	-1.97	0.0561	-16.269	0.21881
CNTNAP2	ChABC	Before	CNTNAP2	P	Before	-6.43039	4.080889	-1.58	0.1228	-14.6743	1.81351
CNTNAP2	P	After	CNTNAP2	P	Before	1.5947	3.777917	0.42	0.6761	-6.1384	9.3278

Mouse	treatment	Time		Least Squares Mean
CNTNAP2	P	After	A	27.04883
C57	P	After	A	26.54
CNTNAP2	P	Before	A	25.45414
C57	ChABC	Before	A	22.655
CNTNAP2	ChABC	After	A	22.55198
C57	ChABC	After	A	21.55667
C57	P	Before	A	19.63167
CNTNAP2	ChABC	Before	A	19.02375

S-FIG-28A

Source	Nparm	DFNum	DFDen	F Ratio	Prob > F
Mouse	1	1	49.9	0.034004	0.8544
treatment	1	1	49.9	1.164806	0.2857
Mouse*trea	1	1	49.9	0.455331	0.5029
Time	1	1	48.8	5.36874	0.0247
Mouse*Tim	1	1	48.8	1.307491	0.2584
treatment*	1	1	48.8	4.666024	0.0357
Mouse*trea	1	1	48.8	0.038066	0.8461

Mouse	treatment	Time	-Mouse	-treatment	-Time	Difference	Std Error	t Ratio	Prob>|t|	Lower 95%	Upper 95%
CNTNAP2	P	After	CNTNAP2	P	Before	8.00483	3.022375	2.65	0.0107	1.9378	14.0719
CNTNAP2	ChABC	Before	CNTNAP2	P	After	-8.18587	3.441766	-2.38	0.0195	-15.0234	-1.3484
C57	P	Before	CNTNAP2	P	After	-7.839	3.657611	-2.14	0.0348	-15.1057	-0.5723
C57	ChABC	After	CNTNAP2	P	After	-7.01292	3.535885	-1.98	0.0504	-14.0376	0.0117
CNTNAP2	ChABC	After	CNTNAP2	P	After	-6.66451	3.482177	-1.91	0.0588	-13.5816	0.2526
C57	ChABC	Before	CNTNAP2	P	After	-5.92364	3.535885	-1.68	0.0973	-12.9483	1.101
C57	P	After	C57	P	Before	4.32	2.831175	1.53	0.1337	-1.3745	10.0145
C57	P	After	CNTNAP2	ChABC	Before	4.66688	3.275142	1.42	0.1578	-1.8429	11.1766
C57	P	After	CNTNAP2	P	Before	4.48583	3.501274	1.28	0.2035	-2.4734	11.445
C57	ChABC	After	C57	P	After	-3.49393	3.373912	-1.04	0.3033	-10.2	3.2121
C57	P	After	CNTNAP2	P	After	-3.519	3.657611	-0.96	0.3386	-10.7857	3.7477
C57	P	After	CNTNAP2	ChABC	After	3.14552	3.317583	0.95	0.3457	-3.4476	9.7387
C57	ChABC	Before	CNTNAP2	ChABC	Before	2.26223	3.138617	0.72	0.473	-3.9762	8.5006
C57	ChABC	Before	C57	P	After	-2.40464	3.373912	-0.71	0.4779	-9.1107	4.3014
C57	ChABC	Before	CNTNAP2	P	Before	2.08119	3.373912	0.62	0.5389	-4.6249	8.7873
CNTNAP2	ChABC	After	CNTNAP2	ChABC	Before	1.52136	2.508279	0.61	0.547	-3.5199	6.5627
C57	ChABC	Before	C57	P	Before	1.91536	3.373912	0.57	0.5717	-4.7907	8.6214
C57	ChABC	After	C57	ChABC	Before	-1.08929	2.621159	-0.42	0.6796	-6.3613	4.1828
CNTNAP2	ChABC	After	CNTNAP2	P	Before	1.34032	3.317583	0.4	0.6872	-5.2528	7.9335
C57	ChABC	After	CNTNAP2	ChABC	Before	1.17295	3.138617	0.37	0.7095	-5.0654	7.4113
C57	P	Before	CNTNAP2	ChABC	After	-1.17448	3.317583	-0.35	0.7242	-7.7676	5.4187
C57	ChABC	After	CNTNAP2	P	Before	0.9919	3.373912	0.29	0.7695	-5.7142	7.698

C57	ChABC	After	C57	P	Before	0.82607	3.373912	0.24	0.8072	-5.88	7.5321
C57	ChABC	Before	CNTNAP2	ChABC	After	0.74087	3.182879	0.23	0.8165	-5.5845	7.0662
C57	ChABC	After	CNTNAP2	ChABC	After	-0.34841	3.182879	-0.11	0.9131	-6.6738	5.977
C57	P	Before	CNTNAP2	ChABC	Before	0.34688	3.275142	0.11	0.9159	-6.1629	6.8566
CNTNAP2	ChABC	Before	CNTNAP2	P	Before	-0.18104	3.275142	-0.06	0.956	-6.6908	6.3287
C57	P	Before	CNTNAP2	P	Before	0.16583	3.501274	0.05	0.9623	-6.7934	7.125

Mouse	treatment	Time			Least Squares Mean
CNTNAP2	P	After	A		41.5565
C57	P	After	A	B	38.0375
C57	ChABC	Before	A	B	35.63286
CNTNAP2	ChABC	After	A	B	34.89198
C57	ChABC	After	A	B	34.54357
C57	P	Before		B	33.7175
CNTNAP2	P	Before		B	33.55167
CNTNAP2	ChABC	Before		B	33.37063

S-FIG-28B

Source	Nparm	DFNum	DFDen	F Ratio	Prob > F
Mouse	1	1	24.7	1.187724	0.2863
treatment	1	1	24.7	0.842019	0.3677
Mouse*trea	1	1	24.7	0.027653	0.8693
Time	1	1	24.9	1.816837	0.1898
Mouse*Tim	1	1	24.9	1.100901	0.3042
treatment*	1	1	24.9	0.47564	0.4968
Mouse*trea	1	1	24.9	0.085575	0.7723

Mouse	treatment	Time	-Mouse	-treatment	-Time	Difference	Std Error	t Ratio	Prob>|t|	Lower 95%	Upper 95%
C57	ChABC	After	C57	ChABC	Before	-0.23375	4.248661	-0.06	0.9566	-9.0168	8.54935
C57	ChABC	After	C57	P	After	1.41625	4.388964	0.32	0.7484	-7.4201	10.25264
C57	ChABC	After	C57	P	Before	3.06792	4.388964	0.7	0.4881	-5.7685	11.90431
C57	ChABC	After	CNTNAP2	ChABC	After	0.9375	4.063391	0.23	0.8186	-7.2434	9.11841
C57	ChABC	After	CNTNAP2	ChABC	Before	4.29625	4.063391	1.06	0.2959	-3.8847	12.47716
C57	ChABC	After	CNTNAP2	P	After	0.24113	5.018108	0.05	0.9619	-9.8604	10.34267
C57	ChABC	After	CNTNAP2	P	Before	8.26292	4.388964	1.88	0.0661	-0.5735	17.09931
C57	ChABC	Before	C57	P	After	1.65	4.388964	0.38	0.7087	-7.1864	10.48639
C57	ChABC	Before	C57	P	Before	3.30167	4.388964	0.75	0.4558	-5.5347	12.13806
C57	ChABC	Before	CNTNAP2	ChABC	After	1.17125	4.063391	0.29	0.7745	-7.0097	9.35216
C57	ChABC	Before	CNTNAP2	ChABC	Before	4.53	4.063391	1.11	0.2708	-3.6509	12.71091
C57	ChABC	Before	CNTNAP2	P	After	0.47488	5.018108	0.09	0.925	-9.6267	10.57642
C57	ChABC	Before	CNTNAP2	P	Before	8.49667	4.388964	1.94	0.0591	-0.3397	17.33306
C57	P	After	C57	P	Before	1.65167	4.905932	0.34	0.7394	-8.4902	11.79351
C57	P	After	CNTNAP2	ChABC	After	-0.47875	4.388964	-0.11	0.9136	-9.3151	8.35764
C57	P	After	CNTNAP2	ChABC	Before	2.88	4.388964	0.66	0.515	-5.9564	11.71639
C57	P	After	CNTNAP2	P	After	-1.17512	5.285193	-0.22	0.825	-11.8144	9.46419
C57	P	After	CNTNAP2	P	Before	6.84667	4.692	1.46	0.1514	-2.5998	16.29317
C57	P	Before	CNTNAP2	ChABC	After	-2.13042	4.388964	-0.49	0.6297	-10.9668	6.70598
C57	P	Before	CNTNAP2	ChABC	Before	1.22833	4.388964	0.28	0.7808	-7.6081	10.06473
C57	P	Before	CNTNAP2	P	After	-2.82679	5.285193	-0.53	0.5953	-13.4661	7.81252
C57	P	Before	CNTNAP2	P	Before	5.195	4.692	1.11	0.274	-4.2515	14.6415

CNTNAP2	ChABC	After	CNTNAP2	ChABC	Before	3.35875	4.248661	0.79	0.4372	-5.4243	12.14185
CNTNAP2	ChABC	After	CNTNAP2	P	After	-0.69637	5.018108	-0.14	0.8902	-10.7979	9.40517
CNTNAP2	ChABC	After	CNTNAP2	P	Before	7.32542	4.388964	1.67	0.102	-1.511	16.16181
CNTNAP2	ChABC	Before	CNTNAP2	P	After	-4.05512	5.018108	-0.81	0.4232	-14.1567	6.04642
CNTNAP2	ChABC	Before	CNTNAP2	P	Before	3.96667	4.388964	0.9	0.3709	-4.8697	12.80306
CNTNAP2	P	After	CNTNAP2	P	Before	8.02179	5.475999	1.46	0.1539	-3.1882	19.23182

Mouse	treatment	Time		Least Squares Mean
C57	ChABC	Before	A	36.775
C57	ChABC	After	A	36.54125
CNTNAP2	P	After	A	36.30013
CNTNAP2	ChABC	After	A	35.60375
C57	P	After	A	35.125
C57	P	Before	A	33.47333
CNTNAP2	ChABC	Before	A	32.245
CNTNAP2	P	Before	A	28.27833

S-FIG-28C

Source	Nparm	DFNum	DFDen	F Ratio	Prob > F
Mouse	1	1	23.7	0.589409	0.4502
treatment	1	1	23.7	3.158715	0.0884
Mouse*trea	1	1	23.7	0.11591	0.7365
Time	1	1	21.2	3.529856	0.0741
Mouse*Tim	1	1	21.2	0.096013	0.7597
treatment*	1	1	21.2	7.448997	0.0125
Mouse*trea	1	1	21.2	0.096231	0.7594

Mouse	treatment	Time	-Mouse	-treatment	-Time	Difference	Std Error	t Ratio	Prob>|t|	Lower 95%	Upper 95%
C57	ChABC	After	CNTNAP2	P	After	-12.51	4.969014	-2.52	0.0166	-22.6038	-2.4162
C57	P	After	C57	P	Before	6.9883	2.971083	2.35	0.0293	0.7797	13.197
CNTNAP2	ChABC	After	CNTNAP2	P	After	-10.2432	4.720525	-2.17	0.0367	-19.8168	-0.6695
CNTNAP2	ChABC	Before	CNTNAP2	P	After	-9.8937	4.632017	-2.14	0.0398	-19.2989	-0.4885
C57	P	Before	CNTNAP2	P	After	-10.4283	4.969014	-2.1	0.0433	-20.5221	-0.3345
C57	ChABC	Before	CNTNAP2	P	After	-10.28	4.969014	-2.07	0.0461	-20.3738	-0.1862
CNTNAP2	P	After	CNTNAP2	P	Before	6.9873	3.419026	2.04	0.0517	-0.0558	14.0303
C57	ChABC	After	C57	P	After	-9.07	5.087647	-1.78	0.0842	-19.4374	1.2974
C57	P	After	CNTNAP2	ChABC	After	6.8032	4.845246	1.4	0.1697	-3.0553	16.6617
C57	P	After	CNTNAP2	ChABC	Before	6.4538	4.759058	1.36	0.1847	-3.2441	16.1516
C57	ChABC	Before	C57	P	After	-6.84	5.087647	-1.34	0.1884	-17.2074	3.5274
C57	ChABC	After	CNTNAP2	P	Before	-5.5227	4.969014	-1.11	0.2741	-15.6165	4.5711
C57	ChABC	After	C57	ChABC	Before	-2.23	2.971083	-0.75	0.4619	-8.4387	3.9787
C57	P	After	CNTNAP2	P	Before	3.5473	4.969014	0.71	0.4801	-6.5465	13.6411
C57	P	Before	CNTNAP2	P	Before	-3.4411	4.969014	-0.69	0.4933	-13.5348	6.6527
C57	P	After	CNTNAP2	P	After	-3.44	4.969014	-0.69	0.4934	-13.5338	6.6538
CNTNAP2	ChABC	After	CNTNAP2	P	Before	-3.2559	4.720525	-0.69	0.4948	-12.8295	6.3177
C57	ChABC	Before	CNTNAP2	P	Before	-3.2927	4.969014	-0.66	0.512	-13.3865	6.8011
CNTNAP2	ChABC	Before	CNTNAP2	P	Before	-2.9065	4.632017	-0.63	0.5344	-12.3117	6.4987

C57	ChABC	After	CNTNAP2	ChABC	Before	-2.6162	4.759058	-0.55	0.5864	-12.3141	7.0816
C57	ChABC	After	CNTNAP2	ChABC	After	-2.2668	4.845246	-0.47	0.643	-12.1253	7.5917
C57	ChABC	After	C57	P	Before	-2.0817	5.087647	-0.41	0.6852	-12.4491	8.2858
CNTNAP2	ChABC	After	CNTNAP2	ChABC	Before	-0.3494	2.729153	-0.13	0.8994	-6.0389	5.3401
C57	P	Before	CNTNAP2	ChABC	Before	-0.5346	4.759058	-0.11	0.9113	-10.2324	9.1633
C57	ChABC	Before	CNTNAP2	ChABC	Before	-0.3862	4.759058	-0.08	0.9358	-10.0841	9.3116
C57	P	Before	CNTNAP2	ChABC	After	-0.1852	4.845246	-0.04	0.9697	-10.0437	9.6734
C57	ChABC	Before	C57	P	Before	0.1483	5.087647	0.03	0.9769	-10.2191	10.5158
C57	ChABC	Before	CNTNAP2	ChABC	After	-0.0368	4.845246	-0.01	0.994	-9.8953	9.8217

Mouse	treatment	Time				Least Squares Mean
CNTNAP2	P	After	A			44.38999
C57	P	After	A	B		40.95
CNTNAP2	P	Before	A	B	C	37.40273
CNTNAP2	ChABC	Before		B	C	34.49625
CNTNAP2	ChABC	After		B	C	34.14683
C57	ChABC	Before		B	C	34.11
C57	P	Before			C	33.96167
C57	ChABC	After		B	C	31.88

S-FIG-29A

Source	Nparm	DFNum	DFDen	F Ratio	Prob > F
Mouse	1	1	49.8	0.771958	0.3838
treatment	1	1	49.8	1.958953	0.1678
Mouse*trea	1	1	49.8	1.133107	0.2922
Time	1	1	48.7	0.401451	0.5293
Mouse*Tim	1	1	48.7	1.387407	0.2446
treatment*	1	1	48.7	6.259257	0.0158
Mouse*trea	1	1	48.7	0.01693	0.897

Mouse	treatment	Time	-Mouse	-treatment	-Time	Difference	Std Error	t Ratio	Prob>|t|	Lower 95%	Upper 95%
C57	ChABC	After	C57	P	After	-3.7381	1.562229	-2.39	0.0189	-6.84322	-0.63297
C57	ChABC	Before	C57	P	After	-3.38095	1.562229	-2.16	0.0332	-6.48608	-0.27583
C57	ChABC	After	CNTNAP2	ChABC	Before	-2.94643	1.45328	-2.03	0.0457	-5.83501	-0.05785
C57	P	After	C57	P	Before	2.66667	1.311223	2.03	0.0476	0.02924	5.3041
C57	P	After	CNTNAP2	ChABC	After	2.81642	1.536154	1.83	0.0701	-0.23644	5.86928
C57	ChABC	Before	CNTNAP2	ChABC	Before	-2.58929	1.45328	-1.78	0.0783	-5.47786	0.29929
C57	ChABC	After	CNTNAP2	P	After	-2.90226	1.637254	-1.77	0.0797	-6.15496	0.35044
CNTNAP2	ChABC	After	CNTNAP2	ChABC	Before	-2.02475	1.161676	-1.74	0.0877	-4.35966	0.31015
C57	ChABC	Before	CNTNAP2	P	After	-2.54512	1.637254	-1.55	0.1236	-5.79782	0.70758
C57	P	After	CNTNAP2	P	Before	2.16667	1.621202	1.34	0.1849	-1.05568	5.38901
C57	P	Before	CNTNAP2	ChABC	Before	-1.875	1.516495	-1.24	0.2196	-4.88923	1.13923
CNTNAP2	ChABC	After	CNTNAP2	P	After	-1.98058	1.612392	-1.23	0.2225	-5.18351	1.22234
C57	P	Before	CNTNAP2	P	After	-1.83083	1.693616	-1.08	0.2826	-5.1956	1.53394
C57	ChABC	After	CNTNAP2	P	Before	-1.57143	1.562229	-1.01	0.3173	-4.67656	1.5337
CNTNAP2	P	After	CNTNAP2	P	Before	1.33083	1.399767	0.95	0.3462	-1.47912	4.14078
CNTNAP2	ChABC	Before	CNTNAP2	P	Before	1.375	1.516495	0.91	0.3671	-1.63923	4.38923
C57	ChABC	Before	CNTNAP2	P	Before	-1.21429	1.562229	-0.78	0.4391	-4.31941	1.89084
C57	ChABC	After	C57	P	Before	-1.07143	1.562229	-0.69	0.4946	-4.17656	2.0337
C57	ChABC	After	CNTNAP2	ChABC	After	-0.92167	1.473782	-0.63	0.5333	-3.85054	2.00719
C57	P	After	CNTNAP2	ChABC	Before	0.79167	1.516495	0.52	0.603	-2.22256	3.80589
C57	P	After	CNTNAP2	P	After	0.83584	1.693616	0.49	0.6228	-2.52893	4.2006
C57	ChABC	Before	C57	P	Before	-0.71429	1.562229	-0.46	0.6487	-3.81941	2.39084

CNTNAP2	ChABC	After	CNTNAP2	P	Before	-0.64975	1.536154	-0.42	0.6733	-3.70261	2.4031
C57	ChABC	Before	CNTNAP2	ChABC	After	-0.56453	1.473782	-0.38	0.7026	-3.4934	2.36434
C57	P	Before	CNTNAP2	P	Before	-0.5	1.621202	-0.31	0.7585	-3.72234	2.72234
C57	ChABC	After	C57	ChABC	Before	-0.35714	1.213957	-0.29	0.7699	-2.79893	2.08464
C57	P	Before	CNTNAP2	ChABC	After	0.14975	1.536154	0.1	0.9226	-2.9031	3.20261
CNTNAP2	ChABC	Before	CNTNAP2	P	After	0.04417	1.593675	0.03	0.978	-3.12188	3.21022

Mouse	treatment	Time				Least Squares Mean
C57	P	After	A			12.16667
CNTNAP2	ChABC	Before	A	B		11.375
CNTNAP2	P	After	A	B	C	11.33083
CNTNAP2	P	Before	A	B	C	10
C57	P	Before		B	C	9.5
CNTNAP2	ChABC	After	A	B	C	9.350246
C57	ChABC	Before		B	C	8.785714
C57	ChABC	After			C	8.428571

S-FIG-29B

Source	Nparm	DFNum	DFDen	F Ratio	Prob > F
Mouse	1	1	24.8	0.007338	0.9324
treatment	1	1	24.8	3.154487	0.088
Mouse*trea	1	1	24.8	0.209774	0.6509
Time	1	1	23.6	2.79E-05	0.9958
Mouse*Tim	1	1	23.6	0.003283	0.9548
treatment*	1	1	23.6	6.450111	0.0181
Mouse*trea	1	1	23.6	4.416968	0.0465

Mouse	treatment	Time	-Mouse	-treatment	-Time	Difference	Std Error	t Ratio	Prob>|t|	Lower 95%	Upper 95%
CNTNAP2	ChABC	After	CNTNAP2	ChABC	Before	-3.125	1.166612	-2.68	0.0136	-5.54118	-0.70882
CNTNAP2	ChABC	After	CNTNAP2	P	After	-4.63907	2.050443	-2.26	0.029	-8.77894	-0.4992
C57	P	After	CNTNAP2	ChABC	After	3.91667	1.860716	2.1	0.0423	0.14407	7.68926
C57	P	Before	CNTNAP2	ChABC	After	3.58333	1.860716	1.93	0.062	-0.18926	7.35593
CNTNAP2	P	After	CNTNAP2	P	Before	3.05574	1.598968	1.91	0.0673	-0.23368	6.34516
C57	ChABC	After	CNTNAP2	P	After	-3.63907	2.050443	-1.77	0.0833	-7.77894	0.5008
C57	ChABC	Before	CNTNAP2	P	After	-3.38907	2.050443	-1.65	0.1059	-7.52894	0.7508
C57	ChABC	After	C57	P	After	-2.91667	1.860716	-1.57	0.1257	-6.68926	0.85593
C57	ChABC	Before	C57	P	After	-2.66667	1.860716	-1.43	0.1604	-6.43926	1.10593
C57	ChABC	After	C57	P	Before	-2.58333	1.860716	-1.39	0.1735	-6.35593	1.18926
C57	ChABC	Before	C57	P	Before	-2.33333	1.860716	-1.25	0.2179	-6.10593	1.43926
C57	ChABC	After	CNTNAP2	ChABC	Before	-2.125	1.722688	-1.23	0.2253	-5.61774	1.36774
C57	P	After	CNTNAP2	P	Before	2.33333	1.989189	1.17	0.2484	-1.69974	6.36641
C57	ChABC	Before	CNTNAP2	ChABC	Before	-1.875	1.722688	-1.09	0.2836	-5.36774	1.61774
C57	P	Before	CNTNAP2	P	Before	2	1.989189	1.01	0.3213	-2.03307	6.03307
CNTNAP2	ChABC	After	CNTNAP2	P	Before	-1.58333	1.860716	-0.85	0.4004	-5.35593	2.18926
CNTNAP2	ChABC	Before	CNTNAP2	P	Before	1.54167	1.860716	0.83	0.4128	-2.23093	5.31426
CNTNAP2	ChABC	Before	CNTNAP2	P	After	-1.51407	2.050443	-0.74	0.4644	-5.65394	2.6258
C57	ChABC	Before	CNTNAP2	ChABC	After	1.25	1.722688	0.73	0.4727	-2.24274	4.74274
C57	ChABC	After	CNTNAP2	ChABC	After	1	1.722688	0.58	0.5652	-2.49274	4.49274
C57	P	Before	CNTNAP2	P	After	-1.05574	2.167701	-0.49	0.6288	-5.43393	3.32246
C57	P	After	CNTNAP2	ChABC	Before	0.79167	1.860716	0.43	0.673	-2.98093	4.56426

C57	P	After	CNTNAP2	P	After	-0.7224	2.167701	-0.33	0.7406	-5.1006	3.65579
C57	ChABC	After	CNTNAP2	P	Before	-0.58333	1.860716	-0.31	0.7557	-4.35593	3.18926
C57	P	After	C57	P	Before	0.33333	1.347088	0.25	0.8068	-2.45663	3.12329
C57	P	Before	CNTNAP2	ChABC	Before	0.45833	1.860716	0.25	0.8068	-3.31426	4.23093
C57	ChABC	After	C57	ChABC	Before	-0.25	1.166612	-0.21	0.8322	-2.66618	2.16618
C57	ChABC	Before	CNTNAP2	P	Before	-0.33333	1.860716	-0.18	0.8588	-4.10593	3.43926

Mouse	treatment	Time			Least Squares Mean
CNTNAP2	P	After	A		11.88907
C57	P	After	A		11.16667
C57	P	Before	A	B	10.83333
CNTNAP2	ChABC	Before	A		10.375
CNTNAP2	P	Before	A	B	8.833333
C57	ChABC	Before	A	B	8.5
C57	ChABC	After	A	B	8.25
CNTNAP2	ChABC	After		B	7.25

S-FIG-29C

Source	Nparm	DFNum	DFDen	F Ratio	Prob > F
Mouse	1	1	23.2	1.507789	0.2318
treatment	1	1	23.2	0.065314	0.8005
Mouse*trea	1	1	23.2	0.981214	0.3321
Time	1	1	22.2	0.500155	0.4868
Mouse*Tim	1	1	22.2	2.054476	0.1657
treatment*	1	1	22.2	1.615929	0.2168
Mouse*trea	1	1	22.2	1.809157	0.1922

Mouse	treatment	Time	-Mouse	-treatment	-Time	Difference	Std Error	t Ratio	Prob>|t|	Lower 95%	Upper 95%
C57	P	After	C57	P	Before	5	2.107265	2.37	0.0279	0.60268	9.397321
C57	ChABC	After	C57	P	After	-4.5	2.545238	-1.77	0.0848	-9.64557	0.645568
C57	P	Before	CNTNAP2	ChABC	Before	-4.20833	2.380852	-1.77	0.0848	-9.02157	0.604905
C57	ChABC	Before	C57	P	After	-4	2.545238	-1.57	0.124	-9.14557	1.145568
C57	ChABC	After	CNTNAP2	ChABC	Before	-3.70833	2.380852	-1.56	0.1273	-8.52157	1.104905
C57	P	Before	CNTNAP2	ChABC	After	-3.52271	2.45145	-1.44	0.1584	-8.47598	1.430554
C57	ChABC	Before	CNTNAP2	ChABC	Before	-3.20833	2.380852	-1.35	0.1855	-8.02157	1.604905
C57	P	Before	CNTNAP2	P	Before	-3.25378	2.543465	-1.28	0.208	-8.39057	1.883015
C57	ChABC	After	CNTNAP2	ChABC	After	-3.02271	2.45145	-1.23	0.2247	-7.97598	1.930554
C57	ChABC	After	CNTNAP2	P	Before	-2.75378	2.543465	-1.08	0.2853	-7.89057	2.383015
C57	ChABC	Before	CNTNAP2	ChABC	After	-2.52271	2.45145	-1.03	0.3096	-7.47598	2.430554
C57	P	After	CNTNAP2	P	After	2.58711	2.543465	1.02	0.315	-2.54968	7.7239
C57	P	Before	CNTNAP2	P	After	-2.41289	2.543465	-0.95	0.3484	-7.54968	2.7239
C57	ChABC	Before	CNTNAP2	P	Before	-2.25378	2.543465	-0.89	0.3807	-7.39057	2.883015
CNTNAP2	ChABC	Before	CNTNAP2	P	After	1.79544	2.378957	0.75	0.4547	-3.00851	6.599395
C57	ChABC	After	CNTNAP2	P	After	-1.91289	2.543465	-0.75	0.4563	-7.04968	3.2239
C57	P	After	CNTNAP2	P	Before	1.74622	2.543465	0.69	0.4962	-3.39057	6.883015
C57	P	After	CNTNAP2	ChABC	After	1.47729	2.45145	0.6	0.5501	-3.47598	6.430554
C57	ChABC	Before	CNTNAP2	P	After	-1.41289	2.543465	-0.56	0.5816	-6.54968	3.7239
CNTNAP2	ChABC	After	CNTNAP2	P	After	1.10982	2.44961	0.45	0.6529	-3.83507	6.054716
CNTNAP2	ChABC	Before	CNTNAP2	P	Before	0.95456	2.378957	0.4	0.6903	-3.8494	5.758511
C57	ChABC	Before	C57	P	Before	1	2.545238	0.39	0.6965	-4.14557	6.145568

CNTNAP2	P	After	CNTNAP2	P	Before	-0.84088	2.26388	-0.37	0.7131	-5.47759	3.795822
CNTNAP2	ChABC	After	CNTNAP2	ChABC	Before	-0.68562	1.916136	-0.36	0.724	-4.66884	3.297607
C57	P	After	CNTNAP2	ChABC	Before	0.79167	2.380852	0.33	0.7413	-4.02157	5.604905
C57	ChABC	After	C57	ChABC	Before	-0.5	2.107265	-0.24	0.8149	-4.89732	3.897321
C57	ChABC	After	C57	P	Before	0.5	2.545238	0.2	0.8453	-4.64557	5.645568
CNTNAP2	ChABC	After	CNTNAP2	P	Before	0.26894	2.44961	0.11	0.9131	-4.67595	5.213831

Mouse	treatment	Time			Least Squares Mean
C57	P	After	A		13.16667
CNTNAP2	ChABC	Before	A	B	12.375
CNTNAP2	ChABC	After	A	B	11.68938
CNTNAP2	P	Before	A	B	11.42044
CNTNAP2	P	After	A	B	10.57956
C57	ChABC	Before	A	B	9.166667
C57	ChABC	After	A	B	8.666667
C57	P	Before		B	8.166667

S-FIG-30A

Source	Nparm	DFNum	DFDen	F Ratio	Prob > F
Mouse	1	1	24.2	2.570973	0.1218
treatment	1	1	24.2	2.177603	0.1529
Mouse*trea	1	1	24.2	0.28404	0.5989
Time	1	1	24	3.09104	0.0915
Mouse*Tim	1	1	24	0.275673	0.6044
treatment*	1	1	24	0.331835	0.57
Mouse*trea	1	1	24	7.394655	0.012

Mouse	treatment	Time	-Mouse	-treatment	-Time	Difference	Std Error	t Ratio	Prob>|t|	Lower 95%	Upper 95%
C57	ChABC	After	C57	P	After	10.0117	3.688399	2.71	0.0093	2.585	17.4383
C57	ChABC	After	C57	ChABC	Before	8.2125	3.221744	2.55	0.0181	1.538	14.887
C57	P	After	CNTNAP2	P	After	-10.1758	4.442062	-2.29	0.0266	-19.1182	-1.2335
C57	P	After	CNTNAP2	ChABC	After	-8.1829	3.688399	-2.22	0.0316	-15.6096	-0.7562
C57	P	After	CNTNAP2	ChABC	Before	-7.9292	3.688399	-2.15	0.0369	-15.3558	-0.5025
C57	ChABC	After	CNTNAP2	P	Before	7.8633	3.688399	2.13	0.0384	0.4367	15.29
C57	ChABC	Before	CNTNAP2	P	After	-8.3767	4.217635	-1.99	0.053	-16.8671	0.1138
C57	ChABC	Before	CNTNAP2	ChABC	After	-6.3838	3.414794	-1.87	0.068	-13.2595	0.492
CNTNAP2	P	After	CNTNAP2	P	Before	8.0275	4.245429	1.89	0.0693	-0.6794	16.7344
C57	ChABC	Before	CNTNAP2	ChABC	Before	-6.13	3.414794	-1.8	0.0793	-13.0058	0.7458
C57	ChABC	After	C57	P	Before	6.2717	3.688399	1.7	0.0959	-1.155	13.6983
CNTNAP2	ChABC	After	CNTNAP2	P	Before	6.0346	3.688399	1.64	0.1087	-1.3921	13.4613
CNTNAP2	ChABC	Before	CNTNAP2	P	Before	5.7808	3.688399	1.57	0.124	-1.6458	13.2075
C57	P	Before	CNTNAP2	P	After	-6.4358	4.442062	-1.45	0.1542	-15.3782	2.5065
C57	P	Before	CNTNAP2	ChABC	After	-4.4429	3.688399	-1.2	0.2346	-11.8696	2.9838
C57	P	Before	CNTNAP2	ChABC	Before	-4.1892	3.688399	-1.14	0.262	-11.6158	3.2375
C57	P	After	C57	P	Before	-3.74	3.720149	-1.01	0.3255	-11.4471	3.9671
C57	ChABC	After	CNTNAP2	ChABC	Before	2.0825	3.414794	0.61	0.545	-4.7933	8.9583
C57	P	After	CNTNAP2	P	Before	-2.1483	3.943065	-0.54	0.5885	-10.0878	5.7911
C57	ChABC	After	CNTNAP2	ChABC	After	1.8287	3.414794	0.54	0.5949	-5.047	8.7045
CNTNAP2	ChABC	Before	CNTNAP2	P	After	-2.2467	4.217635	-0.53	0.5968	-10.7371	6.2438

C57	ChABC	Before	C57	P	Before	-1.9408	3.688399	-0.53	0.6013	-9.3675	5.4858
C57	ChABC	Before	C57	P	After	1.7992	3.688399	0.49	0.628	-5.6275	9.2258
CNTNAP2	ChABC	After	CNTNAP2	P	After	-1.9929	4.217635	-0.47	0.6388	-10.4833	6.4975
C57	P	Before	CNTNAP2	P	Before	1.5917	3.943065	0.4	0.6884	-6.3478	9.5311
C57	ChABC	Before	CNTNAP2	P	Before	-0.3492	3.688399	-0.09	0.925	-7.7758	7.0775
CNTNAP2	ChABC	After	CNTNAP2	ChABC	Before	0.2538	3.221744	0.08	0.9379	-6.4208	6.9283
C57	ChABC	After	CNTNAP2	P	After	-0.1642	4.217635	-0.04	0.9691	-8.6546	8.3263

Mouse	treatment	Time				Least Squares Mean
CNTNAP2	P	After	A	B		39.11417
C57	ChABC	After	A			38.95
CNTNAP2	ChABC	After	A	B		37.12125
CNTNAP2	ChABC	Before	A	B		36.8675
C57	P	Before	A	B	C	32.67833
CNTNAP2	P	Before		B	C	31.08667
C57	ChABC	Before		B	C	30.7375
C57	P	After			C	28.93833

S-FIG-30B

Source	Nparm	DFNum	DFDen	F Ratio	Prob > F
Mouse	1	1	22.3	0.543105	0.4688
treatment	1	1	22.3	0.004904	0.9448
Mouse*trea	1	1	22.3	0.080486	0.7793
Time	1	1	21.4	2.894075	0.1034
Mouse*Tim	1	1	21.4	0.449299	0.5098
treatment*	1	1	21.4	0.320919	0.5769
Mouse*trea	1	1	21.4	0.100988	0.7537

Mouse	treatment	Time	-Mouse	-treatment	-Time	Difference	Std Error	t Ratio	Prob>|t|	Lower 95%	Upper 95%
C57	ChABC	After	C57	ChABC	Before	2.78667	2.925165	0.95	0.3527	-3.33451	8.90784
C57	ChABC	After	C57	P	After	0.89167	3.408266	0.26	0.7949	-5.99469	7.77802
C57	ChABC	After	C57	P	Before	1.105	3.408266	0.32	0.7475	-5.78136	7.99136
C57	ChABC	After	CNTNAP2	ChABC	After	0.325	3.286763	0.1	0.9217	-6.31301	6.96301
C57	ChABC	After	CNTNAP2	ChABC	Before	4.1375	3.188141	1.3	0.2017	-2.3041	10.5791
C57	ChABC	After	CNTNAP2	P	After	1.34081	3.413961	0.39	0.6965	-5.55161	8.23323
C57	ChABC	After	CNTNAP2	P	Before	4.42954	3.413961	1.3	0.2016	-2.46288	11.32196
C57	ChABC	Before	C57	P	After	-1.895	3.408266	-0.56	0.5813	-8.78136	4.99136
C57	ChABC	Before	C57	P	Before	-1.68167	3.408266	-0.49	0.6244	-8.56802	5.20469
C57	ChABC	Before	CNTNAP2	ChABC	After	-2.46167	3.286763	-0.75	0.4582	-9.09968	4.17635
C57	ChABC	Before	CNTNAP2	ChABC	Before	1.35083	3.188141	0.42	0.674	-5.09076	7.79243
C57	ChABC	Before	CNTNAP2	P	After	-1.44586	3.413961	-0.42	0.6741	-8.33828	5.44656
C57	ChABC	Before	CNTNAP2	P	Before	1.64288	3.413961	0.48	0.6329	-5.24955	8.5353
C57	P	After	C57	P	Before	0.21333	2.925165	0.07	0.9426	-5.90784	6.33451
C57	P	After	CNTNAP2	ChABC	After	-0.56667	3.286763	-0.17	0.864	-7.20468	6.07135
C57	P	After	CNTNAP2	ChABC	Before	3.24583	3.188141	1.02	0.3147	-3.19576	9.68743
C57	P	After	CNTNAP2	P	After	0.44914	3.413961	0.13	0.896	-6.44328	7.34156
C57	P	After	CNTNAP2	P	Before	3.53788	3.413961	1.04	0.3061	-3.35455	10.4303
C57	P	Before	CNTNAP2	ChABC	After	-0.78	3.286763	-0.24	0.8136	-7.41801	5.85801
C57	P	Before	CNTNAP2	ChABC	Before	3.0325	3.188141	0.95	0.3472	-3.4091	9.4741
C57	P	Before	CNTNAP2	P	After	0.23581	3.413961	0.07	0.9453	-6.65661	7.12823
C57	P	Before	CNTNAP2	P	Before	3.32454	3.413961	0.97	0.3358	-3.56788	10.21696

CNTNAP2	ChABC	After	CNTNAP2	ChABC	Before	3.8125	2.656315	1.44	0.1664	-1.72217	9.34718
CNTNAP2	ChABC	After	CNTNAP2	P	After	1.01581	3.292668	0.31	0.7592	-5.62928	7.6609
CNTNAP2	ChABC	After	CNTNAP2	P	Before	4.10454	3.292668	1.25	0.2195	-2.54055	10.74964
CNTNAP2	ChABC	Before	CNTNAP2	P	After	-2.79669	3.194229	-0.88	0.3863	-9.24488	3.65149
CNTNAP2	ChABC	Before	CNTNAP2	P	Before	0.29204	3.194229	0.09	0.9276	-6.15614	6.74023
CNTNAP2	P	After	CNTNAP2	P	Before	3.08873	3.115284	0.99	0.3301	-3.29781	9.47527

Mouse	treatment	Time		Least Squares Mean
C57	ChABC	After	A	30.475
CNTNAP2	ChABC	After	A	30.15
C57	P	After	A	29.58333
C57	P	Before	A	29.37
CNTNAP2	P	After	A	29.13419
C57	ChABC	Before	A	27.68833
CNTNAP2	ChABC	Before	A	26.3375
CNTNAP2	P	Before	A	26.04546

S-FIG-31A

Source	Nparm	DFNum	DFDen	F Ratio	Prob > F
Mouse	1	1	24.8	2.531567	0.1243
treatment	1	1	24.8	0.001861	0.9659
Mouse*trea	1	1	24.8	0.133846	0.7176
Time	1	1	25	1.102922	0.3037
Mouse*Tim	1	1	25	0.278075	0.6026
treatment*	1	1	25	0.122166	0.7296
Mouse*trea	1	1	25	0.879934	0.3572

Mouse	treatment	Time	-Mouse	-treatment	-Time	Difference	Std Error	t Ratio	Prob>|t|	Lower 95%	Upper 95%
C57	P	After	CNTNAP2	ChABC	Before	-8.45542	4.589944	-1.84	0.0719	-17.696	0.78513
C57	P	After	CNTNAP2	P	Before	-7.93833	4.906856	-1.62	0.1126	-17.8169	1.94023
C57	ChABC	Before	CNTNAP2	ChABC	Before	-6.425	4.249462	-1.51	0.1374	-14.9801	2.13008
C57	ChABC	Before	CNTNAP2	P	Before	-5.90792	4.589944	-1.29	0.2045	-15.1485	3.33263
CNTNAP2	ChABC	After	CNTNAP2	ChABC	Before	-5.35	4.418964	-1.21	0.2381	-14.4826	3.78259
C57	ChABC	After	CNTNAP2	ChABC	Before	-4.53625	4.249462	-1.07	0.2914	-13.0913	4.01883
CNTNAP2	ChABC	After	CNTNAP2	P	Before	-4.83292	4.589944	-1.05	0.2979	-14.0735	4.40763
C57	P	After	CNTNAP2	P	After	-5.49556	5.528009	-0.99	0.3254	-16.6235	5.63235
C57	ChABC	After	CNTNAP2	P	Before	-4.01917	4.589944	-0.88	0.3858	-13.2597	5.22138
C57	P	After	C57	P	Before	-4.47167	5.10258	-0.88	0.3897	-15.0171	6.07373
C57	P	Before	CNTNAP2	ChABC	Before	-3.98375	4.589944	-0.87	0.39	-13.2243	5.2568
C57	ChABC	After	C57	P	After	3.91917	4.589944	0.85	0.3976	-5.3214	13.15971
C57	P	Before	CNTNAP2	P	Before	-3.46667	4.906856	-0.71	0.4835	-13.3452	6.41189
C57	P	After	CNTNAP2	ChABC	After	-3.10542	4.589944	-0.68	0.5021	-12.346	6.13513
C57	ChABC	Before	CNTNAP2	P	After	-3.46515	5.248736	-0.66	0.5124	-14.0308	7.1005
CNTNAP2	ChABC	Before	CNTNAP2	P	After	2.95985	5.248736	0.56	0.5756	-7.6058	13.5255
C57	ChABC	Before	C57	P	Before	-2.44125	4.589944	-0.53	0.5974	-11.6818	6.7993
CNTNAP2	ChABC	After	CNTNAP2	P	After	-2.39015	5.248736	-0.46	0.651	-12.9558	8.1755
C57	ChABC	Before	C57	P	After	2.03042	4.589944	0.44	0.6603	-7.2101	11.27096
CNTNAP2	P	After	CNTNAP2	P	Before	-2.44277	5.702452	-0.43	0.6716	-14.1144	9.22885
C57	ChABC	After	C57	ChABC	Before	1.88875	4.418964	0.43	0.673	-7.2438	11.02134
C57	ChABC	After	CNTNAP2	P	After	-1.5764	5.248736	-0.3	0.7653	-12.142	8.98925

C57	P	Before	CNTNAP2	ChABC	After	1.36625	4.589944	0.3	0.7673	-7.8743	10.6068
C57	ChABC	Before	CNTNAP2	ChABC	After	-1.075	4.249462	-0.25	0.8014	-9.6301	7.48008
C57	ChABC	After	CNTNAP2	ChABC	After	0.81375	4.249462	0.19	0.849	-7.7413	9.36883
C57	P	Before	CNTNAP2	P	After	-1.0239	5.528009	-0.19	0.8539	-12.1518	10.10401
C57	ChABC	After	C57	P	Before	-0.5525	4.589944	-0.12	0.9047	-9.793	8.68805
CNTNAP2	ChABC	Before	CNTNAP2	P	Before	0.51708	4.589944	0.11	0.9108	-8.7235	9.75763

Mouse	treatment	Time		Least Squares Mean
CNTNAP2	ChABC	Before	A	54.02875
CNTNAP2	P	Before	A	53.51167
CNTNAP2	P	After	A	51.0689
C57	P	Before	A	50.045
C57	ChABC	After	A	49.4925
CNTNAP2	ChABC	After	A	48.67875
C57	ChABC	Before	A	47.60375
C57	P	After	A	45.57333

S-FIG-31B

Source	Nparm	DFNum	DFDen	F Ratio	Prob > F
Mouse	1	1	22.9	0.012991	0.9102
treatment	1	1	22.9	0.041024	0.8413
Mouse*trea	1	1	22.9	0.103254	0.7509
Time	1	1	21.1	0.034786	0.8538
Mouse*Tim	1	1	21.1	4.61E-05	0.9946
treatment*	1	1	21.1	4.938877	0.0373
Mouse*trea	1	1	21.1	0.823216	0.3745

Mouse	treatment	Time	-Mouse	-treatment	-Time	Difference	Std Error	t Ratio	Prob>|t|	Lower 95%	Upper 95%
C57	ChABC	After	C57	ChABC	Before	5.11	3.056515	1.67	0.1109	-1.2872	11.50718
C57	P	After	C57	P	Before	-4.555	3.056515	-1.49	0.1526	-10.9522	1.84218
C57	ChABC	Before	C57	P	Before	-5.13333	4.331705	-1.19	0.244	-13.9262	3.65951
C57	ChABC	Before	CNTNAP2	ChABC	After	-4.82195	4.1508	-1.16	0.2529	-13.2373	3.59336
C57	ChABC	After	C57	P	After	4.53167	4.331705	1.05	0.3026	-4.2612	13.32451
C57	P	After	CNTNAP2	ChABC	After	-4.24361	4.1508	-1.02	0.3134	-12.6589	4.17169
C57	P	Before	CNTNAP2	P	After	3.66866	4.285761	0.86	0.3974	-5.0101	12.34745
C57	ChABC	After	CNTNAP2	P	After	3.64533	4.285761	0.85	0.4004	-5.0335	12.32412
CNTNAP2	ChABC	After	CNTNAP2	ChABC	Before	2.32903	2.796005	0.83	0.4147	-3.5033	8.16132
CNTNAP2	ChABC	After	CNTNAP2	P	After	3.35728	4.102831	0.82	0.4182	-4.9414	11.65596
C57	ChABC	Before	CNTNAP2	P	Before	-3.19674	4.285761	-0.75	0.4604	-11.8755	5.48204
C57	P	Before	CNTNAP2	ChABC	Before	2.64042	4.051939	0.65	0.5189	-5.5845	10.86537
C57	ChABC	After	CNTNAP2	ChABC	Before	2.61708	4.051939	0.65	0.5226	-5.6079	10.84203
C57	ChABC	Before	CNTNAP2	ChABC	Before	-2.49292	4.051939	-0.62	0.5424	-10.7179	5.73203
C57	P	After	CNTNAP2	P	Before	-2.61841	4.285761	-0.61	0.5449	-11.2972	6.06037
CNTNAP2	P	After	CNTNAP2	P	Before	-1.73207	3.412523	-0.51	0.616	-8.7454	5.28129
C57	P	After	CNTNAP2	ChABC	Before	-1.91458	4.051939	-0.47	0.6395	-10.1395	6.31037
C57	P	Before	CNTNAP2	P	Before	1.93659	4.285761	0.45	0.654	-6.7422	10.61537
C57	ChABC	After	CNTNAP2	P	Before	1.91326	4.285761	0.45	0.6579	-6.7655	10.59204
CNTNAP2	ChABC	After	CNTNAP2	P	Before	1.6252	4.102831	0.4	0.6942	-6.6735	9.92388
C57	ChABC	Before	CNTNAP2	P	After	-1.46467	4.285761	-0.34	0.7344	-10.1435	7.21412
CNTNAP2	ChABC	Before	CNTNAP2	P	After	1.02825	4.002785	0.26	0.7987	-7.075	9.13151

C57	P	After	CNTNAP2	P	After	-0.88634	4.285761	-0.21	0.8373	-9.5651	7.79245
CNTNAP2	ChABC	Before	CNTNAP2	P	Before	-0.70383	4.002785	-0.18	0.8614	-8.8071	7.39944
C57	ChABC	Before	C57	P	After	-0.57833	4.331705	-0.13	0.8946	-9.3712	8.21451
C57	P	Before	CNTNAP2	ChABC	After	0.31139	4.1508	0.08	0.9406	-8.1039	8.72669
C57	ChABC	After	CNTNAP2	ChABC	After	0.28805	4.1508	0.07	0.9451	-8.1273	8.70336
C57	ChABC	After	C57	P	Before	-0.02333	4.331705	-0.01	0.9957	-8.8162	8.76951

Mouse	treatment	Time		Least Squares Mean
C57	P	Before	A	48.01667
C57	ChABC	After	A	47.99333
CNTNAP2	ChABC	After	A	47.70528
CNTNAP2	P	Before	A	46.08008
CNTNAP2	ChABC	Before	A	45.37625
CNTNAP2	P	After	A	44.348
C57	P	After	A	43.46167
C57	ChABC	Before	A	42.88333

S-FIG-32A

Fit Mixed

Fixed Effects Tests

Source	Nparm	DFNum	DFDen	F Ratio	Prob > F
Mouse	1	1	50.9	0.0104388	0.9190
treatment	1	1	50.9	1.0354406	0.3137
Mouse*treatment	1	1	50.9	0.2948771	0.5895
Time	1	1	49.6	0.5346504	0.4681
Mouse*Time	1	1	49.6	0.0380502	0.8461
treatment*Time	1	1	49.6	0.4120298	0.5239
Mouse*treatment*Time	1	1	49.6	0.6139649	0.4370

Multiple Comparisons for Mouse*treatment*Time

Student's t All Pairwise Comparisons

All Pairwise Differences

Mouse	treatment	Time	-Mouse	-treatment	-Time	Difference	Std Error	t Ratio	Prob>|t|	Lower 95%	Upper 95%
C57	ChABC	After	C57	ChABC	Before	-0.42857	1.038848	-0.41	0.6818	-2.51704	1.659895
C57	ChABC	After	C57	P	After	-1.25000	1.429885	-0.87	0.3845	-4.09401	1.594011
C57	ChABC	After	C57	P	Before	-1.83333	1.429885	-1.28	0.2034	-4.67734	1.010677
C57	ChABC	After	CNTNAP2	ChABC	After	-0.05352	1.347510	-0.04	0.9684	-2.73312	2.626075
C57	ChABC	After	CNTNAP2	ChABC	Before	-1.12500	1.330165	-0.85	0.4001	-3.77067	1.520671
C57	ChABC	After	CNTNAP2	P	After	-1.23577	1.493435	-0.83	0.4102	-4.20424	1.732690
C57	ChABC	After	CNTNAP2	P	Before	-0.75000	1.429885	-0.52	0.6013	-3.59401	2.094011
C57	ChABC	Before	C57	P	After	-0.82143	1.429885	-0.57	0.5672	-3.66544	2.022582
C57	ChABC	Before	C57	P	Before	-1.40476	1.429885	-0.98	0.3287	-4.24877	1.439249
C57	ChABC	Before	CNTNAP2	ChABC	After	0.37505	1.347510	0.28	0.7814	-2.30455	3.054647
C57	ChABC	Before	CNTNAP2	ChABC	Before	-0.69643	1.330165	-0.52	0.6020	-3.34210	1.949242
C57	ChABC	Before	CNTNAP2	P	After	-0.80720	1.493435	-0.54	0.5902	-3.77567	2.161261
C57	ChABC	Before	CNTNAP2	P	Before	-0.32143	1.429885	-0.22	0.8227	-3.16544	2.522582
C57	P	After	C57	P	Before	-0.58333	1.122084	-0.52	0.6055	-2.83914	1.672469
C57	P	After	CNTNAP2	ChABC	After	1.19648	1.404656	0.85	0.3967	-1.59680	3.989759
C57	P	After	CNTNAP2	ChABC	Before	0.12500	1.388025	0.09	0.9285	-2.63575	2.885753
C57	P	After	CNTNAP2	P	After	0.01423	1.545193	0.01	0.9927	-3.05724	3.085690
C57	P	After	CNTNAP2	P	Before	0.50000	1.483861	0.34	0.7370	-2.45137	3.451369
C57	P	Before	CNTNAP2	ChABC	After	1.77981	1.404656	1.27	0.2086	-1.01347	4.573092
C57	P	Before	CNTNAP2	ChABC	Before	0.70833	1.388025	0.51	0.6112	-2.05242	3.469086
[illegible]	[illegible]	[illegible]	[illegible]	[illegible]	[illegible]	[illegible]	[illegible]	[illegible]	[illegible]	[illegible]	[illegible]

C57	P	Before	CNTNAP2	P	After	0.59756	1.545193	0.39	0.6999	-2.47390	3.669024
C57	P	Before	CNTNAP2	P	Before	1.08333	1.483861	0.73	0.4674	-1.86804	4.034703
CNTNAP2	ChABC	After	CNTNAP2	ChABC	Before	-1.07148	0.995364	-1.08	0.2869	-3.07122	0.928261
CNTNAP2	ChABC	After	CNTNAP2	P	After	-1.18225	1.469298	-0.80	0.4232	-4.10225	1.737741
CNTNAP2	ChABC	After	CNTNAP2	P	Before	-0.69648	1.404656	-0.50	0.6213	-3.48976	2.096803
CNTNAP2	ChABC	Before	CNTNAP2	P	After	-0.11077	1.453407	-0.08	0.9394	-2.99958	2.778032
CNTNAP2	ChABC	Before	CNTNAP2	P	Before	0.37500	1.388025	0.27	0.7877	-2.38575	3.135753
CNTNAP2	P	After	CNTNAP2	P	Before	0.48577	1.202019	0.40	0.6878	-1.92649	2.898035

All Pairwise Differences Connecting Letters

Mouse	treatment	Time		Least Squares Mean
C57	P	Before	A	11.833333
C57	P	After	A	11.250000
CNTNAP2	P	After	A	11.235774
CNTNAP2	ChABC	Before	A	11.125000
CNTNAP2	P	Before	A	10.750000
C57	ChABC	Before	A	10.428571
CNTNAP2	ChABC	After	A	10.053522
C57	ChABC	After	A	10.000000

Levels not connected by same letter are significantly different.

S-FIG-32B

Fit Mixed Gender=M

Fixed Effects Tests

Source	Nparm	DFNum	DFDen	F Ratio	Prob > F
Mouse	1	1	23.3	0.2824125	0.6002
treatment	1	1	23.3	9.5129724	0.0052*
Mouse*treatment	1	1	23.3	0.2175958	0.6452
Time	1	1	22.8	0.0217175	0.8841
Mouse*Time	1	1	22.8	1.2731434	0.2709
treatment*Time	1	1	22.8	0.1687175	0.6851
Mouse*treatment*Time	1	1	22.8	2.8441391	0.1054

Multiple Comparisons for Mouse*treatment*Time

Student's t All Pairwise Comparisons

All Pairwise Differences

Mouse	treatment	Time	-Mouse	-treatment	-Time	Difference	Std Error	t Ratio	Prob>|t|	Lower 95%	Upper 95%
C57	P	Before	CNTNAP2	ChABC	After	4.91667	1.693269	2.90	0.0058*	1.50376	8.32957
C57	ChABC	After	C57	P	Before	-4.41667	1.693269	-2.61	0.0124*	-7.82957	-1.00376
C57	ChABC	Before	C57	P	Before	-4.41667	1.693269	-2.61	0.0124*	-7.82957	-1.00376
C57	P	Before	CNTNAP2	ChABC	Before	4.04167	1.693269	2.39	0.0214*	0.62876	7.45457
CNTNAP2	ChABC	After	CNTNAP2	P	After	-4.16292	1.929079	-2.16	0.0363*	-8.04753	-0.27831
C57	ChABC	After	CNTNAP2	P	After	-3.66292	1.929079	-1.90	0.0640	-7.54753	0.22169
C57	ChABC	Before	CNTNAP2	P	After	-3.66292	1.929079	-1.90	0.0640	-7.54753	0.22169
C57	P	Before	CNTNAP2	P	Before	3.16667	1.810181	1.75	0.0872	-0.48188	6.81521
C57	P	After	CNTNAP2	ChABC	After	2.91667	1.693269	1.72	0.0920	-0.49624	6.32957
CNTNAP2	ChABC	Before	CNTNAP2	P	After	-3.28792	1.929079	-1.70	0.0952	-7.17253	0.59669
C57	ChABC	After	C57	P	After	-2.41667	1.693269	-1.43	0.1606	-5.82957	0.99624
C57	ChABC	Before	C57	P	After	-2.41667	1.693269	-1.43	0.1606	-5.82957	0.99624
CNTNAP2	P	After	CNTNAP2	P	Before	2.41292	1.849089	1.30	0.2034	-1.38891	6.21476
C57	P	After	C57	P	Before	-2.00000	1.601544	-1.25	0.2253	-5.32782	1.32782
C57	P	After	CNTNAP2	ChABC	Before	2.04167	1.693269	1.21	0.2344	-1.37124	5.45457
CNTNAP2	ChABC	After	CNTNAP2	P	Before	-1.75000	1.693269	-1.03	0.3070	-5.16290	1.66290
C57	ChABC	After	CNTNAP2	P	Before	-1.25000	1.693269	-0.74	0.4643	-4.66290	2.16290
C57	ChABC	Before	CNTNAP2	P	Before	-1.25000	1.693269	-0.74	0.4643	-4.66290	2.16290
C57	P	After	CNTNAP2	P	Before	1.16667	1.810181	0.64	0.5226	-2.48188	4.81521
CNTNAP2	ChABC	After	CNTNAP2	ChABC	Before	-0.87500	1.386978	-0.63	0.5348	-3.75698	2.00698
C57	P	After	CNTNAP2	P	After	-1.24626	2.032471	-0.61	0.5428	-5.33935	2.84684

CNTNAP2	ChABC	Before	CNTNAP2	P	Before	-0.87500	1.693269	-0.52	0.6079	-4.28790	2.53790
C57	P	Before	CNTNAP2	P	After	0.75374	2.032471	0.37	0.7125	-3.33935	4.84684
C57	ChABC	After	CNTNAP2	ChABC	After	0.50000	1.567663	0.32	0.7513	-2.65973	3.65973
C57	ChABC	Before	CNTNAP2	ChABC	After	0.50000	1.567663	0.32	0.7513	-2.65973	3.65973
C57	ChABC	After	CNTNAP2	ChABC	Before	-0.37500	1.567663	-0.24	0.8121	-3.53473	2.78473
C57	ChABC	Before	CNTNAP2	ChABC	Before	-0.37500	1.567663	-0.24	0.8121	-3.53473	2.78473
C57	ChABC	After	C57	ChABC	Before	0.00000	1.386978	0.00	1.0000	-2.88198	2.88198

All Pairwise Differences Connecting Letters

Mouse	treatment	Time				Least Squares Mean
C57	P	Before	A			13.666667
CNTNAP2	P	After	A	B		12.912923
C57	P	After	A	B	C	11.666667
CNTNAP2	P	Before	A	B	C	10.500000
CNTNAP2	ChABC	Before		B	C	9.625000
C57	ChABC	After		B	C	9.250000
C57	ChABC	Before		B	C	9.250000
CNTNAP2	ChABC	After			C	8.750000

Levels not connected by same letter are significantly different.

S-FIG-32C

Fit Mixed Gender=F

Fixed Effects Tests

Source	Nparm	DFNum	DFDen	F Ratio	Prob > F
Mouse	1	1	24.8	0.0536684	0.8187
treatment	1	1	24.8	0.9746541	0.3331
Mouse*treatment	1	1	24.8	0.0303508	0.8631
Time	1	1	23.0	0.3735222	0.5471
Mouse*Time	1	1	23.0	0.2557054	0.6179
treatment*Time	1	1	23.0	0.5795731	0.4542
Mouse*treatment*Time	1	1	23.0	0.1592877	0.6935

Multiple Comparisons for Mouse*treatment*Time

Student's t All Pairwise Comparisons

All Pairwise Differences

Mouse	treatment	Time	-Mouse	-treatment	-Time	Difference	Std Error	t Ratio	Prob>|t|	Lower 95%	Upper 95%
C57	ChABC	After	C57	ChABC	Before	-1.00000	1.563049	-0.64	0.5293	-4.25147	2.251472
C57	ChABC	After	C57	P	After	0.16667	2.265304	0.07	0.9418	-4.42885	4.762179
C57	ChABC	After	C57	P	Before	1.00000	2.265304	0.44	0.6616	-3.59551	5.595513
C57	ChABC	After	CNTNAP2	ChABC	After	-0.45664	2.168478	-0.21	0.8344	-4.85097	3.937694
C57	ChABC	After	CNTNAP2	ChABC	Before	-1.62500	2.118998	-0.77	0.4482	-5.92371	2.673708
C57	ChABC	After	CNTNAP2	P	After	0.80485	2.236451	0.36	0.7209	-3.72248	5.332188
C57	ChABC	After	CNTNAP2	P	Before	0.20872	2.236451	0.09	0.9261	-4.31862	4.736050
C57	ChABC	Before	C57	P	After	1.16667	2.265304	0.52	0.6097	-3.42885	5.762179
C57	ChABC	Before	C57	P	Before	2.00000	2.265304	0.88	0.3832	-2.59551	6.595513
C57	ChABC	Before	CNTNAP2	ChABC	After	0.54336	2.168478	0.25	0.8035	-3.85097	4.937694
C57	ChABC	Before	CNTNAP2	ChABC	Before	-0.62500	2.118998	-0.29	0.7697	-4.92371	3.673708
C57	ChABC	Before	CNTNAP2	P	After	1.80485	2.236451	0.81	0.4247	-2.72248	6.332188
C57	ChABC	Before	CNTNAP2	P	Before	1.20872	2.236451	0.54	0.5920	-3.31862	5.736050
C57	P	After	C57	P	Before	0.83333	1.563049	0.53	0.5996	-2.41814	4.084805
C57	P	After	CNTNAP2	ChABC	After	-0.62330	2.168478	-0.29	0.7754	-5.01764	3.771027
C57	P	After	CNTNAP2	ChABC	Before	-1.79167	2.118998	-0.85	0.4034	-6.09038	2.507042
C57	P	After	CNTNAP2	P	After	0.63819	2.236451	0.29	0.7769	-3.88915	5.165521
C57	P	After	CNTNAP2	P	Before	0.04205	2.236451	0.02	0.9851	-4.48529	4.569383
C57	P	Before	CNTNAP2	ChABC	After	-1.45664	2.168478	-0.67	0.5059	-5.85097	2.937694
C57	P	Before	CNTNAP2	ChABC	Before	-2.62500	2.118998	-1.24	0.2235	-6.92371	1.673708
C57	P	Before	CNTNAP2	P	After	-0.19515	2.236451	-0.09	0.9309	-4.72248	4.332188

[illegible]	[illegible]	[illegible]	[illegible]	[illegible]	[illegible]	[illegible]	[illegible]	[illegible]	[illegible]	[illegible]	[illegible]
C57	P	Before	CNTNAP2	P	Before	-0.79128	2.236451	-0.35	0.7254	-5.31862	3.736050
CNTNAP2	ChABC	After	CNTNAP2	ChABC	Before	-1.16836	1.429855	-0.82	0.4227	-4.13471	1.797984
CNTNAP2	ChABC	After	CNTNAP2	P	After	1.26149	2.138318	0.59	0.5586	-3.06269	5.585670
CNTNAP2	ChABC	After	CNTNAP2	P	Before	0.66535	2.138318	0.31	0.7573	-3.65883	4.989532
CNTNAP2	ChABC	Before	CNTNAP2	P	After	2.42985	2.088124	1.16	0.2518	-1.79606	6.655763
CNTNAP2	ChABC	Before	CNTNAP2	P	Before	1.83372	2.088124	0.88	0.3853	-2.39219	6.059624
CNTNAP2	P	After	CNTNAP2	P	Before	-0.59614	1.747483	-0.34	0.7356	-4.17752	2.985245

All Pairwise Differences Connecting Letters

Mouse	treatment	Time		Least Squares Mean
CNTNAP2	ChABC	Before	A	12.625000
C57	ChABC	Before	A	12.000000
CNTNAP2	ChABC	After	A	11.456638
C57	ChABC	After	A	11.000000
C57	P	After	A	10.833333
CNTNAP2	P	Before	A	10.791285
CNTNAP2	P	After	A	10.195147
C57	P	Before	A	10.000000

Levels not connected by same letter are significantly different.

S-FIG-33A

Fit Mixed

Fixed Effects Tests

Source	Nparm	DFNum	DFDen	F Ratio	Prob > F
Mouse	1	1	50.6	2.4759553	0.1218
treatment	1	1	50.6	1.4629982	0.2321
Mouse*treatment	1	1	50.6	0.7792502	0.3815
Time	1	1	50.1	3.6339896	0.0624
Mouse*Time	1	1	50.1	0.7705606	0.3842
treatment*Time	1	1	50.1	0.6040755	0.4407
Mouse*treatment*Time	1	1	50.1	3.7979087	0.0569

Multiple Comparisons for Mouse*treatment*Time

Least Squares Means Estimates

Mouse	treatment	Time	Estimate	Std Error	DF	Lower 95%	Upper 95%
C57	ChABC	After	16.605000	1.4534866	94.918	13.719432	19.490568
C57	ChABC	Before	18.782857	1.4534866	94.918	15.897289	21.668425
C57	P	After	17.389167	1.5699449	94.918	14.272397	20.505936
C57	P	Before	17.247500	1.5699449	94.918	14.130730	20.364270
CNTNAP2	ChABC	After	16.871264	1.4048794	95.402	14.082377	19.660150
CNTNAP2	ChABC	Before	16.928750	1.3596122	94.918	14.229548	19.627952
CNTNAP2	P	After	11.768010	1.7220019	96.074	8.349896	15.186123
CNTNAP2	P	Before	17.222500	1.5699449	94.918	14.105730	20.339270

Student's t All Pairwise Comparisons

All Pairwise Differences

Mouse	treatment	Time	-Mouse	-treatment	-Time	Difference	Std Error	t Ratio	Prob>|t|	Lower 95%	Upper 95%
C57	ChABC	Before	CNTNAP2	P	After	7.01485	2.253423	3.11	0.0024*	2.54163	11.4881
CNTNAP2	P	After	CNTNAP2	P	Before	-5.45449	2.161843	-2.52	0.0147*	-9.79084	-1.1181
C57	P	After	CNTNAP2	P	After	5.62116	2.330240	2.41	0.0178*	0.99542	10.2469
CNTNAP2	ChABC	Before	CNTNAP2	P	After	5.16074	2.194046	2.35	0.0207*	0.80541	9.5161
C57	P	Before	CNTNAP2	P	After	5.47949	2.330240	2.35	0.0208*	0.85376	10.1052
CNTNAP2	ChABC	After	CNTNAP2	P	After	5.10325	2.222381	2.30	0.0238*	0.69176	9.5147
C57	ChABC	After	CNTNAP2	P	After	4.83699	2.253423	2.15	0.0344*	0.36377	9.3102
C57	ChABC	After	C57	ChABC	Before	-2.17786	1.891258	-1.15	0.2552	-5.97954	1.6238
C57	ChABC	Before	CNTNAP2	ChABC	After	1.91159	2.021462	0.95	0.3467	-2.10143	5.9246

C57	ChABC	Before	CNTNAP2	ChABC	Before	1.85411	1.990268	0.93	0.3539	-2.09712	5.8053
C57	ChABC	Before	CNTNAP2	P	Before	1.56036	2.139474	0.73	0.4676	-2.68708	5.8078
C57	ChABC	Before	C57	P	Before	1.53536	2.139474	0.72	0.4747	-2.71208	5.7828
C57	ChABC	Before	C57	P	After	1.39369	2.139474	0.65	0.5164	-2.85375	5.6411
C57	ChABC	After	C57	P	After	-0.78417	2.139474	-0.37	0.7148	-5.03161	3.4633
C57	ChABC	After	C57	P	Before	-0.64250	2.139474	-0.30	0.7646	-4.88994	3.6049
C57	ChABC	After	CNTNAP2	P	Before	-0.61750	2.139474	-0.29	0.7735	-4.86494	3.6299
C57	P	After	CNTNAP2	ChABC	After	0.51790	2.106754	0.25	0.8063	-3.66445	4.7003
C57	P	After	CNTNAP2	ChABC	Before	0.46042	2.076842	0.22	0.8250	-3.66268	4.5835
C57	P	Before	CNTNAP2	ChABC	After	0.37624	2.106754	0.18	0.8586	-3.80612	4.5586
CNTNAP2	ChABC	After	CNTNAP2	P	Before	-0.35124	2.106754	-0.17	0.8679	-4.53359	3.8311
C57	ChABC	After	CNTNAP2	ChABC	Before	-0.32375	1.990268	-0.16	0.8711	-4.27498	3.6275
C57	P	Before	CNTNAP2	ChABC	Before	0.31875	2.076842	0.15	0.8783	-3.80435	4.4418
CNTNAP2	ChABC	Before	CNTNAP2	P	Before	-0.29375	2.076842	-0.14	0.8878	-4.41685	3.8293
C57	ChABC	After	CNTNAP2	ChABC	After	-0.26626	2.021462	-0.13	0.8955	-4.27929	3.7468
C57	P	After	CNTNAP2	P	Before	0.16667	2.220237	0.08	0.9403	-4.24111	4.5744
C57	P	After	C57	P	Before	0.14167	2.042792	0.07	0.9450	-3.96462	4.2480
CNTNAP2	ChABC	After	CNTNAP2	ChABC	Before	-0.05749	1.804132	-0.03	0.9747	-3.68119	3.5662
C57	P	Before	CNTNAP2	P	Before	0.02500	2.220237	0.01	0.9910	-4.38278	4.4328

All Pairwise Differences Connecting Letters

Mouse	treatment	Time			Least Squares Mean
C57	ChABC	Before	A		18.782857
C57	P	After	A		17.389167
C57	P	Before	A		17.247500
CNTNAP2	P	Before	A		17.222500
CNTNAP2	ChABC	Before	A		16.928750
CNTNAP2	ChABC	After	A		16.871264
C57	ChABC	After	A		16.605000
CNTNAP2	P	After		B	11.768010

Levels not connected by same letter are significantly different.

S-FIG-33B

Fit Mixed Gender=M

Fixed Effects Tests

Source	Nparm	DFNum	DFDen	F Ratio	Prob > F
Mouse	1	1	23.8	1.0275883	0.3209
treatment	1	1	23.8	2.2528817	0.1465
Mouse*treatment	1	1	23.8	0.912527	0.3491
Time	1	1	24.3	0.1729091	0.6812
Mouse*Time	1	1	24.3	0.5779579	0.4544
treatment*Time	1	1	24.3	0.2783969	0.6025
Mouse*treatment*Time	1	1	24.3	3.7303985	0.0652

Multiple Comparisons for Mouse*treatment*Time

Student's t All Pairwise Comparisons

All Pairwise Differences

Mouse	treatment	Time	-Mouse	-treatment	-Time	Difference	Std Error	t Ratio	Prob>|t|	Lower 95%	Upper 95%
C57	P	After	CNTNAP2	P	After	6.62555	3.252357	2.04	0.0475*	0.0755	13.17564
C57	P	After	CNTNAP2	ChABC	Before	5.47500	2.714601	2.02	0.0499*	0.0029	10.94715
C57	ChABC	After	C57	P	After	-5.22875	2.714601	-1.93	0.0606	-10.7009	0.24340
CNTNAP2	ChABC	Before	CNTNAP2	P	Before	-4.49833	2.714601	-1.66	0.1047	-9.9705	0.97381
CNTNAP2	P	After	CNTNAP2	P	Before	-5.64889	3.546734	-1.59	0.1225	-12.9158	1.61802
C57	ChABC	After	CNTNAP2	P	Before	-4.25208	2.714601	-1.57	0.1245	-9.7242	1.22006
C57	ChABC	Before	C57	P	After	-3.58250	2.714601	-1.32	0.1938	-9.0546	1.88965
C57	P	After	CNTNAP2	ChABC	After	3.48125	2.714601	1.28	0.2065	-1.9909	8.95340
C57	P	Before	CNTNAP2	P	After	3.90889	3.252357	1.20	0.2357	-2.6412	10.45897
CNTNAP2	ChABC	After	CNTNAP2	P	After	3.14430	3.086277	1.02	0.3137	-3.0708	9.35944
C57	P	Before	CNTNAP2	ChABC	Before	2.75833	2.714601	1.02	0.3152	-2.7138	8.23048
C57	ChABC	Before	CNTNAP2	P	After	3.04305	3.086277	0.99	0.3294	-3.1721	9.25819
C57	ChABC	Before	CNTNAP2	P	Before	-2.60583	2.714601	-0.96	0.3424	-8.0780	2.86631
C57	ChABC	After	C57	P	Before	-2.51208	2.714601	-0.93	0.3598	-7.9842	2.96006
CNTNAP2	ChABC	After	CNTNAP2	P	Before	-2.50458	2.714601	-0.92	0.3613	-7.9767	2.96756
C57	P	After	C57	P	Before	2.71667	3.228510	0.84	0.4088	-3.9655	9.39880
C57	ChABC	Before	CNTNAP2	ChABC	Before	1.89250	2.513232	0.75	0.4555	-3.1737	6.95872
CNTNAP2	ChABC	After	CNTNAP2	ChABC	Before	1.99375	2.795972	0.71	0.4830	-3.7932	7.78065
C57	ChABC	After	CNTNAP2	ChABC	After	-1.74750	2.513232	-0.70	0.4905	-6.8137	3.31872
C57	P	Before	CNTNAP2	P	Before	-1.74000	2.902031	-0.60	0.5519	-7.5900	4.10997
C57	ChABC	After	C57	ChABC	Before	-1.64625	2.795972	-0.59	0.5618	-7.4332	4.14065

[illegible]	[illegible]	[illegible]	[illegible]	[illegible]	[illegible]	[illegible]	[illegible]	[illegible]	[illegible]	[illegible]	[illegible]
C57	ChABC	After	CNTNAP2	P	After	1.39680	3.086277	0.45	0.6530	-4.8183	7.61194
CNTNAP2	ChABC	Before	CNTNAP2	P	After	1.15055	3.086277	0.37	0.7110	-5.0646	7.36569
C57	P	After	CNTNAP2	P	Before	0.97667	2.902031	0.34	0.7381	-4.8733	6.82664
C57	ChABC	Before	C57	P	Before	-0.86583	2.714601	-0.32	0.7513	-6.3380	4.60631
C57	P	Before	CNTNAP2	ChABC	After	0.76458	2.714601	0.28	0.7795	-4.7076	6.23673
C57	ChABC	After	CNTNAP2	ChABC	Before	0.24625	2.513232	0.10	0.9224	-4.8200	5.31247
C57	ChABC	Before	CNTNAP2	ChABC	After	-0.10125	2.513232	-0.04	0.9680	-5.1675	4.96497

All Pairwise Differences Connecting Letters

Mouse	treatment	Time			Least Squares Mean
C57	P	After	A		19.195000
CNTNAP2	P	Before	A	B	18.218333
C57	P	Before	A	B	16.478333
CNTNAP2	ChABC	After	A	B	15.713750
C57	ChABC	Before	A	B	15.612500
C57	ChABC	After	A	B	13.966250
CNTNAP2	ChABC	Before		B	13.720000
CNTNAP2	P	After		B	12.569446

Levels not connected by same letter are significantly different.

S-FIG-33C

Fit Mixed Gender=F

Fixed Effects Tests

Source	Nparm	DFNum	DFDen	F Ratio	Prob > F
Mouse	1	1	25.0	2.9708646	0.0971
treatment	1	1	25.0	10.187097	0.0038*
Mouse*treatment	1	1	25.0	0.041909	0.8395
Time	1	1	24.3	5.7347304	0.0247*
Mouse*Time	1	1	24.3	0.1018799	0.7523
treatment*Time	1	1	24.3	0.2424408	0.6269
Mouse*treatment*Time	1	1	24.3	0.4478904	0.5097

Multiple Comparisons for Mouse*treatment*Time

Least Squares Means Estimates

Mouse	treatment	Time	Estimate	Std Error	DF	Lower 95%	Upper 95%
C57	ChABC	After	20.123333	2.0974376	41.261	15.888284	24.358382
C57	ChABC	Before	23.010000	2.0974376	41.261	18.774951	27.245049
C57	P	After	15.583333	2.0974376	41.261	11.348284	19.818382
C57	P	Before	18.016667	2.0974376	41.261	13.781618	22.251716
CNTNAP2	ChABC	After	18.148101	1.9439340	42.047	14.225214	22.070988
CNTNAP2	ChABC	Before	20.137500	1.8164343	41.261	16.469840	23.805160
CNTNAP2	P	After	11.239325	2.1020434	42.522	6.998772	15.479879
CNTNAP2	P	Before	16.206193	2.1020434	42.522	11.965639	20.446747

Student's t All Pairwise Comparisons

All Pairwise Differences

Mouse	treatment	Time	-Mouse	-treatment	-Time	Difference	Std Error	t Ratio	Prob>|t|	Lower 95%	Upper 95%
C57	ChABC	Before	CNTNAP2	P	After	11.7707	2.969483	3.96	0.0003*	5.7779	17.76343
CNTNAP2	ChABC	Before	CNTNAP2	P	After	8.8982	2.778132	3.20	0.0026*	3.2919	14.50441
C57	ChABC	After	CNTNAP2	P	After	8.8840	2.969483	2.99	0.0046*	2.8913	14.87676
C57	ChABC	Before	C57	P	After	7.4267	2.966225	2.50	0.0163*	1.4374	13.41593
CNTNAP2	ChABC	After	CNTNAP2	P	After	6.9088	2.863122	2.41	0.0202*	1.1321	12.68549
C57	ChABC	Before	CNTNAP2	P	Before	6.8038	2.969483	2.29	0.0270*	0.8111	12.79656
C57	P	Before	CNTNAP2	P	After	6.7773	2.969483	2.28	0.0276*	0.7846	12.77009
CNTNAP2	P	After	CNTNAP2	P	Before	-4.9669	2.729062	-1.82	0.0787	-10.5398	0.60610
C57	ChABC	Before	CNTNAP2	ChABC	After	4.8619	2.859742	1.70	0.0966	-0.9107	10.63453
[illegible]	[illegible]	[illegible]	[illegible]	[illegible]	[illegible]	[illegible]	[illegible]	[illegible]	[illegible]	[illegible]	[illegible]

C57	ChABC	Before	C57	P	Before	4.9933	2.966225	1.68	0.0999	-0.9959	10.98260
C57	P	After	CNTNAP2	ChABC	Before	-4.5542	2.774649	-1.64	0.1083	-10.1566	1.04828
C57	ChABC	After	C57	P	After	4.5400	2.966225	1.53	0.1335	-1.4493	10.52926
C57	P	After	CNTNAP2	P	After	4.3440	2.969483	1.46	0.1509	-1.6487	10.33676
CNTNAP2	ChABC	Before	CNTNAP2	P	Before	3.9313	2.778132	1.42	0.1644	-1.6749	9.53754
C57	ChABC	After	CNTNAP2	P	Before	3.9171	2.969483	1.32	0.1943	-2.0756	9.90989
C57	ChABC	After	C57	ChABC	Before	-2.8867	2.583333	-1.12	0.2759	-8.2459	2.47254
C57	ChABC	Before	CNTNAP2	ChABC	Before	2.8725	2.774649	1.04	0.3066	-2.7299	8.47494
C57	P	After	C57	P	Before	-2.4333	2.583333	-0.94	0.3565	-7.7925	2.92587
C57	P	After	CNTNAP2	ChABC	After	-2.5648	2.859742	-0.90	0.3750	-8.3374	3.20787
CNTNAP2	ChABC	After	CNTNAP2	ChABC	Before	-1.9894	2.341934	-0.85	0.4043	-6.8316	2.85283
C57	P	Before	CNTNAP2	ChABC	Before	-2.1208	2.774649	-0.76	0.4490	-7.7233	3.48161
C57	ChABC	After	C57	P	Before	2.1067	2.966225	0.71	0.4816	-3.8826	8.09593
C57	ChABC	After	CNTNAP2	ChABC	After	1.9752	2.859742	0.69	0.4936	-3.7974	7.74787
CNTNAP2	ChABC	After	CNTNAP2	P	Before	1.9419	2.863122	0.68	0.5013	-3.8348	7.71862
C57	P	Before	CNTNAP2	P	Before	1.8105	2.969483	0.61	0.5454	-4.1823	7.80323
C57	P	After	CNTNAP2	P	Before	-0.6229	2.969483	-0.21	0.8349	-6.6156	5.36989
C57	P	Before	CNTNAP2	ChABC	After	-0.1314	2.859742	-0.05	0.9636	-5.9041	5.64120
C57	ChABC	After	CNTNAP2	ChABC	Before	-0.0142	2.774649	-0.01	0.9960	-5.6166	5.58828

All Pairwise Differences Connecting Letters

Mouse	treatment	Time				Least Squares Mean
C57	ChABC	Before	A			23.010000
CNTNAP2	ChABC	Before	A	B		20.137500
C57	ChABC	After	A	B		20.123333
CNTNAP2	ChABC	After	A	B		18.148101
C57	P	Before	A	B		18.016667
CNTNAP2	P	Before		B	C	16.206193
C57	P	After		B	C	15.583333
CNTNAP2	P	After			C	11.239325

Levels not connected by same letter are significantly different.

S-FIG-34A

Fit Mixed

Fixed Effects Tests

Source	Nparm	DFNum	DFDen	F Ratio	Prob > F
Mouse	1	1	50.5	0.3857308	0.5373
treatment	1	1	50.5	2.0134894	0.1621
Mouse*treatment	1	1	50.5	0.8918345	0.3495
Time	1	1	49.1	0.0719995	0.7896
Mouse*Time	1	1	49.1	0.9513438	0.3342
treatment*Time	1	1	49.1	4.0639972	0.0493*
Mouse*treatment*Time	1	1	49.1	0.5524378	0.4609

Multiple Comparisons for Mouse*treatment*Time

Least Squares Means Estimates

Mouse	treatment	Time	Estimate	Std Error	DF	Lower 95%	Upper 95%
C57	ChABC	After	18.214286	1.5980123	81.656	15.035129	21.393442
C57	ChABC	Before	18.714286	1.5980123	81.656	15.535129	21.893442
C57	P	After	22.666667	1.7260506	81.656	19.232785	26.100548
C57	P	Before	20.916667	1.7260506	81.656	17.482785	24.350548
CNTNAP2	ChABC	After	18.899081	1.5354419	84.298	15.845844	21.952318
CNTNAP2	ChABC	Before	22.437500	1.4948037	81.656	19.463672	25.411328
CNTNAP2	P	After	22.006318	1.8632838	88.493	18.303720	25.708915
CNTNAP2	P	Before	20.666667	1.7260506	81.656	17.232785	24.100548

Student's t All Pairwise Comparisons

All Pairwise Differences

Mouse	treatment	Time	-Mouse	-treatment	-Time	Difference	Std Error	t Ratio	Prob>|t|	Lower 95%	Upper 95%
CNTNAP2	ChABC	After	CNTNAP2	ChABC	Before	-3.53842	1.610781	-2.20	0.0328*	-6.77536	-0.30148
C57	ChABC	After	CNTNAP2	ChABC	Before	-4.22321	2.188168	-1.93	0.0571	-8.57645	0.13002
C57	ChABC	After	C57	P	After	-4.45238	2.352210	-1.89	0.0619	-9.13197	0.22721
C57	ChABC	Before	CNTNAP2	ChABC	Before	-3.72321	2.188168	-1.70	0.0927	-8.07645	0.63002
C57	ChABC	Before	C57	P	After	-3.95238	2.352210	-1.68	0.0967	-8.63197	0.72721
C57	P	After	CNTNAP2	ChABC	After	3.76759	2.310159	1.63	0.1067	-0.82736	8.36253
C57	ChABC	After	CNTNAP2	P	After	-3.79203	2.454683	-1.54	0.1261	-8.67200	1.08793
C57	ChABC	Before	CNTNAP2	P	After	-3.29203	2.454683	-1.34	0.1834	-8.17200	1.58793
CNTNAP2	ChABC	After	CNTNAP2	P	After	-3.10724	2.414417	-1.29	0.2015	-7.90626	1.69179
C57	ChABC	After	C57	P	Before	-2.70238	2.352210	-1.15	0.2540	-7.38197	1.97721

C57	ChABC	After	CNTNAP2	P	Before	-2.45238	2.352210	-1.04	0.3002	-7.13197	2.22721
C57	P	After	C57	P	Before	1.75000	1.815295	0.96	0.3399	-1.90030	5.40030
C57	ChABC	Before	C57	P	Before	-2.20238	2.352210	-0.94	0.3519	-6.88197	2.47721
C57	P	Before	CNTNAP2	ChABC	After	2.01759	2.310159	0.87	0.3850	-2.57736	6.61253
C57	ChABC	Before	CNTNAP2	P	Before	-1.95238	2.352210	-0.83	0.4089	-6.63197	2.72721
C57	P	After	CNTNAP2	P	Before	2.00000	2.441004	0.82	0.4150	-2.85624	6.85624
CNTNAP2	ChABC	Before	CNTNAP2	P	Before	1.77083	2.283350	0.78	0.4403	-2.77176	6.31343
CNTNAP2	ChABC	After	CNTNAP2	P	Before	-1.76759	2.310159	-0.77	0.4464	-6.36253	2.82736
CNTNAP2	P	After	CNTNAP2	P	Before	1.33965	1.946245	0.69	0.4944	-2.56712	5.24643
C57	P	Before	CNTNAP2	ChABC	Before	-1.52083	2.283350	-0.67	0.5073	-6.06343	3.02176
C57	P	Before	CNTNAP2	P	After	-1.08965	2.539897	-0.43	0.6690	-6.13924	3.95994
C57	ChABC	After	CNTNAP2	ChABC	After	-0.68479	2.216128	-0.31	0.7581	-5.09263	3.72304
C57	ChABC	After	C57	ChABC	Before	-0.50000	1.680636	-0.30	0.7674	-3.87952	2.87952
C57	P	After	CNTNAP2	P	After	0.66035	2.539897	0.26	0.7955	-4.38924	5.70994
CNTNAP2	ChABC	Before	CNTNAP2	P	After	0.43118	2.388779	0.18	0.8572	-4.31759	5.17996
C57	P	Before	CNTNAP2	P	Before	0.25000	2.441004	0.10	0.9187	-4.60624	5.10624
C57	P	After	CNTNAP2	ChABC	Before	0.22917	2.283350	0.10	0.9203	-4.31343	4.77176
C57	ChABC	Before	CNTNAP2	ChABC	After	-0.18479	2.216128	-0.08	0.9337	-4.59263	4.22304

All Pairwise Differences Connecting Letters

Mouse	treatment	Time			Least Squares Mean
C57	P	After	A	B	22.666667
CNTNAP2	ChABC	Before	A		22.437500
CNTNAP2	P	After	A	B	22.006318
C57	P	Before	A	B	20.916667
CNTNAP2	P	Before	A	B	20.666667
CNTNAP2	ChABC	After		B	18.899081
C57	ChABC	Before	A	B	18.714286
C57	ChABC	After	A	B	18.214286

Levels not connected by same letter are significantly different.

S-FIG-34B

Fit Mixed Gender=M

Fixed Effects Tests

Source	Nparm	DFNum	DFDen	F Ratio	Prob > F
Mouse	1	1	24.2	0.0730405	0.7893
treatment	1	1	24.2	9.0789357	0.0060*
Mouse*treatment	1	1	24.2	0.4176008	0.5242
Time	1	1	23.2	0.1290473	0.7227
Mouse*Time	1	1	23.2	0.470092	0.4997
treatment*Time	1	1	23.2	2.9946186	0.0968
Mouse*treatment*Time	1	1	23.2	6.4677071	0.0181*

Multiple Comparisons for Mouse*treatment*Time

Student's t All Pairwise Comparisons

All Pairwise Differences

Mouse	treatment	Time	-Mouse	-treatment	-Time	Difference	Std Error	t Ratio	Prob>|t|	Lower 95%	Upper 95%
C57	P	Before	CNTNAP2	ChABC	After	8.58333	2.717717	3.16	0.0031*	3.0859	14.0808
CNTNAP2	ChABC	After	CNTNAP2	P	After	-8.53620	3.037279	-2.81	0.0074*	-14.6610	-2.4114
C57	ChABC	After	C57	P	Before	-7.20833	2.717717	-2.65	0.0115*	-12.7058	-1.7109
C57	ChABC	Before	C57	P	Before	-6.95833	2.717717	-2.56	0.0145*	-12.4558	-1.4609
C57	P	After	CNTNAP2	ChABC	After	6.58333	2.717717	2.42	0.0202*	1.0859	12.0808
C57	ChABC	After	CNTNAP2	P	After	-7.16120	3.037279	-2.36	0.0230*	-13.2860	-1.0364
C57	ChABC	Before	CNTNAP2	P	After	-6.91120	3.037279	-2.28	0.0279*	-13.0360	-0.7864
CNTNAP2	ChABC	After	CNTNAP2	ChABC	Before	-4.25000	1.892237	-2.25	0.0351*	-8.1744	-0.3256
C57	ChABC	After	C57	P	After	-5.20833	2.717717	-1.92	0.0627	-10.7058	0.2891
CNTNAP2	P	After	CNTNAP2	P	Before	4.95287	2.571606	1.93	0.0653	-0.3370	10.2427
C57	ChABC	Before	C57	P	After	-4.95833	2.717717	-1.82	0.0758	-10.4558	0.5391
C57	P	Before	CNTNAP2	P	Before	5.00000	2.905361	1.72	0.0932	-0.8770	10.8770
C57	P	Before	CNTNAP2	ChABC	Before	4.33333	2.717717	1.59	0.1189	-1.1641	9.8308
CNTNAP2	ChABC	Before	CNTNAP2	P	After	-4.28620	3.037279	-1.41	0.1654	-10.4110	1.8386
CNTNAP2	ChABC	After	CNTNAP2	P	Before	-3.58333	2.717717	-1.32	0.1950	-9.0808	1.9141
C57	ChABC	After	CNTNAP2	ChABC	Before	-2.87500	2.516117	-1.14	0.2602	-7.9646	2.2146
C57	ChABC	Before	CNTNAP2	ChABC	Before	-2.62500	2.516117	-1.04	0.3033	-7.7146	2.4646
C57	P	After	CNTNAP2	P	Before	3.00000	2.905361	1.03	0.3082	-2.8770	8.8770
C57	P	After	C57	P	Before	-2.00000	2.184967	-0.92	0.3699	-6.5315	2.5315
C57	P	After	CNTNAP2	ChABC	Before	2.33333	2.717717	0.86	0.3958	-3.1641	7.8308
C57	ChABC	After	CNTNAP2	P	Before	-2.20833	2.717717	-0.81	0.4214	-7.7058	3.2891

C57	ChABC	Before	CNTNAP2	P	Before	-1.95833	2.717717	-0.72	0.4755	-7.4558	3.5391
C57	ChABC	Before	CNTNAP2	ChABC	After	1.62500	2.516117	0.65	0.5222	-3.4646	6.7146
C57	P	After	CNTNAP2	P	After	-1.95287	3.206275	-0.61	0.5457	-8.4201	4.5143
C57	ChABC	After	CNTNAP2	ChABC	After	1.37500	2.516117	0.55	0.5879	-3.7146	6.4646
CNTNAP2	ChABC	Before	CNTNAP2	P	Before	0.66667	2.717717	0.25	0.8075	-4.8308	6.1641
C57	ChABC	After	C57	ChABC	Before	-0.25000	1.892237	-0.13	0.8961	-4.1744	3.6744
C57	P	Before	CNTNAP2	P	After	0.04713	3.206275	0.01	0.9883	-6.4201	6.5143

All Pairwise Differences Connecting Letters

Mouse	treatment	Time				Least Squares Mean
C57	P	Before	A			24.333333
CNTNAP2	P	After	A			24.286202
C57	P	After	A	B		22.333333
CNTNAP2	ChABC	Before	A	B		20.000000
CNTNAP2	P	Before	A	B	C	19.333333
C57	ChABC	Before		B	C	17.375000
C57	ChABC	After		B	C	17.125000
CNTNAP2	ChABC	After			C	15.750000

Levels not connected by same letter are significantly different.

S-FIG-34C

Fit Mixed Gender=F

Fixed Effects Tests

Source	Nparm	DFNum	DFDen	F Ratio	Prob > F
Mouse	1	1	24.4	1.0981713	0.3050
treatment	1	1	24.4	0.2568525	0.6168
Mouse*treatment	1	1	24.4	0.3407937	0.5647
Time	1	1	22.7	0.0002723	0.9870
Mouse*Time	1	1	22.7	2.9251936	0.1008
treatment*Time	1	1	22.7	1.5779602	0.2218
Mouse*treatment*Time	1	1	22.7	1.1823792	0.2883

Multiple Comparisons for Mouse*treatment*Time

Student's t All Pairwise Comparisons

All Pairwise Differences

Mouse	treatment	Time	-Mouse	-treatment	-Time	Difference	Std Error	t Ratio	Prob>|t|	Lower 95%	Upper 95%
C57	ChABC	After	C57	ChABC	Before	-0.83333	2.680570	-0.31	0.7590	-6.4151	4.7484
C57	ChABC	After	C57	P	After	-3.33333	3.725577	-0.89	0.3768	-10.8860	4.2193
C57	ChABC	After	C57	P	Before	2.16667	3.725577	0.58	0.5644	-5.3860	9.7193
C57	ChABC	After	CNTNAP2	ChABC	After	-2.69151	3.571487	-0.75	0.4558	-9.9245	4.5415
C57	ChABC	After	CNTNAP2	ChABC	Before	-5.20833	3.484958	-1.49	0.1437	-12.2732	1.8565
C57	ChABC	After	CNTNAP2	P	After	-0.55808	3.688946	-0.15	0.8805	-8.0219	6.9058
C57	ChABC	After	CNTNAP2	P	Before	-2.61874	3.688946	-0.71	0.4820	-10.0826	4.8451
C57	ChABC	Before	C57	P	After	-2.50000	3.725577	-0.67	0.5064	-10.0527	5.0527
C57	ChABC	Before	C57	P	Before	3.00000	3.725577	0.81	0.4259	-4.5527	10.5527
C57	ChABC	Before	CNTNAP2	ChABC	After	-1.85818	3.571487	-0.52	0.6059	-9.0912	5.3748
C57	ChABC	Before	CNTNAP2	ChABC	Before	-4.37500	3.484958	-1.26	0.2173	-11.4399	2.6899
C57	ChABC	Before	CNTNAP2	P	After	0.27525	3.688946	0.07	0.9409	-7.1886	7.7391
C57	ChABC	Before	CNTNAP2	P	Before	-1.78541	3.688946	-0.48	0.6311	-9.2493	5.6784
C57	P	After	C57	P	Before	5.50000	2.680570	2.05	0.0531	-0.0817	11.0817
C57	P	After	CNTNAP2	ChABC	After	0.64182	3.571487	0.18	0.8583	-6.5912	7.8748
C57	P	After	CNTNAP2	ChABC	Before	-1.87500	3.484958	-0.54	0.5938	-8.9399	5.1899
C57	P	After	CNTNAP2	P	After	2.77525	3.688946	0.75	0.4564	-4.6886	10.2391
C57	P	After	CNTNAP2	P	Before	0.71459	3.688946	0.19	0.8474	-6.7493	8.1784
C57	P	Before	CNTNAP2	ChABC	After	-4.85818	3.571487	-1.36	0.1819	-12.0912	2.3748
C57	P	Before	CNTNAP2	ChABC	Before	-7.37500	3.484958	-2.12	0.0412*	-14.4399	-0.3101
C57	P	Before	CNTNAP2	P	After	-2.72475	3.688946	-0.74	0.4646	-10.1886	4.7391

C57	P	Before	CNTNAP2	P	After	[illegible]	[illegible]	[illegible]	[illegible]	[illegible]	[illegible]
C57	P	Before	CNTNAP2	P	Before	-4.78541	3.688946	-1.30	0.2022	-12.2493	2.6784
CNTNAP2	ChABC	After	CNTNAP2	ChABC	Before	-2.51682	2.449424	-1.03	0.3155	-7.6021	2.5684
CNTNAP2	ChABC	After	CNTNAP2	P	After	2.13343	3.533259	0.60	0.5494	-5.0087	9.2755
CNTNAP2	ChABC	After	CNTNAP2	P	Before	0.07277	3.533259	0.02	0.9837	-7.0693	7.2149
CNTNAP2	ChABC	Before	CNTNAP2	P	After	4.65025	3.445770	1.35	0.1850	-2.3198	11.6203
CNTNAP2	ChABC	Before	CNTNAP2	P	Before	2.58959	3.445770	0.75	0.4569	-4.3805	9.5597
CNTNAP2	P	After	CNTNAP2	P	Before	-2.06066	2.973655	-0.69	0.4941	-8.1547	4.0334

All Pairwise Differences Connecting Letters

Mouse	treatment	Time			Least Squares Mean
CNTNAP2	ChABC	Before	A		24.875000
C57	P	After	A	B	23.000000
CNTNAP2	ChABC	After	A	B	22.358175
CNTNAP2	P	Before	A	B	22.285409
C57	ChABC	Before	A	B	20.500000
CNTNAP2	P	After	A	B	20.224750
C57	ChABC	After	A	B	19.666667
C57	P	Before		B	17.500000

Levels not connected by same letter are significantly different.

S-FIG-35A

Fit Mixed

Fixed Effects Tests

Source	Nparm	DFNum	DFDen	F Ratio	Prob > F
Mouse	1	1	50.5	0.2325072	0.6318
treatment	1	1	50.5	2.0792213	0.1555
Mouse*treatment	1	1	50.5	0.9580709	0.3323
Time	1	1	49.1	0.0000354	0.9953
Mouse*Time	1	1	49.1	0.4927166	0.4860
treatment*Time	1	1	49.1	4.444224	0.0401*
Mouse*treatment*Time	1	1	49.1	0.294836	0.5896

Multiple Comparisons for Mouse*treatment*Time

Student's t All Pairwise Comparisons

All Pairwise Differences

Mouse	treatment	Time	-Mouse	-treatment	-Time	Difference	Std Error	t Ratio	Prob>|t|	Lower 95%	Upper 95%
C57	ChABC	After	C57	P	After	-4.98810	2.448864	-2.04	0.0449*	-9.86019	-0.11600
CNTNAP2	ChABC	After	CNTNAP2	ChABC	Before	-3.06777	1.670142	-1.84	0.0723	-6.42395	0.28841
C57	ChABC	After	CNTNAP2	ChABC	Before	-4.07143	2.278082	-1.79	0.0776	-8.60375	0.46089
C57	ChABC	Before	C57	P	After	-4.20238	2.448864	-1.72	0.0900	-9.07448	0.66972
C57	P	After	CNTNAP2	ChABC	After	3.98444	2.404936	1.66	0.1014	-0.79922	8.76810
C57	ChABC	After	CNTNAP2	P	After	-4.11339	2.554989	-1.61	0.1111	-9.19294	0.96616
C57	ChABC	Before	CNTNAP2	ChABC	Before	-3.28571	2.278082	-1.44	0.1530	-7.81803	1.24661
C57	ChABC	Before	CNTNAP2	P	After	-3.32768	2.554989	-1.30	0.1963	-8.40723	1.75187
CNTNAP2	ChABC	After	CNTNAP2	P	After	-3.10974	2.512917	-1.24	0.2192	-8.10470	1.88523
C57	ChABC	After	C57	P	Before	-2.90476	2.448864	-1.19	0.2390	-7.77686	1.96734
C57	P	After	C57	P	Before	2.08333	1.882061	1.11	0.2739	-1.70116	5.86782
C57	P	After	CNTNAP2	P	Before	2.66667	2.541306	1.05	0.2971	-2.38935	7.72268
C57	ChABC	After	CNTNAP2	P	Before	-2.32143	2.448864	-0.95	0.3460	-7.19353	2.55067
CNTNAP2	P	After	CNTNAP2	P	Before	1.79196	2.018214	0.89	0.3787	-2.25925	5.84318
C57	ChABC	Before	C57	P	Before	-2.11905	2.448864	-0.87	0.3894	-6.99114	2.75305
C57	P	Before	CNTNAP2	ChABC	After	1.90111	2.404936	0.79	0.4315	-2.88255	6.68477
CNTNAP2	ChABC	Before	CNTNAP2	P	Before	1.75000	2.377175	0.74	0.4637	-2.97947	6.47947
C57	ChABC	Before	CNTNAP2	P	Before	-1.53571	2.448864	-0.63	0.5323	-6.40781	3.33638
CNTNAP2	ChABC	After	CNTNAP2	P	Before	-1.31777	2.404936	-0.55	0.5852	-6.10143	3.46589
C57	P	Before	CNTNAP2	ChABC	Before	-1.16667	2.377175	-0.49	0.6249	-5.89613	3.56280
C57	P	Before	CNTNAP2	P	After	-1.20863	2.643723	-0.46	0.6487	-6.46482	4.04756

C57	P	Before	CNTNAP2	P	After	-1.20865	2.643723	-0.46	0.6487	-6.46482	4.04750
C57	ChABC	After	C57	ChABC	Before	-0.78571	1.742450	-0.45	0.6541	-4.28947	2.71804
C57	ChABC	After	CNTNAP2	ChABC	After	-1.00366	2.307036	-0.44	0.6647	-5.59250	3.58519
C57	P	After	CNTNAP2	ChABC	Before	0.91667	2.377175	0.39	0.7008	-3.81280	5.64613
C57	P	After	CNTNAP2	P	After	0.87470	2.643723	0.33	0.7416	-4.38149	6.13089
C57	P	Before	CNTNAP2	P	Before	0.58333	2.541306	0.23	0.8190	-4.47268	5.63935
C57	ChABC	Before	CNTNAP2	ChABC	After	-0.21794	2.307036	-0.09	0.9250	-4.80678	4.37090
CNTNAP2	ChABC	Before	CNTNAP2	P	After	-0.04196	2.486361	-0.02	0.9866	-4.98489	4.90097

All Pairwise Differences Connecting Letters

Mouse	treatment	Time			Least Squares Mean
C57	P	After	A		23.416667
CNTNAP2	P	After	A	B	22.541965
CNTNAP2	ChABC	Before	A	B	22.500000
C57	P	Before	A	B	21.333333
CNTNAP2	P	Before	A	B	20.750000
CNTNAP2	ChABC	After	A	B	19.432228
C57	ChABC	Before	A	B	19.214286
C57	ChABC	After		B	18.428571

Levels not connected by same letter are significantly different.

S-FIG-35B

Fit Mixed Gender=M

Fixed Effects Tests

Source	Nparm	DFNum	DFDen	F Ratio	Prob > F
Mouse	1	1	24.2	0.1102483	0.7427
treatment	1	1	24.2	9.3720381	0.0053*
Mouse*treatment	1	1	24.2	0.3098044	0.5829
Time	1	1	23.3	0.0022703	0.9624
Mouse*Time	1	1	23.3	0.6251696	0.4371
treatment*Time	1	1	23.3	3.2806084	0.0830
Mouse*treatment*Time	1	1	23.3	5.9091568	0.0231*

Multiple Comparisons for Mouse*treatment*Time

Least Squares Means Estimates

Mouse	treatment	Time	Estimate	Std Error	DF	Lower 95%	Upper 95%
C57	ChABC	After	17.500000	1.8150464	40.249	13.832360	21.167640
C57	ChABC	Before	17.750000	1.8150464	40.249	14.082360	21.417640
C57	P	After	22.833333	2.0958351	40.249	18.598307	27.068360
C57	P	Before	24.500000	2.0958351	40.249	20.264974	28.735026
CNTNAP2	ChABC	After	16.000000	1.8150464	40.249	12.332360	19.667640
CNTNAP2	ChABC	Before	20.000000	1.8150464	40.249	16.332360	23.667640
CNTNAP2	P	After	25.032127	2.5313653	45.091	19.933979	30.130276
CNTNAP2	P	Before	19.333333	2.0958351	40.249	15.098307	23.568360

Student's t All Pairwise Comparisons

All Pairwise Differences

Mouse	treatment	Time	-Mouse	-treatment	-Time	Difference	Std Error	t Ratio	Prob>|t|	Lower 95%	Upper 95%
C57	P	Before	CNTNAP2	ChABC	After	8.50000	2.772529	3.07	0.0039*	2.8976	14.1024
CNTNAP2	ChABC	After	CNTNAP2	P	After	-9.03213	3.114836	-2.90	0.0058*	-15.3104	-2.7538
C57	ChABC	After	C57	P	Before	-7.00000	2.772529	-2.52	0.0156*	-12.6024	-1.3976
C57	P	After	CNTNAP2	ChABC	After	6.83333	2.772529	2.46	0.0181*	1.2309	12.4357
C57	ChABC	Before	C57	P	Before	-6.75000	2.772529	-2.43	0.0194*	-12.3524	-1.1476
C57	ChABC	After	CNTNAP2	P	After	-7.53213	3.114836	-2.42	0.0198*	-13.8104	-1.2538
C57	ChABC	Before	CNTNAP2	P	After	-7.28213	3.114836	-2.34	0.0240*	-13.5604	-1.0038
CNTNAP2	P	After	CNTNAP2	P	Before	5.69879	2.722763	2.09	0.0463*	0.1012	11.2964
CNTNAP2	ChABC	After	CNTNAP2	ChABC	Before	-4.00000	2.012117	-1.99	0.0594	-8.1723	0.1723
[illegible]	[illegible]	[illegible]	[illegible]	[illegible]	[illegible]	[illegible]	[illegible]	[illegible]	[illegible]	[illegible]	[illegible]

C57	ChABC	After	C57	P	After	-5.33333	2.772529	-1.92	0.0615	-10.9357	0.2691
C57	ChABC	Before	C57	P	After	-5.08333	2.772529	-1.83	0.0741	-10.6857	0.5191
C57	P	Before	CNTNAP2	P	Before	5.16667	2.963958	1.74	0.0889	-0.8226	11.1559
C57	P	Before	CNTNAP2	ChABC	Before	4.50000	2.772529	1.62	0.1124	-1.1024	10.1024
CNTNAP2	ChABC	Before	CNTNAP2	P	After	-5.03213	3.114836	-1.62	0.1134	-11.3104	1.2462
CNTNAP2	ChABC	After	CNTNAP2	P	Before	-3.33333	2.772529	-1.20	0.2363	-8.9357	2.2691
C57	P	After	CNTNAP2	P	Before	3.50000	2.963958	1.18	0.2446	-2.4892	9.4892
C57	P	After	CNTNAP2	ChABC	Before	2.83333	2.772529	1.02	0.3129	-2.7691	8.4357
C57	ChABC	After	CNTNAP2	ChABC	Before	-2.50000	2.566863	-0.97	0.3359	-7.6868	2.6868
C57	ChABC	Before	CNTNAP2	ChABC	Before	-2.25000	2.566863	-0.88	0.3859	-7.4368	2.9368
C57	P	After	C57	P	Before	-1.66667	2.323392	-0.72	0.4807	-6.4845	3.1511
C57	ChABC	Before	CNTNAP2	ChABC	After	1.75000	2.566863	0.68	0.4993	-3.4368	6.9368
C57	P	After	CNTNAP2	P	After	-2.19879	3.286386	-0.67	0.5070	-8.8242	4.4266
C57	ChABC	After	CNTNAP2	P	Before	-1.83333	2.772529	-0.66	0.5122	-7.4357	3.7691
C57	ChABC	After	CNTNAP2	ChABC	After	1.50000	2.566863	0.58	0.5622	-3.6868	6.6868
C57	ChABC	Before	CNTNAP2	P	Before	-1.58333	2.772529	-0.57	0.5711	-7.1857	4.0191
CNTNAP2	ChABC	Before	CNTNAP2	P	Before	0.66667	2.772529	0.24	0.8112	-4.9357	6.2691
C57	P	Before	CNTNAP2	P	After	-0.53213	3.286386	-0.16	0.8721	-7.1575	6.0933
C57	ChABC	After	C57	ChABC	Before	-0.25000	2.012117	-0.12	0.9022	-4.4223	3.9223

All Pairwise Differences Connecting Letters

Mouse	treatment	Time					Least Squares Mean
CNTNAP2	P	After	A				25.032127
C57	P	Before	A	B			24.500000
C57	P	After	A	B	C		22.833333
CNTNAP2	ChABC	Before	A	B	C	D	20.000000
CNTNAP2	P	Before		B	C	D	19.333333
C57	ChABC	Before			C	D	17.750000
C57	ChABC	After			C	D	17.500000
CNTNAP2	ChABC	After				D	16.000000

Levels not connected by same letter are significantly different.

S-FIG-35C

Fit Mixed Gender=F

Fixed Effects Tests

Source	Nparm	DFNum	DFDen	F Ratio	Prob > F
Mouse	1	1	24.5	0.7870785	0.3836
treatment	1	1	24.5	0.1782302	0.6766
Mouse*treatment	1	1	24.5	0.5045421	0.4842
Time	1	1	22.6	0.0167407	0.8982
Mouse*Time	1	1	22.6	2.1162772	0.1595
treatment*Time	1	1	22.6	1.7593861	0.1979
Mouse*treatment*Time	1	1	22.6	1.83444	0.1890

Multiple Comparisons for Mouse*treatment*Time

Least Squares Means Estimates

Mouse	treatment	Time	Estimate	Std Error	DF	Lower 95%	Upper 95%
C57	ChABC	After	19.666667	2.7794475	35.464	14.026727	25.306606
C57	ChABC	Before	21.166667	2.7794475	35.464	15.526727	26.806606
C57	P	After	24.000000	2.7794475	35.464	18.360061	29.639939
C57	P	Before	18.166667	2.7794475	35.464	12.526727	23.806606
CNTNAP2	ChABC	After	23.225556	2.5360673	37.976	18.091450	28.359662
CNTNAP2	ChABC	Before	25.000000	2.4070722	35.464	20.115669	29.884331
CNTNAP2	P	After	20.567029	2.7078109	40.053	15.094564	26.039494
CNTNAP2	P	Before	22.418056	2.7078109	40.053	16.945591	27.890522

Student's t All Pairwise Comparisons

All Pairwise Differences

Mouse	treatment	Time	-Mouse	-treatment	-Time	Difference	Std Error	t Ratio	Prob>|t|	Lower 95%	Upper 95%
C57	P	After	C57	P	Before	5.83333	2.705368	2.16	0.0431*	0.1997	11.46696
C57	P	Before	CNTNAP2	ChABC	Before	-6.83333	3.676863	-1.86	0.0714	-14.2943	0.62760
C57	ChABC	After	CNTNAP2	ChABC	Before	-5.33333	3.676863	-1.45	0.1557	-12.7943	2.12760
C57	P	Before	CNTNAP2	ChABC	After	-5.05889	3.762574	-1.34	0.1870	-12.6852	2.56740
CNTNAP2	ChABC	Before	CNTNAP2	P	After	4.43297	3.623015	1.22	0.2286	-2.9004	11.76629
C57	ChABC	After	C57	P	After	-4.33333	3.930732	-1.10	0.2777	-12.3094	3.64275
C57	P	Before	CNTNAP2	P	Before	-4.25139	3.880408	-1.10	0.2802	-12.1079	3.60517
C57	ChABC	Before	CNTNAP2	ChABC	Before	-3.83333	3.676863	-1.04	0.3042	-11.2943	3.62760
C57	ChABC	After	CNTNAP2	ChABC	After	-3.55889	3.762574	-0.95	0.3504	-11.1852	4.06740

C57	P	After	CNTNAP2	P	After	3.43297	3.880408	0.88	0.3819	-4.4236	11.28953
C57	ChABC	Before	C57	P	Before	3.00000	3.930732	0.76	0.4504	-4.9761	10.97608
C57	ChABC	Before	C57	P	After	-2.83333	3.930732	-0.72	0.4757	-10.8094	5.14275
CNTNAP2	ChABC	After	CNTNAP2	P	After	2.65853	3.709970	0.72	0.4779	-4.8448	10.16187
CNTNAP2	ChABC	Before	CNTNAP2	P	Before	2.58194	3.623015	0.71	0.4804	-4.7514	9.91527
CNTNAP2	ChABC	After	CNTNAP2	ChABC	Before	-1.77444	2.475258	-0.72	0.4812	-6.9145	3.36563
C57	ChABC	After	CNTNAP2	P	Before	-2.75139	3.880408	-0.71	0.4826	-10.6079	5.10517
C57	P	Before	CNTNAP2	P	After	-2.40036	3.880408	-0.62	0.5399	-10.2569	5.45620
CNTNAP2	P	After	CNTNAP2	P	Before	-1.85103	3.027572	-0.61	0.5460	-8.0595	4.35741
C57	ChABC	After	C57	ChABC	Before	-1.50000	2.705368	-0.55	0.5853	-7.1336	4.13363
C57	ChABC	Before	CNTNAP2	ChABC	After	-2.05889	3.762574	-0.55	0.5876	-9.6852	5.56740
C57	P	After	CNTNAP2	P	Before	1.58194	3.880408	0.41	0.6858	-6.2746	9.43850
C57	ChABC	After	C57	P	Before	1.50000	3.930732	0.38	0.7050	-6.4761	9.47608
C57	ChABC	Before	CNTNAP2	P	Before	-1.25139	3.880408	-0.32	0.7489	-9.1079	6.60517
C57	P	After	CNTNAP2	ChABC	Before	-1.00000	3.676863	-0.27	0.7872	-8.4609	6.46094
C57	ChABC	After	CNTNAP2	P	After	-0.90036	3.880408	-0.23	0.8178	-8.7569	6.95620
CNTNAP2	ChABC	After	CNTNAP2	P	Before	0.80750	3.709970	0.22	0.8288	-6.6958	8.31085
C57	P	After	CNTNAP2	ChABC	After	0.77444	3.762574	0.21	0.8381	-6.8518	8.40073
C57	ChABC	Before	CNTNAP2	P	After	0.59964	3.880408	0.15	0.8780	-7.2569	8.45620

All Pairwise Differences Connecting Letters

Mouse	treatment	Time			Least Squares Mean
CNTNAP2	ChABC	Before	A	B	25.000000
C57	P	After	A		24.000000
CNTNAP2	ChABC	After	A	B	23.225556
CNTNAP2	P	Before	A	B	22.418056
C57	ChABC	Before	A	B	21.166667
CNTNAP2	P	After	A	B	20.567029
C57	ChABC	After	A	B	19.666667
C57	P	Before		B	18.166667

Levels not connected by same letter are significantly different.

S-FIG-36A

Fit Mixed

Fixed Effects Tests

Source	Nparm	DFNum	DFDen	F Ratio	Prob > F
Mouse	1	1	43.6	15.790318	0.0003*
treatment	1	1	43.6	0.8953423	0.3493
Mouse*treatment	1	1	43.6	0.4453111	0.5081
Time	1	1	42.2	8.5502132	0.0055*
Mouse*Time	1	1	42.2	3.9291208	0.0540
treatment*Time	1	1	42.2	0.0109639	0.9171
Mouse*treatment*Time	1	1	42.2	0.0001561	0.9901

Multiple Comparisons for Mouse*treatment*Time

Student's t All Pairwise Comparisons

All Pairwise Differences

Mouse	treatment	Time	-Mouse	-treatment	-Time	Difference	Std Error	t Ratio	Prob>|t|	Lower 95%	Upper 95%
C57	ChABC	After	CNTNAP2	ChABC	Before	-3.42821	0.8309220	-4.13	<.0001*	-5.08251	-1.77390
C57	ChABC	After	CNTNAP2	P	Before	-3.61664	0.8932143	-4.05	0.0001*	-5.39497	-1.83832
C57	ChABC	Before	CNTNAP2	ChABC	Before	-3.14213	0.8309220	-3.78	0.0003*	-4.79644	-1.48783
C57	ChABC	Before	CNTNAP2	P	Before	-3.33057	0.8932143	-3.73	0.0004*	-5.10890	-1.55225
C57	P	After	CNTNAP2	P	Before	-2.78583	0.9269323	-3.01	0.0036*	-4.63129	-0.94038
C57	P	After	CNTNAP2	ChABC	Before	-2.59740	0.8670658	-3.00	0.0037*	-4.32366	-0.87113
C57	P	Before	CNTNAP2	P	Before	-2.43767	0.9269323	-2.63	0.0103*	-4.28312	-0.59221
C57	P	Before	CNTNAP2	ChABC	Before	-2.24923	0.8670658	-2.59	0.0113*	-3.97549	-0.52296
CNTNAP2	ChABC	After	CNTNAP2	ChABC	Before	-1.61243	0.6135182	-2.63	0.0119*	-2.85045	-0.37441
CNTNAP2	P	After	CNTNAP2	P	Before	-1.69135	0.7415533	-2.28	0.0274*	-3.18553	-0.19717
C57	ChABC	After	CNTNAP2	ChABC	After	-1.81577	0.8416900	-2.16	0.0340*	-3.49100	-0.14054
C57	ChABC	After	CNTNAP2	P	After	-1.92529	0.9326706	-2.06	0.0421*	-3.78042	-0.07016
CNTNAP2	ChABC	After	CNTNAP2	P	Before	-1.80087	0.8773903	-2.05	0.0434*	-3.54719	-0.05454
C57	ChABC	Before	CNTNAP2	ChABC	After	-1.52970	0.8416900	-1.82	0.0729	-3.20493	0.14553
C57	ChABC	Before	CNTNAP2	P	After	-1.63922	0.9326706	-1.76	0.0825	-3.49435	0.21591
CNTNAP2	ChABC	Before	CNTNAP2	P	After	1.50292	0.9076595	1.66	0.1015	-0.30238	3.30821
C57	ChABC	After	C57	P	Before	-1.17898	0.8932143	-1.32	0.1907	-2.95730	0.59935
C57	P	After	CNTNAP2	P	After	-1.09448	0.9650110	-1.13	0.2600	-3.01405	0.82509
C57	P	After	CNTNAP2	ChABC	After	-0.98497	0.8773903	-1.12	0.2650	-2.73129	0.76136
C57	ChABC	Before	C57	P	Before	-0.89290	0.8932143	-1.00	0.3206	-2.67123	0.88542
C57	ChABC	After	C57	P	After	-0.83081	0.8932143	-0.93	0.3552	-2.60913	0.94752

C57	P	Before	CNTNAP2	P	After	-0.74631	0.9650110	-0.77	0.4415	-2.66588	1.17325
C57	P	Before	CNTNAP2	ChABC	After	-0.63680	0.8773903	-0.73	0.4701	-2.38313	1.10953
C57	ChABC	Before	C57	P	After	-0.54474	0.8932143	-0.61	0.5437	-2.32306	1.23359
C57	P	After	C57	P	Before	-0.34817	0.6912732	-0.50	0.6172	-1.74431	1.04798
C57	ChABC	After	C57	ChABC	Before	-0.28607	0.6399946	-0.45	0.6572	-1.57865	1.00651
CNTNAP2	ChABC	Before	CNTNAP2	P	Before	-0.18844	0.8670658	-0.22	0.8285	-1.91470	1.53783
CNTNAP2	ChABC	After	CNTNAP2	P	After	-0.10952	0.9175273	-0.12	0.9053	-1.93408	1.71505

All Pairwise Differences Connecting Letters

Mouse	treatment	Time					Least Squares Mean
CNTNAP2	P	Before	A				13.351500
CNTNAP2	ChABC	Before	A	B			13.163063
CNTNAP2	P	After		B	C		11.660147
CNTNAP2	ChABC	After			C		11.550632
C57	P	Before			C	D	10.913833
C57	P	After			C	D	10.565667
C57	ChABC	Before			C	D	10.020929
C57	ChABC	After				D	9.734857

Levels not connected by same letter are significantly different.

S-FIG-36B

Fit Mixed

Fixed Effects Tests

Source	Nparm	DFNum	DFDen	F Ratio	Prob > F
Mouse	1	1	43.6	15.343911	0.0003*
treatment	1	1	43.6	0.9428412	0.3369
Mouse*treatment	1	1	43.6	0.4666114	0.4982
Time	1	1	42.2	8.4021208	0.0059*
Mouse*Time	1	1	42.2	3.5991013	0.0647
treatment*Time	1	1	42.2	0.0035036	0.9531
Mouse*treatment*Time	1	1	42.2	0.0027132	0.9587

Multiple Comparisons for Mouse*treatment*Time

Least Squares Means Estimates

Mouse	treatment	Time	Estimate	Std Error	DF	Lower 95%	Upper 95%
C57	ChABC	After	0.03242857	0.00202391	77.828	0.02839913	0.03645801
C57	ChABC	Before	0.03342857	0.00202391	77.828	0.02939913	0.03745801
C57	P	After	0.03525000	0.00218607	77.828	0.03089771	0.03960229
C57	P	Before	0.03650000	0.00218607	77.828	0.03214771	0.04085229
CNTNAP2	ChABC	After	0.03843338	0.00194541	81.011	0.03456263	0.04230413
CNTNAP2	ChABC	Before	0.04381250	0.00189320	77.828	0.04004330	0.04758170
CNTNAP2	P	After	0.03893825	0.00236234	86.169	0.03424221	0.04363430
CNTNAP2	P	Before	0.04433333	0.00218607	77.828	0.03998104	0.04868563

Student's t All Pairwise Comparisons

All Pairwise Differences

Mouse	treatment	Time	-Mouse	-treatment	-Time	Difference	Std Error	t Ratio	Prob>|t|	Lower 95%	Upper 95%
C57	ChABC	After	CNTNAP2	ChABC	Before	-0.011384	0.0027714	-4.11	<.0001*	-0.016901	-0.005866
C57	ChABC	After	CNTNAP2	P	Before	-0.011905	0.0029791	-4.00	0.0001*	-0.017836	-0.005974
C57	ChABC	Before	CNTNAP2	ChABC	Before	-0.010384	0.0027714	-3.75	0.0003*	-0.015901	-0.004866
C57	ChABC	Before	CNTNAP2	P	Before	-0.010905	0.0029791	-3.66	0.0005*	-0.016836	-0.004974
C57	P	After	CNTNAP2	ChABC	Before	-0.008563	0.0028919	-2.96	0.0041*	-0.014320	-0.002805
C57	P	After	CNTNAP2	P	Before	-0.009083	0.0030916	-2.94	0.0043*	-0.015238	-0.002928
CNTNAP2	ChABC	After	CNTNAP2	ChABC	Before	-0.005379	0.0020469	-2.63	0.0119*	-0.009510	-0.001249
C57	P	Before	CNTNAP2	P	Before	-0.007833	0.0030916	-2.53	0.0133*	-0.013988	-0.001678

C57	P	Before	CNTNAP2	ChABC	Before	-0.007313	0.0028919	-2.53	0.0135*	-0.013070	-0.001555
CNTNAP2	P	After	CNTNAP2	P	Before	-0.005395	0.0024740	-2.18	0.0346*	-0.010380	-0.000410
C57	ChABC	After	CNTNAP2	ChABC	After	-0.006005	0.0028073	-2.14	0.0355*	-0.011592	-0.000417
C57	ChABC	After	CNTNAP2	P	After	-0.006510	0.0031108	-2.09	0.0394*	-0.012697	-0.000322
CNTNAP2	ChABC	After	CNTNAP2	P	Before	-0.005900	0.0029264	-2.02	0.0472*	-0.011724	-0.000075
C57	ChABC	Before	CNTNAP2	ChABC	After	-0.005005	0.0028073	-1.78	0.0784	-0.010592	0.000583
C57	ChABC	Before	CNTNAP2	P	After	-0.005510	0.0031108	-1.77	0.0802	-0.011697	0.000678
CNTNAP2	ChABC	Before	CNTNAP2	P	After	0.004874	0.0030273	1.61	0.1112	-0.001147	0.010895
C57	ChABC	After	C57	P	Before	-0.004071	0.0029791	-1.37	0.1757	-0.010003	0.001860
C57	P	After	CNTNAP2	P	After	-0.003688	0.0032186	-1.15	0.2551	-0.010091	0.002714
C57	P	After	CNTNAP2	ChABC	After	-0.003183	0.0029264	-1.09	0.2800	-0.009008	0.002641
C57	ChABC	Before	C57	P	Before	-0.003071	0.0029791	-1.03	0.3057	-0.009003	0.002860
C57	ChABC	After	C57	P	After	-0.002821	0.0029791	-0.95	0.3465	-0.008753	0.003110
C57	P	Before	CNTNAP2	P	After	-0.002438	0.0032186	-0.76	0.4509	-0.008841	0.003964
C57	P	Before	CNTNAP2	ChABC	After	-0.001933	0.0029264	-0.66	0.5107	-0.007758	0.003891
C57	ChABC	Before	C57	P	After	-0.001821	0.0029791	-0.61	0.5427	-0.007753	0.004110
C57	P	After	C57	P	Before	-0.001250	0.0023063	-0.54	0.5908	-0.005908	0.003408
C57	ChABC	After	C57	ChABC	Before	-0.001000	0.0021352	-0.47	0.6420	-0.005312	0.003312
CNTNAP2	ChABC	Before	CNTNAP2	P	Before	-0.000521	0.0028919	-0.18	0.8575	-0.006278	0.005237
CNTNAP2	ChABC	After	CNTNAP2	P	After	-0.000505	0.0030603	-0.16	0.8694	-0.006590	0.005581

All Pairwise Differences Connecting Letters

Mouse	treatment	Time					Least Squares Mean
CNTNAP2	P	Before	A				0.04433333
CNTNAP2	ChABC	Before	A	B			0.04381250
CNTNAP2	P	After		B	C		0.03893825
CNTNAP2	ChABC	After			C		0.03843338
C57	P	Before			C	D	0.03650000
C57	P	After			C	D	0.03525000
C57	ChABC	Before			C	D	0.03342857
C57	ChABC	After				D	0.03242857

Levels not connected by same letter are significantly different.

All Pairwise Comparisons Scatterplot

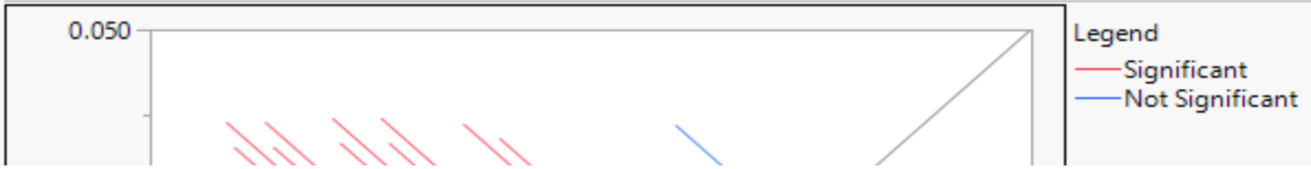

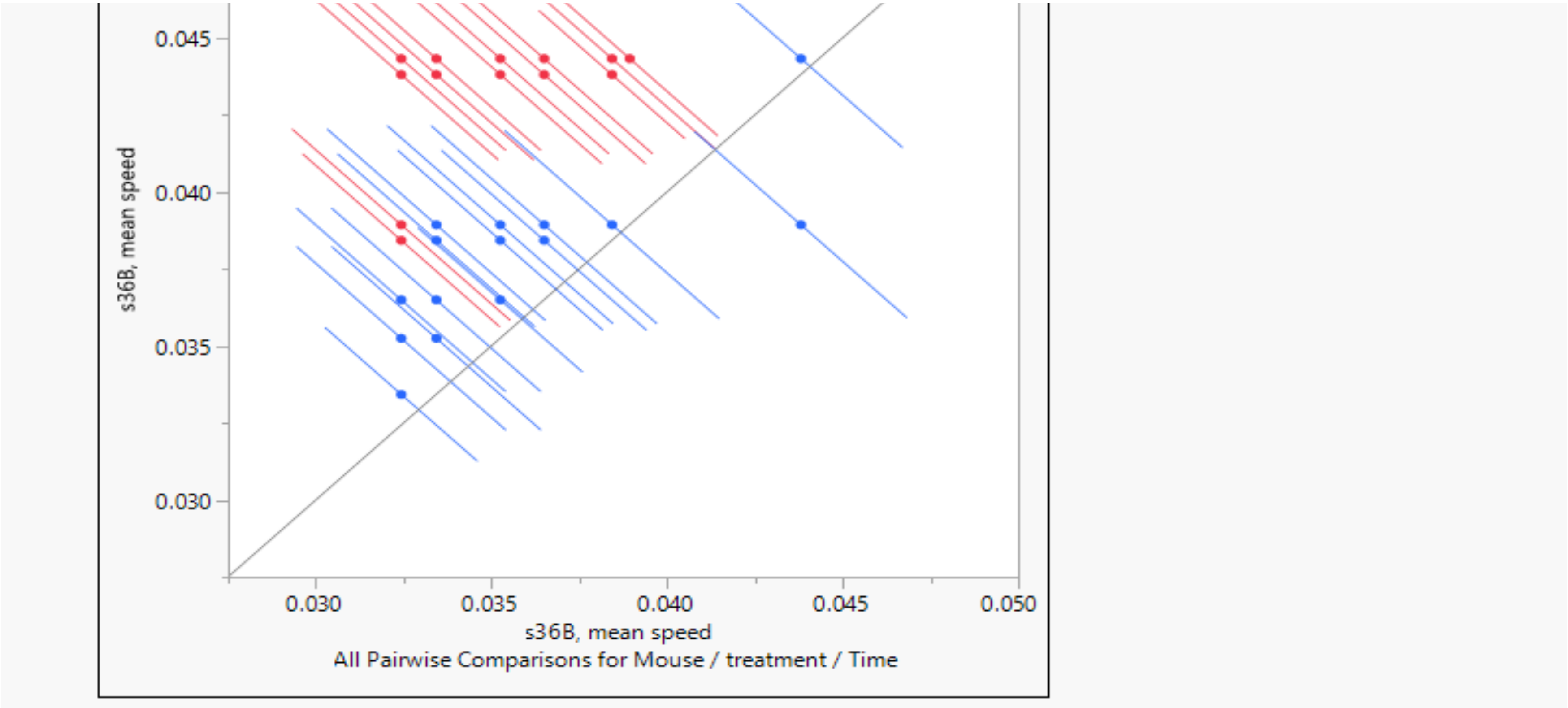
0.045
0.040
0.035
0.030
s36B, mean speed
0.030
0.035
0.040
0.045
0.050
s36B, mean speed
All Pairwise Comparisons for Mouse / treatment / Time

S-FIG-37A

Fit Mixed Gender=M

Fixed Effects Tests

Source	Nparm	DFNum	DFDen	F Ratio	Prob > F
Mouse	1	1	23.7	3.2536351	0.0840
treatment	1	1	23.7	6.9797127	0.0144*
Mouse*treatment	1	1	23.7	0.6942286	0.4131
Time	1	1	23.0	1.2234225	0.2801
Mouse*Time	1	1	23.0	2.7421885	0.1113
treatment*Time	1	1	23.0	0.476011	0.4971
Mouse*treatment*Time	1	1	23.0	0.229774	0.6362

Multiple Comparisons for Mouse*treatment*Time

Student's t All Pairwise Comparisons

All Pairwise Differences

Mouse	treatment	Time	-Mouse	-treatment	-Time	Difference	Std Error	t Ratio	Prob>|t|	Lower 95%	Upper 95%
C57	ChABC	After	CNTNAP2	P	Before	-3.36192	1.022140	-3.29	0.0020*	-5.42374	-1.30009
C57	ChABC	Before	CNTNAP2	P	Before	-3.08004	1.022140	-3.01	0.0043*	-5.14187	-1.01821
C57	ChABC	After	C57	P	After	-2.59608	1.022140	-2.54	0.0148*	-4.65791	-0.53426
C57	ChABC	After	CNTNAP2	ChABC	Before	-2.38350	0.946318	-2.52	0.0156*	-4.29238	-0.47462
CNTNAP2	ChABC	After	CNTNAP2	P	Before	-2.33117	1.022140	-2.28	0.0276*	-4.39299	-0.26934
C57	ChABC	Before	C57	P	After	-2.31421	1.022140	-2.26	0.0287*	-4.37604	-0.25238
C57	ChABC	Before	CNTNAP2	ChABC	Before	-2.10162	0.946318	-2.22	0.0317*	-4.01051	-0.19274
C57	ChABC	After	CNTNAP2	P	After	-2.20083	1.159491	-1.90	0.0641	-4.53634	0.13468
C57	ChABC	After	C57	P	Before	-1.81375	1.022140	-1.77	0.0831	-3.87558	0.24808
C57	ChABC	Before	CNTNAP2	P	After	-1.91896	1.159491	-1.65	0.1049	-4.25446	0.41655
CNTNAP2	ChABC	After	CNTNAP2	ChABC	Before	-1.35275	0.803619	-1.68	0.1067	-3.02117	0.31567
C57	P	After	CNTNAP2	ChABC	After	1.56533	1.022140	1.53	0.1330	-0.49649	3.62716
C57	ChABC	Before	C57	P	Before	-1.53187	1.022140	-1.50	0.1413	-3.59370	0.52995
C57	P	Before	CNTNAP2	P	Before	-1.54817	1.092713	-1.42	0.1638	-3.75235	0.65602
C57	ChABC	After	CNTNAP2	ChABC	After	-1.03075	0.946318	-1.09	0.2822	-2.93963	0.87813
CNTNAP2	P	After	CNTNAP2	P	Before	-1.16109	1.077368	-1.08	0.2911	-3.37591	1.05374
CNTNAP2	ChABC	After	CNTNAP2	P	After	-1.17008	1.159491	-1.01	0.3183	-3.50559	1.16543
CNTNAP2	ChABC	Before	CNTNAP2	P	Before	-0.97842	1.022140	-0.96	0.3438	-3.04024	1.08341
C57	P	After	C57	P	Before	0.78233	0.927940	0.84	0.4084	-1.14419	2.70885
C57	ChABC	Before	CNTNAP2	ChABC	After	-0.74888	0.946318	-0.79	0.4331	-2.65776	1.16001
C57	P	Before	CNTNAP2	ChABC	After	0.78300	1.022140	0.77	0.4479	-1.27883	2.84483

C57	P	Before	CNTNAP2	ChABC	After	[illegible]	1.022140	0.77	0.4479	[illegible]	[illegible]
C57	P	After	CNTNAP2	P	Before	-0.76583	1.092713	-0.70	0.4872	-2.97002	1.43835
C57	P	Before	CNTNAP2	ChABC	Before	-0.56975	1.022140	-0.56	0.5802	-2.63158	1.49208
C57	ChABC	After	C57	ChABC	Before	-0.28188	0.803619	-0.35	0.7292	-1.95029	1.38654
C57	P	After	CNTNAP2	P	After	0.39525	1.222159	0.32	0.7479	-2.06675	2.85726
C57	P	Before	CNTNAP2	P	After	-0.38708	1.222159	-0.32	0.7529	-2.84909	2.07493
C57	P	After	CNTNAP2	ChABC	Before	0.21258	1.022140	0.21	0.8362	-1.84924	2.27441
CNTNAP2	ChABC	Before	CNTNAP2	P	After	0.18267	1.159491	0.16	0.8755	-2.15284	2.51818

All Pairwise Differences Connecting Letters

Mouse	treatment	Time				Least Squares Mean
CNTNAP2	P	Before	A			12.478667
C57	P	After	A	B		11.712833
CNTNAP2	ChABC	Before	A	B		11.500250
CNTNAP2	P	After	A	B	C	11.317581
C57	P	Before	A	B	C	10.930500
CNTNAP2	ChABC	After		B	C	10.147500
C57	ChABC	Before			C	9.398625
C57	ChABC	After			C	9.116750

Levels not connected by same letter are significantly different.

S-FIG-37B

Fit Mixed Gender=F

Fixed Effects Tests

Source	Nparm	DFNum	DFDen	F Ratio	Prob > F
Mouse	1	1	18.5	14.314428	0.0013*
treatment	1	1	18.5	1.0565208	0.3173
Mouse*treatment	1	1	18.5	0.1168281	0.7364
Time	1	1	16.5	12.300374	0.0028*
Mouse*Time	1	1	16.5	2.4071629	0.1397
treatment*Time	1	1	16.5	1.8137131	0.1962
Mouse*treatment*Time	1	1	16.5	0.0012577	0.9721

Multiple Comparisons for Mouse*treatment*Time

Least Squares Means Estimates

Mouse	treatment	Time	Estimate	Std Error	DF	Lower 95%	Upper 95%
C57	ChABC	After	10.559000	0.94826826	30.251	8.623050	12.494950
C57	ChABC	Before	10.850667	0.94826826	30.251	8.914717	12.786616
C57	P	After	9.418500	0.94826826	30.251	7.482550	11.354450
C57	P	Before	10.897167	0.94826826	30.251	8.961217	12.833116
CNTNAP2	ChABC	After	13.161853	0.86472120	33.888	11.404314	14.919392
CNTNAP2	ChABC	Before	14.825875	0.82122440	30.251	13.149293	16.502457
CNTNAP2	P	After	11.444218	0.92338460	37.299	9.573769	13.314668
CNTNAP2	P	Before	14.359446	0.92338460	37.299	12.488996	16.229895

Student's t All Pairwise Comparisons

All Pairwise Differences

Mouse	treatment	Time	-Mouse	-treatment	-Time	Difference	Std Error	t Ratio	Prob>|t|	Lower 95%	Upper 95%
C57	P	After	CNTNAP2	ChABC	Before	-5.40738	1.254441	-4.31	0.0002*	-7.96840	-2.84635
C57	P	After	CNTNAP2	P	Before	-4.94095	1.323575	-3.73	0.0007*	-7.63162	-2.25027
C57	ChABC	After	CNTNAP2	ChABC	Before	-4.26687	1.254441	-3.40	0.0019*	-6.82790	-1.70585
C57	ChABC	Before	CNTNAP2	ChABC	Before	-3.97521	1.254441	-3.17	0.0035*	-6.53623	-1.41419
C57	P	Before	CNTNAP2	ChABC	Before	-3.92871	1.254441	-3.13	0.0038*	-6.48973	-1.36769
C57	P	After	CNTNAP2	ChABC	After	-3.74335	1.283338	-2.92	0.0064*	-6.35778	-1.12893
C57	ChABC	After	CNTNAP2	P	Before	-3.80045	1.323575	-2.87	0.0070*	-6.49112	-1.10977
CNTNAP2	P	After	CNTNAP2	P	Before	-2.91523	1.014113	-2.87	0.0091*	-5.02390	-0.80655
CNTNAP2	ChABC	Before	CNTNAP2	P	After	3.38166	1.235738	2.74	0.0098*	0.87094	5.89237

C57	ChABC	Before	CNTNAP2	P	Before	-3.50878	1.323575	-2.65	0.0121*	-6.19946	-0.81810
C57	P	Before	CNTNAP2	P	Before	-3.46228	1.323575	-2.62	0.0132*	-6.15296	-0.77160
C57	ChABC	After	CNTNAP2	ChABC	After	-2.60285	1.283338	-2.03	0.0510	-5.21728	0.01157
CNTNAP2	ChABC	After	CNTNAP2	ChABC	Before	-1.66402	0.816565	-2.04	0.0590	-3.39949	0.07145
C57	ChABC	Before	CNTNAP2	ChABC	After	-2.31119	1.283338	-1.80	0.0812	-4.92561	0.30324
C57	P	Before	CNTNAP2	ChABC	After	-2.26469	1.283338	-1.76	0.0872	-4.87911	0.34974
C57	P	After	C57	P	Before	-1.47867	0.889528	-1.66	0.1176	-3.37823	0.42089
C57	P	After	CNTNAP2	P	After	-2.02572	1.323575	-1.53	0.1352	-4.71640	0.66496
CNTNAP2	ChABC	After	CNTNAP2	P	After	1.71763	1.265062	1.36	0.1831	-0.84870	4.28397
C57	ChABC	Before	C57	P	After	1.43217	1.341054	1.07	0.2940	-1.30568	4.17001
CNTNAP2	ChABC	After	CNTNAP2	P	Before	-1.19759	1.265062	-0.95	0.3502	-3.76392	1.36874
C57	ChABC	After	C57	P	After	1.14050	1.341054	0.85	0.4018	-1.59735	3.87835
C57	ChABC	After	CNTNAP2	P	After	-0.88522	1.323575	-0.67	0.5082	-3.57590	1.80546
C57	ChABC	Before	CNTNAP2	P	After	-0.59355	1.323575	-0.45	0.6567	-3.28423	2.09713
C57	P	Before	CNTNAP2	P	After	-0.54705	1.323575	-0.41	0.6820	-3.23773	2.14363
CNTNAP2	ChABC	Before	CNTNAP2	P	Before	0.46643	1.235738	0.38	0.7082	-2.04429	2.97715
C57	ChABC	After	C57	ChABC	Before	-0.29167	0.889528	-0.33	0.7476	-2.19123	1.60789
C57	ChABC	After	C57	P	Before	-0.33817	1.341054	-0.25	0.8026	-3.07601	2.39968
C57	ChABC	Before	C57	P	Before	-0.04650	1.341054	-0.03	0.9726	-2.78435	2.69135

All Pairwise Differences Connecting Letters

Mouse	treatment	Time				Least Squares Mean
CNTNAP2	ChABC	Before	A			14.825875
CNTNAP2	P	Before	A			14.359446
CNTNAP2	ChABC	After	A	B		13.161853
CNTNAP2	P	After		B	C	11.444218
C57	P	Before		B	C	10.897167
C57	ChABC	Before		B	C	10.850667
C57	ChABC	After		B	C	10.559000
C57	P	After			C	9.418500

Levels not connected by same letter are significantly different.

S-FIG-37C

Fit Mixed Gender=M

Fixed Effects Tests

Source	Nparm	DFNum	DFDen	F Ratio	Prob > F
Mouse	1	1	23.7	3.0241527	0.0950
treatment	1	1	23.7	7.1160216	0.0135*
Mouse*treatment	1	1	23.7	0.7377485	0.3990
Time	1	1	23.1	1.1345302	0.2978
Mouse*Time	1	1	23.1	2.4171392	0.1336
treatment*Time	1	1	23.1	0.4749359	0.4976
Mouse*treatment*Time	1	1	23.1	0.2053707	0.6547

Multiple Comparisons for Mouse*treatment*Time

Student's t All Pairwise Comparisons

All Pairwise Differences

Mouse	treatment	Time	-Mouse	-treatment	-Time	Difference	Std Error	t Ratio	Prob>|t|	Lower 95%	Upper 95%
C57	ChABC	After	CNTNAP2	P	Before	-0.011125	0.0034381	-3.24	0.0023*	-0.018060	-0.004190
C57	ChABC	Before	CNTNAP2	P	Before	-0.010125	0.0034381	-2.94	0.0052*	-0.017060	-0.003190
C57	ChABC	After	C57	P	After	-0.008792	0.0034381	-2.56	0.0142*	-0.015727	-0.001857
C57	ChABC	After	CNTNAP2	ChABC	Before	-0.007875	0.0031830	-2.47	0.0174*	-0.014295	-0.001455
C57	ChABC	Before	C57	P	After	-0.007792	0.0034381	-2.27	0.0286*	-0.014727	-0.000857
CNTNAP2	ChABC	After	CNTNAP2	P	Before	-0.007625	0.0034381	-2.22	0.0319*	-0.014560	-0.000690
C57	ChABC	Before	CNTNAP2	ChABC	Before	-0.006875	0.0031830	-2.16	0.0364*	-0.013295	-0.000455
C57	ChABC	After	CNTNAP2	P	After	-0.007473	0.0039002	-1.92	0.0617	-0.015329	0.000383
C57	ChABC	After	C57	P	Before	-0.006292	0.0034381	-1.83	0.0742	-0.013227	0.000643
C57	ChABC	Before	CNTNAP2	P	After	-0.006473	0.0039002	-1.66	0.1040	-0.014329	0.001383
CNTNAP2	ChABC	After	CNTNAP2	ChABC	Before	-0.004375	0.0027055	-1.62	0.1203	-0.009991	0.001241
C57	ChABC	Before	C57	P	Before	-0.005292	0.0034381	-1.54	0.1311	-0.012227	0.001643
C57	P	After	CNTNAP2	ChABC	After	0.005292	0.0034381	1.54	0.1311	-0.001643	0.012227
C57	P	Before	CNTNAP2	P	Before	-0.004833	0.0036754	-1.32	0.1955	-0.012247	0.002580
C57	ChABC	After	CNTNAP2	ChABC	After	-0.003500	0.0031830	-1.10	0.2777	-0.009920	0.002920
CNTNAP2	ChABC	After	CNTNAP2	P	After	-0.003973	0.0039002	-1.02	0.3138	-0.011829	0.003883
CNTNAP2	P	After	CNTNAP2	P	Before	-0.003652	0.0036264	-1.01	0.3232	-0.011106	0.003802
CNTNAP2	ChABC	Before	CNTNAP2	P	Before	-0.003250	0.0034381	-0.95	0.3498	-0.010185	0.003685
C57	P	Before	CNTNAP2	ChABC	After	0.002792	0.0034381	0.81	0.4213	-0.004143	0.009727
[illegible]	[illegible]	[illegible]	[illegible]	[illegible]	[illegible]	[illegible]	[illegible]	[illegible]	[illegible]	[illegible]	[illegible]

C57	P	After	C57	P	Before	0.002500	0.0031240	0.80	0.4323	-0.003985	0.008985
C57	ChABC	Before	CNTNAP2	ChABC	After	-0.002500	0.0031830	-0.79	0.4365	-0.008920	0.003920
C57	P	After	CNTNAP2	P	Before	-0.002333	0.0036754	-0.63	0.5289	-0.009747	0.005080
C57	P	Before	CNTNAP2	ChABC	Before	-0.001583	0.0034381	-0.46	0.6475	-0.008518	0.005352
C57	ChABC	After	C57	ChABC	Before	-0.001000	0.0027055	-0.37	0.7153	-0.006616	0.004616
C57	P	After	CNTNAP2	P	After	0.001319	0.0041110	0.32	0.7499	-0.006963	0.009600
C57	P	Before	CNTNAP2	P	After	-0.001181	0.0041110	-0.29	0.7752	-0.009463	0.007100
C57	P	After	CNTNAP2	ChABC	Before	0.000917	0.0034381	0.27	0.7910	-0.006018	0.007852
CNTNAP2	ChABC	Before	CNTNAP2	P	After	0.000402	0.0039002	0.10	0.9184	-0.007454	0.008258

All Pairwise Differences Connecting Letters

Mouse	treatment	Time				Least Squares Mean
CNTNAP2	P	Before	A			0.04150000
C57	P	After	A	B		0.03916667
CNTNAP2	ChABC	Before	A	B		0.03825000
CNTNAP2	P	After	A	B	C	0.03784801
C57	P	Before	A	B	C	0.03666667
CNTNAP2	ChABC	After		B	C	0.03387500
C57	ChABC	Before			C	0.03137500
C57	ChABC	After			C	0.03037500

Levels not connected by same letter are significantly different.

All Pairwise Comparisons Scatterplot

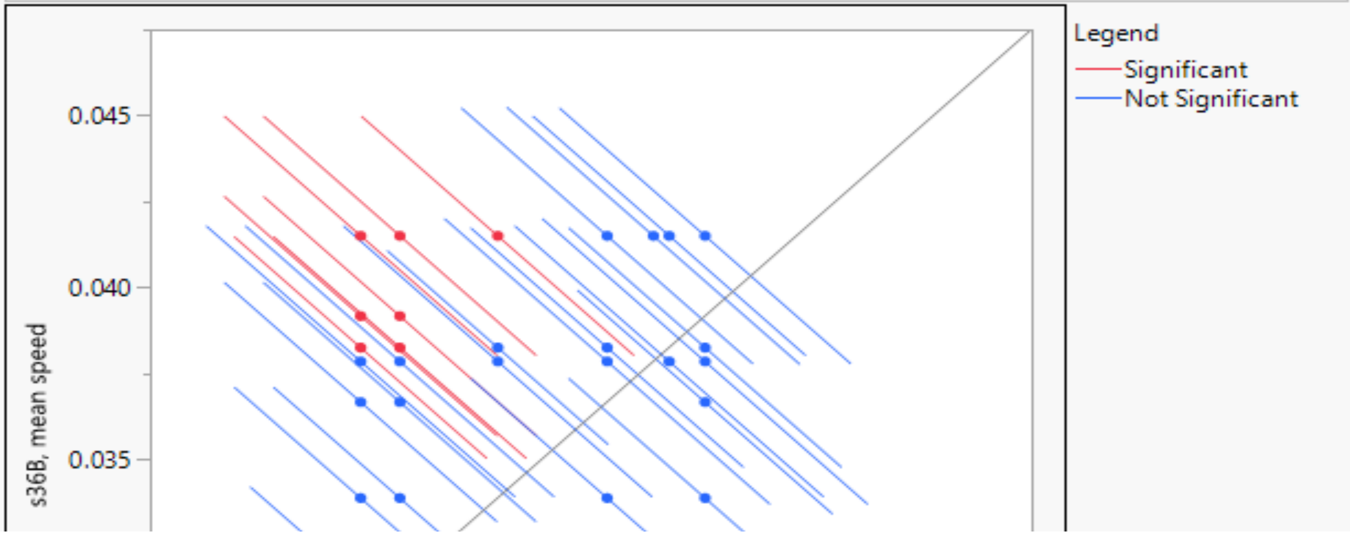

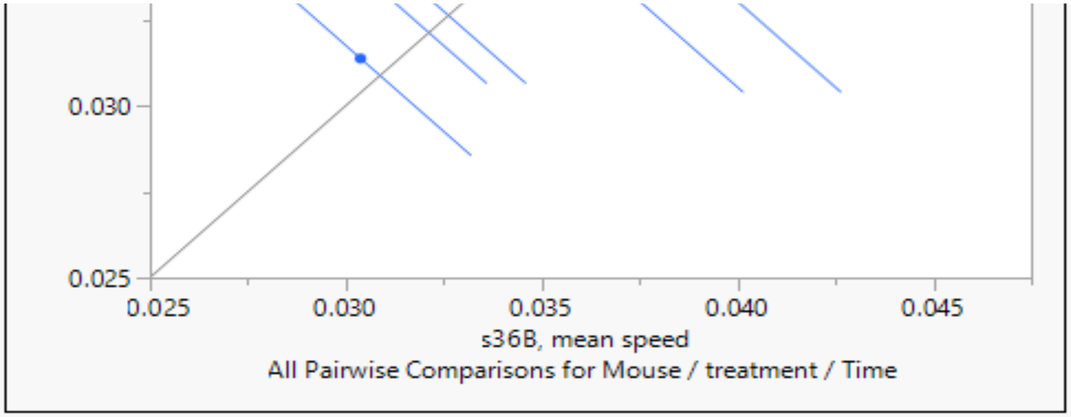
0.030
0.025
0.025
0.030
0.035
0.040
0.045
s36B, mean speed
All Pairwise Comparisons for Mouse / treatment / Time

S-FIG-37D

Fit Mixed Gender=F

Fixed Effects Tests

Source	Nparm	DFNum	DFDen	F Ratio	Prob > F
Mouse	1	1	18.4	14.207694	0.0014*
treatment	1	1	18.4	1.0594298	0.3167
Mouse*treatment	1	1	18.4	0.1125363	0.7411
Time	1	1	16.5	12.18339	0.0029*
Mouse*Time	1	1	16.5	2.2618755	0.1515
treatment*Time	1	1	16.5	1.5983094	0.2237
Mouse*treatment*Time	1	1	16.5	0.0036123	0.9528

Multiple Comparisons for Mouse*treatment*Time

Student's t All Pairwise Comparisons

All Pairwise Differences

Mouse	treatment	Time	-Mouse	-treatment	-Time	Difference	Std Error	t Ratio	Prob>|t|	Lower 95%	Upper 95%
C57	ChABC	After	C57	ChABC	Before	-0.001000	0.0029689	-0.34	0.7410	-0.007341	0.005341
C57	ChABC	After	C57	P	After	0.003833	0.0044506	0.86	0.3958	-0.005251	0.012918
C57	ChABC	After	C57	P	Before	-0.001167	0.0044506	-0.26	0.7950	-0.010251	0.007918
C57	ChABC	After	CNTNAP2	ChABC	After	-0.008484	0.0042600	-1.99	0.0550	-0.017161	0.000193
C57	ChABC	After	CNTNAP2	ChABC	Before	-0.014208	0.0041632	-3.41	0.0018*	-0.022706	-0.005711
C57	ChABC	After	CNTNAP2	P	After	-0.003060	0.0043945	-0.70	0.4910	-0.011992	0.005872
C57	ChABC	After	CNTNAP2	P	Before	-0.012421	0.0043945	-2.83	0.0078*	-0.021353	-0.003488
C57	ChABC	Before	C57	P	After	0.004833	0.0044506	1.09	0.2860	-0.004251	0.013918
C57	ChABC	Before	C57	P	Before	-0.000167	0.0044506	-0.04	0.9704	-0.009251	0.008918
C57	ChABC	Before	CNTNAP2	ChABC	After	-0.007484	0.0042600	-1.76	0.0885	-0.016161	0.001193
C57	ChABC	Before	CNTNAP2	ChABC	Before	-0.013208	0.0041632	-3.17	0.0034*	-0.021706	-0.004711
C57	ChABC	Before	CNTNAP2	P	After	-0.002060	0.0043945	-0.47	0.6423	-0.010992	0.006872
C57	ChABC	Before	CNTNAP2	P	Before	-0.011421	0.0043945	-2.60	0.0138*	-0.020353	-0.002488
C57	P	After	C57	P	Before	-0.005000	0.0029689	-1.68	0.1133	-0.011341	0.001341
C57	P	After	CNTNAP2	ChABC	After	-0.012318	0.0042600	-2.89	0.0068*	-0.020995	-0.003641
C57	P	After	CNTNAP2	ChABC	Before	-0.018042	0.0041632	-4.33	0.0001*	-0.026539	-0.009544
C57	P	After	CNTNAP2	P	After	-0.006893	0.0043945	-1.57	0.1261	-0.015825	0.002039
C57	P	After	CNTNAP2	P	Before	-0.016254	0.0043945	-3.70	0.0008*	-0.025186	-0.007322
C57	P	Before	CNTNAP2	ChABC	After	-0.007318	0.0042600	-1.72	0.0955	-0.015995	0.001359

C57	P	Before	CNTNAP2	ChABC	Before	-0.013042	0.0041632	-3.13	0.0038*	-0.021539	-0.004544
C57	P	Before	CNTNAP2	P	After	-0.001893	0.0043945	-0.43	0.6694	-0.010825	0.007039
C57	P	Before	CNTNAP2	P	Before	-0.011254	0.0043945	-2.56	0.0151*	-0.020186	-0.002322
CNTNAP2	ChABC	After	CNTNAP2	ChABC	Before	-0.005724	0.0027251	-2.10	0.0524	-0.011516	0.000068
CNTNAP2	ChABC	After	CNTNAP2	P	After	0.005425	0.0042013	1.29	0.2049	-0.003097	0.013946
CNTNAP2	ChABC	After	CNTNAP2	P	Before	-0.003936	0.0042013	-0.94	0.3551	-0.012458	0.004585
CNTNAP2	ChABC	Before	CNTNAP2	P	After	0.011149	0.0041031	2.72	0.0102*	0.002813	0.019484
CNTNAP2	ChABC	Before	CNTNAP2	P	Before	0.001788	0.0041031	0.44	0.6658	-0.006548	0.010123
CNTNAP2	P	After	CNTNAP2	P	Before	-0.009361	0.0033816	-2.77	0.0115*	-0.016392	-0.002330

All Pairwise Differences Connecting Letters

Mouse	treatment	Time				Least Squares Mean
CNTNAP2	ChABC	Before	A			0.04937500
CNTNAP2	P	Before	A			0.04758729
CNTNAP2	ChABC	After	A	B		0.04365099
CNTNAP2	P	After		B	C	0.03822635
C57	P	Before		B	C	0.03633333
C57	ChABC	Before		B	C	0.03616667
C57	ChABC	After		B	C	0.03516667
C57	P	After			C	0.03133333

Levels not connected by same letter are significantly different.
